# Non-coding RNAs identification and regulatory networks in pathogen-host interaction in the microsporidia congenital infection

**DOI:** 10.1186/s12864-023-09490-3

**Published:** 2023-07-26

**Authors:** Zigang Shen, Qiong Yang, Lie Luo, Tangxin Li, Zhuojun Ke, Tian Li, Jie Chen, Xianzhi Meng, Heng Xiang, Chunfeng Li, Zeyang Zhou, Ping Chen, Guoqing Pan

**Affiliations:** 1 State Key Laboratory of Resource Insects, Southwest University, Tiansheng Street, Chongqing, 400715 People’s Republic of China; 2 College of Sericulture, Textile and Biomass Sciences, Southwest University, Chongqing, People’s Republic of China; 3 Chongqing Key Laboratory of Microsporidia Infection and Control, Southwest University, Chongqing, People’s Republic of China; 4 Sericulture and Agri-Food Research Institute, Guangdong Academy of Agricultural Sciences, Guangzhou, People’s Republic of China; 5 College of Animal Science and Technology, Southwest University, Chongqing, People’s Republic of China; 6 College of Life Sciences, Chongqing Normal University, Chongqing, People’s Republic of China

**Keywords:** Congenital infection, Microsporidia, *Nosema bombycis*, Non-coding RNAs, Regulatory networks, Host–pathogen interaction

## Abstract

**Background:**

The interaction networks between coding and non-coding RNAs (ncRNAs) including long non-coding RNA (lncRNA), covalently closed circular RNA (circRNA) and miRNA are significant to elucidate molecular processes of biological activities and interactions between host and pathogen. Congenital infection caused by vertical transmission of microsporidia *N. bombycis* can result in severe economic losses in the silkworm-feeding industry. However, little is known about ncRNAs that take place in the microsporidia congenital infection. Here we conducted whole-transcriptome RNA-Seq analyses to identify ncRNAs and regulatory networks for both *N. bombycis* and host including silkworm embryos and larvae during the microsporidia congenital infection.

**Results:**

A total of 4,171 mRNAs, 403 lncRNA, 62 circRNAs, and 284 miRNAs encoded by *N. bombycis* were identified, among which some differentially expressed genes formed cross-talk and are involved in *N. bombycis* proliferation and infection. For instance, a lncRNA/circRNA competing endogenous RNA (ceRNA) network including 18 lncRNAs, one circRNA, and 20 miRNAs was constructed to describe 14 key parasites genes regulation, such as polar tube protein 3 (*PTP3*), ricin-B-lectin, spore wall protein 4 (*SWP4*), and heat shock protein 90 (*HSP90*). Regarding host silkworm upon *N. bombycis* congenital infection, a total of 14,889 mRNAs, 3,038 lncRNAs, 19,039 circRNAs, and 3,413 miRNAs were predicted based on silkworm genome with many differentially expressed coding and non-coding genes during distinct developmental stages. Different species of RNAs form interacting network to modulate silkworm biological processes, such as growth, metamorphosis and immune responses. Furthermore, a lncRNA/circRNA ceRNA network consisting of 140 lncRNAs, five circRNA, and seven miRNAs are constructed hypothetically to describe eight key host genes regulation, such as *Toll-6*, *Serpin-6*, inducible nitric oxide synthase (*iNOS*) and *Caspase-8*. Notably, cross-species analyses indicate that parasite and host miRNAs play a vital role in pathogen-host interaction in the microsporidia congenital infection.

**Conclusion:**

This is the first comprehensive pan-transcriptome study inclusive of both *N. bombycis* and its host silkworm with a specific focus on the microsporidia congenital infection, and show that ncRNA-mediated regulation plays a vital role in the microsporidia congenital infection, which provides a new insight into understanding the basic biology of microsporidia and pathogen-host interaction.

**Supplementary Information:**

The online version contains supplementary material available at 10.1186/s12864-023-09490-3.

## Introduction

Microsporidia are a group of fungi-related obligate intracellular parasites that infect most animal groups including humans [1, 2]. As the first reported microsporidia, *Nosema bombycis* was described by Nageli in 1857. As of now, more than 1,500 species belonging to 197 genera have been reported [3], and  700 species are parasitic on insects alone [4]. Microsporidia are capable of vertical transmission, *N. bombycis* transovarial transmission can cause a highly fatal silkworm disease (pébrine). Therefore, *N. bombycis* is the only mandatory inspection target for silkworm egg production by silkworm feeding countries.

It has been known that ncRNAs, including miRNAs, lncRNAs, and circRNAs play an important role in many biological processes via regulating gene expression at both transcriptional and post-transcriptional levels [5–7]. MiRNAs, usually referred to as small ncRNAs ranging in size from 19 to 22 nucleotides, are well conserved in eukaryotic organisms. They are thought to be a class of critical post-transcriptional regulators to play a vital role in host–pathogen interaction. In the nucleus, primary miRNA transcripts (pri-miRNAs) are processed by the microprocessor complex, consisting of an RNA binding protein DiGeorge Syndrome Critical Region (DGCR8) and a ribonuclease III enzyme, Drosha, and generate precursor miRNAs (pre-miRNAs) [8]. The pre-miRNAs are subsequently cleaved by Dicer to produce an ∼22-nt miRNA/miRNA^*^ duplex [9]. Although both strands of the duplex are necessarily produced in equal amounts by transcription, their accumulation is asymmetric at the steady state. By convention, the most abundant duplex strand is defined as the mature miRNA strand, whereas the less abundant strand is known as the miRNA^*^ strand [10]. For example, *Mycobacteria tuberculosis* H37Rv induced an increase in miR-33 and miR-33^*^ expression in peritoneal macrophages to inhibit autophagy and enhance host lipid metabolism to facilitate their survival [11]. In addition, *B. bassiana* deploys bba-milR1 to modulate mosquito immunity by targeting the mosquito genes Spätzle4 (*Spz4*) and CLIP-domain serine protease (*CLIPB9*) [12]. RNA interference (RNAi) and related RNA silencing pathways are widespread in animals, plants, fungi, and protozoans, and control gene silencing in all living cells [13]. RNAi relies on small noncoding RNAs, such as miRNAs and siRNAs. Two proteins, Dicer and Argonaute (Ago), are responsible for small noncoding RNA biogenesis. Among microsporidia, Huang et al. showed that 11 species including *N. apis*, *N. ceranae* and *N. bombycis* maintain both Dicer and Ago orthologs [14], suggesting that those microsporidia retained the canonical RNAi signaling pathway. A few studies have revealed that miRNAs played an essential role in *N. bombycis*-silkworm interactions [15, 16]*.* However, the identification of miRNAs in *N. bombycis*-silkworm interactions, especially in the congenital infection of *N. bombycis* in silkworm including embryo and larvae stages, still remain elusive.

LncRNAs are a novel class of ncRNAs with nucleotides from 200 bp to several kilobases in size. Structurally, lncRNAs are similar to mRNAs, and are subject to post-transcriptional modifications such as capping, polyadenylation, and splicing. Unlike miRNAs, lncRNAs are generally less conserved in eukaryotic organisms [17]. LncRNAs can compete with endogenous RNAs, acting as miRNA sponges to reduce their regulatory effect on target mRNAs involved in various of biological processes [18, 19]. In microsporidia, Guo et al. have reported that 83 novel lncRNAs including 59 antisense lncRNAs, 21 long intergenic noncoding RNAs, and three sense lncRNAs produced by *N. ceranae* [20]. In addition, Wu et al. have described that 1,180 lncRNAs were identified in the silkworm, including 6,250 intergenic lncRNAs, 474 intronic lncRNAs and 5,086 natural antisense lncRNAs [21]. Chen et al. have demonstrated that lncRNAs and their regulatory networks were involved in the host honeybee response to *N. ceranae* infection [22]. Furthermore, one lncRNA lncRNA4.2 can act as a decoy and titrate away dimerization of Toll, resulting in dysregulation of the Toll signaling pathway in silkworm after virus infection [23]. Up to date, the function of *N. bombycis* and silkworm lncRNAs in host–pathogen interactions, especially in the congenital infection of *N. bombycis* in silkworm is still obscure.

CircRNAs are another novel class of ncRNAs with a special structure, which is characterized with a covalent closed-loop without 5’ caps or 3’ poly (A) tails. Those circRNAs are produced from back-splicing of precursor mRNA (pre-mRNA) in eukaryotes, and are generally expressed at low levels [24]. Many circRNAs are more abundant than their linear counterparts [25], and their expression levels are generally tissue-specific and cell-type-specific [26]. There is increasing evidence that circRNAs function as sponges for miRNAs [27, 28], regulating transcription, splicing [29], or polypeptide production [30, 31]. In microsporidia, 204 circRNAs produced by *N. ceranae* have been identified by using deep sequencing technology, of which include 174 exonic circRNAs and 30 intergenic circRNAs [32]. Furthermore, ceRNA regulatory networks were constructed, and 15 *N. ceranae* circRNAs were found to act as sponges of the corresponding three miRNAs (Nce-miR9769, Nce-miR20213, and Nce-miR34537). However, current research on microsporidia and host circRNAs mainly focuses on circRNAs identification, and the studies on the function of circRNAs in host-microsporidia interactions are scant.

To obtain a better understanding of various species of ncRNAs in *N. bombycis* and its host *B. mori*, we have performed small RNA and strand-specific library construction for high-throughput RNA sequencing and genome-wide analysis of the coding and noncoding RNAs in the *N. bombycis* congenitally infected and uninfected silkworm embryos of 5-day, and larvae of 1, 5, 10-day, respectively. The expression and cross-talk of mRNA, miRNA, lncRNA, and circRNA were analyzed. Based on ceRNA hypothesis, the lncRNA/circRNA-miRNA- mRNA networks were constructed to understand the functional effects of lncRNAs/circRNAs, through sponging miRNAs. Furthermore, we predicted that parasite and host genes were potentially targeted by parasite and/or silkworm miRNAs. This is the first pan-transcriptome analysis of miRNA, lncRNA, circRNA and mRNA expression profiles and their cross-talks in the embryos and larvae of insects following the congenital microsporidia infection, which provides a new insight into understanding the basic biology of microsporidia and pathogen-host interaction.

## Materials and methods

### Silkworm embryos and larva congenitally infected by *N. bombycis*

The silkworm 305 strain was reared at 26℃ with a 70% relative humidity and 12-h light/dark cycle. The *N. bombycis* isolate CQ1 (Chongqing, China) was conserved in the China Veterinary Culture Collection Center (CVCC No. 102059). The silkworms were orally challenged by mature spores with approximately 10^6^ spores per larva at the fifth instar larvae and were then reared under the above feeding conditions until cocoon. Surviving pupae hatched into adults and fertilized female adult laid progeny eggs. Lastly, the progeny eggs were collected and hatched, and hatched larvae were reared under the above-mentioned feeding conditions until the 10^th^ day of larvae.

### Embryos and larvae collection

To confirm the congenital *N. bombycis* infection in silkworm, eggs produced by mothers infected with *N. bombycis* were hatched. The hatched eggs of 5-day and larvae of 1, 5, 10-day were collected to detect the presence of spores by hematoxylin & eosin (H&E) staining and transmission electron microscopy (TEM). In addition, to sequence the whole transcriptome, a 0.6 g hatched eggs of 5-day and larvae of 1, 5, 10-day from either the *N. bombycis* congenitally infected or uninfected control groups were collected.

### H&E staining

The collected eggs were immersed in 10% potassium hydroxide solution (w/v) at room temperature for 7 min to soften cell walls followed by fixing with Smith fixative (0.5 g potassium dichromate, 2.5 mL glacial acetic acid, 10 mL methanol, 87.5 mL distilled water) for 12 h. The silkworm tissues were dehydrated in a series of ethanol and then embedded in paraffin and cross-sectioned into 5–7 μm slices. After deparaffination, the sections were treated with xylene and washed with distilled water. Lastly, the sections were stained with H&E straining according to the manufacturer’s protocol, and the sections were observed under a light microscope.

### Transmission electron microscopy

The collected silkworm embryos and larvae were fixed with the 2.5% glutaraldehyde 0.1 M phosphate buffer saline (PBS) solution. The sections were cut using an ultramicrotome (Lecia, Wetzlar, Germany) and placed on nickel grids. The sections were stained in 3% uranyl acetate, followed by lead citrate. Lastly, the sections were washed three times in ddH2O, and photographed with a JEM-1400 Plus TEM at an accelerating voltage of 80 kV.

### Experimental designs

Non-infected (normal) eggs were placed in an incubator at -26℃. On the fifth day, a certain number of eggs were taken out and divided into three samples, designated NI-E5-1 (non-infected embryos day 5 replicate 1), NI-E5-2 (non-infected embryos day 5 replicate 2), and NI-E5-3 (non-infected embryos day 5 replicate 3). The remaining eggs were placed and hatched into larvae in an incubator. On the first day of larvae, a certain number of larvae were taken out and divided into three samples, designated NI-L1-1 (non-infected larvae day 1 replicate 1), NI-L1-2 (non-infected larvae day 1 replicate 2), and NI-L1-3 (non-infected larvae day 1 replicate 3). On the fifth day of larvae, a certain number of larvae were taken out and divided into three samples, designated NI-L5-1 (non-infected larvae day 5 replicate 1), NI-L5-2 (non-infected larvae day 5 replicate 2), and NI-L5-3 (non-infected larvae day 5 replicate 3). On the tenth day of larvae, a certain number of larvae were taken out and divided into three samples, designated NI-L10-1 (non-infected larvae day 10 replicate 1), NI-L10-2 (non-infected larvae day 10 replicate 2), and NI-L10-3 (non-infected larvae day 10 replicate 3). Similarly, *N. bombycis*-infected eggs were also placed in an incubator at -26℃。Infected-samples were taken at the same way as non-infected samples, named I-E5-1 (infected embryos day 5 replicate 1), I-E5-2 (infected embryos day 5 replicate 2), I-E5-3 (infected embryos day 5 replicate 3), I-L1-1 (infected larvae day 1 replicate 1), I-L1-2 (infected larvae day 1 replicate 2), I-L1-3 (infected larvae day 1 replicate 3), I-L5-1 (infected larvae day 5 replicate 1), I-L5-2 (infected larvae day 5 replicate 2), I-L5-3 (infected larvae day5replicate 3), I-L10-1 (infected larvae day 10 replicate 1), I-L10-2 (infected larvae day 10 replicate 2), and I-L10-3 (infected larvae day 10 replicate 3), respectively. A total of 24 samples were sequenced and analyzed.

### RNA extraction, library construction and sequencing

Three replicates were included at each time point, and each sample included 0.6 g eggs or larvae. RNA extraction, library construction, and RNA-sequencing were carried out by the Gene Denovo Biotechnology Co. (Guangzhou, China). Total RNA was extracted using TRIzol reagent (Invitrogen, CA, USA) according to the manufacturer’s protocols. The integrity and quality of RNA were assessed using the Agilent 2100 Bioanalyzer (Agilent Technologies, CA, USA) and checked using RNase free agarose gel electrophoresis. For strand-specific cDNA libraries, rRNAs were removed to retain mRNAs and ncRNAs using Ribo-Zero rRNA removal kit (H/M/R) (Illumina, San Diego, USA) according to the manufacturer’s protocols. The enriched mRNAs and ncRNAs were fragmented into short fragments by using fragmentation buffer and reverse transcribed into cDNA with random primers. Second-strand cDNA were synthesized by DNA polymerase I, RNase H, dNTP (dUTP instead of dTTP) and buffer. Next, the cDNA fragments were purified with QIAquick polymerase chain reaction (PCR) extraction kit (Qiagen, Venlo, The Netherlands), end repaired, A base added, and ligated to Illumina sequencing adapters. Then UNG (Uracil-N-Glycosylase) were used to digest the second-strand cDNA. The digested products were size selected by agarose gel electrophoresis, PCR amplified, and sequenced to perform pair-end sequencing using Illumina HiSeq™ 4000 by Gene Denovo Biotechnology Co. (Guangzhou, China). For small RNA cDNA library, after total RNA was extracted by Trizol reagent kit (Invitrogen, CA, USA), the RNA molecules in a size range of 18–30 nt were purified from total RNA on a urea polyacrylamide gel electrophoresis (PAGE). The purified small RNA was ligated with 3’ RNA adaptor to add a 3’ hydroxyl group. The 5’ adapters were then ligated to the RNAs as well. Reverse transcription was done to convert the RNA to cDNA, which was then selectively enriched by 12 cycles of PCR. The PCR products were purified on PAGE in a size range of 140 to 160 bp. Libraries were sequenced to perform single-end sequencing using Illumina HiSeq TM 2500 by Gene Denovo Biotechnology Co. (Guangzhou, China).

### Bioinformatics analysis

For mRNAs and lncRNAs, reads obtained from the sequencing machines included raw reads containing adapters or low-quality bases which would affect the following assembly and analysis. Thus, to get high quality clean reads, reads were further filtered by fastp (version 0.18.0) [33]. The parameters were as follows: 1) removing reads containing adapters; 2) removing reads containing more than 10% of unknown nucleotides (N); 3) removing low quality reads containing more than 50% of low quality (Q-value ≤ 20) bases. High-quality reads were aligned with reference *N. bombycis* (http://silkpathdb.swu.edu.cn/datasets) or silkworm (https://silkdb.bioinfotoolkits.net /main/species-info/-1) genome using HISAT2 (version 2.1.0) [34]. Then, guided by the Ensembl gene annotation file, the StringTie software program [35] was used to the reconstruction of transcripts. Differential gene expression and transcript abundance (expressed as fragment per kilobase of transcript per million mapped reads (FPKM) values) were calculated using the RESM program [36]. Accordingly, absolute a fold change ≥ 2 and a* P* value < 0.05 were calculated based on FPKM, and differentially expressed (DE) mRNAs and lncRNAs were identified. All DE mRNAs were mapped to GO terms (http://www.geneontology.org/) and Kyoto Encyclopedia of Genes and Genomes (KEGG) pathway [37–39]. LncRNA target genes were predicted by locations in relation to nearby genes, and KEGG pathway analyses were carried out on these target genes [37–39]. In addition, we performed lncRNA antisense and trans-regulation analysis.

For circRNAs, 20 mers from both ends of the unmapped reads were extracted, and aligned to the reference genomes to identify unique anchor positions within the splice site using the methods described by Memczak et al. [28], respectively. Then anchor reads that aligned in the reverse orientation indicated circRNA splicing was subjected to Find_circ software [28] to identify circRNAs. To identify differentially expressed circRNAs across samples or groups, the edgeR package (http://www.rproject.org/) was used. We identified circRNAs with a fold change ≥ 2 and a* P* value < 0.05 in a comparison between samples or groups as significant differentially expressed circRNAs. KEGG pathway analyses were performed for the DE circRNA-associated genes [37–39].

For miRNAs, reads obtained from the sequencing machines included dirty reads containing adapters or low-quality bases which would affect the following assembly and analysis. Low-quality sRNA reads were first filtered form the raw sequences. Several types of impurities such as 3’ adaptor null reads, insert null reads, and 5’ adaptor contaminants, were removed from the remaining sRNA reads. Subsequently, reads smaller than 18 nt or having Poly (A) tails were removed from the high-quality sRNA reads. Lastly, all of the clean tags were aligned with small RNAs in GenBank database (Release 209.0) [40] to identify and remove rRNA, scRNA, snoRNA, snRNA and tRNA. Meanwhile all of the clean tags were aligned with small RNAs in Rfam database (Release 11.0) [41] to identify and remove rRNA, scRNA, sonRNA, snRNA and tRNA. All of the clean tags were also aligned with reference genomes. Those mapped to exons or introns might be fragments from mRNA degradation, so these tags were removed. The tags mapped to repeat sequences were also removed. All of the clean tags were then searched against miRbase database (Release 22) [42] to identify exist miRNAs or known miRNAs. Additionally, all of the unannotated data were aligned with *N. bombycis* or silkworm genome. According to their genome positions and hairpin structures predicted by software MiReap_v0.2, the novel miRNA candidates were identified. We identified miRNAs with a fold change ≥ 2 and a* P* value < 0.05 in a comparison as DE miRNAs. Based on the sequences of the miRNAs, the candidate target genes were predicted by RNAhybrid [43], Miranda [44] and TargetScan [45] with default settings. KEGG pathway analyses were performed for the DE miRNA-targeted genes [37–39].

### Analysis of the ceRNA (lncRNA/circRNA) regulatory network

CeRNA regulatory networks were constructed based on the overlapping miRNA target between the mRNA and lncRNA/circRNA. The nodes in the networks include lncRNAs/circRNA, miRNAs, and mRNAs. The lncRNAs/circRNA-miRNA-mRNA regulatory networks were visualized with Cytoscape (v3.6.0) ( http://www.cytoscape.org/).

### Statistical analysis

Statistical significance was assessed by one-way ANOVA for comparisons among multiple groups. Statistical significance was defined as *P* value < 0.05.

## Results

### Validation of the congenital *N. bombycis* infection in silkworm embryos and larvae

To confirm the congenital *N. bombycis* infection in silkworm, embryos of 5-day from the parent silkworms infected with *N. bombycis* were used to detect the microsporidia spores by H&E staining. A few *N. bombycis* spores appeared at the region of the yolk granules (Fig. S1A). Histological examination of larvae of silkworm reared in isolation on 1, 5 and 10-day revealed that a number of spores were present in various cells such as muscle cells, adipose cells and epidermis cells (Fig. S1B-D). The presence of *N. bombycis* spores in silkworms derived from congenital infection was further confirmed by TEM. Consistent with H&E staining, microsporidia spores were mainly around the yolk granules in embryos of 5-day as revealed by TEM (Fig. S1E). On larvae of 1-day, spores were predominantly present in the midgut of silkworms (Fig. S1F) and spread to fat body and skin tissues on 5 and 10-day larvae (Fig. S1G, H). In contrast, no spores were observed in the 5-day embryos and 1, 5, 10-day larvae from parent silkworms uninfected with *N. bombycis*.

### The summary of the whole transcriptome sequencing

To investigate the RNA transcripts of host and pathogen at different developmental stages, the specimen including 5-day infected and uninfected embryos, 1, 5, 10-day infected and uninfected larvae were collected and processed for RNA-seq analysis using *N. bombycis* and *B. mori* genome as references. For strand-specific cDNA libraries, each of 24 samples tested in the platform produced an average of 27.43 Gb of data. In the infected eggs and larvae, with 1.55% to 6.91% of these reads mapped to the *N. bombycis* genome, and in the uninfected eggs and larvae, there was negligible reads that can be mapped to the *N. bombycis* genome (Table 1). For small RNA libraries, the sequencing analysis also found negligible tags that can be mapped to the *N. bombycis* genome in the uninfected eggs and larvae, and 23.02% to 56.73% of tags can be mapped to the *N. bombycis* genome in the infected eggs and larvae (Table 2). These data showed that the experimental materials and whole transcriptome sequencing have good reliability.

**Table 1:** Summary of the strand-specific cDNA libraries sequencing in whole transcriptome

Samples	Total reads	Unique Mapped (*B. mori*)	Unique_Mapped (*N. bombycis*)	Reads ratio (parasite/host)	Q30
NI-E5-1	140058926	10883736(77.71%)	52644 (0.04%)	0.05%	97.83%
NI-E5-2	132179004	105171708(79.57%)	42462 (0.03%)	0.04%	97.78%
NI-E5-3	154367904	123593975(80.06%)	50727 (0.03%)	0.04%	97.79%
I-E5-1	134841582	102893581 (76.31%)	2379283 (1.76%)	2.31%	97.84%
I-E5-2	128693762	97181765 (75.51%)	1991059 (1.55%)	2.05%	97.86%
I-E5-3	184873794	137942661 (74.61%)	2966177 (1.60%)	2.15%	97.94%
NI-L1-1	133407530	110497135 (82.83%)	42325 (0.03%)	0.04%	97.84%
NI-L1-2	166036466	137202200 (82.63%)	43398 (0.03%)	0.03%	98.10%
NI-L1-3	154418918	126570961 (81.97%)	41816 (0.03%)	0.03%	97.8%
I-L1-1	116250514	89236733 (76.76%)	3438194 (2.96%)	3.85%	97.85%
I-L1-2	159468356	120839202 (75.78%)	4225884 (2.65%)	3.50%	97.88%
I-L1-3	139668848	107348301 (76.86%)	4135813 (2.96%)	3.85%	97.80%
NI-L5-1	133719228	108935406 (81.47%)	27207 (0.02%)	0.02%	98.13%
NI-L5-2	184218162	150306946 (81.59%)	74902 (0.04%)	0.05%	98.10%
NI-L5-3	134570248	110797829 (82.33%)	21183 (0.02%)	0.02%	98.12%
I-L5-1	122534234	89108792 (72.72%)	6935455 (5.66%)	7.78%	97.78%
I-L5-2	187390432	136464116 (72.82%)	12951556(6.91%)	9.49%	98.02%
I-L5-3	152617240	112612509 (73.79%)	7110884 (4.66%)	6.31%	98.05%
NI-L10-1	153990740	117009774 (75.98%)	44195 (0.03%)	0.04%	97.94%
NI-L10-2	146599288	111144277 (75.82%)	82669 (0.06%)	0.07%	98.07%
NI-L10-3	127527200	96669928 (75.80%)	19775 (0.02%)	0.02%	97.87%
I-L10-1	141422932	104326489 (73.77%)	4265627 (3.02%)	4.09%	98.01%
I-L10-2	129004988	91673388 (71.06%)	4373382 (3.39%)	4.77%	97.97%
I-L10-3	132129234	91628241 (69.35%)	8727149 (6.61%)	9.52%	97.94%

Three replicates were applied at all of time points of uninfected and infected samples. Total reads refer total clean reads including host and parasite. Mapped reads refer reads can be mapped to *N. bombycis* or silkworm genome. Reads ratio refers *N. bombycis* reads divide silkworm reads

**Table 2:** Summary of the small RNA libraries sequencing in whole transcriptome

Samples	Total tags	Mapped tags (*B. mori*)	Mapped tags (*N. bombycis*)	Tags ratio(parasite/host)
NI-E5-1	1809583	12131745 (87.85%)	26827 (0.19%)	0.22%
NI-E5-2	10584907	9126448 (86.22%)	13871 (0.13%)	0.15%
NI-E5-3	12086145	10477844 (86.69%)	20727 (0.17%)	0.20%
I-E5-1	12839414	5609336 (43.69%)	4761066 (37.08%)	84.88%
I-E5-2	14159566	6368998 (44.98%)	5222428 (36.88%)	82.00%
I-E5-3	11837155	5041904 (42.59%)	4596470 (38.83%)	91.17%
NI-L1-1	9772396	7980968 (81.67%)	47510 (0.49%)	0.60%
NI-L1-2	13683260	10622680 (77.63%)	220540 (1.61%)	2.08%
NI-L1-3	11701558	9036998 (77.23%)	38081 (0.33%)	0.42%
I-L1-1	9754743	4429750 (45.41%)	2322325 (23.81%)	52.43%
I-L1-2	10607820	4807451 (45.32%)	2441992 (23.02%)	50.80%
I-L1-2	8831025	3462318 (39.21%)	2946789 (33.37%)	85.11%
NI-L5-1	12135683	3305586 (27.24%)	47474 (0.39%)	1.44%
NI-L5-2	13179834	3008785 (22.83%)	78851 (0.60%)	2.62%
NI-L5-3	12799412	2660845 (20.79%)	44164 (0.35%)	1.66%
I-L5-1	12614602	1500769 (11.90%)	7079881 (56.12%)	471.75%
I-L5-2	10053638	1014524 (10.09%)	5340257 (53.12%)	526.38%
I-L5-3	9822926	1031901 (10.51%)	5572322 (56.73%)	540.01%
NI-L10-1	10841051	1938008 (17.88%)	118100 (1.09%)	6.09%
NI-L10-2	11767281	2367569 (20.12%)	104389 (0.89%)	4.41%
NI-L10-3	9174235	1781290 (19.42%)	34160 (0.37%)	1.92%
I-L10-1	14633132	932937 (6.38%)	7828950 (53.50%)	839.17%
I-L10-2	10841706	742098 (6.84%)	5849551 (53.95%)	788.25%
I-L10-3	11269705	1322587 (11.74%)	6244127 (55.41%)	472.11%

Three replicates were applied at all of time points of uninfected and infected samples. Total tags refer total clean tags including host and parasite. Mapped tags refer tags can be mapped to *N. bombycis* or silkworm genome. Tags ratio refers *N. bombycis* tags divide silkworm tags

### Transcriptome-wide identification of *N. bombycis* mRNAs, lncRNAs, circRNAs and miRNAs in congenitally infected silkworm embryos and larvae

As shown in Fig. S2, we performed principal component analysis (PCA) based on the original mRNA, lncRNA, circRNA, and miRNA data. For mRNA data, PCA demonstrated a clear separation between infected groups and uninfected groups at the four different stages, and the distance between infected and uninfected groups at the fifth day of embryos of silkworms was relatively short (Fig. S2A). Similarly, based on lncRNA data, the infected groups and uninfected samples at different stages were distinguished by PCA (Fig. S2B). For circRNA data, PCA could not show an unambiguous separation between infected groups and uninfected groups at the fifth day of embryos and first day of larvae of silkworms, and a clear separation between infected groups and uninfected groups was present at the fifth and tenth day of larvae (Fig. S2C). For miRNA data, PCA showed that the overall distance between different groups was relatively long, indicating that there were major differences among samples (Fig. S2D).

**Fig. 1:**
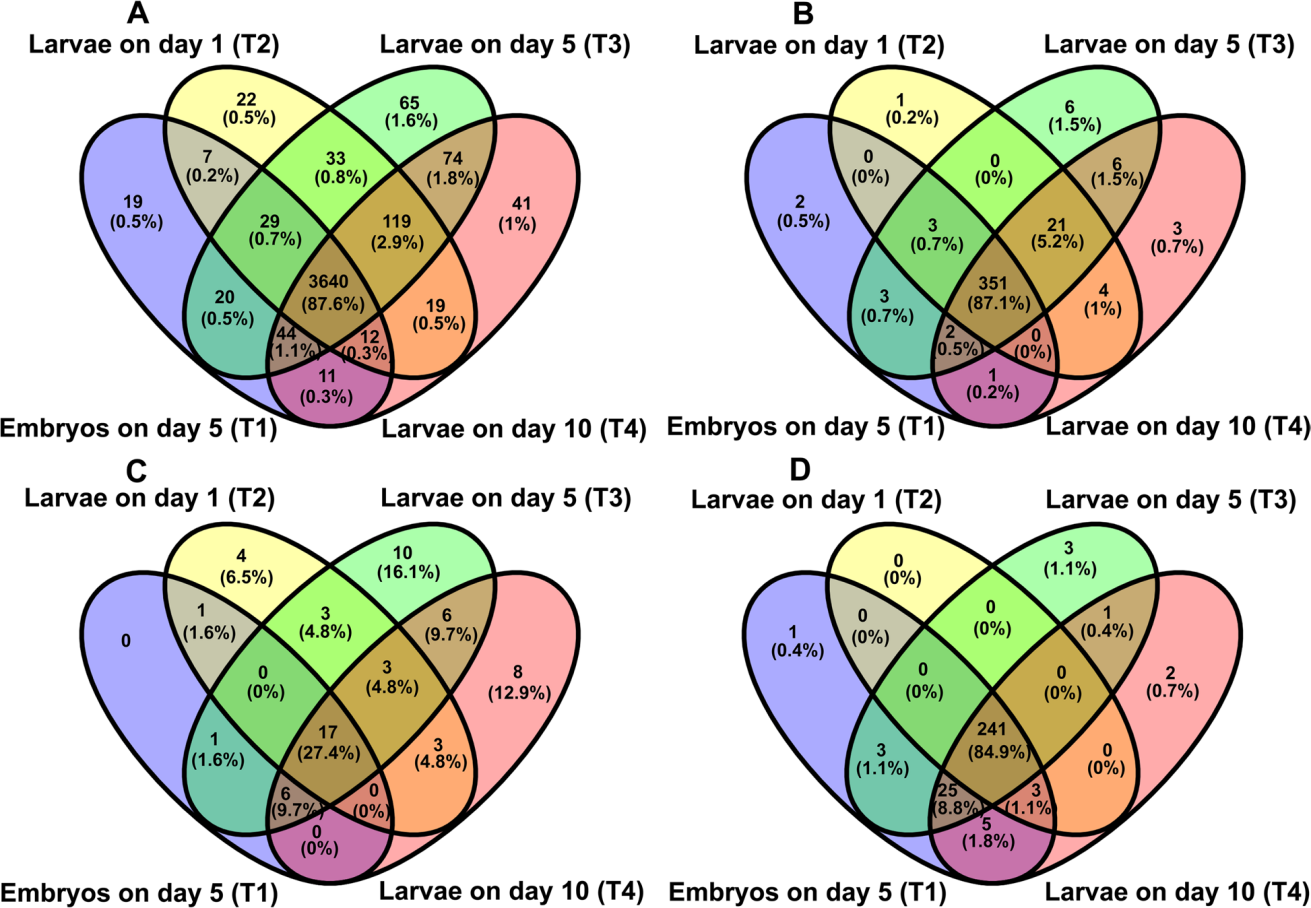
Venn diagram showing the number of exclusive and shared expressed mRNAs, lncRNAs, circRNAs and miRNAs of microsporidia between 5-day embryos, 1-day larvae, 5-day larvae, and 10-day larvae challenged by *N. bombycis* congenital infection. **A**: The Venn diagram of *N. bombycis* mRNA expression; **B**: The Venn diagram of *N. bombycis* lncRNA expression; **C**: The Venn diagram of *N. bombycis* circRNA expression; **D**: The Venn diagram of *N. bombycis* miRNA expression. T1: 5-day embryos with *N. bombycis* congenital infection; T2: 1-day larvae with *N. bombycis* congenital infection; T3: 5-day larvae with *N. bombycis* congenital infection; T4: 10-day of larvae with *N. bombycis* congenital infection

We subsequently analyzed four RNA molecules (mRNA, lncRNA, circRNA and miRNAs) of *N. bombycis*. For *N. bombycis* mRNAs, a total of 4,051 mRNAs were aligned to reference genes and 104 mRNAs were defined as novel genes (Table S1). Additionally, the total numbers of the identified *N. bombycis* mRNA genes at different developmental stages were comparable with 3,782, 3,881, 4,024, and 3,960 mRNA genes in 5-day embryos (T1 stage), 1-day (T2 stage), 5-day (T3-stage), 10-day (T4 stage) larvae, respectively (Table 3), and 3,640 genes were identified in all four above mentioned stages and only 19, 22, 65, and 41 genes were stage-specifically expressed genes based on Venn diagram analysis (Fig. 1A and Table S2), indicating that *N. bombycis* mRNAs expression was not substantially affected by host growth and development.

**Table 3:** The coding RNA (mRNA) and non-coding RNAs(lncRNA, circRNA and miRNA) identification of *N. bombycis* in *N. bombycis* congenitally infected silkworm embryos and larvae

Stage RNA types	5-day Embryos	1-day Larvae	5-day Larvae	10-day Larvae	Sum
mRNA	3782	3881	4024	3960	4155
lncRNA	362	380	392	388	403
circRNA	25	31	46	43	62
miRNA	278	244	273	277	284

With regard to the *N. bombycis* lncRNAs, 403 putative novel lncRNAs were identified that can be classified into five categories according to their genomic locations relative to protein coding genes: 8 sense lncRNAs, 166 antisense lncRNAs, 17 bidirectional lncRNAs, 168 intergenic lncRNAs and 44 other lncRNAs. There were 362, 380, 392, and 388 lncRNAs that were predicted in T1 (5-day embryos), T2 (1-day larvae), T3 (5-day larvae), and T4 (10-day larvae) stages, respectively (Table 3). When comparing lncRNAs profiles at different stages, we found that only two, one, six and three lncRNAs were uniquely expressed at T1, T2, T3 and T4 stage, respectively, and 352 lncRNAs were shared by all four stages, which account for 87.6% of all predicted *N. bombycis* lncRNAs (Fig. 1B).

A total of 62 novel circRNAs were predicted in the *N. bombycis* specimens collected in our study, of which 25, 31, 46, and 43 circRNAs were detected in T1 (5-day embryos), T2 (1-day larvae), T3 (5-day larvae), and T4 (10-day larvae) stages, respectively (Table 3). For circRNA type, 62 circRNAs can be classified into five categories: 9 antisense circRNAs, 16 exon_intron circRNAs, 12 intergenic circRNAs and 25 one_exon circRNAs. 62 circRNAs remained with sizes ranging from 129 to 37,391 bp. Interestingly, only 17 *N. bombycis* circRNAs were identified in all four stages, accounting for 27.4% of all predicted *N. bombycis* circRNAs identified in this study. Zero, four, ten, and eight circRNAs were uniquely expressed in T1, T2, T3, and T4 stages, respectively (Fig. 1C), suggesting that the expression pattern of circRNAs was different from that of mRNAs and lncRNAs, and *N. bombycis* circRNAs expression was more likely to be affected by host development pressure.

Regarding miRNA, a total of 284 miRNAs were identified based on the *N. bombycis* genome, and there were 277, 244, 273, and 277 miRNAs that were detected in T1 (5-day embryos), T2 (1-day larvae), T3 (5-day larvae), and T4 (10-day larvae) stages, respectively (Table 3), with 241 miRNAs being identified in all four stages (Fig. 1D). Consistent with expression profile of mRNAs and lncRNAs, only a small fraction of miRNA is stage specific. One, zero, three, and two miRNAs were uniquely expressed in T1, T2, T3, and T4 stages, respectively. In addition, all 284 miRNAs can be classified into intergenic miRNAs.

### Expression profiles analysis of *N. bombycis* mRNAs, lncRNAs, circRNAs and miRNAs in congenitally infected silkworm embryos and larvae

**Fig. 2:**
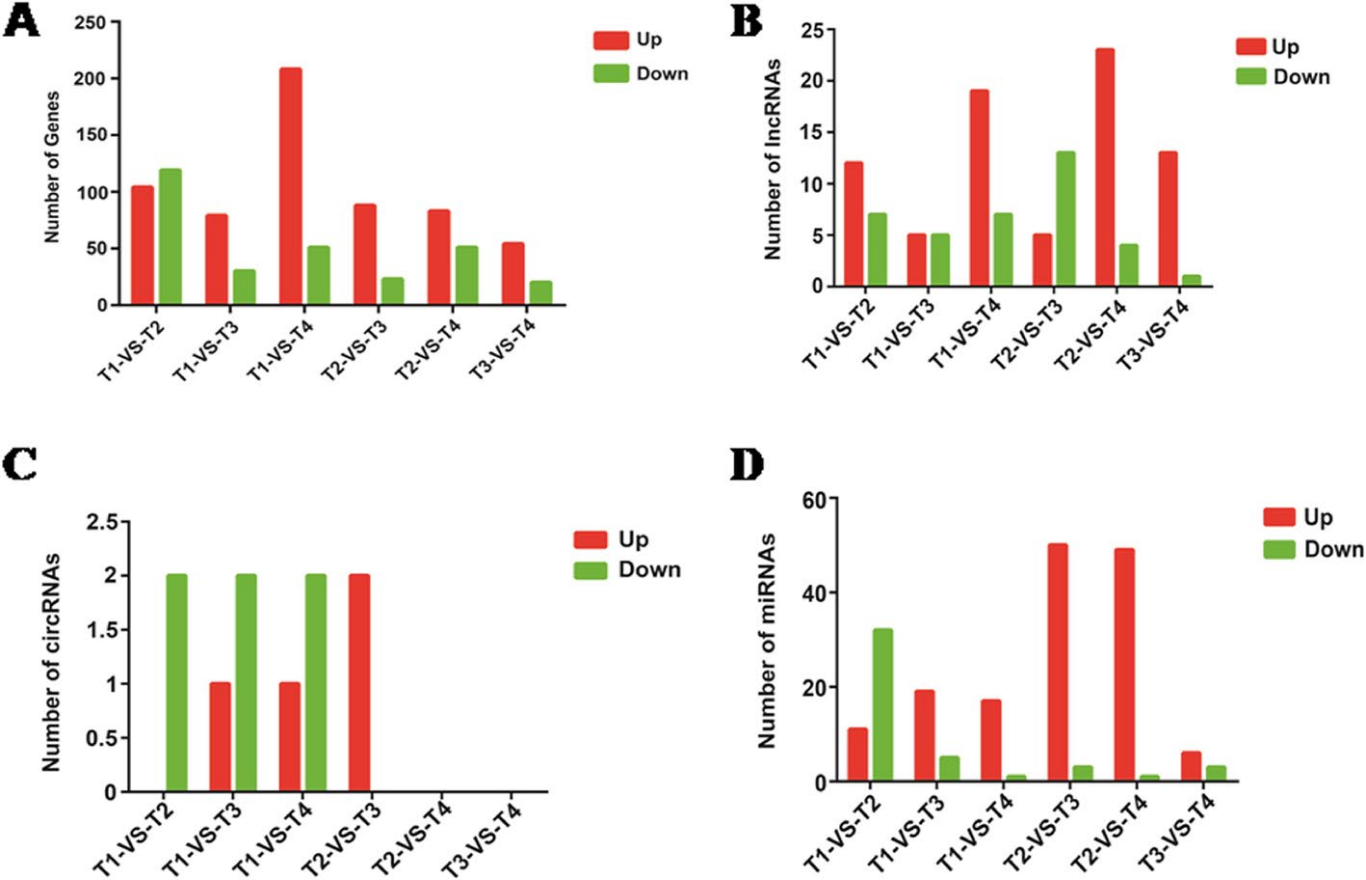
The differentially expressed mRNAs, lncRNAs, circRNAs, and miRNAs of microsporidia between 5-day embryos, 1-day larvae, 5-day larvae, and 10-day larvae challenged by *N. bombycis* congenital infection. **A**: The differentially expressed mRNAs of microsporidia at four different developmental stages; **B**: The differentially expressed lncRNAs of microsporidia at four different developmental stages; **C**: The differentially expressed circRNAs of microsporidia at four different developmental stages; **D**: The differentially expressed miRNAs of microsporidia at four different developmental stages. T1: 5-day embryos with *N. bombycis* congenital infection; T2: 1-day larvae with *N. bombycis* congenital infection; T3: 1-day larvae with *N. bombycis* congenital infection; T4: 10-day larvae with *N. bombycis* congenital infection

To identify expression pattern of *N. bombycis* coding and non-coding RNAs in four different stages of congenitally infected silkworm, differentially expressed (DE) RNA between four stages were characterized based on the combined criteria of absolute fold change ≥ 2 and a* P* value < 0.05. As shown in Fig. 2, different subsets of RNAs were up or down- regulated in *N. bombycis* at different stages. Many differentially expressed mRNAs, lncRNAs and miRNAs were found, however rare differentially expressed circRNAs were shown at the different developmental stages.

**Fig. 3:**
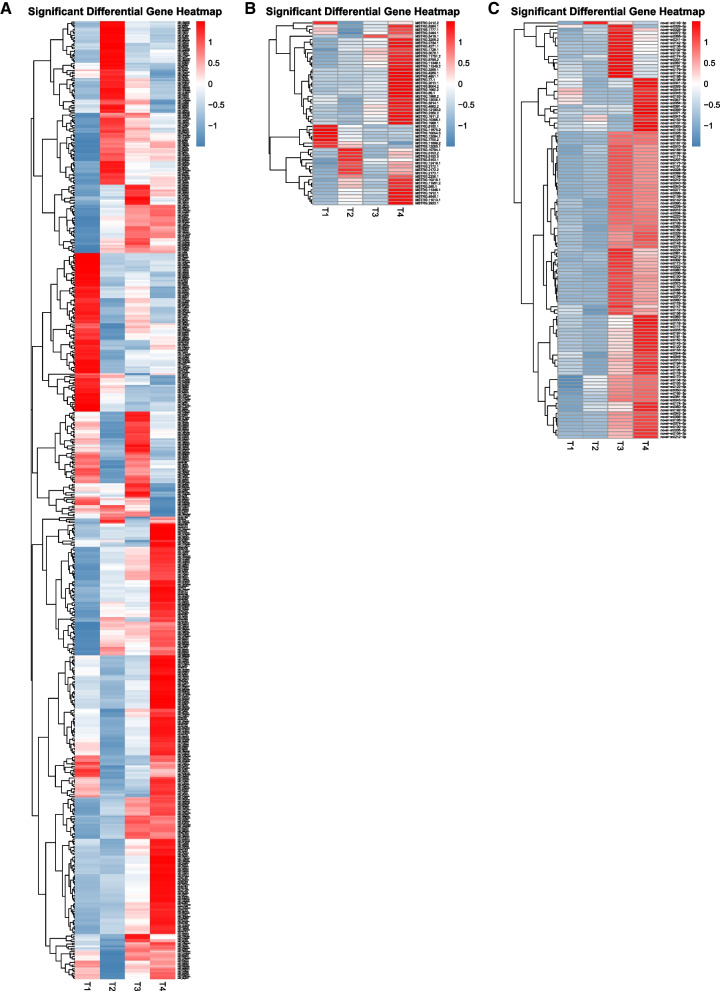
Significant differential microsporidia mRNA, lncRNA and miRNA heatmap analysis between 5-day embryos, 1-day larvae, 5-day larvae, and 10-day larvae challenged by *N. bombycis* congenital infection. **A**: Significant differential microsporidia mRNA heatmap analysis was showed at four different stages, and the DE genes were fallen into 5 clusters with the highest expressed gene clusters; **B**: Significant differential microsporidia lncRNA heatmap analysis was showed at four different stages; **C**: Significant differential microsporidia miRNA heatmap analysis was showed at four different stages, and the DE miRNAs were fallen into 4 clusters. Genes which are down-regulated are depicted in blue, with darker colors representative of greater down-regulation. Up-regulated genes are depicted in red, with darker shading corresponding to a greater degree of expression. T1: 5-day embryos with *N. bombycis* congenital infection; T2: 1-day larvae with *N. bombycis* congenital infection; T3: 1-day larvae with *N. bombycis* congenital infection; T4: 10-day larvae with *N. bombycis* congenital infection

To further visualize the expression of DE mRNAs, we generated heat maps with down-regulated RNAs showing in blue and up-regulated RNAs showing in red. As shown in Fig. 3A, there were 574 DE genes that were fallen into five clusters according to the different gene expression patterns (Table S3). KEGG pathways were enriched by up-and down-regulated DE genes. The expression of genes in cluster I rapidly increased from the fifth day of embryos to the first day of larvae, and gradually decreased. Genes were associated with ribosome biogenesis in eukaryotes, protein processing in endoplasmic reticulum, aminoacyl-tRNA biosynthesis, RNA polymerase, and pyrimidine metabolism (Fig. S3A). Genes in cluster II continued to increase from the fifth day of embryos to the fifth day of larvae, and remained temporally stable at the tenth day of larvae. These genes were mainly involved in purine metabolism, RNA polymerase, pyrimidine metabolism, non-homologous end-joining, and SNARE interactions in vesicular transport (Fig. S3B). The expression of genes in cluster III was highest at the fifth day of embryos, and gradually decreased in larvae. KEGG pathways analysis showed that genes were mainly involved in starch and sucrose metabolism, biosynthesis of antibiotics, metabolic pathways, carbon metabolism, and microbial metabolism in diverse environments (Fig. S3C). The expression of genes in cluster IV decreased from the fifth day of embryos to the first day of larvae, and occurred a same trend from fifth day of larvae to tenth day of larvae. As shown in Fig. S3D, these genes were mainly involved in inositol phosphate metabolism, fructose and mannose metabolism, glycolysis/gluconeogenesis, biosynthesis of amino acids, and protein export. Genes in cluster V continued to increase their highest expression at the tenth day of larvae, and these genes were associated with biosynthesis of antibiotics, metabolic pathways, MAPK signaling pathway-yeast, sphingolipid metabolism, and amino sugar and nucleotide sugar metabolism (Fig. S3E).

Similarly, we generated a heat map based on 55 DE lncRNAs of *N. bombycis* (Fig. 3B, Table S3). We found that most lncRNAs were highly expressed in 10-day larvae, and those lncRNAs can be classified into four clusters. The expression of lncRNAs in cluster I was highly expression at the fifth day of embryos and the tenth day of larvae, and remained low expression at the other stages. LncRNAs in cluster II continued to increase their highest expression at the tenth day of larvae. Sever lncRNAs (MSTRG10794.1, MSTRG5153.1, MSTRG5153.2, MSTRG5153.3, MSTRG12418.1, MSTRG2172.1, MSTRG2172.2, and MSTRG2172.3) in cluster III was highest at the fifth day of embryos, and gradually decreased in larvae. The expression of lncRNAs in cluster IV increased from the fifth day of embryos to the first day of larvae, and occurred a same trend from fifth day of larvae to tenth day of larvae. Furthermore, lncRNA-mRNA association analysis revealed target genes of lncRNAs in antisense, cis, and trans-regulation (Table S4).

The heat map showed that the expression levels of most DE miRNAs in larvae were higher than those in embryos, and 125 DE miRNAs (Table S3) target genes were divided into 4 clusters (Fig. 3C, Table S5). The expression of miRNAs in cluster I increased from the fifth day of embryos to the fifth day of larvae, and showed downregulation at the tenth day of larvae. These miRNAs were mainly involved in mRNA surveillance pathway, cell cycle-yeast, aminoacyl-tRNA biosynthesis, ether lipid metabolism, and glycerophospholipid metabolism (Fig. S4A). The expression of miRNAs in cluster II was highest at the tenth day of larvae, and showed low expression at the other stages. In second cluster miRNAs targeted genes were associated with protein processing in endoplasmic reticulum, glycerophospholipid metabolism, RNA polymerase, glycine, serine and threonine metabolism, and methane metabolism (Fig. S4B). The expression of miRNAs in cluster III continued to increase from the fifth day of embryos to the fifth day of larvae, and remained temporally stable at the tenth day of larvae. As shown in Fig. S4C, miRNAs targeted genes in cluster III were associated with aminoacyl-tRNA biosynthesis, protein processing in endoplasmic reticulum, starch and sucrose metabolism, proteasome, and glycolysis /gluconeogenesis. The expression of miRNAs in cluster IV continued to increase their highest expression at the tenth day of larvae. As shown in Fig. S4D, miRNAs targeted genes in cluster IV were involved into protein processing in endoplasmic reticulum, cell cycle-yeast, aminoacyl-tRNA biosynthesis, glycolysis/gluconeogenesis, and arachidonic acid metabolism.

### Intraspecies ceRNA networks of *N. bombycis* lncRNAs and circRNAs in congenitally infected silkworm embryos and larvae

**Fig. 4:**
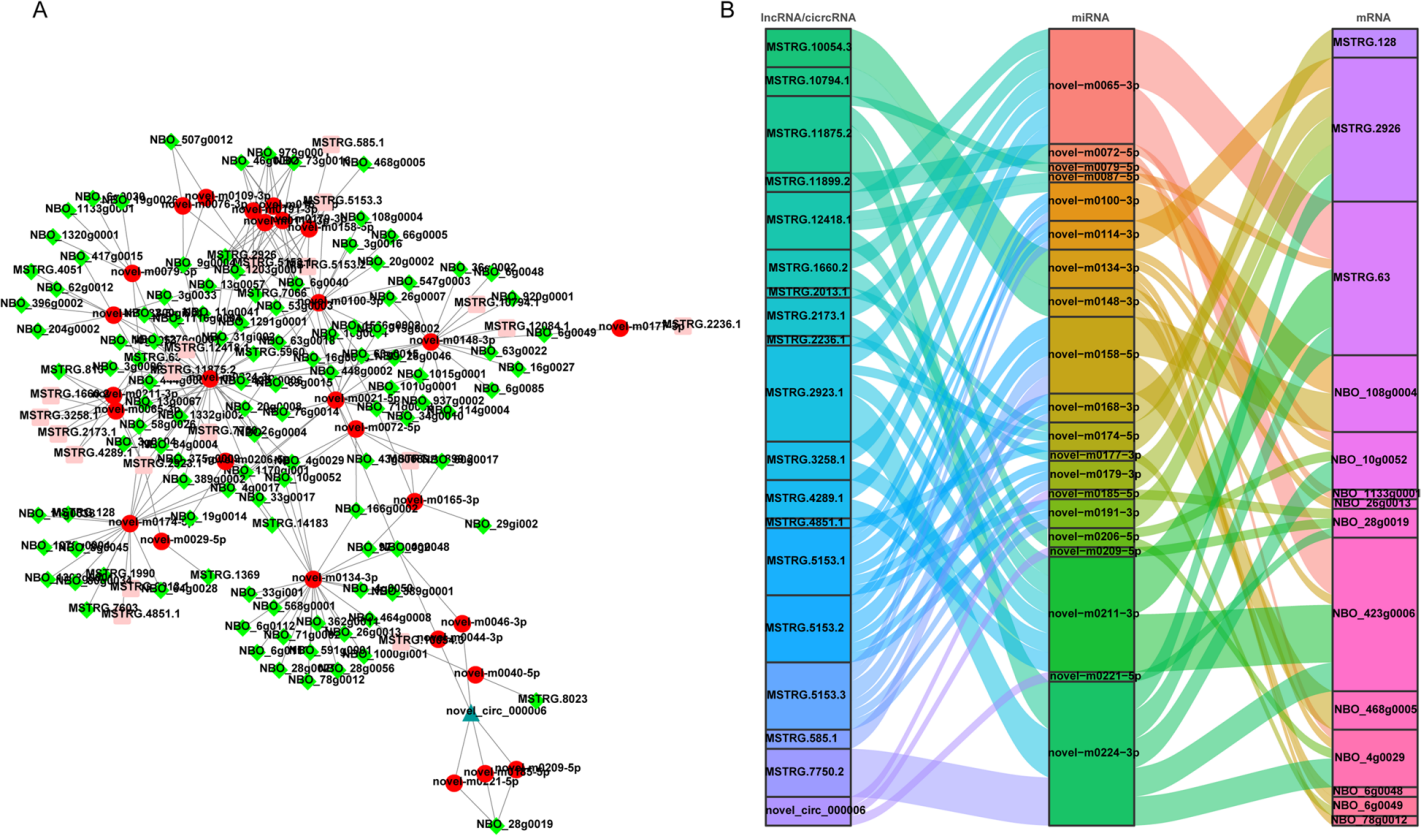
Intraspecies ceRNA networks of *N. bombycis* in congenitally infected silkworm embryos and larvae. **A**. CeRNA network for *N. bombycis* differentially expressed mRNAs, lncRNAs, circRNAs and miRNAs 5-day embryos, 1-day larvae, 5-day larvae, and 10-day larvae challenged by *N. bombycis* congenital infection. Green quadrangle nodes, mRNAs; Red circle nodes, miRNAs; Blue triangle nodes, circRNAs; Light red quadrangle nodes, lncRNAs. **B**. Sankey diagram for the lncRNA/circRNA-miRNA-mRNA ceRNA network of *N. bombycis* in congenitally infected silkworm embryos and larvae. Each rectangle represents a gene, and the connection degree of each gene is visualized based on the size of the rectangle

To discover the function of lncRNAs and circRNAs of *N. bombycis* ceRNA networks for DE lncRNAs and circRNAs were constructed. We first apply the principle of base complementary pairing to match the miRNAs with mRNAs, lncRNAs and circRNAs, resulting in values of the correlation between miRNA-mRNA, miRNA-lncRNA or miRNA-circRNA node. Then, only the negatively correlated pairs of miRNA-mRNA, miRNA-lncRNA or miRNA-circRNA were retained, and the final ceRNA networks included lncRNA-miRNA-mRNA and circRNA-miRNA-mRNA networks were constructed (Table S6, 7), and this ceRNA networks include 18 lncRNAs, one circRNA, 28 miRNA, and 113 mRNA were visualized by Cytoscape software (Fig. 4A). Meanwhile, Sankey diagrams showed the ceRNA networks involving 14 key parasites genes such as *PTP3*, ricin-B-lectin, *SWP4*, *HSP90*, 18 lncRNAs, one circRNA, and 20 miRNAs (Fig. 4B). Furthermore, we used topology method to calculate the connection degree of each gene to illustrate its importance in the ceRNA network (Table S8). Three miRNAs (novel-m0224-3p, novel-m0211-3p and novel-m0065-3p), three lncRNAs (MSTRG.5153.1, MSTRG.5153.2 and MSTRG.5153.3), and one circRNA (novel-circ-000006) had high connection degrees.

### Transcriptome-wide identification and characteristics of *B. mori* mRNAs, lncRNAs, circRNAs and miRNAs in the congenital *N. bombycis*-infected silkworm embryos and larvae

**Fig. 5:**
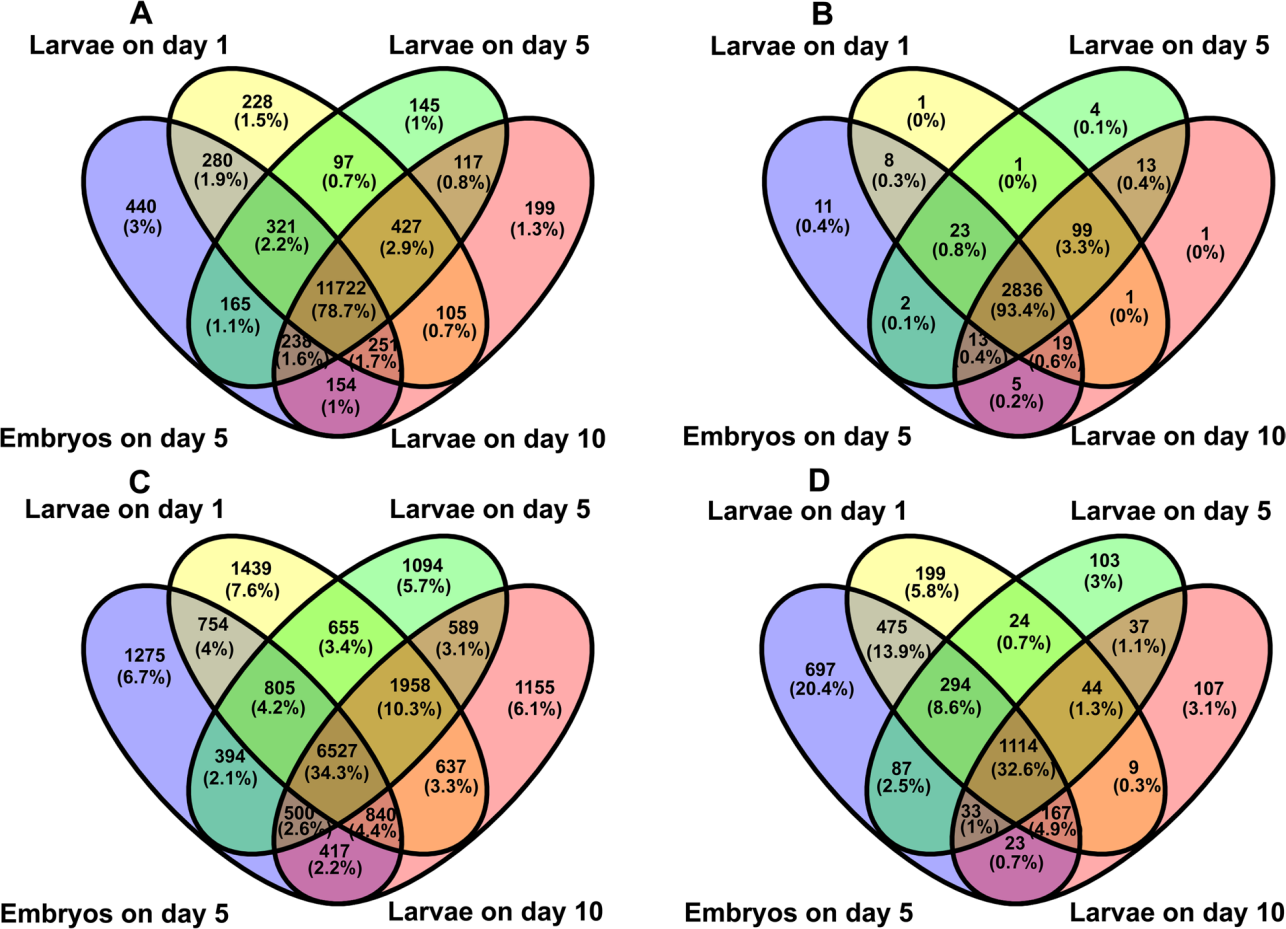
Venn diagram showing the number of exclusive and shared expressed mRNAs, lncRNAs, circRNAs and miRNAs of silkworm both Uninfected and infected silkworm embryos and larvae. **A**: The Venn diagram of silkworm mRNA expression; **B**: The Venn diagram of silkworm lncRNA expression; **C**: The Venn diagram of silkworm circRNA expression; **D**: The Venn diagram of silkworm miRNA expression

For *B. mori* mRNA, 13,592 genes were aligned to reference genes and 937 genes were defined as novel genes (Table S9). Furthermore, the total numbers of the predicted *B. mori* genes at different stages were comparable with 13,571, 13,431, 13,232, and 13,213 genes in 5-day embryos (CK1 and T1), 1-day (CK2 and T2), 5-day (CK3 and T3), 10-day (CK4 and T4) larvae, respectively (Table 4), and common 11,722 genes were detected in four stages, accounting for 78.7% of all predicted *B. mori* genes and only 440, 228, 145, and 199 genes were stage-specifically expressed mRNA genes based on Venn diagram analysis (Fig. 5A).

**Table 4:** The coding and non-coding RNAs identification of silkworm in *N. bombycis* congenitally infected and uninfected silkworm embryos and larvae

Stage RNA types	Embryos on day 5		Larvae on day 1		Larvae on day 5		Larvae on day 10		Sum
	Uninfected	Infected	Uninfected	Infected	Uninfected	Infected	Uninfected	Infected	
mRNA	12835	12836	12696	12704	12500	12437	12405	12348	14889
lncRNA	2868	2862	2955	2943	2949	2942	2918	2936	3037
circRNA	9127	8661	10696	10709	9114	10065	8788	10530	19039
miRNA	2768	2458	2236	1171	1600	835	1429	883	3403

Regarding lncRNA, a total of 3,038 *B. mori* lncRNAs were identified in the uninfected and infected embryos and larvae, and there were 2,917, 2,988, 2,991, and 2,987 lncRNAs that were detected in 5-day embryos (CK1 and T1), 1-day (CK2 and T2), 5-day (CK3 and T3), 10-day (CK4 and T4) larvae, respectively (Table 4). Additionally, there were 2,836 lncRNAs that were identified in all four stages, and only 11, 1, 4, and 1 lncRNAs were stage-specifically expressed lncRNA genes (Fig. 5B).

With regard to the *B. mori* circRNAs, a total of 19,039 lncRNAs were identified in the uninfected and infected embryos and larvae, and there were 11,512, 13,615, 12,522, and 12,623 circRNAs that were identified in CK1 and T1, CK2 and T2, CK3 and T3, and CK4 and T4 stages, respectively (Table 4) with 6,527 circRNAs being identified in all four stages and 1,275, 1,439, 1,094, and 1,155 circRNAs being identified in CK1 and T1, CK2 and T2, CK3 and T3, and CK4 and T4 stages, respectively (Fig. 5C).

Similarly, as shown in Table 4, a total of 3,403 *B. mori* miRNAs were identified in all four above mentioned stages. There were 2,890, 2,326, 1,736, and 1,534 miRNAs that were detected in CK1 and T1, CK2 and T2, CK3 and T3, and CK4 and T4 stages, respectively (Table 4). Additionally, there were 1,114 miRNAs were shared in four stages, accounting for 32.6% of all predicted *B. mori* miRNAs, and 697, 199, 103, and 107 miRNAs that were stage-specifically expressed miRNA genes based on Venn diagram analysis (Fig. 5D).

### Analysis of differentially expressed mRNAs, lncRNAs, circRNAs and miRNAs of *B. mori* in the congenital *N. bombycis*-infected silkworm embryos and larvae

**Fig. 6:**
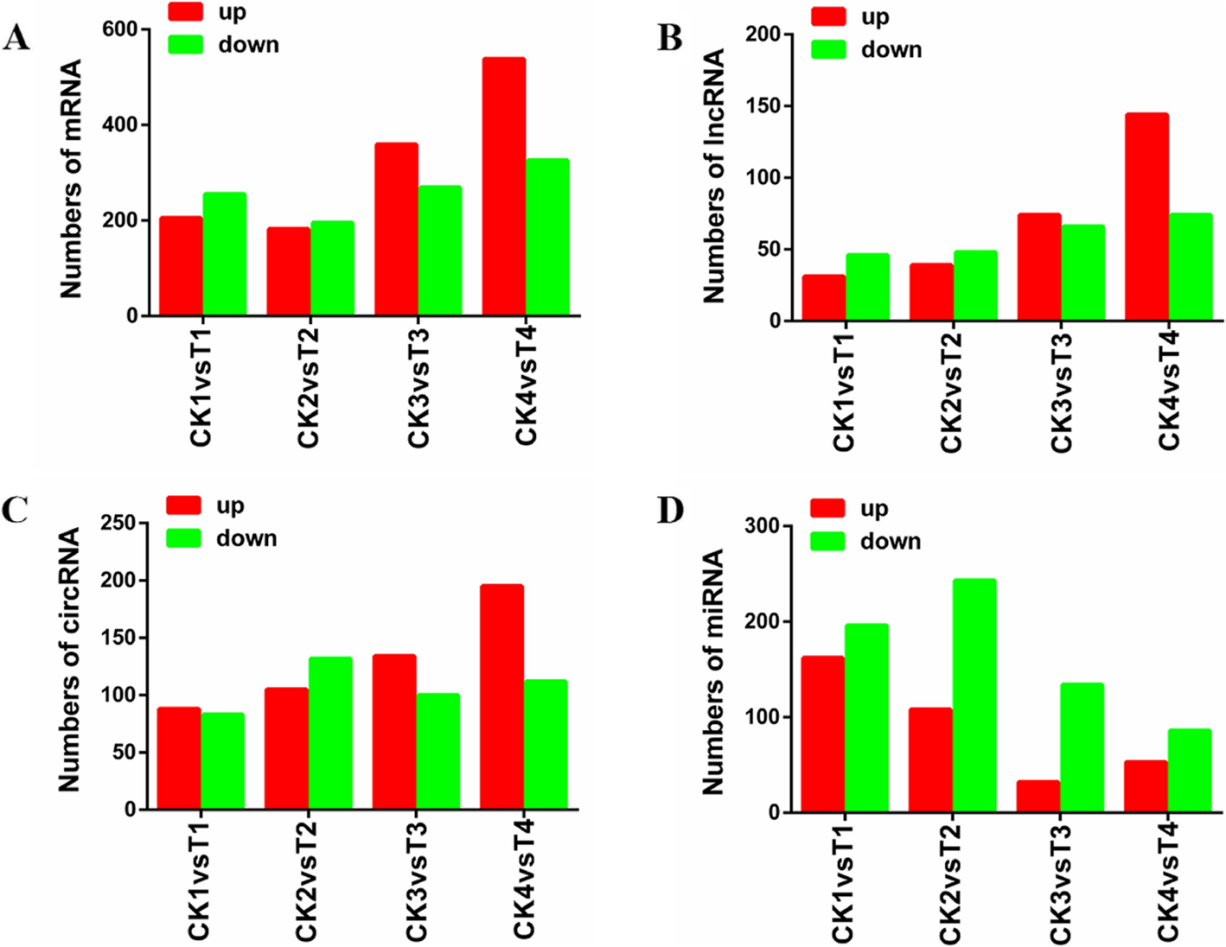
The differentially expressed mRNAs, lncRNAs, circRNAs, and miRNAs of *B. mori* by comparing uninfected and congenitally infected embryos and larvae. **A**: The differentially expressed mRNAs of *B. mori* by comparing uninfected and congenitally infected specimens; **B**: The differentially expressed lncRNAs of *B. mori* by comparing uninfected and congenitally infected specimens; **C**: The differentially expressed circRNAs of *B. mori* by comparing uninfected and congenitally infected specimens; **D**: The differentially expressed miRNAs of *B. mori* by comparing uninfected and congenitally infected specimens. CK1-4: Uninfected 5-day embryos (CK1), 1-day larvae (CK2), 5-day larvae (CK3), and10-day larvae (CK4); T1-4: Infected 5-day embryos (T1), 1-day larvae (T2), 5-day larvae (T3), and 10-day larvae (T4)

To understand the role of various forms of host RNAs in *N. bombycis* congenital infection, we next analyzed the differentially expressed mRNAs, lncRNAs, circRNAs, and miRNAs by comparing uninfected and congenitally infected specimen. We found that there are a large number of differentially expressed mRNAs, lncRNAs, circRNAs and miRNAs in the embryos and larvae congenitally infected by *N. bombycis*. As shown in Fig. 6A, 460 (205 up-regulated and 255 down-regulated), 377 (182 up-regulated and 195 down-regulated), 628 (359 up-regulated and 269 down-regulated), and 864 mRNAs (538 up-regulated and 326 down-regulated) were DE genes during the congenital *N. bombycis* infection in the embryos on day 5, larvae on day 1, 5, and 10, respectively. For lncRNAs, there were 77 (31 up-regulated and 46 down-regulated), 87 (39 up-regulated and 48 down-regulated), 140 (74 up-regulated and 66 down-regulated), and 218 DE lncRNAs (144 up-regulated and 74 down-regulated) after the congenital *N. bombycis* infection in the embryos on day 5, larvae on 1, 5 and 10 days, respectively (Fig. 6B). Regarding circRNAs, there were 171 (88 up-regulated and 83 down-regulated), 237 (105 up-regulated and 132 down-regulated), 234 (134 up-regulated and 100 down-regulated) and 307 (195 up-regulated and 112 down-regulated) DE circRNAs in the silkworm 5-day embryos, 1, 5 and 10-day larvae on of the congenital infected group versus the control group, respectively (Fig. 6C). As shown in Fig. 6D, in the silkworm embryos on 5 days, 358 DE miRNAs (162 up-regulated and 196 down-regulated) were observed in the silkworm embryos following the congenital *N. bombycis* infection; there were 351 DE miRNAs (108 up-regulated and 243 down-regulated), 166 DE miRNAs (32 up-regulated and 134 down-regulated), 139 DE miRNAs (53 up-regulated and 86 down-regulated) in the larvae on 1, 5 and 10 days of the infected group versus the control group, respectively.

**Fig. 7:**
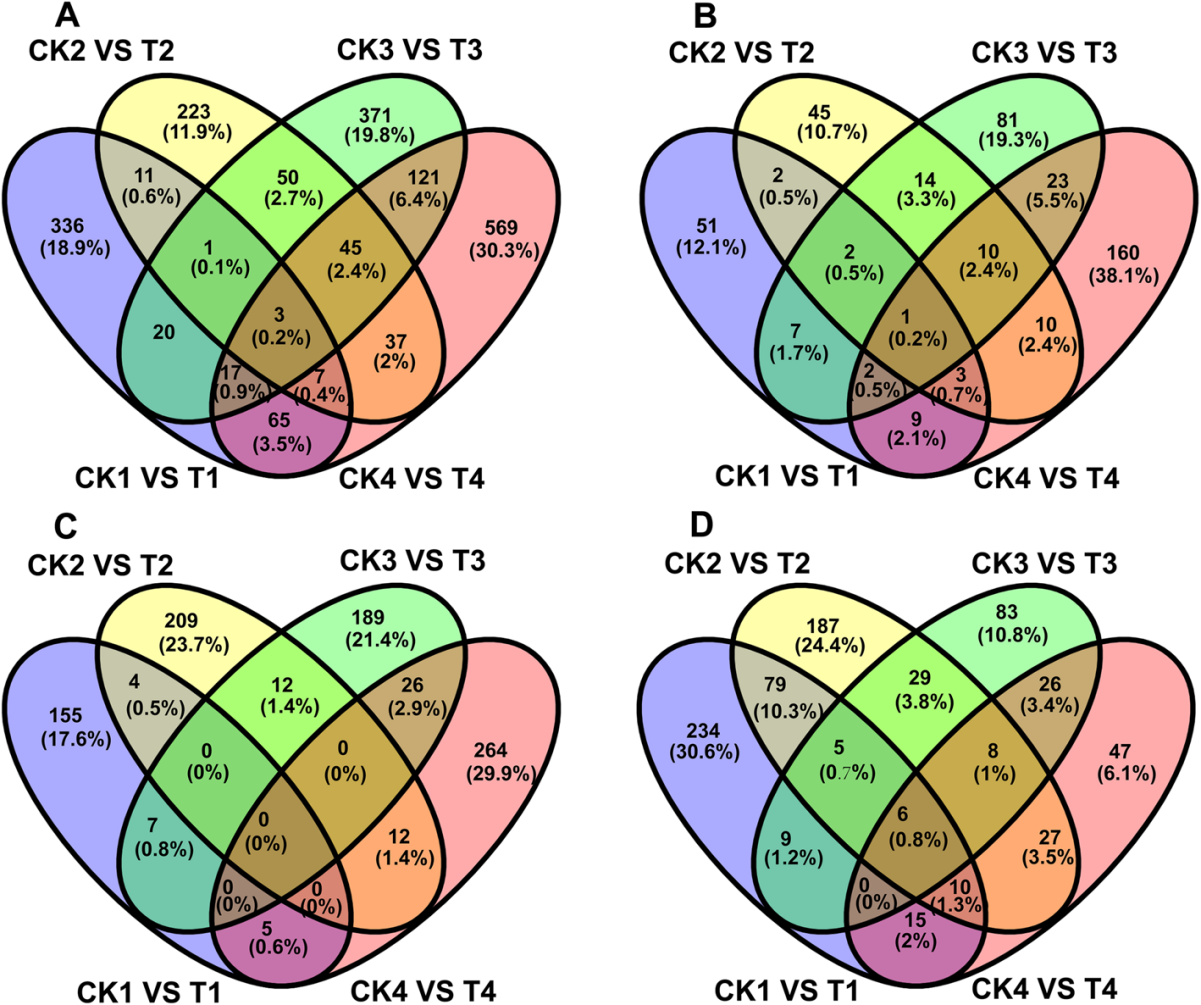
Venn diagrams showing the number of exclusive and shared differentially expressed mRNAs, lncRNAs, circRNAs and miRNAs during the *N. bombycis* congenital infection in 5-day embryos, 1-day larvae, 5-day larvae, and 10-day larvae, respectively. **A**: Exclusive and shared differentially expressed mRNAs in four stages mentioned; **B**: Exclusive and shared differentially expressed lncRNAs in four stages mentioned; **C**: Exclusive and shared differentially expressed circRNAs in four stages mentioned; **D**: Exclusive and shared differentially expressed miRNAs in four stages mentioned. CK1-4: Uninfected 5-day embryos (CK1), 1-day larvae (CK2), 5-day larvae (CK3), and10-day larvae (CK4); T1-4: Infected 5-day embryos (T1), 1-day larvae (T2), 5-day larvae (T3), and 10-day larvae (T4)

Moreover, we analyzed exclusive and shared differentially expressed mRNAs, lncRNAs, circRNAs and miRNAs over all the stages and stage-specific differentially expressed mRNAs, lncRNAs, circRNAs and miRNAs (Fig. 7A-D). Figure 7 represented that there were three mRNAs (protein lethal (2) essential for life-like, nuclear receptor GRF and transferrin precursor), one lncRNA (MSTRG.23380.1), no circRNA and six miRNAs (bmo-miR-2780a-5p, bmo-miR-2780b, bmo-miR-3001, bmo-miR-306b, miR-8517-x and novel-m0636-3p) that were common differentially expressed genes over all the stages.

### Functional analysis of congenital *N. bombycis* infection-induced DE mRNAs, lncRNAs, circRNAs and miRNA in the silkworm embryos and larvae

**Fig. 8:**
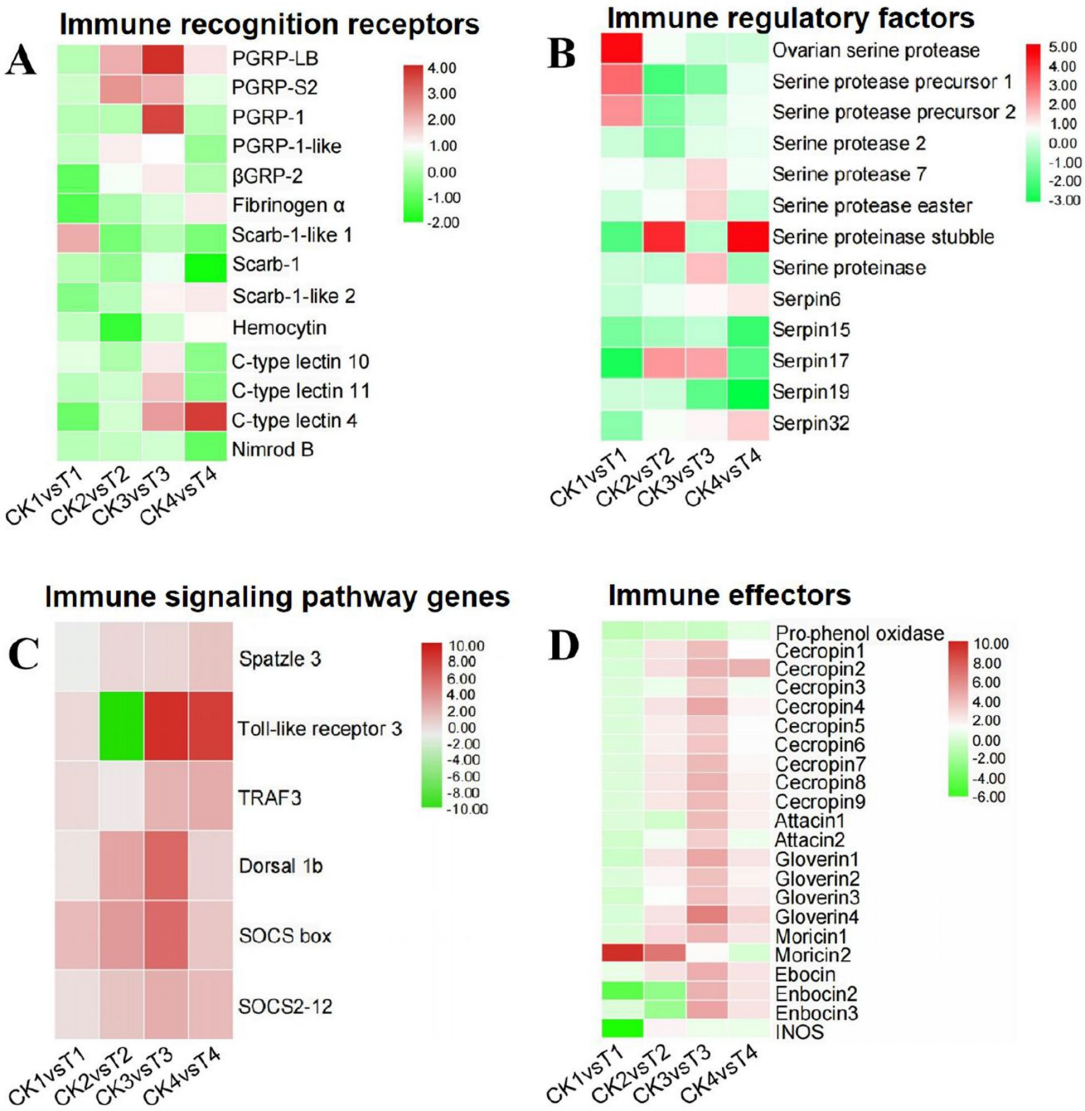
The heatmap of *B. mori* differentially expressed related immune genes during the *N. bombycis* congenital infection in 5-day embryos, 1-day larvae, 5-day larvae, and 10-day larvae, respectively. **A**: Heatmap of differentially expressed immune recognition receptors; **B**: Heatmap of differentially expressed immune regulatory factors; **C**: Heatmap of differentially expressed immune signaling pathway genes; **D**: Heatmap of differentially expressed immune effectors. Genes which are down-regulated are depicted in green, with greener colors representative of greater down-regulation. Up-regulated genes are depicted in red, with darker shading corresponding to more expression. CK1-4: Uninfected 5-day embryos (CK1), 1-day larvae (CK2), 5-day larvae (CK3), and10-day larvae (CK4); T1-4: Infected 5-day embryos (T1), 1-day larvae (T2), 5-day larvae (T3), and 10-day larvae (T4)

To reveal the potential functions of DE mRNAs, we analyzed KEGG pathways of those up-regulated and down-regulated mRNAs (Fig. S5A-D). KEGG enrichment analysis of DE mRNAs showed that those genes involved in cell cycle, oocyte meiosis, Toll and Imd signaling pathway, longevity regulating pathway-multiple species, and antigen processing and presentation. In order to investigate the immune status of the host, *B. mori* immune function related DE genes were constructed to form four heat maps (Fig. 8A-D). We found that immune recognition receptors (beta-1,3-glucan recognition protein 2 precursor (*βGRP2*), fibrinogen alpha chain, and c-type lectin 4), immune regulatory genes (ovarian serine protease and *serpin-15*), immune signaling pathway gene (*Spz3*) and immunoeffector genes (pro-phenol oxidase and *iNOS*) were down-regulated in 5-day embryos exposed to the congenital *N. bombycis* challenge, while those genes and other immune-related genes such as peptidoglycan recognition protein, scavenger receptor, serine protease 7, dorsal and most antimicrobial peptide genes were up-regulated in larvae, especially in 5-day larvae exposed to the congenital *N. bombycis* challenge. The above results suggest that immune system involved in Toll and Imd signaling pathway and hemolymph melanization is in immunosuppression in the embryos exposed to the congenital *N. bombycis* infection, and host immune system involved in Toll and Imd signaling pathway, hemolymph melanization, and phagocytosis is activated by *N. bombycis* infection in the larvae, especially in the early and middle larvae.

**Fig. 9:**
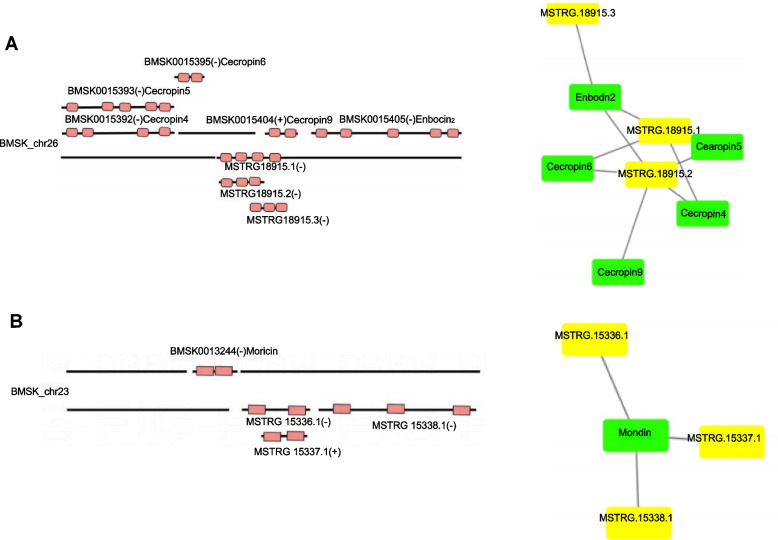
LncRNAs were predicted to regulate their neighboring related immune genes on the same alleles *in cis* in silkworm embryos and larvae during the *N. bombycis* congenital infection. The lncRNAs which have intersection of promoter or other cis-elements may regulate gene expression in transcriptional or post-transcriptional level. lncRNAs in less than 100 kb up/downstream of a gene were likely to be cis-regulators. **A**: Schematic diagram of three lncRNAs(MSTRG18915.1, MSTRG18915.2, and MSTRG18915.3) and its nearly genes(cecropin and enbocin); **B**: Schematic diagram of three lncRNAs(MSTRG15336.1, MSTRG15337.2, and MSTRG15338.1) and moricin. Green rectangle nodes, mRNA; Yellow rectangle nodes, lncRNA

One of the functions of lncRNAs is cis-regulation of their neighboring genes on the same allele. LncRNAs in less than 100 Kb up/downstream of a gene were likely to be cis-regulators. We identified lncRNA-mRNA pairs including 99 lncRNAs and 105 mRNAs (Table S10). The cis-target genes of lncRNAs were then subjected to enrichment analysis of KEGG pathways. KEGG enrichment analysis showed that systemic lupus erythematosus, Toll and Imd signaling pathway, drug metabolism-other enzymes, longevity regulating pathway-multiple species, caffeine metabolism, and alcoholism were enriched under the congenital *N. bombycis* stress (Fig. S6). We specifically focused on lncRNAs regulating immune-related gene expression in *cis* (Fig. 9). A string of tandem genes encoded cecropin and enbocin, which were lncRNAs (MSTRG18915.1, MSTRG18915.2 and MSTRG18915.3) nearly genes (Fig. 9A). In addition, one gene (moricin) was predicted to be three lncRNAs (MSTRG15336.1, MSTRG15337.1 and MSTRG15338.1) nearly genes that were likely to be regulated by lncRNAs *in cis* (Fig. 9B)*.*

We performed the functional enrichment analysis of DE circRNA source genes to study the main functions of these genes (Fig. S7A-D). KEGG enrichment analysis of DE circRNA source genes showed that those genes involved in relaxin signaling pathway, PI3K-Akt signaling pathway, focal adhesion, secretion and action, microRNAs and thyroid hormone synthesis.

MiRNAs recognize and bind to partially complementary sites in the 3′ untranslated regions of target genes and regulate the expression of the target transcripts. To study the functions of the targeted genes, we carried out the functional enrichment analysis of DE miRNA target genes (Fig. S8A-D). KEGG enrichment analysis of DE miRNA target genes showed many important biological processes including protein processing in endoplasmic reticulum, apoptosis-multiple species, endocytosis, VEGF signaling pathway and cell cycle were enriched. In addition, the basic metabolisms of life such as carbohydrate, lipid and amino acid metabolisms were also targets of miRNAs.

### Intraspecies ceRNA networks of DE lncRNAs and circRNAs in *B. mori* in the congenitally infected by *N. bombycis*

It was known that lncRNAs and circRNAs can be considered as two types of ceRNAs, which play significant role in response to various pathogens infection [23, 46]. To access the function of lncRNAs and circRNAs in the congenital *N. bombycis* infection in the silkworm embryos and larvae, dynamical ceRNA networks were analyzed for four different stages during the congenital *N. bombycis* infection. In the 5-day silkworm embryos, lncRNA and circRNA ceRNA networks were constructed following the congenital *N. bombycis* infection (Table S11, 12). The lncRNA ceRNA network contained 59 lncRNAs and 103 miRNAs, and the circRNA ceRNA network consisted of 64 circRNAs and 101 miRNAs. Similarly, in the silkworm 1, 5 and 10-day larvae, the lncRNA/circRNA ceRNA networks were constructed following the congenital *N. bombycis* infection, respectively (Table S13-18). According to previous reports, ceRNA network has two characteristically distinct types: one is common to all stages, which is defined as the common ceRNA networks, and other is unique for each stage, which is defined the unique ceRNA networks [47]. We found that lncRNA/circRNA ceRNA networks are almost the unique ceRNA networks.

**Fig. 10:**
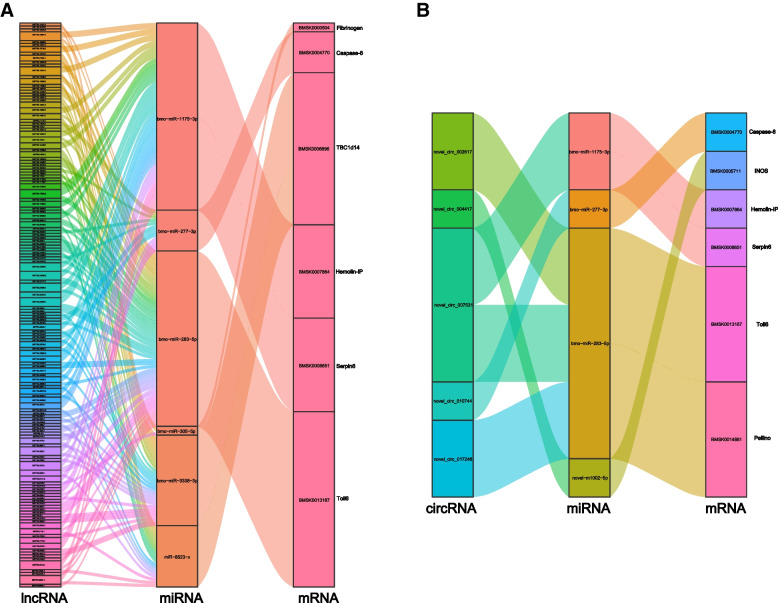
Sankey diagram for the lncRNA/circRNA-miRNA-mRNA ceRNA network in silkworm embryos and larvae during the *N. bombycis* congenitally infection. **A**: LncRNA-miRNA-mRNA ceRNA network; **B**: CircRNA-miRNA-mRNA ceRNA network. Each rectangle represents a gene, and the connection degree of each gene is visualized based on the size of the rectangle

The innate immune response of silkworms plays crucial roles in the resistance to *N. bombycis* infection [48]; therefore, we next focused on the differential expression of immune-related genes in the silkworm embryos and larvae during the congenital *N. bombycis* infection. There were 8 immune-related DE mRNAs including *Toll-6*, fibrinogen alpha chain, *Serpin-6*, *Caspase-8*, *Caspase-1*, *iNOS*, hemolin-interacting protein (*Hemolin-IP*), *Pellino*, and TBC1 domain family member 14 (*TBC1d14*). Based on 8 DE mRNAs, we further constructed and illustrated the ceRNA networks. The lncRNA-miRNA-mRNA pathways were constructed, including 140 lncRNAs and 6 miRNAs (Fig. 10A). And we used topology method to calculate the connection degree of each gene to illustrate its importance in the lncRNA ceRNA network (Table S19). Similarly, the circRNA-miRNA-mRNA pathways were constructed, including 5 circRNAs and 4 miRNAs (Fig. 10B), of which, lncRNA ceRNA networks shares the 4 mRNAs and 3 miRNAs with circRNA ceRNA networks. Furthermore, we used topology method to calculate the connection degree of each gene to illustrate its importance in the circRNA ceRNA network (Table S20).

### Intraspecies and cross-species miRNAs targets analysis

**Fig. 11:**
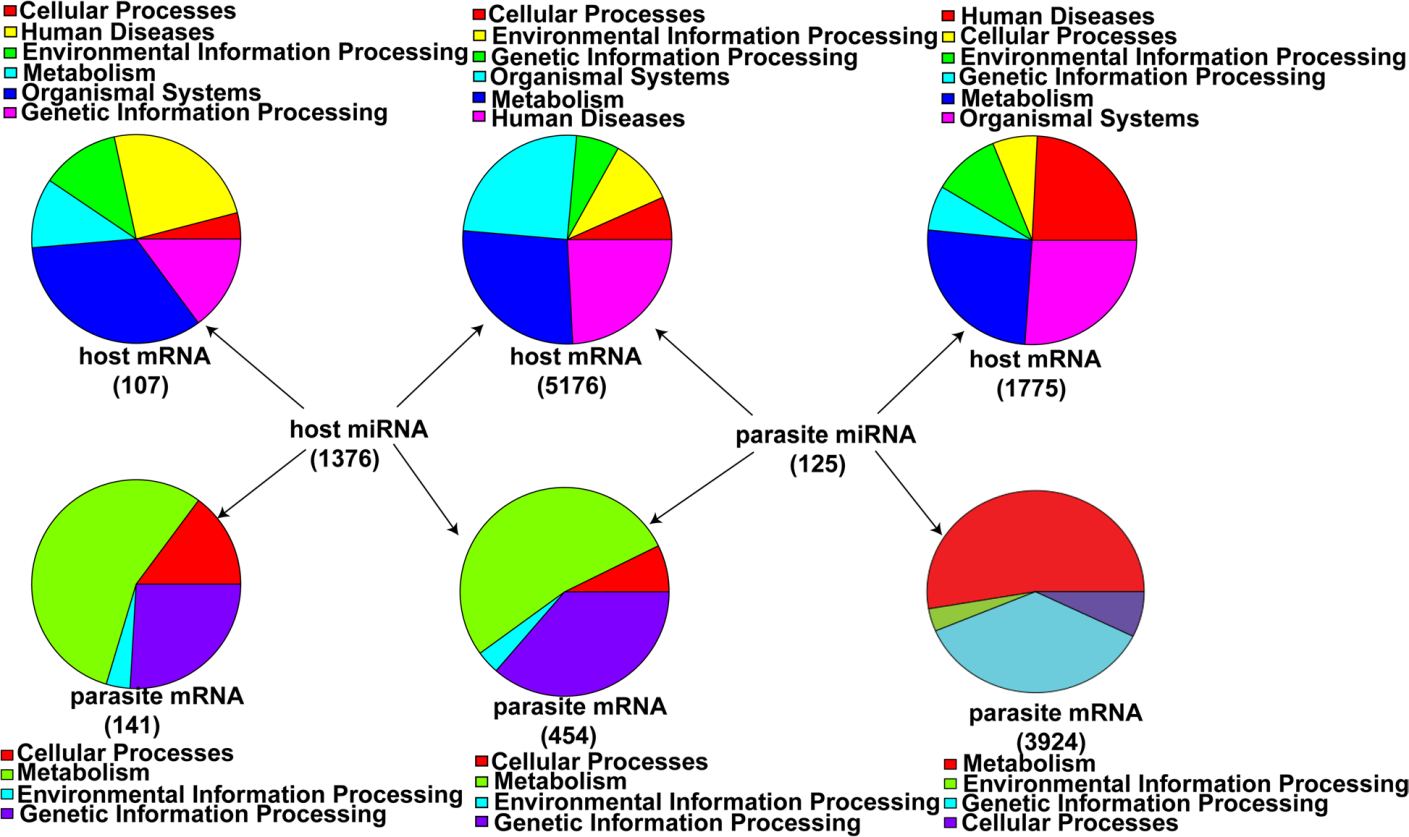
Intraspecies and cross-species miRNA targets analysis. 6,591 host genes and 4,378 parasite genes were potentially targeted by 125 parasite miRNAs. 1,376 host miRNAs could target 5,283 host genes and 595 parasite genes. 5,176 host genes and 454 parasite genes could be targeted by the miRNAs of both host and parasite. The functional category of those genes in each cluster and corresponding color were shown according to the KEGG pathway database

It is known that miRNAs act not only in the cells where they are biologically formed but they also function in cells from different species [49]. We screened 125 parasite and 1,376 silkworms differentially expressed miRNAs, and their intraspecies and cross-species miRNAs targets were predicted in *N. bombycis* and silkworm interaction (Fig. 11). For 125 parasite miRNAs, intraspecies and cross-species miRNA targets analysis showed that 4,378 parasite genes and 6,951 host genes were predicted to be those parasite miRNAs targets, respectively. For 1,376 host miRNAs, 5,283 silkworm genes and 595 parasite genes were predicted to be those host miRNAs targets, respectively. Moreover, 5,176 host genes and 454 parasite genes may be regulated not only by pathogen miRNAs, but also by host miRNAs. KEGG pathways analysis showed that 5,176 host genes were mainly involved in cellular processes, environmental information processing, genetic information processing, organismal systems, metabolism, and human disease, and 454 parasite genes were involved in cellular processes, metabolism, environmental information processing and genetic information processing. In this analysis, miRNA-mediated cross-species regulation is worth further investigation. For example, two *N. bombycis* miRNAs (novel-m0083-3p and novel-m0044-3p) are highly expressed, and their targets are silkworm Pelle and Spz3, two key genes in Toll signaling pathway, which are downregulated in embryos after *N. bombycis* congenital infection. We assume that microsporidia deploy their miRNAs to attenuate silkworm immune responses by suppressing the expression of the related immune genes. In addition, one *N. bombycis* miRNA (novel-m0172-3p) is possible to target ATP binding cassette subfamily D member 3 (ABCD3) to inhibit β-oxidation of fatty acid to reduce lipid consumption for parasite multiplication. It seems that cross-species miRNAs play an important role in host-microsporidia interactions.

## Discussion

Microsporidia, naturally occurring obligate intracellular fungal-related parasites, have been at the forefront of efforts to develop biocontrol agents for agriculture and forestry pests due to their host specificity and transovarial transmission [50–52]. As a typical species of microsporia, *N. bombycis* is an important pathogen of silkworm *B. mori* causing the epidemic disease pébrine through horizontal and vertical transmission. *N. bombycis* may be a model of congenital infection for studying microsporidia pathogenicity and pathogen-host interactions. In microsporidia-host interplays, insects recognize pathogens by its innate immune system, and parasites attenuated host immune responses through various molecular mechanisms [53]. However, these underlying mechanisms remain elusive. Here, we showed the pan-transcriptome including the coding RNA and ncRNAs for *N. bombycis* and host *B. mori* with a specific focus on microsporidia congenital infection, and suggest that ncRNA-mediated regulation plays a vital role in the microsporidia congenital infection, which provides solid foundation to study fungal pathogenicity and pathogen-host interactions in embryos and larvae.

Non-coding RNAs comprise various RNA species, including miRNA, lncRNA and circRNA, which are recognized as key regulators of gene expression to regulate various physiological processes, including growth, development, metabolism and immune responses [54–56]. Predictably, we obtained 4,046 reference genes and 125 novel genes compared with the 4,460 annotated genes in *N. bombycis* (http://silkpathdb.swu.edu.cn). Furthermore, in our study we identified that 403 lncRNAs and 284 miRNAs in *N. bombycis,* which are higher abundance compared to the corresponding species reported for *N. ceranae* [20, 32, 57]. Recent studies suggest that *N. bombycis* modulates small RNA (sRNA)-mediated regulation that increases fungal pathogenicity [15].

We found that a large number of mRNAs were identified in *N. bombycis* at various stages of infection, only a handful of genes were stage-specifically expressed genes for each stage*.* Similarly, there were 351 lncRNAs (87.34% of all predicted *N. bombycis* lncRNAs) and 241 miRNAs (84.86% of all predicted *N. bombycis* miRNAs) that were identified in all four above mentioned stages. However, regarding circRNAs, it is not the same case, only 17 of 62 circRNAs were detected in all four stages of infection, suggesting that the major part of circRNAs encoded from *N. bombycis* exhibit specific development or stress-inducible expression patterns. CircRNAs usually show specific cell-type, tissue, and developmental expression patterns in animals and plants [58, 59]. CircRNAs also exhibit abiotic and biotic stress-inducible expression patterns [60, 61].

As expected, we found that a variety of DE mRNAs, lncRNAs, and miRNAs encoded by *N. bombycis* in the process of *N. bombycis* infecting silkworm embryos and larvae, suggesting that those mRNAs, lncRNAs, and miRNAs may play a critical role in the biological processes of *N. bombycis* in silkworms. In contrast, only three *N. bombycis* circRNAs were differentially expressed during the process. Interestingly, PTP3 was significantly downregulated in 1, 5, 10-day larvae infected with *N. bombycis* compared with 5-day infected embryos. Previous studies have shown that PTP3 is involved in the sporoblast-to-spore polar tube biogenesis and plays a significant role in the control of the polar tube extrusion in *E. cuniculi* [62]. Most importantly, some parasites lncRNAs (MSTRG.7750.2, MSTRG.10054.3, MSTRG.11899.2 and MSTRG.11875.2) were predicted to regulate PTP3 expression by sponging miRNAs. Additionally, two parasite lncRNAs (MSTRG.585.1 and MSTRG.1988.2) were predicted to regulate their neighbor gene PTP3 expression *in cis*, because lncRNAs in less than 100 Kb up/downstream of a gene were likely to be cis-regulators. Interestingly, 32 down-regulated miRNAs of *N. bombycis* were detected in 1-day larvae, compared to 5-day embryos infected with *N. bombycis*. Those highly expressed miRNAs of *N. bombycis* in 5-day embryos may regulate host genes expression to evade the host innate immune response, resulting in contribute to the congenital infection of *N. bombycis* in silkworm embryos. For example, one parasite miRNA (novel-m0044-3p) is highly expressed, and its target Toll ligand Spz3 expression is decreased after *N. bombycis* infection in the embryos. Recent studies showed that Spz3 is mainly involved in the melanization process in the stripe pattern formation in caterpillars [63]. Another melanization factor pro-phenol oxidase plays an important role in defense against intruding parasitoids [64], and is downregulated after *N. bombycis* infection in embryos. These data suggest that microsporidia have evolved to inject parasites miRNAs into the host cells to suppress hemolymph melanization in embryos for the successful development of their progeny. This is an example of the evolution of a cross kingdom miRNA-mediated defense mechanism in microsporidia.

Salmena et al. first proposed that ceRNA cross-talk between RNAs, both coding and non-coding, through miRNA response elements (MREs), forming large-scale regulating network across the transcriptome [65]. CeRNA networks link the function of protein-coding mRNAs with that of non-coding RNAs such as miRNA, lncRNA, and circRNA. In this study, based on known functional genes of *N. bombycis*, we constructed *N. bombycis* lncRNA/ circRNA-miRNA-mRNA ceRNA networks by bioinformatics analysis. We found that one miRNA can bind to different lncRNAs to regulate the same gene. For example, one miRNA (novel-m0174-5p) is predicted to bind to three lncRNAs (MSTRG.2013.1, MSTRG.2923.1 and MSTRG.4851.1) to regulate transposase expression. One lncRNA was predicted to bind to multiple miRNAs to regulate the same expression. For instance, one lncRNA (MSTRG.11875.2) can bind to two miRNAs (novel-m0206-5p and novel-m0224-3p) to regulate PTP3 expression. To our knowledge, this is the comprehensive analysis of microsporidia ncRNAs available, and it will be interest in the future to investigate the function of key lncRNAs, circRNAs, and miRNAs and lncRNA/circRNA-related ceRNA networks in *N. bombycis.*

Regarding silkworms, we found that a total of 14,889 mRNAs, 3,038 lncRNAs, 19,039 circRNAs, and 3,403 miRNAs were identified by the *B. mori* reference genome in the silkworm embryos and larvae with or without the congenital *N. bombycis* infection. Interestingly, for lncRNA, we identified 1,492 lncRNAs with more than 90% homologous sequences reported previously in *B. mori* [66]. And for miRNA, we identified 427 miRNAs with 100% homologous sequences reported previously in *B. mori* [66]. In addition, 19,039 circRNAs and many stage-specific circRNAs were identified, suggesting that circRNAs may play an important role in silkworm development, metabolism, and immune response system. Most importantly, we found that the number of miRNAs was significantly reduced by 50% after *N. bombycis* congenital infection. We also found that the number of small RNAs was twice as much as that of infected samples in small RNA libraries. The data suggest that microsporidia congenital infection greatly interferes with host miRNA biosynthesis.

In the present study, we detected many DE mRNAs during the congenital *N. bombycis* infection. This is consistent with our previous findings that silkworm mRNAs alter during the congenital *N. bombycis* infection in embryos and larvae. KEGG enrichment analysis of silkworm DE mRNAs showed that there were fewer immune-related genes in the embryos on day 5, and more immune-related genes involved in 1, 5, 10-day larvae exposed to the congenital *N. bombycis* challenge. Moreover, the expression of immune-related genes, such as *βGRP2*, *Spz3* and pro-phenol oxidase, decreased in 5-day embryos after infected with *N. bombycis* compared with uninfected samples. While most immune genes, such as peptidoglycan recognition protein like (*PGRP-L*), Toll-like receptor 3 and antimicrobial peptide genes, were upregulated in 1, 5, 10-day larvae exposed to the congenital *N. bombycis* challenge compared with uninfected samples. The results further demonstrated that parasites miRNA-mediated regulation leads to host embryo immunosuppression to contribute to vertical transmission. Of course, the underlying mechanisms of immunosuppression or immune activation in the embryos and larvae exposed to the congenital *N. bombycis* infection remain to be determined. Furthermore, we predicted that there are many ncRNAs (lncRNAs, circRNAs and miRNAs) of silkworms during the congenital *N. bombycis* infection, and plays an important role in the fight against microsporidia infection. For example, we predicted that there were three silkworm genes (alpha-crystallin A chain, HSP25.4, and zinc transporter 2-like) that are likely to be regulated by one lncRNA (MSTRG.23380.1). HSP25.4, a member of the heat shock protein family, is highly expressed following NPV infection in *Antheraea Pernyi* and functions as an immune booster [67]. We assume that lncRNA (MSTRG.23380.1) is possible to participate in the immune response to *N. bombycis* in silkworm by *cis* regulating the expression of HSP25.4. It also has been reported that *N. ceranae*-responsive lncRNAs may regulate the expression of neighboring genes by acting in *cis* fashion [22]. For *B. mori* miRNAs, we found that there was significantly decreased in the number of miRNAs in the silkworm embryos and larvae exposed to the congenital *N. bombycis* challenge, compared with uninfected embryos and larvae. Both miRNA-33 and miRNA-122, which can target lipid metabolism genes to reprogram host lipid metabolism [68–70], are down-regulated during the congenital *N. bombycis* infection. miR-276 is associated with amino acid metabolism [71], which is down-regulated during the congenital *N. bombycis* infection. In addition, miR-306, a conserved miRNA, is worth mentioning as it showed that it is all differentially expressed genes in the silkworm exposed to the congenital *N. bombycis* infection in four different stages, compared with uninfected silkworm embryos and larvae. Some studies have been reported that miR-306 regulates host immune response in the insect during pathogens infection [72, 73]. In this study, we did not investigate translation capacity of these silkworm circRNAs, and regarding circRNA-protein interactions is elusive in microsporidia infection. Therefore, these ncRNAs may play an important role in the host–pathogen interaction, and the underlying regulatory mechanism requires further investigation.

Recent evidence has shown that lncRNA and circRNA, acting as ceRNA, have an important role in regulating miRNA target genes in host–pathogen interactions in insect. In the study, the lncRNA-miRNA-mRNA and circRNA-miRNA-mRNA pathways were established, respectively. Moreover, based on eight immune-related genes, which was differentially expressed in silkworm exposed to the congenital *N. bombycis* challenge, we predicted lncRNA-miRNA-mRNA pathways and circRNA-miRNA-mRNA pathways, respectively. Two LncRNAs (MSTRG.20503.1 and MSTRG.9320.1) was predicted to modulate the TBC1d14 gene by acting as a decoy of miR-8523 during the silkworm immune response to the congenital *N. bombycis* stress. A circRNA (circ_002617) may function as a ceRNA to regulate the *Toll-6* gene by acting as a decoy of bmo-miR-283-5p in immune response to the congenital *N. bombycis* infection. Therefore, the regulatory networks of the lncRNAs and circRNAs need to be proven in the future.

In summary, we analyzed the pan-transcriptome including the coding RNA (mRNA) and ncRNAs (lncRNA, circRNA and miRNA) for both parasites and their host silkworms at different development stages. This is the first pan-transcriptome and cross-talk of the coding and non-coding RNAs in the microsporidia: *N. bombycis* congenital infection of silkworm embryos and larvae, and shows that ncRNA-mediated regulation plays a vital role in the microsporidia congenital infection, which provides new insights into understanding the basic biology of microsporidia and their host–pathogen interaction.

### Supplementary Information

**Additional file 1:** **Fig. S1.** Validation of the microsporidia spores by H&E staining and TEM in N. bombycis congenitally infected silkworm embryos and larvae. 5-day embryos (A), 1-day larvae (B), 5-day larvae (C), and 10-day larvae (D) from the parent silkworms infected with N. bombycis were used to detect the microsporidia spores by H&E staining; 5-day embryos (E), 1-day larvae (F), 5-day larvae (G), and 10-day larvae (H) from the parent silkworms infected with N. bombycis were used to detect the microsporidia spores by TEM. Black arrow: yolks; Yellow arrow: muscle cells; Red arrow: adipocyte cells; Blue arrow: epidermic cell. Green arrow: spores. **Fig. S2.** Principal component analysis (PCA) was performed to evaluate a separation between different stages and study groups. A: PCA was performed based on mRNA data; B: PCA was performed based on lncRNA data; C: PCA was performed based on circRNA data; D: PCA was performed based on miRNA data. **Fig. S3.** KEGG enrichment analysis of significant differential microsporidia genes between 5-day embryos, 1-day larvae, 5-day larvae, and 10-day larvae challenged by N. bombycis congenital infection. A: KEGG enrichment analysis of cluster I microsporidia genes; B: KEGG enrichment analysis of cluster II microsporidia genes; C: KEGG enrichment analysis of cluster III microsporidia genes; D: KEGG enrichment analysis of cluster IV microsporidia genes; E: KEGG enrichment analysis of cluster V microsporidia genes. **Fig. S4.** KEGG enrichment analysis of significant differential microsporidia miRNA target genes between 5-day embryos, 1-day larvae, 5-day larvae, and 10-day larvae challenged by N. bombycis congenital infection. A: KEGG enrichment analysis of cluster I miRNA target genes; B: KEGG enrichment analysis of cluster II miRNA target genes; C: KEGG enrichment analysis of cluster III miRNA target genes; D: KEGG enrichment analysis of cluster IV miRNA target genes. **Fig. S5** KEGG enrichment analysis of significant differential B. mori genes in silkworm embryos and larvae during the N. bombycis congenital infection. A: KEGG enrichment analysis of significant differential B. mori mRNAs in silkworm 5-day embryos exposed to N. bombycis congenital challenge; B: KEGG enrichment analysis of significant differential B. mori mRNAs in silkworm 1-day larvae exposed to N. bombycis congenital challenge; C: KEGG enrichment analysis of significant differential B. mori mRNAs in silkworm 5-day larvae exposed to N. bombycis congenital challenge; D: KEGG enrichment analysis of significant differential B. mori mRNAs in silkworm 10-day larvae exposed to N. bombycis congenital challenge. **Fig. S6.** KEGG enrichment analysis of DE lncRNA cis-target genes in silkworm embryos and larvae during the N. bombycis congenital infection. **Fig. S7.** KEGG enrichment analysis of DE circRNA source genes in silkworm embryos and larvae during the N. bombycis congenital infection. A: KEGG enrichment analysis of DE circRNA source genes in silkworm 5-day embryos during the N. bombycis congenital infection; B: KEGG enrichment analysis of DE circRNA source genes in silkworm 1-day larvae during the N. bombycis congenital infection; C: KEGG enrichment analysis of DE circRNA source genes in silkworm 5-day larvae during the N. bombycis congenital infection; D: KEGG enrichment analysis of DE circRNA source genes in silkworm 10-day larvae during the N. bombycis congenital infection. **Fig. S8.** KEGG enrichment analysis of DE miRNA target genes in silkworm embryos and larvae during the N. bombycis congenital infection. A: KEGG enrichment analysis of DE miRNA target genes in silkworm 5-day embryos during the N. bombycis congenital infection; B: KEGG enrichment analysis of DE miRNA target genes in silkworm 1-day larvae during the N. bombycis congenital infection; C: KEGG enrichment analysis of DE miRNA target genes in silkworm 5-day larvae during the N. bombycis congenital infection; D: KEGG enrichment analysis of DE miRNA target genes in silkworm 10-day larvae during the N. bombycis congenital infection.

**Additional file 2: Table S1.** Novel genes of N. bombycis were identified in the microsporidia congenital infection in silkworm embryos and larvae. **Table S2.** Stage-specifically expressed genes of N. bombycis in the microsporidia congenital infection in silkworm embryos and larvae. **Table S3.** Differentially expressed mRNA, lncRNA, and miRNA of N. bombycis between four stages. **Table S4.** LncRNA-mRNA analysis (lncRNA antisense, cis and trans-regulation) of N. bombycis. **Table S5.** N.bombycis miRNA-target genes analysis. **Table S6.** LncRNA-miRNA-mRNA ceRNA networks of N. bombycis in the microsporidia congenitally infected silkworm embryos and larvae. **Table S7.** CircRNA -miRNA-mRNA ceRNA networks of N. bombycis in the microsporidia congenitally infected silkworm embryos and larvae. **Table S8.** The connection degree of each gene of N. bombycis in the ceRNA networks. **Table S9.** Novel genes of silkworm were identified in the microsporidia congenital infection in silkworm embryos and larvae. **Table S10.** The lncRNA-mRNA pairs including 99 silkworm lncRNAs and 105 mRNAs were predicted in the microsporidia congenitally infected silkworm embryos and larvae. **Table S11.** LncRNA-miRNA-mRNA networks of silkworm in the 5th day of N. bombycis congenitally infected silkworm embryos. **Table S12.** CircRNA-miRNA-mRNA networks of silkworm in the 5th day of N. bombycis congenitally infected silkworm embryos. **Table S13.** LncRNA-miRNA-mRNA networks of silkworm in the 1th day of N. bombycis congenitally infected silkworm larvae. **Table S14.** CircRNA-miRNA-mRNA networks of silkworm in the 1th day of N. bombycis congenitally infected silkworm larvae. **Table S15.** LncRNA-miRNA-mRNA networks of silkworm in the 5th day of N. bombycis congenitally infected silkworm larvae. **Table S16.** CircRNA-miRNA-mRNA networks of silkworm in the 5th day of N. bombycis congenitally infected silkworm larvae. **Table S17.** LncRNA-miRNA-mRNA networks of silkworm in the 10th day of N. bombycis congenitally infected silkworm larvae. **Table S18.** CircRNA-miRNA-mRNA networks of silkworm in the 10th day of N. bombycis congenitally infected silkworm larvae. **Table S19.** The connection degree of each gene of silkworm in the lncRNA ceRNA network during the N. bombycis congenital infection. **Table S20.** The connection degree of each gene of silkworm in the circRNA ceRNA network during the N. bombycis congenital infection.

## Data Availability

The raw data generated was submitted to the National Center for Biotechnology Information (NCBI) Sequence Read Archive (SRA, http://www.ncbi.nlm.nih.gov/sra) with the BioProject accession number (PRJNA953616).

## Abbreviations

ncRNAs: Non-coding RNAs
lncRNA: Long non-coding RNA
circRNA: Covalently closed circular RNA
microRNA: MiRNA
mRNA: Messenger RNA
PTP3: Polar tube protein 3
SWP4: Spore wall protein 4
HSP90: Heat shock protein 90
iNOS: Inducible nitric oxide synthase
Spz4: Spätzle4
CLIPB9: CLIP-domain serine protease
RNAi: RNA interference
Ago: Argonaute
pre-mRNA: Precursor mRNA
ceRNA: Competing endogenous RNA
HE: Hematoxylin & eosin
TEM: Transmission electron microscopy
FPKM: Fragment per kilobase of transcript per million mapped reads
PBS: Phosphate buffer saline
PCR: Polymerase chain reaction
DE: Differentially expressed
RPAC1: DNA-directed RNA polymerases I and III subunit
ABC transporter M2: ABC transporter B family member 2
ABC transporter M24: ABC transporter B family member 24
KEGG: Kyoto Encyclopedia of Genes and Genomes
βGRP2: Beta-1,3-glucan recognition protein 2
PGRP: Peptidoglycan recognition protein like
Hemolin-IP: Hemolin-interacting protein
TBC1d14: TBC1 domain family member 14
ABCD3: ATP binding cassette subfamily D member 3
NCBI: National Center for Biotechnology Information
SRA: Sequence Read Archive
