# Supplementary material for: Non-coding RNAs identification and regulatory networks in pathogen-host interaction in the microsporidia congenital infection
Source: BMC Genomics. 2023 Jul 26;24:420. doi: 10.1186/s12864-023-09490-3 (PMC10373312; doi:10.1186/s12864-023-09490-3)
Supplement: Supplementary file 1 — Additional file 1: Fig. S1. Validation of the microsporidia spores by H&E staining and TEM in N. bombycis congenitally infected silkworm embryos and larvae. 5-day embryos (A), 1-day larvae (B), 5-day larvae (C), and 10-day larvae (D) from the parent silkworms infected with N. bombycis were used to detect the microsporidia spores by H&E staining; 5-day embryos (E), 1-day larvae (F), 5-day larvae (G), and 10-day larvae (H) from the parent silkworms infected with N. bombycis were used to detect the microsporidia spores by TEM. Black arrow: yolks; Yellow arrow: muscle cells; Red arrow: adipocyte cells; Blue arrow: epidermic cell. Green arrow: spores. Fig. S2. Principal component analysis (PCA) was performed to evaluate a separation between different stages and study groups. A: PCA was performed based on mRNA data; B: PCA was performed based on lncRNA data; C: PCA was performed based on circRNA data; D: PCA was performed based on miRNA data. Fig. S3. KEGG enrichment analysis of significant differential microsporidia genes between 5-day embryos, 1-day larvae, 5-day larvae, and 10-day larvae challenged by N. bombycis congenital infection. A: KEGG enrichment analysis of cluster I microsporidia genes; B: KEGG enrichment analysis of cluster II microsporidia genes; C: KEGG enrichment analysis of cluster III microsporidia genes; D: KEGG enrichment analysis of cluster IV microsporidia genes; E: KEGG enrichment analysis of cluster V microsporidia genes. Fig. S4. KEGG enrichment analysis of significant differential microsporidia miRNA target genes between 5-day embryos, 1-day larvae, 5-day larvae, and 10-day larvae challenged by N. bombycis congenital infection. A: KEGG enrichment analysis of cluster I miRNA target genes; B: KEGG enrichment analysis of cluster II miRNA target genes; C: KEGG enrichment analysis of cluster III miRNA target genes; D: KEGG enrichment analysis of cluster IV miRNA target genes. Fig. S5 KEGG enrichment analysis of significant differential B. mori genes i [file 12864_2023_9490_MOESM1_ESM.docx]

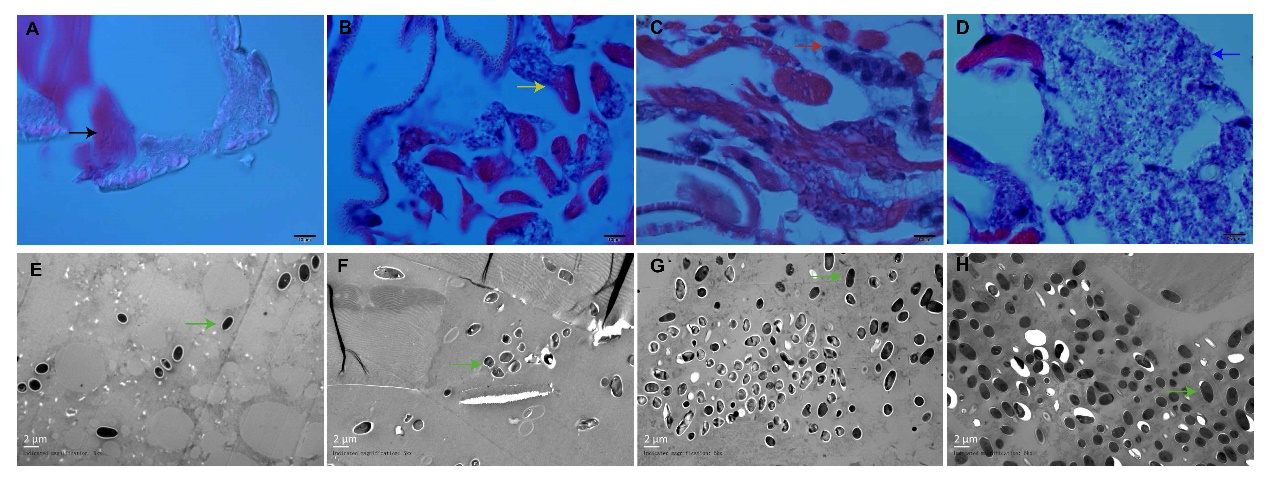


Fig. S1 Validation of the microsporidia spores by H&E staining and TEM in *N. bombycis* congenitally infected silkworm embryos and larvae.

5-day embryos (A), 1-day larvae (B), 5-day larvae (C), and 10-day larvae (D) from the parent silkworms infected with *N. bombycis* were used to detect the microsporidia spores by H&E staining; 5-day embryos (E), 1-day larvae (F), 5-day larvae (G), and 10-day larvae (H) from the parent silkworms infected with *N. bombycis* were used to detect the microsporidia spores by TEM. Black arrow: yolks; Yellow arrow: muscle cells; Red arrow: adipocyte cells; Blue arrow: epidermic cell. Green arrow: spores.


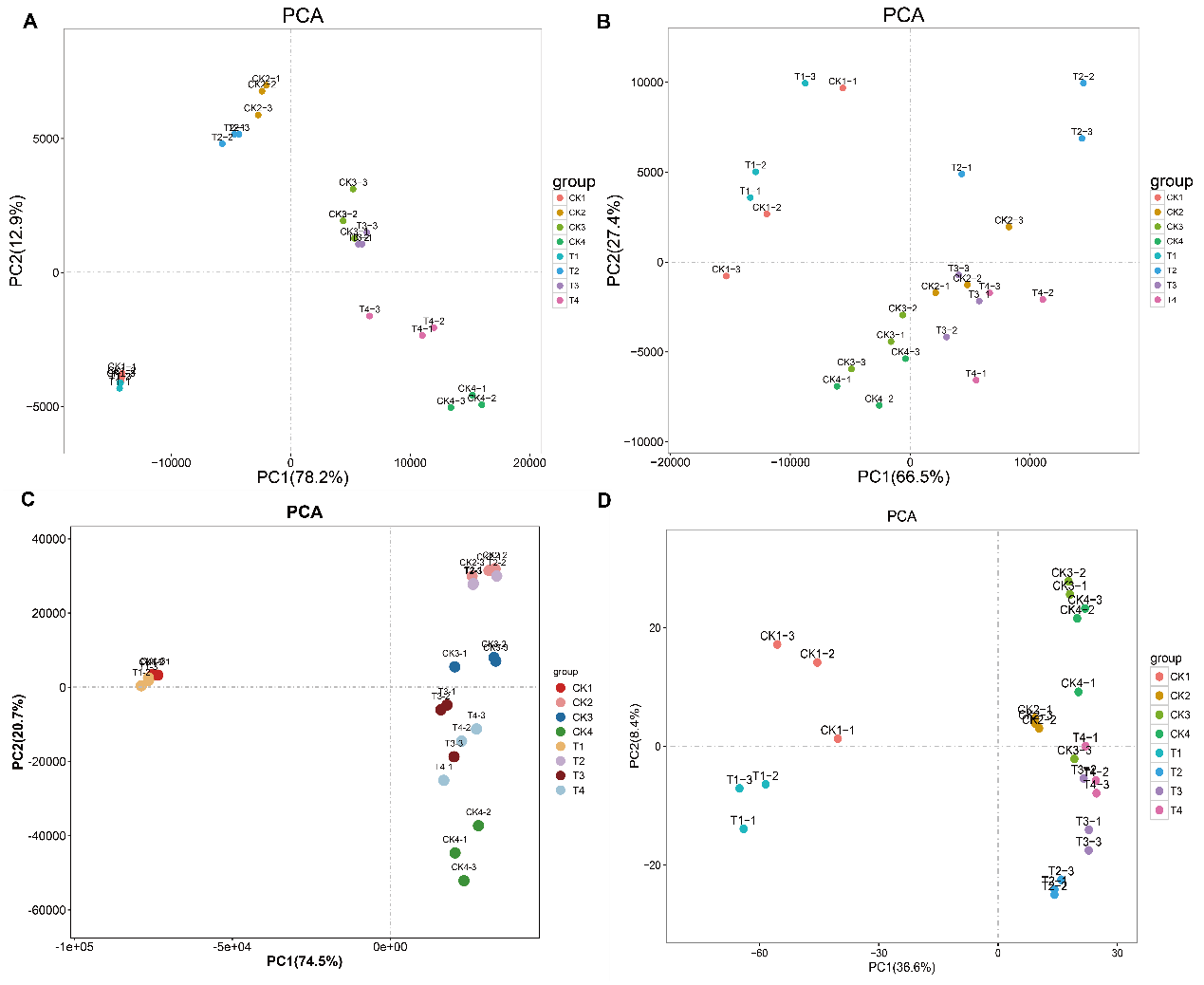


Fig. S2 Principal component analysis (PCA) was performed to evaluate a separation between different stages and study groups. A: PCA was performed based on mRNA data; B: PCA was performed based on lncRNA data; C: PCA was performed based on circRNA data; D: PCA was performed based on miRNA data.


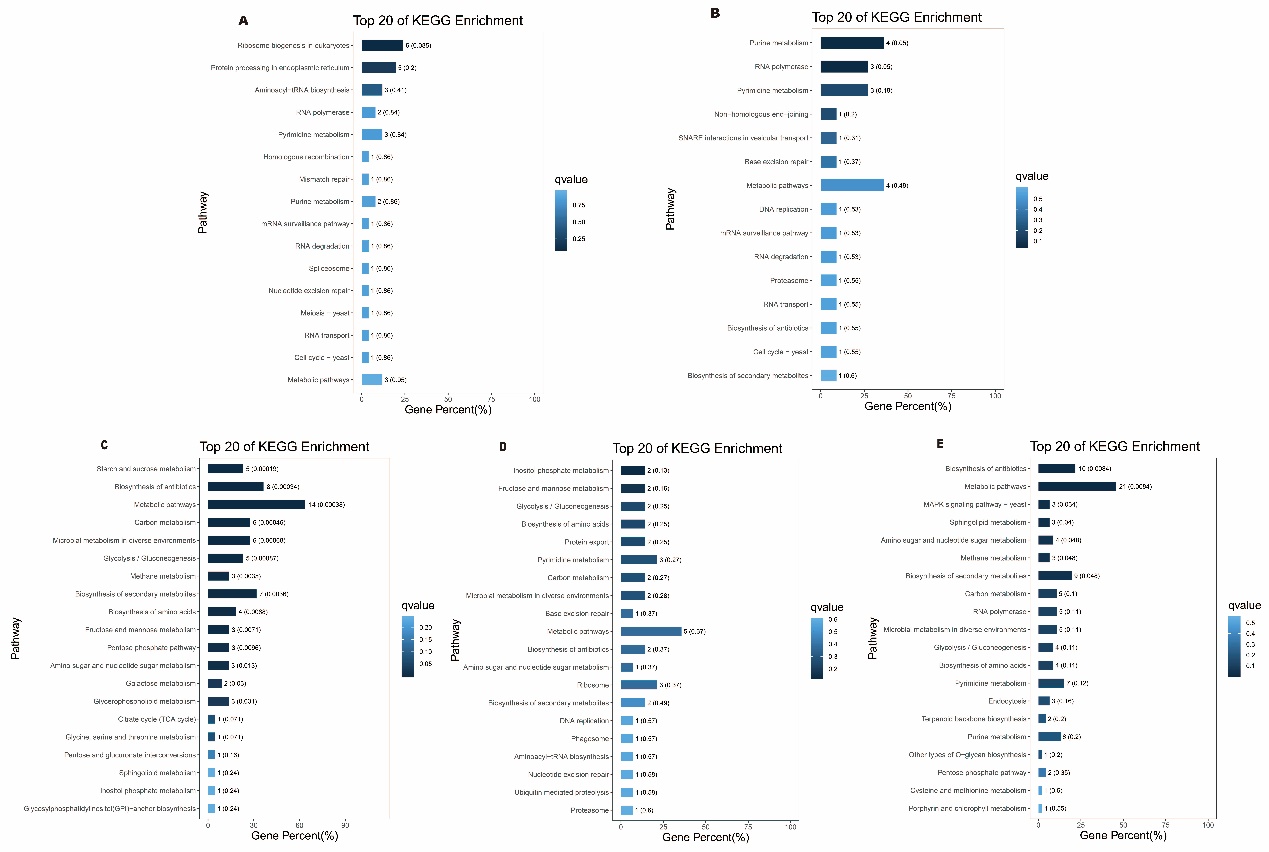


Fig. S3 KEGG enrichment analysis of significant differential microsporidia genes between 5-day embryos, 1-day larvae, 5-day larvae, and 10-day larvae challenged by *N. bombycis* congenital infection.

A: KEGG enrichment analysis of cluster I microsporidia genes; B: KEGG enrichment analysis of cluster II microsporidia genes; C: KEGG enrichment analysis of cluster III microsporidia genes; D: KEGG enrichment analysis of cluster IV microsporidia genes; E: KEGG enrichment analysis of cluster V microsporidia genes.


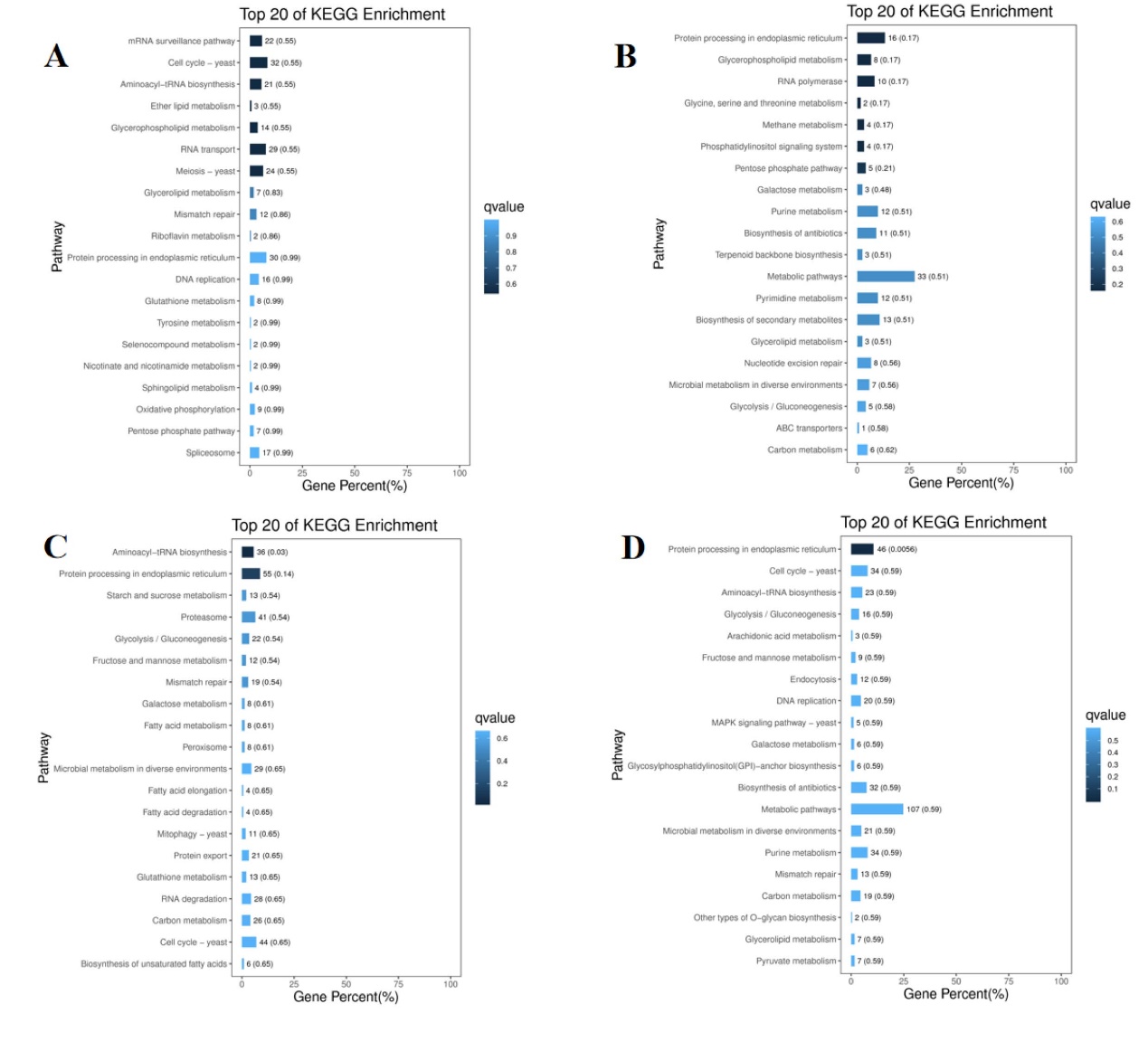


Fig. S4 KEGG enrichment analysis of significant differential microsporidia miRNA target genes between 5-day embryos, 1-day larvae, 5-day larvae, and 10-day larvae challenged by *N. bombycis* congenital infection.

A: KEGG enrichment analysis of cluster I miRNA target genes; B: KEGG enrichment analysis of cluster II miRNA target genes; C: KEGG enrichment analysis of cluster III miRNA target genes; D: KEGG enrichment analysis of cluster IV miRNA target genes.


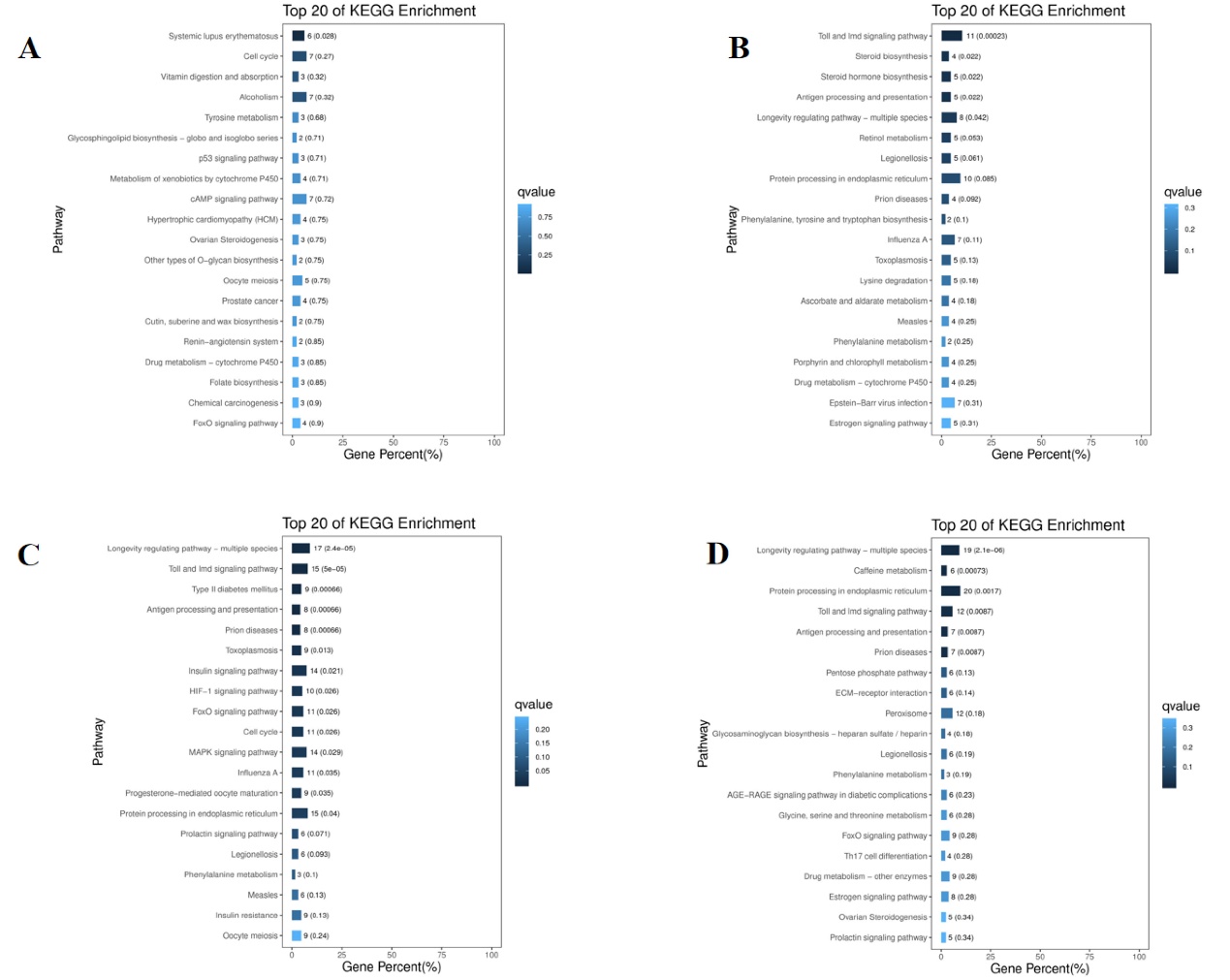


Fig. S5 KEGG enrichment analysis of significant differential *B. mori* genes in silkworm embryos and larvae during the *N. bombycis* congenital infection

A: KEGG enrichment analysis of significant differential *B. mori* mRNAs in silkworm 5-day embryos exposed to *N. bombycis* congenital challenge; B: KEGG enrichment analysis of significant differential *B. mori* mRNAs in silkworm 1-day larvae exposed to *N. bombycis* congenital challenge; C: KEGG enrichment analysis of significant differential *B. mori* mRNAs in silkworm 5-day larvae exposed to *N. bombycis* congenital challenge; D: KEGG enrichment analysis of significant differential *B. mori* mRNAs in silkworm 10-day larvae exposed to *N. bombycis* congenital challenge.


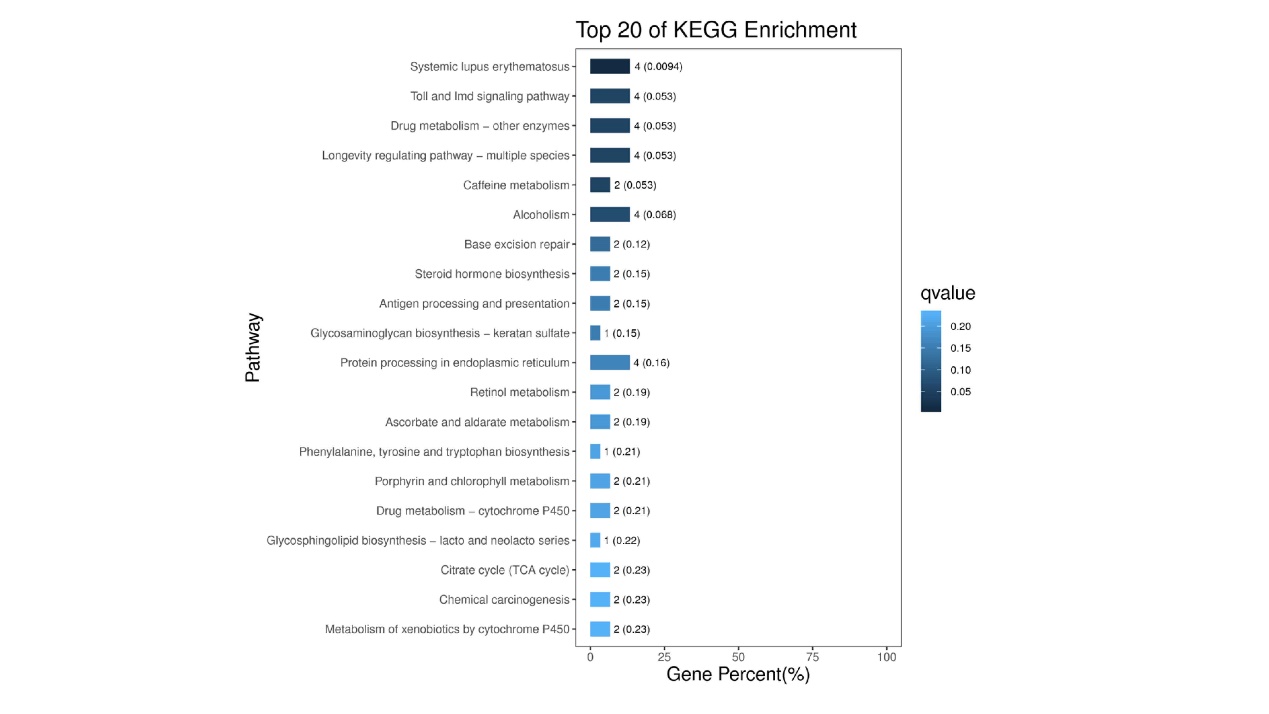


Fig. S6 KEGG enrichment analysis of DE lncRNA cis-target genes in silkworm embryos and larvae during the *N. bombycis* congenital infection


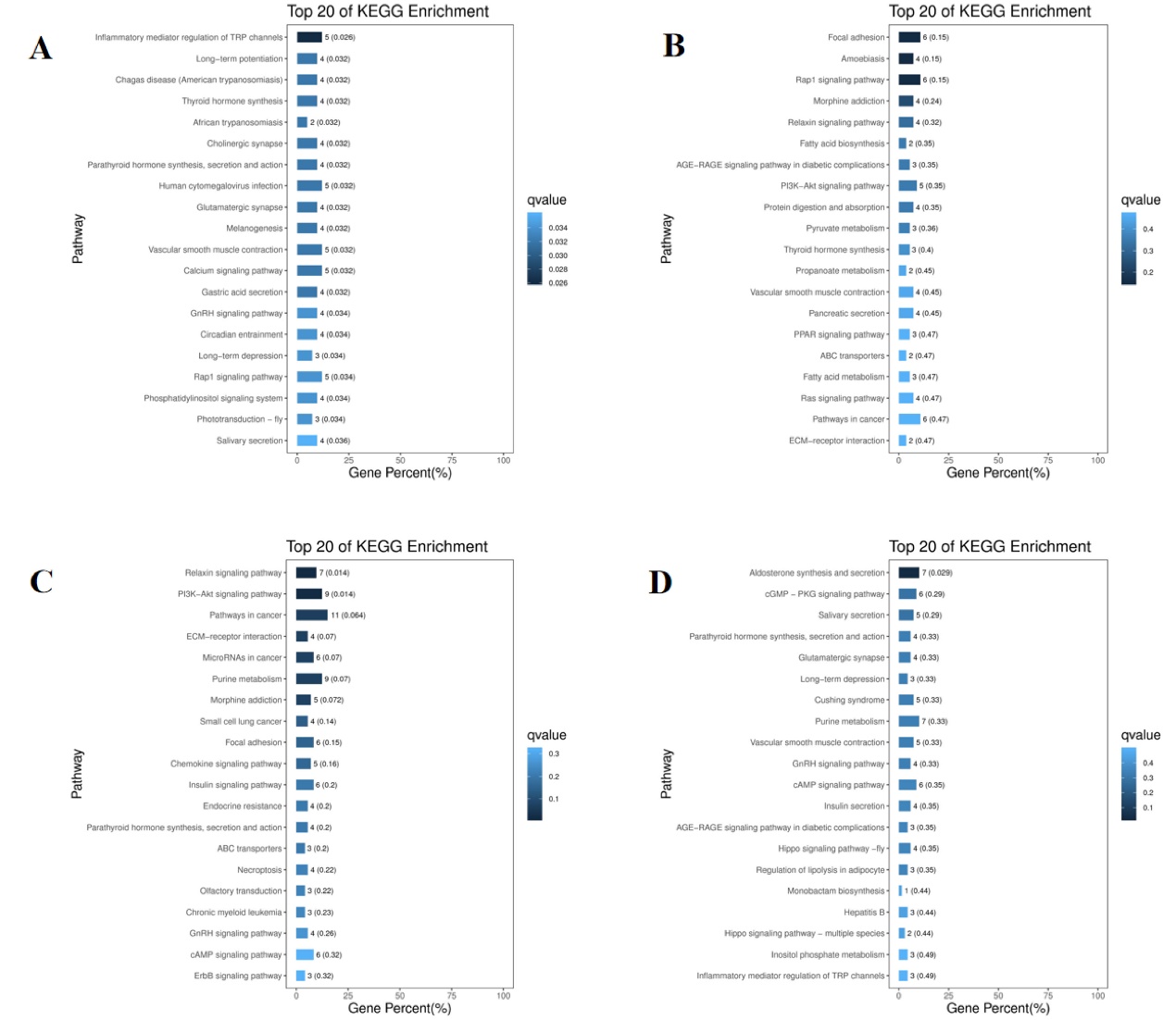


Fig. S7 KEGG enrichment analysis of DE circRNA source genes in silkworm embryos and larvae during the *N. bombycis* congenital infection

A: KEGG enrichment analysis of DE circRNA source genes in silkworm 5-day embryos during the *N. bombycis* congenital infection; B: KEGG enrichment analysis of DE circRNA source genes in silkworm 1-day larvae during the *N. bombycis* congenital infection; C: KEGG enrichment analysis of DE circRNA source genes in silkworm 5-day larvae during the *N. bombycis* congenital infection; D: KEGG enrichment analysis of DE circRNA source genes in silkworm 10-day larvae during the *N. bombycis* congenital infection.


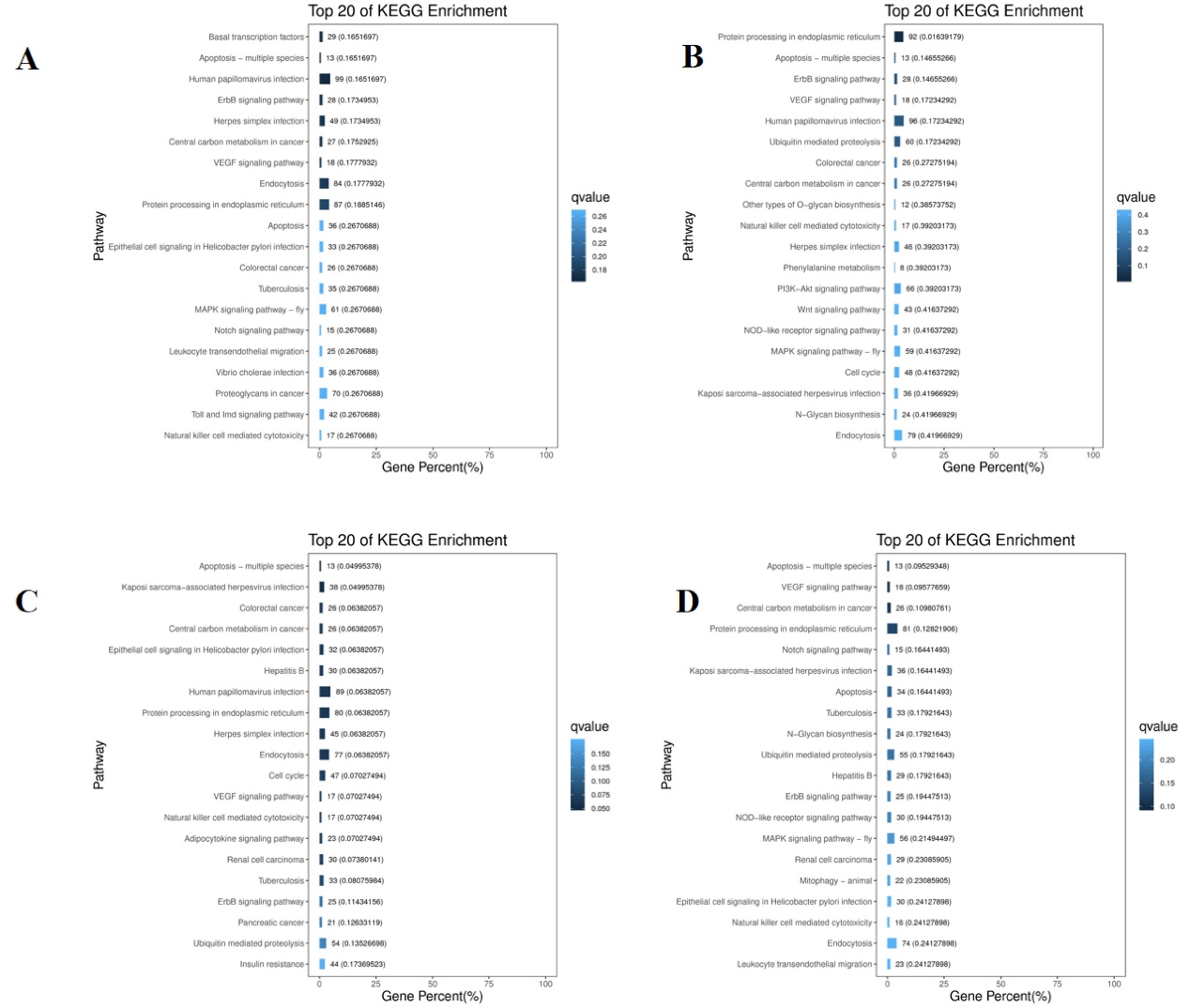


Fig. S8 KEGG enrichment analysis of DE miRNA target genes in silkworm embryos and larvae during the *N. bombycis* congenital infection

A: KEGG enrichment analysis of DE miRNA target genes in silkworm 5-day embryos during the *N. bombycis* congenital infection; B: KEGG enrichment analysis of DE miRNA target genes in silkworm 1-day larvae during the *N. bombycis* congenital infection; C: KEGG enrichment analysis of DE miRNA target genes in silkworm 5-day larvae during the *N. bombycis* congenital infection; D: KEGG enrichment analysis of DE miRNA target genes in silkworm 10-day larvae during the *N. bombycis* congenital infection.
