# Supplementary material for: Non-coding RNAs identification and regulatory networks in pathogen-host interaction in the microsporidia congenital infection
Source: BMC Genomics. 2023 Jul 26;24:420. doi: 10.1186/s12864-023-09490-3 (PMC10373312; doi:10.1186/s12864-023-09490-3)
Supplement: Supplementary file 2 — Additional file 2: Table S1. Novel genes of N. bombycis were identified in the microsporidia congenital infection in silkworm embryos and larvae. Table S2. Stage-specifically expressed genes of N. bombycis in the microsporidia congenital infection in silkworm embryos and larvae. Table S3. Differentially expressed mRNA, lncRNA, and miRNA of N. bombycis between four stages. Table S4. LncRNA-mRNA analysis (lncRNA antisense, cis and trans-regulation) of N. bombycis. Table S5. N.bombycis miRNA-target genes analysis. Table S6. LncRNA-miRNA-mRNA ceRNA networks of N. bombycis in the microsporidia congenitally infected silkworm embryos and larvae. Table S7. CircRNA -miRNA-mRNA ceRNA networks of N. bombycis in the microsporidia congenitally infected silkworm embryos and larvae. Table S8. The connection degree of each gene of N. bombycis in the ceRNA networks. Table S9. Novel genes of silkworm were identified in the microsporidia congenital infection in silkworm embryos and larvae. Table S10. The lncRNA-mRNA pairs including 99 silkworm lncRNAs and 105 mRNAs were predicted in the microsporidia congenitally infected silkworm embryos and larvae. Table S11. LncRNA-miRNA-mRNA networks of silkworm in the 5th day of N. bombycis congenitally infected silkworm embryos. Table S12. CircRNA-miRNA-mRNA networks of silkworm in the 5th day of N. bombycis congenitally infected silkworm embryos. Table S13. LncRNA-miRNA-mRNA networks of silkworm in the 1th day of N. bombycis congenitally infected silkworm larvae. Table S14. CircRNA-miRNA-mRNA networks of silkworm in the 1th day of N. bombycis congenitally infected silkworm larvae. Table S15. LncRNA-miRNA-mRNA networks of silkworm in the 5th day of N. bombycis congenitally infected silkworm larvae. Table S16. CircRNA-miRNA-mRNA networks of silkworm in the 5th day of N. bombycis congenitally infected silkworm larvae. Table S17. LncRNA-miRNA-mRNA networks of silkworm in the 10th day of N. bombycis congenitally infected silkworm larvae. Table S18. [file 12864_2023_9490_MOESM2_ESM.docx]

| **Table S1 Novel genes of *N. bombycis* were identified in the microsporidia congenital infection in silkworm embryos and larvae** | | |
| --- | --- | --- |
| GeneID | Symbol | Description |
| MSTRG.10238 | -- | ankyrin repeat-containing protein [Nosema bombycis CQ1] |
| MSTRG.10270 | -- | hypothetical protein NBO_354g0002 [Nosema bombycis CQ1] |
| MSTRG.1057 | -- | hypothetical protein NBO_43g0003 [Nosema bombycis CQ1] |
| MSTRG.10701 | -- | - |
| MSTRG.10858 | -- | hypothetical protein NBO_78g0008 [Nosema bombycis CQ1] |
| MSTRG.11026 | -- | hypothetical protein NBO_67g0011 [Nosema bombycis CQ1] |
| MSTRG.11031 | -- | hypothetical protein NBO_4g0044 [Nosema bombycis CQ1] |
| MSTRG.1108 | -- | hypothetical protein NBO_499gi001, partial [Nosema bombycis CQ1] |
| MSTRG.11334 | -- | hypothetical protein NBO_429g0003 [Nosema bombycis CQ1] |
| MSTRG.1149 | pol | krab-a domain-containing protein, partial [Nosema ceranae] |
| MSTRG.11623 | ECU01_0140 | M1 family aminopeptidase 1 [Nosema bombycis CQ1] |
| MSTRG.1182 | -- | hypothetical protein NBO_18g0017 [Nosema bombycis CQ1] |
| MSTRG.12428 | -- | PI3/PI4 kinase family like protein [Nosema bombycis CQ1] |
| MSTRG.12950 | -- | hypothetical protein NBO_40g0002 [Nosema bombycis CQ1] |
| MSTRG.13276 | -- | Zinc (Zn2+)-Iron (Fe2+) Permease (ZIP) Family [Trachipleistophora hominis] |
| MSTRG.1396 | -- | transcription initiation factor tfiid subunit [Nosema ceranae] |
| MSTRG.14040 | -- | hypothetical protein NBO_11g0042 [Nosema bombycis CQ1] |
| MSTRG.14183 | -- | - |
| MSTRG.14271 | -- | hypothetical spore wall protein 14 [Nosema bombycis] |
| MSTRG.1515 | -- | hypothetical protein AAJ76_2200003265, partial [Nosema ceranae] |
| MSTRG.1578 | -- | hypothetical protein NAPIS_ORF01137 [Nosema apis BRL 01] |
| MSTRG.1618 | tc1a | transposase [Nosema bombycis] |
| MSTRG.1871 | -- | DNA helicase PIF1/RRM3 [Trachipleistophora hominis] |
| MSTRG.1885 | -- | - |
| MSTRG.1895 | -- | - |
| MSTRG.1898 | -- | - |
| MSTRG.1899 | -- | - |
| MSTRG.1908 | -- | - |
| MSTRG.1909 | -- | - |
| MSTRG.1911 | -- | - |
| MSTRG.1917 | -- | hypothetical protein NBO_6g0074 [Nosema bombycis CQ1] |
| MSTRG.1990 | PIF1 | ATP-dependent DNA helicase PIF1 [Hypsizygus marmoreus] |
| MSTRG.2048 | -- | hypothetical protein NBO_57g0003 [Nosema bombycis CQ1] |
| MSTRG.2104 | -- | Proteoglycan-4 [Nosema bombycis CQ1] |
| MSTRG.2169 | -- | threonyl-tRNA synthetase [Nosema bombycis CQ1] |
| MSTRG.2183 | abcB2 | ATP-binding cassette sub-family B member 7, mitochondrial, partial [Nosema bombycis CQ1] | | |
| MSTRG.223 | -- | hypothetical protein NBO_1086g0001 [Nosema bombycis CQ1] | | |
| MSTRG.2412 | -- | - | | |
| MSTRG.2451 | -- | - | | |
| MSTRG.2470 | -- | DNA replication fork-blocking protein FOB1, partial [Nosema bombycis CQ1] | | |
| MSTRG.266 | -- | hypothetical protein NBO_1071g0001 [Nosema bombycis CQ1] | | |
| MSTRG.3032 | -- | hypothetical protein NBO_8g0009 [Nosema bombycis CQ1] | | |
| MSTRG.3119 | -- | MULE transposase domain protein [Spraguea lophii 42_110] | | |
| MSTRG.3156 | -- | glucose transporter type 3 [Nosema bombycis CQ1] | | |
| MSTRG.3391 | -- | hypothetical spore wall protein [Nosema bombycis] | | |
| MSTRG.3393 | -- | hypothetical protein NBO_569g0002 [Nosema bombycis CQ1] | | |
| MSTRG.3484 | -- | krab-a domain-containing protein 2-like protein [Nosema ceranae] | | |
| MSTRG.3561 | -- | hypothetical spore wall protein 14 [Nosema bombycis] | | |
| MSTRG.3633 | TIGD1 | Tigger transposable element-derived protein 1 [Smittium culicis] | | |
| MSTRG.3872 | -- | krab-a domain-containing protein 2-like protein [Nosema ceranae] | | |
| MSTRG.4001 | -- | ATP-dependent DNA helicase PIF1 [Hypsizygus marmoreus] | | |
| MSTRG.4004 | -- | transcriptional factor B3 [Mycena chlorophos] | | |
| MSTRG.4051 | -- | - | | |
| MSTRG.4126 | -- | hypothetical protein NBO_542g0002 [Nosema bombycis CQ1] | | |
| MSTRG.414 | asnA | aspartate-ammonia ligase [Nosema ceranae] | | |
| MSTRG.4141 | -- | - | | |
| MSTRG.415 | -- | hypothetical protein NCER_101562 [Nosema ceranae BRL01] | | |
| MSTRG.4180 | -- | - | | |
| MSTRG.4184 | -- | - | | |
| MSTRG.4188 | -- | reverse transcriptase [Nosema ceranae] | | |
| MSTRG.4316 | -- | - | | |
| MSTRG.4355 | pif1 | atp-dependent helicase rrm3-like protein, partial [Nosema ceranae] | | |
| MSTRG.4557 | -- | krab-a domain-containing protein 2-like protein [Nosema ceranae] | | |
| MSTRG.4585 | TIGD1 | Tigger transposable element-derived protein 6 [Rhizoctonia solani AG-1 IB] | | |
| MSTRG.466 | SWP25 | RecName: Full=Spore wall protein 25; Flags: Precursor | | |
| MSTRG.5336 | -- | - | | |
| MSTRG.5484 | pol | krab-a domain-containing protein [Nosema apis BRL 01] | | |
| MSTRG.5534 | -- | hypothetical protein NBO_24g0021 [Nosema bombycis CQ1] | | |
| MSTRG.5535 | -- | hypothetical protein NBO_24g0021 [Nosema bombycis CQ1] | | |
| MSTRG.5613 | -- | serine protease inhibitor 106 [Nosema bombycis] | | |
| MSTRG.5720 | -- | hypothetical spore wall protein 14 [Nosema bombycis] | | |
| MSTRG.5781 | EP3 | endochitinase [Nosema ceranae] | | |
| MSTRG.5879 | -- | hypothetical protein NBO_67g0010 [Nosema bombycis CQ1] | | |
| MSTRG.63 | -- | hypothetical spore wall protein 14 [Nosema bombycis] | | |
| MSTRG.6316 | -- | Transcription-associated protein 1 [Nosema bombycis CQ1] | | |
| MSTRG.635 | -- | DNA replication fork-blocking protein FOB1, partial [Nosema bombycis CQ1] | | |
| MSTRG.6740 | -- | - | | |
| MSTRG.701 | -- | n-acetylglucosaminylphosphatidylinositol de-n-acetylase family protein [Nosema apis BRL 01] | | |
| MSTRG.7066 | -- | - | | |
| MSTRG.7103 | -- | hypothetical protein NBO_69g0010 [Nosema bombycis CQ1] | | |
| MSTRG.7149 | -- | Chromosome segregation ATPase [Nosema bombycis CQ1] | | |
| MSTRG.7168 | -- | hypothetical protein NBO_13g0021 [Nosema bombycis CQ1] | | |
| MSTRG.7278 | -- | Tricalbin-2, partial [Nosema bombycis CQ1] | | |
| MSTRG.733 | -- | - | | |
| MSTRG.75 | -- | hypothetical protein H312_01097 [Anncaliia algerae PRA339] | | |
| MSTRG.7523 | catp-8 | CATION-TRANSPORTING ATPase [Encephalitozoon cuniculi GB-M1] | | |
| MSTRG.754 | -- | n-acetylglucosaminylphosphatidylinositol de-n-acetylase family protein [Nosema apis BRL 01] | | |
| MSTRG.7603 | -- | - | | |
| MSTRG.7604 | -- | hypothetical protein NBO_569g0002 [Nosema bombycis CQ1] | | |
| MSTRG.7807 | -- | - | | |
| MSTRG.7825 | -- | hypothetical protein NBO_494g0003 [Nosema bombycis CQ1] | | |
| MSTRG.785 | GTF2IRD2 | unknown [Nosema bombycis] | | |
| MSTRG.8023 | -- | trans-sialidase [Cordyceps brongniartii RCEF 3172] | | |
| MSTRG.820 | -- | hypothetical protein NBO_6g0115 [Nosema bombycis CQ1] | | |
| MSTRG.831 | -- | threonyl-tRNA synthetase [Nosema bombycis CQ1] | | |
| MSTRG.8648 | -- | glucose transporter type 3 [Nosema bombycis CQ1] | | |
| MSTRG.8707 | -- | zinc finger protein [Nosema bombycis CQ1] | | |
| MSTRG.8933 | -- | hypothetical protein NP_05A10 [Nosema pernyi] | | |
| MSTRG.9337 | -- | piggybac-derived 2 (agap012114-pa) [Nosema apis BRL 01] | | |
| MSTRG.9496 | -- | hypothetical spore wall protein [Nosema bombycis] | | |
| MSTRG.9691 | -- | hypothetical protein NBO_1049g0001 [Nosema bombycis CQ1] | | |
| MSTRG.9770 | -- | unknown [Nosema bombycis] | | |
| MSTRG.9776 | -- | hypothetical protein NBO_429g0001 [Nosema bombycis CQ1] | | |
| MSTRG.9947 | -- | hypothetical protein NBO_459g0001, partial [Nosema bombycis CQ1] | |

| **Table S2 Stage-specifically expressed genes of *N. bombycis* in the microsporidia congenital infection in silkworm embryos and larvae** | | | |
| --- | --- | --- | --- |
| Stage-specifically expressed genes of *N. bombycis* in 5-day embryos | | | |
| ID | Description | | |
| NBO_13g0039 | hypothetical protein | | |
| NBO_1191gi001 | arginine/serine rich pre-mRNA splicing factor | | |
| NBO_602g0001 | hypothetical protein | | |
| NBO_1198gi001 | t-SNARE affecting a late Golgi compartment protein 2 | | |
| NBO_16g0026 | udp-glucose pyrophosphorylase | | |
| NBO_8g0015 | hypothetical protein | | |
| NBO_139g0001 | hypothetical protein | | |
| NBO_284g0002 | hypothetical protein | | |
| NBO_2g0070 | hypothetical protein | | |
| NBO_364g0011 | hypothetical protein | | |
| NBO_386g0019 | hypothetical protein | | |
| NBO_53g0004 | hypothetical protein | | |
| NBO_53g0024 | hypothetical protein | | |
| NBO_78g0016 | ATP-binding/permease protein cydC | | |
| NBO_809g0001 | hypothetical protein | | |
| NBO_894g0001 | NBO_894g0001 | | |
| NBO_89g0005 | Thioredoxin-like protein 4A | | |
| NBO_463g0001 | Ricin B lectin | | |
| NBO_1358g0001 | hypothetical protein | | |
| Stage-specifically expressed genes of *N. bombycis* in 1-day larvae | | | |
| NBO_1198gi001 | hypothetical protein | | |
| NBO_1191gi001 | Peptidase aspartic | | |
| NBO_16g0026 | RING finger protein 44 | | |
| NBO_602g0001 | hypothetical protein | | |
| NBO_13g0039 | hypothetical protein | | |
| NBO_284g0002 | hypothetical protein | | |
| NBO_8g0015 | hypothetical protein | | |
| NBO_809g0001 | exosome complex exonuclease rrp4 | | |
| NBO_78g0016 | Eukaryotic translation initiation factor 5B | | |
| NBO_89g0005 | hypothetical protein | | |
| NBO_894g0001 | hypothetical protein | | |
| NBO_53g0004 | hypothetical protein | | |
| NBO_2g0070 | hypothetical protein | | |
| NBO_386g0019 | hypothetical protein | | |
| NBO_364g0011 | NBO_491g0005 | | |
| NBO_139g0001 | hypothetical protein | | |
| NBO_53g0024 | hypothetical protein | | |
| NBO_463g0001 | hypothetical protein | | |
| NBO_1358g0001 | hypothetical protein | | |
| NBO_1198gi001 | hypothetical protein | | |
| NBO_1191gi001 | hypothetical protein | | |
| NBO_16g0026 | amino acid permease | | |
| Stage-specifically expressed genesof *N. bombycis* in 5-day larvae | | | |
| NBO_6g0018 | hypothetical protein | | |
| NBO_461g0002 | hypothetical protein | | |
| NBO_582g0001 | tristetraproline | | |
| NBO_270g0003 | H/ACA ribonucleoprotein complex subunit 1 | | |
| NBO_2g0002 | hypothetical protein | | |
| NBO_221g0001 | hypothetical protein | | |
| NBO_41g0022 | hypothetical protein | | |
| NBO_83g0008 | Putative zinc finger protein | | |
| NBO_1365gi001 | hypothetical protein | | |
| NBO_1506g0001 | hypothetical protein | | |
| NBO_1591g0001 | hypothetical protein | | |
| NBO_238g0002 | hypothetical protein | | |
| NBO_24g0027 | hypothetical protein | | |
| NBO_26g0006 | hypothetical protein | | |
| NBO_607g0001 | hypothetical protein | | |
| NBO_643g0001 | hypothetical protein | | |
| NBO_3g0040 | Eukaryotic translation initiation factor 3 | | |
| NBO_10g0031 | hypothetical protein | | |
| NBO_1435g0001 | hypothetical protein | | |
| NBO_218g0003 | hypothetical protein | | |
| NBO_28g0001 | hypothetical protein | | |
| NBO_29g0002 | small nuclear ribonucleoprotein Sm D1 | | |
| NBO_29g0040 | hypothetical protein | | |
| NBO_353g0001 | hypothetical protein | | |
| NBO_421g0005 | hypothetical protein | | |
| NBO_438g0001 | hypothetical protein | | |
| NBO_44g0006 | serine protease inhibitor 106 | | |
| NBO_54g0008 | hypothetical protein | | |
| NBO_57g0005 | hypothetical protein | | |
| NBO_70g0010 | hypothetical protein | | |
| NBO_8g0019 | hypothetical protein | | |
| NBO_95g0001 | hypothetical protein | | |
| NBO_812g0002 | hypothetical protein | | |
| NBO_1015g0002 | nuclear scaffold like protein | | |
| NBO_10g0108 | hypothetical protein | | |
| NBO_119g0001 | hypothetical protein | | |
| NBO_11g0028 | hypothetical protein | |
| NBO_1206g0002 | hypothetical protein | |
| NBO_1280g0001 | hypothetical protein | |
| NBO_16g0055 | hypothetical protein | |
| NBO_20g0013 | hypothetical protein | |
| NBO_27g0022 | hypothetical protein | |
| NBO_28g0016 | Homeobox protein HD-11 | |
| NBO_317g0004 | hypothetical protein | |
| NBO_36g0006 | hypothetical protein | |
| NBO_389g0010 | hypothetical protein | |
| NBO_40g0003 | hypothetical protein | |
| NBO_462g0005 | hypothetical protein | |
| NBO_48g0001 | hypothetical protein | |
| NBO_495g0004 | hypothetical protein | |
| NBO_498g0004 | hypothetical protein | |
| NBO_4g0059 | hypothetical protein | |
| NBO_508g0024 | hypothetical protein | |
| NBO_519g0001 | hypothetical protein | |
| NBO_562g0001 | hypothetical protein | |
| NBO_632g0002 | hypothetical protein | |
| NBO_662g0001 | hypothetical protein | |
| NBO_66g0007 | Ribulose-phosphate 3-epimerase | |
| NBO_69g0012 | hypothetical protein | |
| NBO_73g0030 | hypothetical protein | |
| NBO_799g0001 | hypothetical protein | |
| NBO_919g0003 | hypothetical protein | |
| NBO_94g0002 | cdc73-like rna polymerase ii accessory factor | |
| NBO_2g0003 | hypothetical protein | |
| NBO_64g0013 | SEC18-like vesicular fusion protein | |
| Stage-specifically expressed genes of *N. bombycis* in 10-day larvae | | |
| NBO_29g0006 | hypothetical protein | |
| NBO_41g0026 | phospholipid-transporting ATPase IIA | |
| NBO_1173g0004 | hypothetical protein | |
| NBO_27g0056 | transcription initiation factor TFIIE subunit alpha | |
| NBO_4g0060 | hypothetical protein | |
| NBO_1100g0001 | hypothetical protein | |
| NBO_11g0076 | hypothetical protein | |
| NBO_1341g0003 | hypothetical protein | |
| NBO_1391g0001 | hypothetical protein | |
| NBO_24g0025 | hypothetical protein | |
| NBO_468g0003 | hypothetical protein | |
| NBO_582g0003 | hypothetical protein | |
| NBO_655g0003 | Transcription-associated recombination protein |
| NBO_750g0002 | hypothetical protein |
| NBO_80g0003 | hypothetical protein |
| NBO_2g0016 | transcriptional repressor for RNA polymerase II, partial |
| NBO_58g0011 | Casein kinase II subunit alpha |
| NBO_1059gi001 | deoxyuridine 5'triphosphate nucleotidohydrolase |
| NBO_1303gi001 | IMP4-like U3 small nucleolar ribonucleoprotein |
| MSTRG.5535 | hypothetical protein |
| MSTRG.7523 | Cation-transporting ATPase |
| NBO_10g0090 | hypothetical protein |
| NBO_11g0078 | hypothetical protein |
| NBO_1604g0001 | hypothetical protein |
| NBO_18g0017 | hypothetical protein |
| NBO_20g0015 | hypothetical protein |
| NBO_222g0002 | hypothetical protein |
| NBO_2g0055 | hypothetical protein |
| NBO_33g0010 | hypothetical protein |
| NBO_33g0029 | hypothetical protein |
| NBO_375g0001 | hypothetical protein |
| NBO_38g0033 | hypothetical protein |
| NBO_445g0003 | hypothetical protein |
| NBO_458g0007 | hypothetical protein |
| NBO_575g0001 | hypothetical protein |
| NBO_6g0052 | hypothetical protein |
| NBO_6g0064 | hypothetical protein |
| NBO_744g0001 | hypothetical protein |
| NBO_795g0001 | hypothetical protein |
| NBO_820g0002 | hypothetical protein |
| NBO_895g0001 | hypothetical protein |

| **Table S3 Differentially expressed mRNA, lncRNA, and miRNA of *N. bombycis* between four stages** | | |
| --- | --- | --- |
| GeneID (mRNA) | GeneID (lncRNA) | GeneID (miRNA) |
| NBO_169g0003 | MSTRG.2412.2 | novel−m0149−3p |
| NBO_108g0004 | MSTRG.6264.1 | novel−m0029−5p |
| NBO_59g0003 | MSTRG.1777.1 | novel−m0025−3p |
| NBO_6g0049 | MSTRG.3464.1 | novel−m0072−5p |
| NBO_1303g0002 | MSTRG.5419.1 | novel−m0065−3p |
| NBO_66g0005 | MSTRG.3258.2 | novel−m0211−3p |
| NBO_12g0014 | MSTRG.2734.1 | novel−m0142−5p |
| NBO_7g0058 | MSTRG.4271.1 | novel−m0136−5p |
| NBO_911gi001 | MSTRG.1726.1 | novel−m0102−5p |
| NBO_26g0007 | MSTRG.3576.1 | novel−m0131−5p |
| NBO_73g0016 | MSTRG.11751.2 | novel−m0174−5p |
| NBO_355g0002 | MSTRG.8795.2 | novel−m0201−3p |
| NBO_78g0013 | MSTRG.11349.1 | novel−m0087−5p |
| NBO_20g0008 | MSTRG.11349.2 | novel−m0191−3p |
| NBO_33g0020 | MSTRG.3258.1 | novel−m0179−3p |
| NBO_18g0007 | MSTRG.4289.1 | novel−m0114−3p |
| NBO_448g0002 | MSTRG.4851.1 | novel−m0168−3p |
| NBO_204g0002 | MSTRG.77.1 | novel−m0105−5p |
| NBO_76g0027 | MSTRG.2013.1 | novel−m0041−3p |
| NBO_13g0067 | MSTRG.8004.2 | novel−m0083−3p |
| NBO_1170gi001 | MSTRG.1660.2 | novel−m0044−3p |
| NBO_4g0017 | MSTRG.89.1 | novel−m0046−3p |
| NBO_1203g0001 | MSTRG.1988.2 | novel−m0153−3p |
| NBO_468g0005 | MSTRG.12005.1 | novel−m0047−3p |
| NBO_633g0001 | MSTRG.3814.1 | novel−m0054−3p |
| NBO_53g0003 | MSTRG.4862.2 | novel−m0091−5p |
| NBO_6g0086 | MSTRG.12180.2 | novel−m0093−3p |
| MSTRG.4051 | MSTRG.2359.2 | novel−m0071−5p |
| NBO_63g0015 | MSTRG.1911.2 | novel−m0047−5p |
| NBO_6g0040 | MSTRG.10368.1 | novel−m0153−5p |
| NBO_464g0008 | MSTRG.1988.1 | novel−m0107−5p |
| NBO_4g0048 | MSTRG.8150.1 | novel−m0003−3p |
| NBO_389g0001 | MSTRG.11875.2 | novel−m0118−3p |
| NBO_6g0030 | MSTRG.10054.3 | novel−m0026−3p |
| NBO_16g0051 | MSTRG.12084.1 | novel−m0165−3p |
| NBO_333g0001 | MSTRG.7750.2 | novel−m0163−5p |
| NBO_362g0010 | MSTRG.11899.2 | novel−m0167−5p |
| NBO_71g0003 | MSTRG.12083.1 | novel−m0010−5p |
| NBO_10g0037 | MSTRG.10794.1 | novel−m0184−3p |
| NBO_696g0002 | MSTRG.5153.2 | novel−m0189−3p |
| NBO_108g0002 | MSTRG.5153.3 | novel−m0177−3p |
| NBO_1010g0001 | MSTRG.5153.1 | novel−m0207−5p |
| NBO_1015g0001 | MSTRG.12418.1 | novel−m0175−5p |
| NBO_36g0002 | MSTRG.2172.1 | novel−m0101−5p |
| NBO_54g0012 | MSTRG.2172.2 | novel−m0028−5p |
| NBO_47g0001 | MSTRG.2173.1 | novel−m0089−5p |
| NBO_915g0002 | MSTRG.2236.1 | novel−m0104−5p |
| NBO_1302g0002 | MSTRG.10216.1 | novel−m0212−5p |
| NBO_11g0069 | MSTRG.11951.2 | novel−m0040−5p |
| NBO_43g0006 | MSTRG.585.1 | novel−m0043−5p |
| NBO_23g0003 | MSTRG.11348.1 | novel−m0077−5p |
| NBO_55g0007 | MSTRG.1912.1 | novel−m0099−3p |
| NBO_63g0018 | MSTRG.4848.1 | novel−m0138−3p |
| NBO_13g0057 | MSTRG.11513.1 | novel−m0140−3p |
| NBO_396g0002 | MSTRG.2923.1 | novel−m0090−3p |
| NBO_452g0005 |  | novel−m0226−3p |
| NBO_1209gi001 |  | novel−m0015−3p |
| NBO_443g0001 |  | novel−m0204−3p |
| NBO_1157g0003 |  | novel−m0222−3p |
| NBO_12g0010 |  | novel−m0076−3p |
| NBO_19g0026 |  | novel−m0109−3p |
| NBO_507g0012 |  | novel−m0082−3p |
| NBO_10g0053 |  | novel−m0169−3p |
| NBO_6g0037 |  | novel−m0229−3p |
| NBO_54g0020 |  | novel−m0196−3p |
| NBO_28g0046 |  | novel−m0228−3p |
| NBO_468g0001 |  | novel−m0148−3p |
| MSTRG.11026 |  | novel−m0079−5p |
| NBO_1608g0001 |  | novel−m0224−3p |
| NBO_401g0008 |  | novel−m0061−3p |
| NBO_13g0046 |  | novel−m0213−3p |
| NBO_6g0088 |  | novel−m0002−3p |
| NBO_64gi001 |  | novel−m0172−3p |
| NBO_32g0034 |  | novel−m0022−5p |
| NBO_198gi002 |  | novel−m0080−5p |
| NBO_3g0049 |  | novel−m0206−5p |
| NBO_64g0035 |  | novel−m0100−3p |
| NBO_28g0052 |  | novel−m0094−3p |
| NBO_6g0004 |  | novel−m0023−5p |
| NBO_28g0019 |  | novel−m0110−5p |
| NBO_740g0001 |  | novel−m0086−5p |
| NBO_384g0005 |  | novel−m0199−5p |
| NBO_925g0001 |  | novel−m0070−3p |
| NBO_929g0004 |  | novel−m0080−3p |
| NBO_16g0023 |  | novel−m0129−3p |
| NBO_554g0002 |  | novel−m0147−5p |
| NBO_20g0002 |  | novel−m0112−3p |
| NBO_6g0048 |  | novel−m0158−5p |
| NBO_29g0016 |  | novel−m0063−5p |
| NBO_1078g0002 |  | novel−m0030−3p |
| NBO_16g0027 |  | novel−m0116−3p |
| NBO_58g0026 |  | novel−m0117−5p |
| NBO_920g0001 |  | novel−m0035−5p |
| NBO_547g0003 |  | novel−m0197−3p |
| NBO_616g0003 |  | novel−m0161−3p |
| NBO_3g0033 |  | novel−m0152−3p |
| NBO_10g0028 |  | novel−m0145−3p |
| NBO_63g0025 |  | novel−m0120−3p |
| NBO_66g0050 |  | novel−m0133−3p |
| NBO_32g0024 |  | novel−m0044−5p |
| NBO_84g0003 |  | novel−m0046−5p |
| NBO_66g0051 |  | novel−m0010−3p |
| NBO_15g0005 |  | novel−m0164−3p |
| NBO_30g0008 |  | novel−m0121−3p |
| NBO_64g0030 |  | novel−m0108−3p |
| NBO_11g0032 |  | novel−m0119−3p |
| NBO_451g0007 |  | novel−m0170−5p |
| NBO_73g0017 |  | novel−m0138−5p |
| NBO_18g0012 |  | novel−m0103−5p |
| NBO_48g0004 |  | novel−m0122−5p |
| NBO_10g0110 |  | novel−m0050−3p |
| NBO_338g0001 |  | novel−m0192−3p |
| NBO_10g0023 |  | novel−m0061−5p |
| NBO_1140g0001 |  | novel−m0045−5p |
| NBO_974g0001 |  | novel−m0174−3p |
| NBO_73g0026 |  | novel−m0050−5p |
| NBO_81g0015 |  | novel−m0192−5p |
| NBO_27gi003 |  | novel−m0043−3p |
| NBO_364g0016 |  | novel−m0066−3p |
| NBO_38g0031 |  | novel−m0106−3p |
| NBO_281g0003 |  | novel−m0074−5p |
| NBO_10g0027 |  | novel−m0100−5p |
| NBO_1552gi001 | -- | novel−m0205−5p |
| NBO_360g0001 | -- | novel−m0104−3p |
| NBO_55g0012 | -- | novel−m0212−3p |
| NBO_13g0031 | -- | novel−m0149−3p |
| NBO_480g0001 |  | novel−m0029−5p |
| NBO_1271g0002 | -- | novel−m0025−3p |
| NBO_503g0004 | -- | novel−m0072−5p |
| NBO_707g0002 | -- | novel−m0065−3p |
| NBO_11g0045 | -- | novel−m0211−3p |
| NBO_376g0007 | -- | novel−m0142−5p |
| NBO_401g0003 |  | novel−m0136−5p |
| NBO_508g0029 |  | novel−m0102−5p |
| NBO_455g0003 | -- | novel−m0131−5p |
| NBO_63g0020 | -- | novel−m0174−5p |
| NBO_369g0001 |  | novel−m0201−3p |
| NBO_11g0052 | -- | novel−m0087−5p |
| NBO_2gi003 | -- | novel−m0191−3p |
| NBO_4g0029 | -- | novel−m0179−3p |
| NBO_6g0118 |  |  |
| NBO_10g0052 |  |  |
| NBO_2g0071 | -- |  |
| NBO_365g0001 |  |  |
| NBO_1332gi002 | -- |  |
| NBO_76gi004 | -- | - |
| NBO_424g0002 | -- |  |
| NBO_568g0004 | -- |  |
| NBO_10g0094 | -- |  |
| NBO_19g0018 |  |  |
| NBO_48g0008 | -- |  |
| NBO_10g0055 | -- | - |
| NBO_800gi001 | -- | - |
| NBO_359g0004 | -- | - |
| NBO_48g0007 | -- | - |
| MSTRG.8023 | -- | - |
| NBO_3g0006 | -- | - |
| NBO_33gi001 | -- | - |
| NBO_78g0012 | -- |  |
| NBO_58g0022 |  |  |
| NBO_974g0002 | -- |  |
| NBO_33g0017 | -- |  |
| NBO_63g0022 | -- |  |
| NBO_174g0001 |  |  | |
| NBO_29g0015 | -- |  | |
| NBO_60g0017 |  |  | |
| NBO_85g0011 | -- | - | |
| NBO_111g0002 | -- | - | |
| NBO_55g0009 | -- |  | |
| NBO_28g0053 | -- |  | |
| NBO_591g0001 | -- |  | |
| NBO_11g0046 |  |  | |
| NBO_26g0013 | -- |  | |
| NBO_76g0013 | -- |  | |
| NBO_1574g0001 | -- |  | |
| NBO_32gi001 | -- |  | |
| NBO_11g0012 | -- |  | |
| NBO_4g0061 | -- |  | |
| NBO_6g0062 |  |  | |
| NBO_362g0011 | -- |  | |
| NBO_4g0049 |  |  | |
| NBO_350g0001 | -- |  | |
| NBO_12g0019 | -- |  | |
| NBO_6gi003 | -- |  | |
| NBO_606g0001 | -- |  | |
| NBO_1133g0001 | -- | - | |
| NBO_85g0005 | -- |  | |
| NBO_27g0019 |  |  | |
| NBO_55g0005 | -- | - | |
| NBO_15g0003 | -- |  | |
| NBO_552g0002 | -- | - | |
| NBO_582gi001 | -- | - | |
| MSTRG.14183 | -- |  | |
| NBO_29g0027 | -- | - | |
| NBO_84g0004 |  |  | |
| NBO_375g0010 |  |  | |
| NBO_49g0003 | -- |  | |
| MSTRG.3032 | -- |  | |
| NBO_7g0015 |  |  | |
| NBO_1116g0003 |  |  | |
| NBO_1000gi001 |  |  | |
| NBO_27g0035 | -- | - | |
| NBO_367g0001 |  |  | |
| NBO_404g0002 | -- |  | |
| NBO_1320g0001 | -- |  | |
| MSTRG.831 | -- |  | |
| NBO_490g0001 | -- |  | |
| NBO_1291g0001 | -- |  | |
| NBO_70g0007 |  |  | |
| MSTRG.2169 | -- |  | |
| NBO_447g0003 | -- |  | |
| NBO_3g0016 | -- |  | |
| NBO_53gi001 | -- |  | |
| NBO_67g0015 | -- |  | |
| NBO_1110g0001 | -- | - | |
| NBO_503g0002 | -- |  | |
| NBO_444g0008 | -- | - | |
| NBO_841g0001 | -- |  | |
| NBO_586gi001 | -- |  | |
| NBO_38g0034 | -- |  | |
| NBO_49g0004 | -- |  | |
| NBO_31gi002 | -- |  | |
| NBO_69g0015 | -- |  | |
| NBO_11g0041 | -- | - | |
| NBO_1218g0002 | -- |  | |
| NBO_11g0003 |  |  | |
| NBO_1110g0002 | -- |  | |
| NBO_114g0004 | -- | - | |
| NBO_3g0004 | -- |  | |
| NBO_58g0013 | -- | - | |
| NBO_1116g0004 | -- |  | |
| NBO_16g0041 |  |  | |
| NBO_29gi002 | -- |  | |
| NBO_378g0013 | -- |  | |
| NBO_444g0002 |  |  | |
| NBO_50g0001 | -- |  | |
| NBO_2g0012 | -- |  | |
| NBO_1118gi001 | -- |  | |
| NBO_28g0026 | -- |  | |
| NBO_2g0038 | -- |  | |
| NBO_32g0022 | -- |  | |
| NBO_16g0013 |  |  | |
| NBO_28g0056 |  |  | |
| NBO_16g0047 |  |  | |
| NBO_28g0048 |  |  | |
| NBO_10g0085 |  |  | |
| NBO_1553gi001 |  |  | |
| NBO_378g0002 |  |  | |
| NBO_18g0028 | -- |  | |
| NBO_81g0010 | -- |  | |
| NBO_429g0001 | -- |  | |
| NBO_29g0020 | -- |  | |
| NBO_466g0001 |  |  | |
| NBO_582g0002 |  |  | |
| NBO_16g0068 |  |  | |
| NBO_76g0016 |  |  | |
| NBO_499g0002 |  |  | |
| NBO_63g0006 |  |  | |
| NBO_429g0002 |  |  | |
| NBO_568g0001 |  |  | |
| NBO_28g0065 |  |  | |
| NBO_49g0001 |  |  | |
| NBO_58g0010 |  |  | |
| NBO_940g0001 |  |  | |
| MSTRG.466 |  |  | |
| NBO_2g0058 |  |  | |
| NBO_29g0031 |  |  | |
| NBO_37g0007 |  |  | |
| NBO_2g0075 |  |  | |
| NBO_552g0005 |  |  | |
| NBO_16g0003 |  |  | |
| NBO_552g0008 |  |  | |
| NBO_4g0050 |  |  | |
| NBO_137g0001 |  |  | |
| NBO_28g0021 |  |  | |
| NBO_29g0013 |  |  | |
| NBO_447g0004 |  |  | |
| NBO_28g0025 |  |  | |
| NBO_63g0005 |  |  | |
| NBO_520g0003 |  |  | |
| NBO_462g0004 |  |  | |
| NBO_6g0085 |  |  | |
| NBO_937g0002 |  |  | |
| NBO_1039g0001 |  |  | |
| NBO_6gi002 |  |  | |
| NBO_20g0024 |  |  | |
| NBO_555g0001 |  |  | |
| NBO_81g0002 |  |  | |
| NBO_166g0002 |  |  | |
| NBO_549g0007 |  |  | |
| NBO_423g0003 |  |  | |
| NBO_76g0014 |  |  | |
| NBO_58g0009 |  |  | |
| NBO_71g0002 |  |  | |
| NBO_6g0112 |  |  | |
| NBO_1566g0002 |  |  | |
| NBO_417g0006 |  |  | |
| MSTRG.7066 |  |  | |
| MSTRG.63 |  |  | |
| NBO_34g0010 |  |  | |
| NBO_423g0006 |  |  | |
| MSTRG.1990 |  |  | |
| MSTRG.2926 |  |  | |
| MSTRG.8131 |  |  | |
| NBO_1066g0001 |  |  | |
| MSTRG.635 |  |  | |
| NBO_318g0001 |  |  | |
| NBO_1314g0001 |  |  | |
| NBO_32g0021 |  |  | |
| NBO_460g0011 |  |  | |
| NBO_675g0001 |  |  | |
| NBO_9g0004 |  |  | |
| NBO_153g0006 |  |  | |
| NBO_1131g0001 |  |  | |
| NBO_417g0015 |  |  | |
| MSTRG.2750 |  |  | |
| MSTRG.7238 |  |  | |
| NBO_437g0004 |  |  | |
| NBO_16g0024 |  |  | |
| NBO_6g0076 |  |  | |
| NBO_1230g0001 |  |  | |
| NBO_1344g0003 |  |  | |
| NBO_447g0002 |  |  | |
| NBO_401g0004 |  |  | |
| NBO_452g0003 |  |  | |
| NBO_72g0013 |  |  | |
| NBO_69g0004 |  |  | |
| NBO_11g0061 |  |  | |
| NBO_37g0005 |  |  | |
| NBO_9g0005 |  |  | |
| NBO_10g0001 |  |  | |
| NBO_74g0004 |  |  | |
| NBO_4g0016 |  |  | |
| NBO_10g0041 |  |  | |
| NBO_73g0028 |  |  | |
| NBO_437g0002 |  |  | |
| NBO_537g0001 |  |  | |
| NBO_1024g0002 |  |  | |
| NBO_85g0001 |  |  | |
| MSTRG.75 |  |  | |
| NBO_1054gi001 |  |  | |
| MSTRG.1911 |  |  | |
| MSTRG.3488 |  |  | |
| MSTRG.1369 |  |  | |
| NBO_483g0001 |  |  | |
| NBO_25g0006 |  |  | |
| MSTRG.2352 |  |  | |
| NBO_39g0002 |  |  | |
| NBO_648gi001 |  |  | |
| NBO_665g0002 |  |  | |
| NBO_979g0001 |  |  | |
| NBO_28g0061 |  |  | |
| NBO_73g0023 |  |  | |
| MSTRG.1400 |  |  | |
| NBO_366gi001 |  |  | |
| MSTRG.4521 |  |  | |
| NBO_6g0060 |  |  | |
| NBO_11gi001 |  |  | |
| MSTRG.5652 |  |  | |
| MSTRG.5960 |  |  | |
| NBO_61g0013 |  |  | |
| NBO_76g0012 |  |  | |
| NBO_16g0010 |  |  | |
| NBO_28g0074 |  |  | |
| NBO_459g0003 |  |  | |
| NBO_32g0025 |  |  | |
| NBO_1530gi001 |  |  | |
| NBO_41g0030 |  |  | |
| NBO_1276g0001 |  |  | |
| NBO_53g0022 |  |  | |
| NBO_80g0017 |  |  | |
| NBO_81g0012 |  |  | |
| NBO_55g0006 |  |  | |
| NBO_380g0001 |  |  | |
| NBO_46g0007 |  |  | |
| MSTRG.6740 |  |  | |
| NBO_60g0027 |  |  | |
| NBO_62g0012 |  |  | |
| NBO_42g0007 |  |  | |
| NBO_929g0005 |  |  | |
| NBO_256g0003 |  |  | |
| NBO_64g0028 |  |  | |
| NBO_41g0024 |  |  | |
| NBO_46g0002 |  |  | |
| NBO_418gi001 |  |  | |
| NBO_417g0013 |  |  | |
| NBO_6g0014 |  |  | |
| MSTRG.128 |  |  | |
| NBO_481g0001 |  |  | |
| NBO_936g0001 |  |  | |
| NBO_24g0012 |  |  | |
| NBO_8g0003 |  |  | |
| NBO_46g0005 |  |  | |
| NBO_13g0005 |  |  | |
| NBO_54g0011 |  |  | |
| MSTRG.8648 |  |  | |
| NBO_76g0003 |  |  | |
| NBO_6g0011 |  |  | |
| NBO_389g0002 |  |  | |
| NBO_27g0061 |  |  | |
| NBO_353g0005 |  |  | |
| NBO_962g0001 |  |  | |
| NBO_1345gi001 |  |  | |
| NBO_1517g0004 |  |  | |
| NBO_27g0024 |  |  | |
| NBO_597g0004 |  |  | |
| NBO_264g0001 |  |  | |
| NBO_27g0025 |  |  | |
| NBO_508g0021 |  |  | |
| NBO_8g0045 |  |  | |
| NBO_23g0004 |  |  | |
| NBO_360g0005 |  |  | |
| NBO_63g0026 |  |  | |
| NBO_7g0005 |  |  | |
| NBO_53g0008 |  |  | |
| NBO_153g0004 |  |  | |
| NBO_73g0012 |  |  | |
| MSTRG.223 |  |  | |
| MSTRG.2412 |  |  | |
| NBO_423g0001 |  |  | |
| NBO_64g0032 |  |  | |
| MSTRG.1057 |  |  | |
| MSTRG.3956 |  |  | |
| NBO_238g0003 |  |  | |
| NBO_417g0011 |  |  | |
| NBO_321g0001 |  |  | |
| NBO_463g0006 |  |  | |
| MSTRG.4180 |  |  | |
| NBO_35g0002 |  |  | |
| NBO_32g0049 |  |  | |
| NBO_401g0005 |  |  | |
| NBO_80g0024 |  |  | |
| NBO_44g0010 |  |  | |
| NBO_12g0008 |  |  | |
| NBO_60g0008 |  |  | |
| NBO_4g0009 |  |  | |
| NBO_9g0003 |  |  | |
| NBO_458g0008 |  |  | |
| NBO_149g0002 |  |  | |
| NBO_975g0001 |  |  | |
| NBO_1053g0002 |  |  | |
| NBO_29g0030 |  |  | |
| NBO_1078g0004 |  |  | |
| NBO_375g0009 |  |  | |
| NBO_19g0014 |  |  | |
| NBO_374g0007 |  |  | |
| NBO_7g0016 |  |  | |
| NBO_943g0004 |  |  | |
| NBO_4g0007 |  |  | |
| NBO_53g0017 |  |  | |
| NBO_943g0003 |  |  | |
| NBO_1302g0001 |  |  | |
| NBO_16g0040 |  |  | |
| NBO_33g0008 |  |  | |
| NBO_100g0001 |  |  | |
| NBO_11g0004 |  |  | |
| NBO_11g0038 |  |  | |
| NBO_609g0001 |  |  | |
| NBO_72g0014 |  |  | |
| NBO_576g0005 |  |  | |
| NBO_80g0034 |  |  | |
| NBO_27g0027 |  |  | |
| NBO_4g0042 |  |  | |
| NBO_24g0011 |  |  | |
| NBO_742g0003 |  |  | |
| NBO_3g0028 |  |  | |
| NBO_404g0004 |  |  | |
| NBO_568g0002 |  |  | |
| NBO_452g0002 |  |  | |
| NBO_32g0030 |  |  | |
| NBO_560g0001 |  |  | |
| NBO_10g0034 |  |  | |
| NBO_1024g0001 |  |  | |
| NBO_34g0043 |  |  | |
| NBO_1230g0003 |  |  | |
| NBO_53g0005 |  |  | |
| NBO_386g0004 |  |  | |
| NBO_1246g0001 |  |  | |
| NBO_20g0004 |  |  | |
| NBO_570g0004 |  |  | |
| NBO_81g0019 |  |  | |
| NBO_1057g0002 |  |  | |
| NBO_1082g0001 |  |  | |
| NBO_64g0031 |  |  | |
| NBO_29g0021 |  |  | |
| NBO_985g0001 |  |  | |
| MSTRG.14040 |  |  | |
| NBO_28g0012 |  |  | |
| NBO_166g0001 |  |  | |
| NBO_261g0001 |  |  | |
| NBO_508g0014 |  |  | |
| NBO_241g0002 |  |  | |
| NBO_11g0058 |  |  | |
| NBO_22g0008 |  |  | |
| NBO_27g0006 |  |  | |
| NBO_462g0003 |  |  | |
| MSTRG.1618 |  |  | |
| NBO_3g0047 |  |  | |
| NBO_13g0072 |  |  | |
| NBO_6g0028 |  |  | |
| NBO_44g0011 |  |  | |
| NBO_770g0002 |  |  | |
| NBO_10g0039 |  |  | |
| NBO_1059gi002 |  |  | |
| NBO_24g0004 |  |  | |
| MSTRG.5613 |  |  | |
| NBO_27g0051 |  |  | |
| NBO_20g0001 |  |  | |
| NBO_76g0021 |  |  | |
| NBO_28g0022 |  |  | |
| NBO_41g0014 |  |  | |
| NBO_53g0011 |  |  | |
| NBO_671gi001 |  |  | |
| MSTRG.4557 |  |  | |
| NBO_29g0012 |  |  | |
| MSTRG.7603 |  |  | |
| MSTRG.9337 |  |  | |
| NBO_753g0001 |  |  | |
| MSTRG.1871 |  |  | |
| MSTRG.4355 |  |  | |
| NBO_411gi001 |  |  | |
| MSTRG.4001 |  |  | |
| NBO_38g0002 |  |  | |
| NBO_3g0041 |  |  | |
| NBO_87g0001 |  |  | |
| NBO_546g0001 |  |  | |
| NBO_1230gi001 |  |  | |
| NBO_376g0002 |  |  | |
| MSTRG.5484 |  |  | |
| NBO_1239g0006 |  |  | |
| MSTRG.1368 |  |  | |
| NBO_1020g0001 |  |  | |
| MSTRG.7278 |  |  | |
| NBO_38g0012 |  |  | |
| NBO_552g0003 |  |  | |
| NBO_6g0033 |  |  | |
| NBO_1232g0001 |  |  | |
| NBO_38g0009 |  |  | |
| MSTRG.1578 |  |  | |
| NBO_845g0001 |  |  | |
| MSTRG.4585 |  |  | |
| NBO_757g0002 |  |  | |
| NBO_92g0005 |  |  | |
| NBO_16g0028 |  |  | |
| NBO_463g0002 |  |  | |
| MSTRG.7314 |  |  | |
| NBO_60g0018 |  |  | |
| NBO_33g0009 |  |  | |
| NBO_1168gi001 |  |  | |
| NBO_293g0001 |  |  | |
| NBO_7g0006 |  |  | |
| NBO_80g0028 |  |  | |
| NBO_1566g0001 |  |  | |
| NBO_7g0040 |  |  | |
| NBO_10g0007 |  |  | |
| NBO_360g0003 |  |  | |
| NBO_6g0058 |  |  | |
| NBO_1053g0001 |  |  | |
| NBO_1135g0001 |  |  | |
| NBO_602gi001 |  |  | |
| NBO_38g0014 |  |  | |
| NBO_1136g0001 |  |  | |
| NBO_32g0012 |  |  | |
| NBO_27g0020 |  |  | |
| NBO_30g0002 |  |  | |
| NBO_28g0050 |  |  | |
| NBO_419g0005 |  |  | |
| NBO_880g0001 |  |  | |
| NBO_376g0001 |  |  | |
| NBO_76g0022 |  |  | |
| NBO_24g0003 |  |  | |
| NBO_928g0001 |  |  | |
| NBO_1086g0001 |  |  | |
| NBO_38g0017 |  |  | |

| **Table S4 LncRNA-mRNA analysis (lncRNA antisense, cis and trans-regulation) of *N. bombycis*** | | |
| --- | --- | --- |
| LncRNA antisense-regulation | | |
| lncRNA | mRNA | Description |
| MSTRG.1912.1 | MSTRG.1911 | - |
| MSTRG.1988.1 | MSTRG.1990 | ATP-dependent DNA helicase PIF1 [Hypsizygus marmoreus] |
| MSTRG.2923.1 | MSTRG.2926 | transposase [Nosema bombycis] |
| MSTRG.5153.1 | NBO_34g0010 | mitochondrial protein import protein MAS5 [Nosema pernyi] |
| MSTRG.5153.2 | NBO_34g0010 | mitochondrial protein import protein MAS5 [Nosema pernyi] |
| MSTRG.5153.3 | NBO_34g0010 | mitochondrial protein import protein MAS5 [Nosema pernyi] |
| MSTRG.77.1 | MSTRG.75 | hypothetical protein H312_01097 [Anncaliia algerae PRA339] |
| LncRNA cis-regulation | | |
| MSTRG.585.1 | NBO_4g0016 | hypothetical protein NBO_4g0016 [Nosema bombycis CQ1] |
| MSTRG.585.1 | NBO_4g0017 | Heat shock protein 101 [Nosema bombycis CQ1] |
| MSTRG.585.1 | NBO_4g0029 | polar tube protein 3 [Nosema bombycis] |
| MSTRG.1911.2 | NBO_10g0001 | dna-directed rna polymerase ii subunit rpb7 [Nosema ceranae] |
| MSTRG.1912.1 | NBO_10g0001 | dna-directed rna polymerase ii subunit rpb7 [Nosema ceranae] |
| MSTRG.1988.1 | NBO_10g0052 | Polar tube protein 3 [Nosema bombycis CQ1] |
| MSTRG.1988.1 | NBO_10g0053 | DNA mismatch repair protein Mlh1 [Nosema bombycis CQ1] |
| MSTRG.1988.2 | NBO_10g0052 | Polar tube protein 3 [Nosema bombycis CQ1] |
| MSTRG.2013.1 | NBO_10g0055 | hypothetical protein NBO_10g0055, partial [Nosema bombycis CQ1] |
| MSTRG.2172.1 | NBO_11gi001 | actin-like 53kDa protein [Nosema bombycis CQ1] |
| MSTRG.2172.1 | MSTRG.2169 | threonyl-tRNA synthetase [Nosema bombycis CQ1] |
| MSTRG.2173.1 | NBO_11gi001 | actin-like 53kDa protein [Nosema bombycis CQ1] |
| MSTRG.2173.1 | MSTRG.2169 | threonyl-tRNA synthetase [Nosema bombycis CQ1] |
| MSTRG.2172.2 | NBO_11gi001 | actin-like 53kDa protein [Nosema bombycis CQ1] |
| MSTRG.2172.2 | MSTRG.2169 | threonyl-tRNA synthetase [Nosema bombycis CQ1] |
| MSTRG.2236.1 | NBO_11g0032 | Peptidase S8 and S53, subtilisin, kexin, sedolisin [Nosema bombycis CQ1] |
| MSTRG.2359.2 | MSTRG.2352 | pol polyprotein [Nosema bombycis] |
| MSTRG.3258.1 | NBO_16g0051 | hypothetical protein NBO_16g0051 [Nosema bombycis CQ1] |
| MSTRG.3258.2 | NBO_16g0051 | hypothetical protein NBO_16g0051 [Nosema bombycis CQ1] |
| MSTRG.10054.3 | NBO_459g0003 | Meiosis-specific protein HOP1 [Nosema bombycis CQ1] |
| MSTRG.10368.1 | NBO_490g0001 | hypothetical protein NBO_490g0001 [Nosema bombycis CQ1] |
| LncRNA trans-regulation | | |
| MSTRG.12180.2 | MSTRG.1057 | hypothetical protein NBO_43g0003 [Nosema bombycis CQ1] |
| MSTRG.1988.2 | MSTRG.1057 | hypothetical protein NBO_43g0003 [Nosema bombycis CQ1] |
| MSTRG.2359.2 | MSTRG.1057 | hypothetical protein NBO_43g0003 [Nosema bombycis CQ1] |
| MSTRG.4862.2 | MSTRG.1057 | hypothetical protein NBO_43g0003 [Nosema bombycis CQ1] |
| MSTRG.2359.2 | MSTRG.1578 | hypothetical protein NAPIS_ORF01137 [Nosema apis BRL 01] |
| MSTRG.12180.2 | MSTRG.1871 | DNA helicase PIF1/RRM3 [Trachipleistophora hominis] |
| MSTRG.4289.1 | MSTRG.1871 | DNA helicase PIF1/RRM3 [Trachipleistophora hominis] |
| MSTRG.1911.2 | MSTRG.1911 | - |
| MSTRG.12180.2 | MSTRG.2412 | - |
| MSTRG.1988.2 | MSTRG.2412 | - |
| MSTRG.3814.1 | MSTRG.2412 | - |
| MSTRG.2013.1 | MSTRG.2926 | transposase [Nosema bombycis] |
| MSTRG.585.1 | MSTRG.2926 | transposase [Nosema bombycis] |
| MSTRG.1988.2 | MSTRG.3956 | pol polyprotein [Nosema apis BRL 01] |
| MSTRG.3814.1 | MSTRG.3956 | pol polyprotein [Nosema apis BRL 01] |
| MSTRG.8795.2 | MSTRG.4521 | pol polyprotein [Nosema bombycis] |
| MSTRG.12180.2 | MSTRG.5613 | serine protease inhibitor 106 [Nosema bombycis] |
| MSTRG.1988.2 | MSTRG.5613 | serine protease inhibitor 106 [Nosema bombycis] |
| MSTRG.3814.1 | MSTRG.5613 | serine protease inhibitor 106 [Nosema bombycis] |
| MSTRG.3576.1 | MSTRG.7278 | Tricalbin-2, partial [Nosema bombycis CQ1] |
| MSTRG.8795.2 | MSTRG.7278 | Tricalbin-2, partial [Nosema bombycis CQ1] |
| MSTRG.3576.1 | MSTRG.7314 | transposable element [Pseudoloma neurophilia] |
| MSTRG.8795.2 | MSTRG.7314 | transposable element [Pseudoloma neurophilia] |
| MSTRG.2013.1 | MSTRG.8131 | pol polyprotein [Nosema bombycis] |
| MSTRG.585.1 | MSTRG.8131 | pol polyprotein [Nosema bombycis] |
| MSTRG.12180.2 | MSTRG.8648 | glucose transporter type 3 [Nosema bombycis CQ1] |
| MSTRG.1988.2 | MSTRG.8648 | glucose transporter type 3 [Nosema bombycis CQ1] |
| MSTRG.3814.1 | MSTRG.8648 | glucose transporter type 3 [Nosema bombycis CQ1] |
| MSTRG.4862.2 | MSTRG.8648 | glucose transporter type 3 [Nosema bombycis CQ1] |
| MSTRG.11899.2 | NBO_1039g0001 | Proteasome component PUP1 [Nosema bombycis CQ1] |
| MSTRG.2236.1 | NBO_1039g0001 | Proteasome component PUP1 [Nosema bombycis CQ1] |
| MSTRG.2013.1 | NBO_1082g0001 | endonuclease [Nosema bombycis CQ1] |
| MSTRG.585.1 | NBO_1082g0001 | endonuclease [Nosema bombycis CQ1] |
| MSTRG.1777.1 | NBO_1086g0001 | hypothetical protein NBO_1086g0001 [Nosema bombycis CQ1] |
| MSTRG.11899.2 | NBO_11g0045 | hypothetical protein NBO_11g0045 [Nosema bombycis CQ1] |
| MSTRG.2236.1 | NBO_11g0045 | hypothetical protein NBO_11g0045 [Nosema bombycis CQ1] |
| MSTRG.4271.1 | NBO_11g0045 | hypothetical protein NBO_11g0045 [Nosema bombycis CQ1] |
| MSTRG.10794.1 | NBO_12g0014 | ribosome biogenesis regulatory protein [Nosema ceranae] |
| MSTRG.5153.2 | NBO_12g0014 | ribosome biogenesis regulatory protein [Nosema ceranae] |
| MSTRG.10794.1 | NBO_1303g0002 | hypothetical protein NBO_1303g0002 [Nosema bombycis CQ1] |
| MSTRG.5153.2 | NBO_1303g0002 | hypothetical protein NBO_1303g0002 [Nosema bombycis CQ1] |
| MSTRG.11899.2 | NBO_1314g0001 | hypothetical protein NBO_1314g0001 [Nosema bombycis CQ1] |
| MSTRG.2236.1 | NBO_1314g0001 | hypothetical protein NBO_1314g0001 [Nosema bombycis CQ1] |
| MSTRG.4271.1 | NBO_1314g0001 | hypothetical protein NBO_1314g0001 [Nosema bombycis CQ1] |
| MSTRG.10794.1 | NBO_13g0072 | hypothetical protein NBO_13g0072 [Nosema bombycis CQ1] |
| MSTRG.5153.2 | NBO_13g0072 | hypothetical protein NBO_13g0072 [Nosema bombycis CQ1] |
| MSTRG.2173.1 | NBO_1566g0002 | Deoxyhypusine hydroxylase [Nosema bombycis CQ1] |
| MSTRG.1777.1 | NBO_16g0040 | hypothetical protein NBO_16g0040 [Nosema bombycis CQ1] |
| MSTRG.10794.1 | NBO_204g0002 | hypothetical protein NBO_204g0002 [Nosema bombycis CQ1] |
| MSTRG.11875.2 | NBO_204g0002 | hypothetical protein NBO_204g0002 [Nosema bombycis CQ1] |
| MSTRG.5153.2 | NBO_204g0002 | hypothetical protein NBO_204g0002 [Nosema bombycis CQ1] |
| MSTRG.10794.1 | NBO_20g0001 | exocyst complex subunit sec6 [Nosema apis BRL 01] |
| MSTRG.11875.2 | NBO_20g0001 | exocyst complex subunit sec6 [Nosema apis BRL 01] |
| MSTRG.5153.2 | NBO_20g0001 | exocyst complex subunit sec6 [Nosema apis BRL 01] |
| MSTRG.3814.1 | NBO_22g0008 | ormdl family protein [Nosema ceranae] |
| MSTRG.10794.1 | NBO_238g0003 | hypothetical protein NBO_238g0003 [Nosema bombycis CQ1] |
| MSTRG.11875.2 | NBO_238g0003 | hypothetical protein NBO_238g0003 [Nosema bombycis CQ1] |
| MSTRG.5153.2 | NBO_238g0003 | hypothetical protein NBO_238g0003 [Nosema bombycis CQ1] |
| MSTRG.8004.2 | NBO_25g0006 | small nuclear ribonucleoprotein F [Nosema bombycis CQ1] |
| MSTRG.3814.1 | NBO_27g0006 | integral membrane [Nosema apis BRL 01] |
| MSTRG.4862.2 | NBO_27g0006 | integral membrane [Nosema apis BRL 01] |
| MSTRG.4271.1 | NBO_28g0061 | Cleavage and polyadenylation specificity factor subunit 4 [Nosema bombycis CQ1] |
| MSTRG.3814.1 | NBO_35g0002 | serpin-like protein [Nosema bombycis CQ1] |
| MSTRG.5419.1 | NBO_360g0003 | Deoxyuridine 5'-triphosphate nucleotidohydrolase [Nosema bombycis CQ1] |
| MSTRG.11899.2 | NBO_375g0009 | 2,3-bisphosphoglycerate-independent phosphoglycerate mutase [Nosema bombycis CQ1] |
| MSTRG.2236.1 | NBO_375g0009 | 2,3-bisphosphoglycerate-independent phosphoglycerate mutase [Nosema bombycis CQ1] |
| MSTRG.4271.1 | NBO_375g0009 | 2,3-bisphosphoglycerate-independent phosphoglycerate mutase [Nosema bombycis CQ1] |
| MSTRG.1988.2 | NBO_376g0002 | hypothetical protein NBO_376g0002 [Nosema bombycis CQ1] |
| MSTRG.8004.2 | NBO_376g0002 | hypothetical protein NBO_376g0002 [Nosema bombycis CQ1] |
| MSTRG.10054.3 | NBO_389g0002 | hypothetical protein NBO_389g0002, partial [Nosema bombycis CQ1] |
| MSTRG.8004.2 | NBO_38g0002 | hypothetical protein NBO_38g0002 [Nosema bombycis CQ1] |
| MSTRG.5153.2 | NBO_3g0033 | hypothetical protein NBO_3g0033 [Nosema bombycis CQ1] |
| MSTRG.3814.1 | NBO_401g0005 | YjeF family domain-containing protein [Anncaliia algerae PRA339] |
| MSTRG.3814.1 | NBO_417g0013 | ATP-binding protein [Ordospora colligata OC4] |
| MSTRG.2173.1 | NBO_423g0006 | Iron-sulfur clusters transporter atm1, mitochondrial, partial [Nosema bombycis CQ1] |
| MSTRG.10054.3 | NBO_452g0002 | Sld5 domain-containing protein [Rhizoctonia solani AG-1 IA] |
| MSTRG.1988.2 | NBO_46g0002 | hypothetical protein NBO_46g0002 [Nosema bombycis CQ1] |
| MSTRG.3814.1 | NBO_46g0002 | hypothetical protein NBO_46g0002 [Nosema bombycis CQ1] |
| MSTRG.3814.1 | NBO_481g0001 | hypothetical protein NBO_936g0001 [Nosema bombycis CQ1] |
| MSTRG.2013.1 | NBO_48g0004 | mevalonate kinase [Nosema bombycis CQ1] |
| MSTRG.585.1 | NBO_48g0004 | mevalonate kinase [Nosema bombycis CQ1] |
| MSTRG.10054.3 | NBO_49g0004 | Fructose-bisphosphate aldolase C [Nosema bombycis CQ1] |
| MSTRG.2173.1 | NBO_4g0048 | hypothetical protein NBO_4g0048 [Nosema bombycis CQ1] |
| MSTRG.1988.2 | NBO_508g0021 | Rac-like GTP-binding protein ARAC7 [Nosema bombycis CQ1] |
| MSTRG.3814.1 | NBO_508g0021 | Rac-like GTP-binding protein ARAC7 [Nosema bombycis CQ1] |
| MSTRG.11899.2 | NBO_54g0020 | Checkpoint protein kinase [Nosema bombycis CQ1] |
| MSTRG.2236.1 | NBO_54g0020 | Checkpoint protein kinase [Nosema bombycis CQ1] |
| MSTRG.3576.1 | NBO_552g0003 | Homeobox protein HD-4 [Nosema bombycis CQ1] |
| MSTRG.4862.2 | NBO_552g0003 | Homeobox protein HD-4 [Nosema bombycis CQ1] |
| MSTRG.8795.2 | NBO_552g0003 | Homeobox protein HD-4 [Nosema bombycis CQ1] |
| MSTRG.10794.1 | NBO_58g0026 | GPN-loop GTPase 1 [Nosema bombycis CQ1] |
| MSTRG.11875.2 | NBO_58g0026 | GPN-loop GTPase 1 [Nosema bombycis CQ1] |
| MSTRG.5153.2 | NBO_58g0026 | GPN-loop GTPase 1 [Nosema bombycis CQ1] |
| MSTRG.1777.1 | NBO_609g0001 | integral membrane [Nosema ceranae] |
| MSTRG.2173.1 | NBO_63g0025 | Serine/threonine-protein kinase ppk23 [Nosema bombycis CQ1] |
| MSTRG.3814.1 | NBO_64g0032 | YjeF family domain-containing protein [Anncaliia algerae PRA339] |
| MSTRG.1988.2 | NBO_675g0001 | hypothetical protein NBO_675g0001 [Nosema bombycis CQ1] |
| MSTRG.3814.1 | NBO_675g0001 | hypothetical protein NBO_675g0001 [Nosema bombycis CQ1] |
| MSTRG.3814.1 | NBO_6g0011 | hypothetical protein NBO_6g0011 [Nosema bombycis CQ1] |
| MSTRG.585.1 | NBO_6g0048 | Ricin B lectin [Nosema bombycis CQ1] |
| MSTRG.585.1 | NBO_6g0086 | hypothetical protein NBO_6g0086 [Nosema bombycis CQ1] |
| MSTRG.11899.2 | NBO_74g0004 | small-conductance mechanosensitive channel protein [Nosema ceranae] |
| MSTRG.2236.1 | NBO_74g0004 | small-conductance mechanosensitive channel protein [Nosema ceranae] |
| MSTRG.4271.1 | NBO_74g0004 | small-conductance mechanosensitive channel protein [Nosema ceranae] |
| MSTRG.2173.1 | NBO_78g0013 | hypothetical protein NBO_78g0013 [Nosema bombycis CQ1] |
| MSTRG.10054.3 | NBO_81g0012 | MADS domain containing protein, partial [Nosema bombycis CQ1] |
| MSTRG.6264.1 | NBO_8g0003 | hypothetical protein NBO_8g0003 [Nosema bombycis CQ1] |
| MSTRG.11899.2 | NBO_8g0045 | signal peptidase like protein [Nosema bombycis CQ1] |
| MSTRG.2236.1 | NBO_8g0045 | signal peptidase like protein [Nosema bombycis CQ1] |
| MSTRG.3814.1 | NBO_936g0001 | hypothetical protein NBO_936g0001 [Nosema bombycis CQ1] |

| **Table S5 *N.bombycis* miRNA-target genes analysis** | | |
| --- | --- | --- |
| miRNA | target | Description |
| novel-m0030-3p | NBO_114g0004 | Zinc phosphodiesterase ELAC protein 2 [Nosema bombycis CQ1] |
| novel-m0030-3p | NBO_13g0067 | f-box domain-containing protein [Nosema ceranae] |
| novel-m0030-3p | NBO_1530gi001 | tryptophanyl tRNA synthetase [Nosema bombycis CQ1] |
| novel-m0030-3p | NBO_481g0001 | hypothetical protein NBO_936g0001 [Nosema bombycis CQ1] |
| novel-m0030-3p | NBO_555g0001 | hypothetical protein NBO_555g0001 [Nosema bombycis CQ1] |
| novel-m0030-3p | NBO_597g0004 | hypothetical protein NBO_597g0004 [Nosema bombycis CQ1] |
| novel-m0030-3p | NBO_936g0001 | hypothetical protein NBO_936g0001 [Nosema bombycis CQ1] |
| novel-m0030-3p | NBO_940g0001 | RecName: Full=Spore wall protein 25; Flags: Precursor |
| novel-m0044-5p | NBO_153g0004 | zinc metalloprotease [Nosema ceranae] |
| novel-m0044-5p | NBO_241g0002 | Mitochondrial protein import protein mas5, partial [Nosema bombycis CQ1] |
| novel-m0044-5p | NBO_3g0006 | 60S ribosomal protein L6, partial [Nosema bombycis] |
| novel-m0044-5p | NBO_73g0026 | hypothetical protein NBO_73g0026 [Nosema bombycis CQ1] |
| novel-m0044-5p | NBO_740g0001 | ribosomal protein l12e-l44-l45-rpp1-rpp2 [Nosema apis BRL 01] |
| novel-m0044-5p | NBO_85g0011 | Solute carrier family 2, facilitated glucose transporter member 2 [Nosema bombycis CQ1] |
| novel-m0046-5p | NBO_153g0004 | zinc metalloprotease [Nosema ceranae] |
| novel-m0046-5p | NBO_241g0002 | Mitochondrial protein import protein mas5, partial [Nosema bombycis CQ1] |
| novel-m0046-5p | NBO_3g0006 | 60S ribosomal protein L6, partial [Nosema bombycis] |
| novel-m0046-5p | NBO_73g0026 | hypothetical protein NBO_73g0026 [Nosema bombycis CQ1] |
| novel-m0046-5p | NBO_740g0001 | ribosomal protein l12e-l44-l45-rpp1-rpp2 [Nosema apis BRL 01] |
| novel-m0046-5p | NBO_85g0011 | Solute carrier family 2, facilitated glucose transporter member 2 [Nosema bombycis CQ1] |
| novel-m0047-3p | NBO_1314g0001 | hypothetical protein NBO_1314g0001 [Nosema bombycis CQ1] |
| novel-m0047-3p | NBO_13g0072 | hypothetical protein NBO_13g0072 [Nosema bombycis CQ1] |
| novel-m0047-3p | NBO_1566g0001 | hemolysin-III-like protein [Encephalitozoon romaleae SJ-2008] |
| novel-m0047-3p | NBO_27g0020 | hypothetical protein NBO_27g0020 [Nosema bombycis CQ1] |
| novel-m0047-3p | NBO_42g0007 | rna polymerase ii ctd phosphatase [Nosema ceranae] |
| novel-m0047-3p | NBO_483g0001 | hypothetical protein NBO_483g0001 [Nosema bombycis CQ1] |
| novel-m0047-3p | NBO_6g0040 | hypothetical protein NBO_6g0040 [Nosema bombycis CQ1] |
| novel-m0047-3p | NBO_73g0012 | hypothetical protein NBO_73g0012 [Nosema bombycis CQ1] |
| novel-m0054-3p | NBO_1314g0001 | hypothetical protein NBO_1314g0001 [Nosema bombycis CQ1] |
| novel-m0054-3p | NBO_13g0072 | hypothetical protein NBO_13g0072 [Nosema bombycis CQ1] |
| novel-m0054-3p | NBO_1566g0001 | hemolysin-III-like protein [Encephalitozoon romaleae SJ-2008] |
| novel-m0054-3p | NBO_27g0020 | hypothetical protein NBO_27g0020 [Nosema bombycis CQ1] |
| novel-m0054-3p | NBO_42g0007 | rna polymerase ii ctd phosphatase [Nosema ceranae] |
| novel-m0054-3p | NBO_483g0001 | hypothetical protein NBO_483g0001 [Nosema bombycis CQ1] |
| novel-m0054-3p | NBO_6g0040 | hypothetical protein NBO_6g0040 [Nosema bombycis CQ1] |
| novel-m0054-3p | NBO_73g0012 | hypothetical protein NBO_73g0012 [Nosema bombycis CQ1] |
| novel-m0087-5p | NBO_1000gi001 | Protein PNS1, partial [Nosema bombycis CQ1] |
| novel-m0087-5p | NBO_1053g0001 | glucose transporter type 3 [Nosema bombycis CQ1] |
| novel-m0087-5p | NBO_10g0053 | DNA mismatch repair protein Mlh1 [Nosema bombycis CQ1] |
| novel-m0087-5p | NBO_1131g0001 | hypothetical protein NBO_1131g0001 [Nosema bombycis CQ1] |
| novel-m0087-5p | NBO_1133g0001 | Ricin B lectin [Nosema bombycis CQ1] |
| novel-m0087-5p | NBO_1135g0001 | ricin b lectin [Nosema ceranae] |
| novel-m0087-5p | NBO_114g0004 | Zinc phosphodiesterase ELAC protein 2 [Nosema bombycis CQ1] |
| novel-m0087-5p | NBO_11g0003 | UTP-glucose-1-phosphate uridylyltransferase [Nosema bombycis CQ1] |
| novel-m0087-5p | NBO_11g0038 | Protein kinase kin1 [Nosema bombycis CQ1] |
| novel-m0087-5p | NBO_11g0041 | small-conductance mechanosensitive channel protein [Nosema ceranae] |
| novel-m0087-5p | NBO_1271g0002 | hypothetical protein NBO_1271g0002 [Nosema bombycis CQ1] |
| novel-m0087-5p | NBO_12g0008 | hypothetical protein NBO_12g0008 [Nosema bombycis CQ1] |
| novel-m0087-5p | NBO_12g0010 | pseudouridylate synthase [Nosema ceranae] |
| novel-m0087-5p | NBO_1302g0001 | hypothetical protein NBO_1302g0001 [Nosema bombycis CQ1] |
| novel-m0087-5p | NBO_1302g0002 | Superkiller viralicidic activity 2-like 2 [Nosema bombycis CQ1] |
| novel-m0087-5p | NBO_1332gi002 | threonyl-tRNA synthetase [Nosema bombycis CQ1] |
| novel-m0087-5p | NBO_13g0005 | longevity assurance protein 1 [Nosema bombycis CQ1] |
| novel-m0087-5p | NBO_13g0057 | transcription factor TAU-like protein [Nosema bombycis CQ1] |
| novel-m0087-5p | NBO_166g0001 | Polysaccharide deacetylase [Nosema bombycis CQ1] |
| novel-m0087-5p | NBO_16g0013 | Structural maintenance of chromosomes protein 4, partial [Nosema bombycis CQ1] |
| novel-m0087-5p | NBO_19g0014 | hypothetical protein NBO_19g0014 [Nosema bombycis CQ1] |
| novel-m0087-5p | NBO_204g0002 | hypothetical protein NBO_204g0002 [Nosema bombycis CQ1] |
| novel-m0087-5p | NBO_20g0001 | exocyst complex subunit sec6 [Nosema apis BRL 01] |
| novel-m0087-5p | NBO_20g0008 | peptide chain release factor 2 [Nosema ceranae] |
| novel-m0087-5p | NBO_27g0061 | hypothetical protein NBO_27g0061 [Nosema bombycis CQ1] |
| novel-m0087-5p | NBO_28g0061 | Cleavage and polyadenylation specificity factor subunit 4 [Nosema bombycis CQ1] |
| novel-m0087-5p | NBO_29g0020 | Tristetraproline [Nosema bombycis CQ1] |
| novel-m0087-5p | NBO_29g0027 | trehalose-phosphatase, partial [Nosema bombycis CQ1] |
| novel-m0087-5p | NBO_29g0031 | Solute carrier family 2, facilitated glucose transporter member 3 [Nosema bombycis CQ1] |
| novel-m0087-5p | NBO_2g0074 | Nuclear elongation and deformation protein 1 [Nosema bombycis CQ1] |
| novel-m0087-5p | NBO_32gi001 | threonyl-tRNA synthetase [Nosema bombycis CQ1] |
| novel-m0087-5p | NBO_338g0001 | hypothetical protein NBO_338g0001 [Nosema bombycis CQ1] |
| novel-m0087-5p | NBO_33gi001 | Protein PNS1, partial [Nosema bombycis CQ1] |
| novel-m0087-5p | NBO_350g0001 | Protein PNS1, partial [Nosema bombycis CQ1] |
| novel-m0087-5p | NBO_362g0010 | hypothetical protein NBO_362g0010 [Nosema bombycis CQ1] |
| novel-m0087-5p | NBO_364g0016 | hypothetical protein NBO_364g0016 [Nosema bombycis CQ1] |
| novel-m0087-5p | NBO_377g0003 | Heat shock 70 kDa protein 6 [Nosema bombycis CQ1] |
| novel-m0087-5p | NBO_37g0005 | Cystine/glutamate transporter [Nosema bombycis CQ1] |
| novel-m0087-5p | NBO_384g0005 | hypothetical protein NBO_384g0005 [Nosema bombycis CQ1] |
| novel-m0087-5p | NBO_396g0002 | TAF4 transcription initiation factor TFIID component [Encephalitozoon intestinalis ATCC 50506] |
| novel-m0087-5p | NBO_411gi001 | GTP-binding protein [Nosema bombycis CQ1] |
| novel-m0087-5p | NBO_417g0011 | Solute carrier family 35 member C2, partial [Nosema bombycis CQ1] |
| novel-m0087-5p | NBO_417g0013 | ATP-binding protein [Ordospora colligata OC4] |
| novel-m0087-5p | NBO_417g0015 | ADP-ribosylation factor 1 [Nosema bombycis CQ1] |
| novel-m0087-5p | NBO_418gi001 | integral membrane protein [Nosema bombycis CQ1] |
| novel-m0087-5p | NBO_41g0030 | hypothetical protein NBO_41g0030 [Nosema bombycis CQ1] |
| novel-m0087-5p | NBO_429g0001 | hypothetical protein NBO_429g0001 [Nosema bombycis CQ1] |
| novel-m0087-5p | NBO_44g0011 | Cell division control protein 50, partial [Nosema bombycis CQ1] |
| novel-m0087-5p | NBO_4g0031 | elongation factor 2 [Nosema bombycis CQ1] |
| novel-m0087-5p | NBO_508g0035 | WD repeat-containing protein, partial [Nosema bombycis CQ1] |
| novel-m0087-5p | NBO_53g0005 | Polysaccharide deacetylase [Nosema bombycis CQ1] |
| novel-m0087-5p | NBO_53g0017 | glucose-6-phosphate isomerase [Nosema bombycis CQ1] |
| novel-m0087-5p | NBO_549g0007 | DNA polymerase epsilon catalytic subunit A [Nosema bombycis CQ1] |
| novel-m0087-5p | NBO_555g0001 | hypothetical protein NBO_555g0001 [Nosema bombycis CQ1] |
| novel-m0087-5p | NBO_568g0001 | Tristetraproline [Nosema bombycis CQ1] |
| novel-m0087-5p | NBO_60g0017 | Transcription-associated protein 1 [Nosema bombycis CQ1] |
| novel-m0087-5p | NBO_60g0018 | Polysaccharide deacetylase [Nosema bombycis CQ1] |
| novel-m0087-5p | NBO_62g0012 | hypothetical protein NBO_62g0012 [Nosema bombycis CQ1] |
| novel-m0087-5p | NBO_64g0035 | transcription elongation factor s-ii [Nosema ceranae] |
| novel-m0087-5p | NBO_69g0004 | DNA polymerase alpha subunit B, partial [Nosema bombycis CQ1] |
| novel-m0087-5p | NBO_6g0028 | hypothetical protein NBO_6g0028 [Nosema bombycis CQ1] |
| novel-m0087-5p | NBO_6g0037 | Meiotic nuclear division protein 1 [Nosema bombycis CQ1] |
| novel-m0087-5p | NBO_6g0058 | hypothetical protein NBO_6g0058, partial [Nosema bombycis CQ1] |
| novel-m0087-5p | NBO_6g0062 | Ricin B lectin [Nosema bombycis CQ1] |
| novel-m0087-5p | NBO_73g0017 | 26S protease regulatory subunit 4, partial [Nosema bombycis CQ1] |
| novel-m0087-5p | NBO_73g0026 | hypothetical protein NBO_73g0026 [Nosema bombycis CQ1] |
| novel-m0087-5p | NBO_753g0001 | leptin receptor gene-related protein [Nosema bombycis CQ1] |
| novel-m0087-5p | NBO_757g0002 | Beta tubulin, autoregulation binding site [Nosema bombycis CQ1] |
| novel-m0087-5p | NBO_76g0003 | hypothetical protein NBO_76g0003 [Nosema bombycis CQ1] |
| novel-m0087-5p | NBO_76g0012 | type 2 peptidyl-tRNA hydrolase [Encephalitozoon romaleae SJ-2008] |
| novel-m0087-5p | NBO_80g0024 | Major facilitator superfamily domain-containing protein 1 [Nosema bombycis CQ1] |
| novel-m0087-5p | NBO_845g0001 | hypothetical protein NBO_845g0001 [Nosema bombycis CQ1] |
| novel-m0087-5p | NBO_974g0001 | Polysaccharide deacetylase [Nosema bombycis CQ1] |
| novel-m0087-5p | NBO_979g0001 | transcription initiation factor brf1 subunit-like protein [Nosema ceranae] |
| novel-m0105-5p | NBO_11g0004 | hypothetical protein NBO_11g0004 [Nosema bombycis CQ1] |
| novel-m0105-5p | NBO_20g0008 | peptide chain release factor 2 [Nosema ceranae] |
| novel-m0105-5p | NBO_362g0011 | CDP-diacylglycerol--inositol 3-phosphatidyltransferase [Nosema bombycis CQ1] |
| novel-m0105-5p | NBO_375g0010 | 2,3-bisphosphoglycerate-independent phosphoglycerate mutase [Nosema bombycis CQ1] |
| novel-m0105-5p | NBO_481g0001 | hypothetical protein NBO_936g0001 [Nosema bombycis CQ1] |
| novel-m0105-5p | NBO_4g0042 | TBC1 domain family member 13 [Nosema bombycis CQ1] |
| novel-m0105-5p | NBO_936g0001 | hypothetical protein NBO_936g0001 [Nosema bombycis CQ1] |
| novel-m0153-3p | NBO_1314g0001 | hypothetical protein NBO_1314g0001 [Nosema bombycis CQ1] |
| novel-m0153-3p | NBO_13g0072 | hypothetical protein NBO_13g0072 [Nosema bombycis CQ1] |
| novel-m0153-3p | NBO_1566g0001 | hemolysin-III-like protein [Encephalitozoon romaleae SJ-2008] |
| novel-m0153-3p | NBO_27g0020 | hypothetical protein NBO_27g0020 [Nosema bombycis CQ1] |
| novel-m0153-3p | NBO_42g0007 | rna polymerase ii ctd phosphatase [Nosema ceranae] |
| novel-m0153-3p | NBO_483g0001 | hypothetical protein NBO_483g0001 [Nosema bombycis CQ1] |
| novel-m0153-3p | NBO_6g0040 | hypothetical protein NBO_6g0040 [Nosema bombycis CQ1] |
| novel-m0153-3p | NBO_73g0012 | hypothetical protein NBO_73g0012 [Nosema bombycis CQ1] |
| novel-m0023-5p | NBO_1116g0003 | hypothetical protein NBO_1116g0003 [Nosema bombycis CQ1] |
| novel-m0023-5p | NBO_1170gi001 | HSP 101 related protein, partial [Nosema bombycis CQ1] |
| novel-m0023-5p | NBO_16g0024 | hypothetical protein NBO_16g0024 [Nosema bombycis CQ1] |
| novel-m0023-5p | NBO_16g0028 | hypothetical protein NBO_16g0028 [Nosema bombycis CQ1] |
| novel-m0023-5p | NBO_16g0040 | hypothetical protein NBO_16g0040 [Nosema bombycis CQ1] |
| novel-m0023-5p | NBO_20g0001 | exocyst complex subunit sec6 [Nosema apis BRL 01] |
| novel-m0023-5p | NBO_27g0027 | ribosomal protein hs6-type [Nosema apis BRL 01] |
| novel-m0023-5p | NBO_28g0053 | Forkhead box protein K2 [Nosema bombycis CQ1] |
| novel-m0023-5p | NBO_33g0018 | 78 kDa glucose-regulated protein [Nosema bombycis CQ1] |
| novel-m0023-5p | NBO_375g0009 | 2,3-bisphosphoglycerate-independent phosphoglycerate mutase [Nosema bombycis CQ1] |
| novel-m0023-5p | NBO_4g0017 | Heat shock protein 101 [Nosema bombycis CQ1] |
| novel-m0023-5p | NBO_4g0050 | Insulin-degrading enzyme [Nosema bombycis CQ1] |
| novel-m0023-5p | NBO_520g0003 | N(2),N(2)-dimethylguanosine tRNA methyltransferase [Nosema bombycis CQ1] |
| novel-m0023-5p | NBO_55g0005 | mitochondrial pyruvate dehydrogenase E1 component subunit alpha [Nosema bombycis] |
| novel-m0023-5p | NBO_606g0001 | hypothetical protein NBO_606g0001 [Nosema bombycis CQ1] |
| novel-m0023-5p | NBO_63g0026 | hypothetical spore wall protein [Nosema bombycis] |
| novel-m0023-5p | NBO_71g0002 | isoleucyl-tRNA synthetase, cytoplasmic [Nosema bombycis CQ1] |
| novel-m0023-5p | NBO_7g0006 | hypothetical protein NBO_7g0006 [Nosema bombycis CQ1] |
| novel-m0023-5p | NBO_81g0012 | MADS domain containing protein, partial [Nosema bombycis CQ1] |
| novel-m0028-5p | NBO_376g0001 | hypothetical protein NBO_376g0001 [Nosema bombycis CQ1] |
| novel-m0028-5p | NBO_76g0022 | hypothetical protein NBO_76g0022 [Nosema bombycis CQ1] |
| novel-m0040-5p | NBO_76g0016 | hypothetical protein NBO_76g0016 [Nosema bombycis CQ1] |
| novel-m0043-3p | NBO_11g0003 | UTP-glucose-1-phosphate uridylyltransferase [Nosema bombycis CQ1] |
| novel-m0043-3p | NBO_1553gi001 | sugar permease, partial [Nosema bombycis CQ1] |
| novel-m0043-3p | NBO_18g0028 | sugar permease [Nosema bombycis CQ1] |
| novel-m0043-3p | NBO_58g0010 | Chitin synthase 7 [Nosema bombycis CQ1] |
| novel-m0061-5p | NBO_1053g0001 | glucose transporter type 3 [Nosema bombycis CQ1] |
| novel-m0061-5p | NBO_1136g0001 | ricin b lectin [Nosema ceranae] |
| novel-m0061-5p | NBO_444g0008 | Cysteinyl-tRNA synthetase, cytoplasmic [Nosema bombycis CQ1] |
| novel-m0066-3p | NBO_377g0003 | Heat shock 70 kDa protein 6 [Nosema bombycis CQ1] |
| novel-m0074-5p | NBO_1024g0001 | DNA-directed RNA polymerase II subunit RPB3 [Nosema bombycis CQ1] |
| novel-m0074-5p | NBO_10g0001 | dna-directed rna polymerase ii subunit rpb7 [Nosema ceranae] |
| novel-m0074-5p | NBO_10g0055 | hypothetical protein NBO_10g0055, partial [Nosema bombycis CQ1] |
| novel-m0074-5p | NBO_11g0064 | Guanine nucleotide-binding protein-like 3 [Nosema bombycis CQ1] |
| novel-m0074-5p | NBO_1203g0001 | hypothetical protein NBO_1203g0001 [Nosema bombycis CQ1] |
| novel-m0074-5p | NBO_1276g0001 | tRNA acetyltransferase TAN1 [Nematocida displodere] |
| novel-m0074-5p | NBO_1302g0001 | hypothetical protein NBO_1302g0001 [Nosema bombycis CQ1] |
| novel-m0074-5p | NBO_13g0067 | f-box domain-containing protein [Nosema ceranae] |
| novel-m0074-5p | NBO_1566g0001 | hemolysin-III-like protein [Encephalitozoon romaleae SJ-2008] |
| novel-m0074-5p | NBO_15g0003 | GPI mannosyltransferase 2 [Nosema bombycis CQ1] |
| novel-m0074-5p | NBO_166g0001 | Polysaccharide deacetylase [Nosema bombycis CQ1] |
| novel-m0074-5p | NBO_19g0014 | hypothetical protein NBO_19g0014 [Nosema bombycis CQ1] |
| novel-m0074-5p | NBO_19g0026 | U3 small nucleolar ribonucleoprotein imp4 [Nosema bombycis CQ1] |
| novel-m0074-5p | NBO_20g0001 | exocyst complex subunit sec6 [Nosema apis BRL 01] |
| novel-m0074-5p | NBO_241g0002 | Mitochondrial protein import protein mas5, partial [Nosema bombycis CQ1] |
| novel-m0074-5p | NBO_24g0004 | myosin heavy chain [Nosema ceranae] |
| novel-m0074-5p | NBO_27g0025 | skt5-like protein [Nosema apis BRL 01] |
| novel-m0074-5p | NBO_29g0020 | Tristetraproline [Nosema bombycis CQ1] |
| novel-m0074-5p | NBO_2g0074 | Nuclear elongation and deformation protein 1 [Nosema bombycis CQ1] |
| novel-m0074-5p | NBO_2gi003 | DNA-directed RNA polymerases I and III subunit RPAC2 [Nosema bombycis CQ1] |
| novel-m0074-5p | NBO_32g0030 | hypothetical protein NBO_32g0030 [Nosema bombycis CQ1] |
| novel-m0074-5p | NBO_32g0049 | Glycolipid 2-alpha-mannosyltransferase [Nosema bombycis CQ1] |
| novel-m0074-5p | NBO_32gi001 | threonyl-tRNA synthetase [Nosema bombycis CQ1] |
| novel-m0074-5p | NBO_377g0003 | Heat shock 70 kDa protein 6 [Nosema bombycis CQ1] |
| novel-m0074-5p | NBO_386g0004 | Meiotic expression up-regulated protein 26 [Nosema bombycis CQ1] |
| novel-m0074-5p | NBO_389g0002 | hypothetical protein NBO_389g0002, partial [Nosema bombycis CQ1] |
| novel-m0074-5p | NBO_3g0028 | vacuolar atp synthase subunit d [Nosema ceranae] |
| novel-m0074-5p | NBO_401g0008 | transcription elongation factor s-ii [Nosema ceranae] |
| novel-m0074-5p | NBO_481g0001 | hypothetical protein NBO_936g0001 [Nosema bombycis CQ1] |
| novel-m0074-5p | NBO_48g0004 | mevalonate kinase [Nosema bombycis CQ1] |
| novel-m0074-5p | NBO_568g0001 | Tristetraproline [Nosema bombycis CQ1] |
| novel-m0074-5p | NBO_582g0002 | Tristetraproline [Nosema bombycis CQ1] |
| novel-m0074-5p | NBO_597g0004 | hypothetical protein NBO_597g0004 [Nosema bombycis CQ1] |
| novel-m0074-5p | NBO_60g0018 | Polysaccharide deacetylase [Nosema bombycis CQ1] |
| novel-m0074-5p | NBO_64g0035 | transcription elongation factor s-ii [Nosema ceranae] |
| novel-m0074-5p | NBO_6g0076 | dna-directed rna polymerase ii subunit rpb7 [Nosema ceranae] |
| novel-m0074-5p | NBO_76gi004 | threonyl-tRNA synthetase [Nosema bombycis CQ1] |
| novel-m0074-5p | NBO_770g0002 | Ubiquitin thioesterase otubain-like protein [Nosema bombycis CQ1] |
| novel-m0074-5p | NBO_84g0004 | Glycerol-3-phosphate dehydrogenase, mitochondrial [Nosema bombycis CQ1] |
| novel-m0074-5p | NBO_87g0001 | Homeobox protein HD-8 [Nosema bombycis CQ1] |
| novel-m0074-5p | NBO_929g0005 | hypothetical protein NBO_929g0005 [Nosema bombycis CQ1] |
| novel-m0074-5p | NBO_936g0001 | hypothetical protein NBO_936g0001 [Nosema bombycis CQ1] |
| novel-m0077-5p | NBO_12g0008 | hypothetical protein NBO_12g0008 [Nosema bombycis CQ1] |
| novel-m0077-5p | NBO_28g0019 | DNA-directed RNA polymerases I and III subunit RPAC1 [Nosema bombycis CQ1] |
| novel-m0077-5p | NBO_32g0022 | Glutaredoxin [Nosema bombycis CQ1] |
| novel-m0077-5p | NBO_37g0005 | Cystine/glutamate transporter [Nosema bombycis CQ1] |
| novel-m0077-5p | NBO_3g0004 | hypothetical protein NBO_3g0004 [Nosema bombycis CQ1] |
| novel-m0077-5p | NBO_41g0047 | thioltransferase [Nosema bombycis CQ1] |
| novel-m0077-5p | NBO_48g0004 | mevalonate kinase [Nosema bombycis CQ1] |
| novel-m0077-5p | NBO_4g0009 | 3-ketodihydrosphingosine reductase [Nosema bombycis CQ1] |
| novel-m0077-5p | NBO_508g0035 | WD repeat-containing protein, partial [Nosema bombycis CQ1] |
| novel-m0077-5p | NBO_53g0022 | DRAP deaminase [Nosema bombycis CQ1] |
| novel-m0077-5p | NBO_547g0003 | hypothetical protein NBO_547g0003 [Nosema bombycis CQ1] |
| novel-m0077-5p | NBO_591g0001 | Transketolase 1, partial [Nosema bombycis CQ1] |
| novel-m0077-5p | NBO_63g0015 | hypothetical protein NBO_63g0015, partial [Nosema bombycis CQ1] |
| novel-m0077-5p | NBO_63g0018 | hypothetical protein NBO_63g0018 [Nosema bombycis CQ1] |
| novel-m0077-5p | NBO_63g0020 | Glutamate synthase NADPH small chain [Nosema bombycis CQ1] |
| novel-m0077-5p | NBO_740g0001 | ribosomal protein l12e-l44-l45-rpp1-rpp2 [Nosema apis BRL 01] |
| novel-m0077-5p | NBO_74g0004 | small-conductance mechanosensitive channel protein [Nosema ceranae] |
| novel-m0077-5p | NBO_911gi001 | XPF/ERCC4/RAD1 family like protein, partial [Nosema bombycis CQ1] |
| novel-m0089-5p | NBO_376g0001 | hypothetical protein NBO_376g0001 [Nosema bombycis CQ1] |
| novel-m0089-5p | NBO_76g0022 | hypothetical protein NBO_76g0022 [Nosema bombycis CQ1] |
| novel-m0101-5p | NBO_376g0001 | hypothetical protein NBO_376g0001 [Nosema bombycis CQ1] |
| novel-m0101-5p | NBO_76g0022 | hypothetical protein NBO_76g0022 [Nosema bombycis CQ1] |
| novel-m0106-3p | NBO_377g0003 | Heat shock 70 kDa protein 6 [Nosema bombycis CQ1] |
| novel-m0110-5p | NBO_1116g0003 | hypothetical protein NBO_1116g0003 [Nosema bombycis CQ1] |
| novel-m0110-5p | NBO_1170gi001 | HSP 101 related protein, partial [Nosema bombycis CQ1] |
| novel-m0110-5p | NBO_16g0024 | hypothetical protein NBO_16g0024 [Nosema bombycis CQ1] |
| novel-m0110-5p | NBO_16g0028 | hypothetical protein NBO_16g0028 [Nosema bombycis CQ1] |
| novel-m0110-5p | NBO_16g0040 | hypothetical protein NBO_16g0040 [Nosema bombycis CQ1] |
| novel-m0110-5p | NBO_20g0001 | exocyst complex subunit sec6 [Nosema apis BRL 01] |
| novel-m0110-5p | NBO_27g0027 | ribosomal protein hs6-type [Nosema apis BRL 01] |
| novel-m0110-5p | NBO_28g0053 | Forkhead box protein K2 [Nosema bombycis CQ1] |
| novel-m0110-5p | NBO_33g0018 | 78 kDa glucose-regulated protein [Nosema bombycis CQ1] |
| novel-m0110-5p | NBO_375g0009 | 2,3-bisphosphoglycerate-independent phosphoglycerate mutase [Nosema bombycis CQ1] |
| novel-m0110-5p | NBO_4g0017 | Heat shock protein 101 [Nosema bombycis CQ1] |
| novel-m0110-5p | NBO_4g0050 | Insulin-degrading enzyme [Nosema bombycis CQ1] |
| novel-m0110-5p | NBO_520g0003 | N(2),N(2)-dimethylguanosine tRNA methyltransferase [Nosema bombycis CQ1] |
| novel-m0110-5p | NBO_55g0005 | mitochondrial pyruvate dehydrogenase E1 component subunit alpha [Nosema bombycis] |
| novel-m0110-5p | NBO_606g0001 | hypothetical protein NBO_606g0001 [Nosema bombycis CQ1] |
| novel-m0110-5p | NBO_63g0026 | hypothetical spore wall protein [Nosema bombycis] |
| novel-m0110-5p | NBO_71g0002 | isoleucyl-tRNA synthetase, cytoplasmic [Nosema bombycis CQ1] |
| novel-m0110-5p | NBO_7g0006 | hypothetical protein NBO_7g0006 [Nosema bombycis CQ1] |
| novel-m0110-5p | NBO_81g0012 | MADS domain containing protein, partial [Nosema bombycis CQ1] |
| novel-m0163-5p | NBO_241g0002 | Mitochondrial protein import protein mas5, partial [Nosema bombycis CQ1] |
| novel-m0163-5p | NBO_4g0050 | Insulin-degrading enzyme [Nosema bombycis CQ1] |
| novel-m0175-5p | NBO_376g0001 | hypothetical protein NBO_376g0001 [Nosema bombycis CQ1] |
| novel-m0175-5p | NBO_76g0022 | hypothetical protein NBO_76g0022 [Nosema bombycis CQ1] |
| novel-m0177-3p | NBO_1057g0002 | histone h3 [Nosema apis BRL 01] |
| novel-m0177-3p | NBO_1110g0002 | Protein transport protein sec31 [Nosema bombycis CQ1] |
| novel-m0177-3p | NBO_11g0003 | UTP-glucose-1-phosphate uridylyltransferase [Nosema bombycis CQ1] |
| novel-m0177-3p | NBO_1608g0001 | Isoleucyl-tRNA synthetase, cytoplasmic [Nosema bombycis CQ1] |
| novel-m0177-3p | NBO_28g0019 | DNA-directed RNA polymerases I and III subunit RPAC1 [Nosema bombycis CQ1] |
| novel-m0177-3p | NBO_29g0015 | Phosphoacetylglucosamine mutase [Nosema bombycis CQ1] |
| novel-m0177-3p | NBO_55g0017 | integral membrane protein [Nosema bombycis CQ1] |
| novel-m0177-3p | NBO_58g0010 | Chitin synthase 7 [Nosema bombycis CQ1] |
| novel-m0177-3p | NBO_58g0026 | GPN-loop GTPase 1 [Nosema bombycis CQ1] |
| novel-m0177-3p | NBO_67g0015 | hypothetical protein NBO_67g0015 [Nosema bombycis CQ1] |
| novel-m0177-3p | NBO_6g0049 | Ricin B lectin [Nosema bombycis CQ1] |
| novel-m0177-3p | NBO_70g0007 | hypothetical protein NBO_70g0007 [Nosema bombycis CQ1] |
| novel-m0177-3p | NBO_71g0002 | isoleucyl-tRNA synthetase, cytoplasmic [Nosema bombycis CQ1] |
| novel-m0177-3p | NBO_73g0023 | hypothetical protein NBO_73g0023 [Nosema bombycis CQ1] |
| novel-m0177-3p | NBO_73g0026 | hypothetical protein NBO_73g0026 [Nosema bombycis CQ1] |
| novel-m0177-3p | NBO_740g0001 | ribosomal protein l12e-l44-l45-rpp1-rpp2 [Nosema apis BRL 01] |
| novel-m0177-3p | NBO_74g0004 | small-conductance mechanosensitive channel protein [Nosema ceranae] |
| novel-m0177-3p | NBO_8g0003 | hypothetical protein NBO_8g0003 [Nosema bombycis CQ1] |
| novel-m0184-3p | NBO_1015g0001 | U3 small nucleolar RNA-associated protein 15 [Nosema bombycis CQ1] |
| novel-m0184-3p | NBO_1053g0001 | glucose transporter type 3 [Nosema bombycis CQ1] |
| novel-m0184-3p | NBO_1086g0001 | hypothetical protein NBO_1086g0001 [Nosema bombycis CQ1] |
| novel-m0184-3p | NBO_10g0023 | hypothetical protein NBO_10g0023 [Nosema bombycis CQ1] |
| novel-m0184-3p | NBO_10g0034 | hypothetical protein NBO_10g0034 [Nosema bombycis CQ1] |
| novel-m0184-3p | NBO_10g0053 | DNA mismatch repair protein Mlh1 [Nosema bombycis CQ1] |
| novel-m0184-3p | NBO_1168gi001 | SCF ubiquitin ligase and anaphase-promoting complex protein [Encephalitozoon intestinalis ATCC 50506] |
| novel-m0184-3p | NBO_11g0003 | UTP-glucose-1-phosphate uridylyltransferase [Nosema bombycis CQ1] |
| novel-m0184-3p | NBO_1203g0001 | hypothetical protein NBO_1203g0001 [Nosema bombycis CQ1] |
| novel-m0184-3p | NBO_1344g0003 | magnesium transporter alr2 [Nosema ceranae] |
| novel-m0184-3p | NBO_13g0057 | transcription factor TAU-like protein [Nosema bombycis CQ1] |
| novel-m0184-3p | NBO_18g0007 | hypothetical protein NBO_18g0007 [Nosema bombycis CQ1] |
| novel-m0184-3p | NBO_20g0008 | peptide chain release factor 2 [Nosema ceranae] |
| novel-m0184-3p | NBO_29g0031 | Solute carrier family 2, facilitated glucose transporter member 3 [Nosema bombycis CQ1] |
| novel-m0184-3p | NBO_32g0034 | DNA polymerase kappa [Nosema bombycis CQ1] |
| novel-m0184-3p | NBO_33g0017 | hypothetical protein NBO_33g0017 [Nosema bombycis CQ1] |
| novel-m0184-3p | NBO_362g0011 | CDP-diacylglycerol--inositol 3-phosphatidyltransferase [Nosema bombycis CQ1] |
| novel-m0184-3p | NBO_366g0002 | Heat shock protein HSP 90-alpha 1 [Nosema bombycis CQ1] |
| novel-m0184-3p | NBO_377g0003 | Heat shock 70 kDa protein 6 [Nosema bombycis CQ1] |
| novel-m0184-3p | NBO_389g0002 | hypothetical protein NBO_389g0002, partial [Nosema bombycis CQ1] |
| novel-m0184-3p | NBO_38g0002 | hypothetical protein NBO_38g0002 [Nosema bombycis CQ1] |
| novel-m0184-3p | NBO_41g0030 | hypothetical protein NBO_41g0030 [Nosema bombycis CQ1] |
| novel-m0184-3p | NBO_41g0047 | thioltransferase [Nosema bombycis CQ1] |
| novel-m0184-3p | NBO_423g0003 | hypothetical protein NBO_423g0003 [Nosema bombycis CQ1] |
| novel-m0184-3p | NBO_452g0003 | Peptidyl-prolyl cis-trans isomerase NIMA-interacting 1 [Nosema bombycis CQ1] |
| novel-m0184-3p | NBO_468g0005 | Heat shock protein 90 [Nosema bombycis CQ1] |
| novel-m0184-3p | NBO_490g0001 | hypothetical protein NBO_490g0001 [Nosema bombycis CQ1] |
| novel-m0184-3p | NBO_53g0003 | hypothetical protein NBO_53g0003 [Nosema bombycis CQ1] |
| novel-m0184-3p | NBO_53g0017 | glucose-6-phosphate isomerase [Nosema bombycis CQ1] |
| novel-m0184-3p | NBO_547g0003 | hypothetical protein NBO_547g0003 [Nosema bombycis CQ1] |
| novel-m0184-3p | NBO_549g0007 | DNA polymerase epsilon catalytic subunit A [Nosema bombycis CQ1] |
| novel-m0184-3p | NBO_552g0005 | surface-antigen protein P30.4, partial [Nosema bombycis] |
| novel-m0184-3p | NBO_560g0001 | DNA-binding protein SMUBP-2 [Nosema bombycis CQ1] |
| novel-m0184-3p | NBO_591g0001 | Transketolase 1, partial [Nosema bombycis CQ1] |
| novel-m0184-3p | NBO_616g0003 | Heat shock factor protein 1 [Nosema bombycis CQ1] |
| novel-m0184-3p | NBO_63g0015 | hypothetical protein NBO_63g0015, partial [Nosema bombycis CQ1] |
| novel-m0184-3p | NBO_6g0088 | hypothetical protein NBO_6g0088 [Nosema bombycis CQ1] |
| novel-m0184-3p | NBO_707g0002 | Cyclin-dependent kinase C-2 [Nosema bombycis CQ1] |
| novel-m0184-3p | NBO_70g0007 | hypothetical protein NBO_70g0007 [Nosema bombycis CQ1] |
| novel-m0184-3p | NBO_73g0016 | hypothetical protein NBO_73g0016 [Nosema bombycis CQ1] |
| novel-m0184-3p | NBO_76g0003 | hypothetical protein NBO_76g0003 [Nosema bombycis CQ1] |
| novel-m0184-3p | NBO_7g0005 | hypothetical protein NBO_7g0005 [Nosema bombycis CQ1] |
| novel-m0184-3p | NBO_85g0001 | Ubiquitin-like modifier-activating enzyme 1 Y, partial [Nosema bombycis CQ1] |
| novel-m0189-3p | NBO_1015g0001 | U3 small nucleolar RNA-associated protein 15 [Nosema bombycis CQ1] |
| novel-m0189-3p | NBO_1053g0001 | glucose transporter type 3 [Nosema bombycis CQ1] |
| novel-m0189-3p | NBO_1086g0001 | hypothetical protein NBO_1086g0001 [Nosema bombycis CQ1] |
| novel-m0189-3p | NBO_10g0023 | hypothetical protein NBO_10g0023 [Nosema bombycis CQ1] |
| novel-m0189-3p | NBO_10g0034 | hypothetical protein NBO_10g0034 [Nosema bombycis CQ1] |
| novel-m0189-3p | NBO_10g0053 | DNA mismatch repair protein Mlh1 [Nosema bombycis CQ1] |
| novel-m0189-3p | NBO_1168gi001 | SCF ubiquitin ligase and anaphase-promoting complex protein [Encephalitozoon intestinalis ATCC 50506] |
| novel-m0189-3p | NBO_11g0003 | UTP-glucose-1-phosphate uridylyltransferase [Nosema bombycis CQ1] |
| novel-m0189-3p | NBO_1203g0001 | hypothetical protein NBO_1203g0001 [Nosema bombycis CQ1] |
| novel-m0189-3p | NBO_1344g0003 | magnesium transporter alr2 [Nosema ceranae] |
| novel-m0189-3p | NBO_13g0057 | transcription factor TAU-like protein [Nosema bombycis CQ1] |
| novel-m0189-3p | NBO_18g0007 | hypothetical protein NBO_18g0007 [Nosema bombycis CQ1] |
| novel-m0189-3p | NBO_20g0008 | peptide chain release factor 2 [Nosema ceranae] |
| novel-m0189-3p | NBO_29g0031 | Solute carrier family 2, facilitated glucose transporter member 3 [Nosema bombycis CQ1] |
| novel-m0189-3p | NBO_32g0034 | DNA polymerase kappa [Nosema bombycis CQ1] |
| novel-m0189-3p | NBO_33g0017 | hypothetical protein NBO_33g0017 [Nosema bombycis CQ1] |
| novel-m0189-3p | NBO_362g0011 | CDP-diacylglycerol--inositol 3-phosphatidyltransferase [Nosema bombycis CQ1] |
| novel-m0189-3p | NBO_366g0002 | Heat shock protein HSP 90-alpha 1 [Nosema bombycis CQ1] |
| novel-m0189-3p | NBO_377g0003 | Heat shock 70 kDa protein 6 [Nosema bombycis CQ1] |
| novel-m0189-3p | NBO_389g0002 | hypothetical protein NBO_389g0002, partial [Nosema bombycis CQ1] |
| novel-m0189-3p | NBO_38g0002 | hypothetical protein NBO_38g0002 [Nosema bombycis CQ1] |
| novel-m0189-3p | NBO_41g0030 | hypothetical protein NBO_41g0030 [Nosema bombycis CQ1] |
| novel-m0189-3p | NBO_41g0047 | thioltransferase [Nosema bombycis CQ1] |
| novel-m0189-3p | NBO_423g0003 | hypothetical protein NBO_423g0003 [Nosema bombycis CQ1] |
| novel-m0189-3p | NBO_452g0003 | Peptidyl-prolyl cis-trans isomerase NIMA-interacting 1 [Nosema bombycis CQ1] |
| novel-m0189-3p | NBO_468g0005 | Heat shock protein 90 [Nosema bombycis CQ1] |
| novel-m0189-3p | NBO_490g0001 | hypothetical protein NBO_490g0001 [Nosema bombycis CQ1] |
| novel-m0189-3p | NBO_53g0003 | hypothetical protein NBO_53g0003 [Nosema bombycis CQ1] |
| novel-m0189-3p | NBO_53g0017 | glucose-6-phosphate isomerase [Nosema bombycis CQ1] |
| novel-m0189-3p | NBO_547g0003 | hypothetical protein NBO_547g0003 [Nosema bombycis CQ1] |
| novel-m0189-3p | NBO_549g0007 | DNA polymerase epsilon catalytic subunit A [Nosema bombycis CQ1] |
| novel-m0189-3p | NBO_552g0005 | surface-antigen protein P30.4, partial [Nosema bombycis] |
| novel-m0189-3p | NBO_560g0001 | DNA-binding protein SMUBP-2 [Nosema bombycis CQ1] |
| novel-m0189-3p | NBO_591g0001 | Transketolase 1, partial [Nosema bombycis CQ1] |
| novel-m0189-3p | NBO_616g0003 | Heat shock factor protein 1 [Nosema bombycis CQ1] |
| novel-m0189-3p | NBO_63g0015 | hypothetical protein NBO_63g0015, partial [Nosema bombycis CQ1] |
| novel-m0189-3p | NBO_6g0088 | hypothetical protein NBO_6g0088 [Nosema bombycis CQ1] |
| novel-m0189-3p | NBO_707g0002 | Cyclin-dependent kinase C-2 [Nosema bombycis CQ1] |
| novel-m0189-3p | NBO_70g0007 | hypothetical protein NBO_70g0007 [Nosema bombycis CQ1] |
| novel-m0189-3p | NBO_73g0016 | hypothetical protein NBO_73g0016 [Nosema bombycis CQ1] |
| novel-m0189-3p | NBO_76g0003 | hypothetical protein NBO_76g0003 [Nosema bombycis CQ1] |
| novel-m0189-3p | NBO_7g0005 | hypothetical protein NBO_7g0005 [Nosema bombycis CQ1] |
| novel-m0189-3p | NBO_85g0001 | Ubiquitin-like modifier-activating enzyme 1 Y, partial [Nosema bombycis CQ1] |
| novel-m0207-5p | NBO_376g0001 | hypothetical protein NBO_376g0001 [Nosema bombycis CQ1] |
| novel-m0207-5p | NBO_76g0022 | hypothetical protein NBO_76g0022 [Nosema bombycis CQ1] |
| novel-m0010-3p | NBO_1053g0002 | Nuclear pore complex protein Nup98-Nup96 [Nosema bombycis CQ1] |
| novel-m0010-3p | NBO_1116g0004 | hypothetical protein NBO_1116g0004 [Nosema bombycis CQ1] |
| novel-m0010-3p | NBO_29g0015 | Phosphoacetylglucosamine mutase [Nosema bombycis CQ1] |
| novel-m0010-3p | NBO_321g0001 | Phosphomannomutase 2 [Nosema bombycis CQ1] |
| novel-m0010-3p | NBO_338g0001 | hypothetical protein NBO_338g0001 [Nosema bombycis CQ1] |
| novel-m0010-3p | NBO_389g0002 | hypothetical protein NBO_389g0002, partial [Nosema bombycis CQ1] |
| novel-m0010-3p | NBO_62g0012 | hypothetical protein NBO_62g0012 [Nosema bombycis CQ1] |
| novel-m0010-3p | NBO_6g0112 | hypothetical protein NBO_6g0112 [Nosema bombycis CQ1] |
| novel-m0010-3p | NBO_6g0118 | hypothetical protein NBO_6g0118 [Nosema bombycis CQ1] |
| novel-m0010-3p | NBO_72g0014 | hypothetical protein NBO_72g0014 [Nosema bombycis CQ1] |
| novel-m0026-3p | NBO_423g0003 | hypothetical protein NBO_423g0003 [Nosema bombycis CQ1] |
| novel-m0026-3p | NBO_490g0001 | hypothetical protein NBO_490g0001 [Nosema bombycis CQ1] |
| novel-m0026-3p | NBO_4g0007 | hypothetical protein NBO_4g0007 [Nosema bombycis CQ1] |
| novel-m0026-3p | NBO_58g0010 | Chitin synthase 7 [Nosema bombycis CQ1] |
| novel-m0035-5p | NBO_10g0052 | Polar tube protein 3 [Nosema bombycis CQ1] |
| novel-m0035-5p | NBO_1239g0006 | ATP-binding domain protein 4 [Nosema bombycis CQ1] |
| novel-m0035-5p | NBO_22g0008 | ormdl family protein [Nosema ceranae] |
| novel-m0035-5p | NBO_31gi002 | small-conductance mechanosensitive channel protein [Nosema ceranae] |
| novel-m0035-5p | NBO_4g0029 | polar tube protein 3 [Nosema bombycis] |
| novel-m0035-5p | NBO_6gi003 | hypothetical protein NBO_6gi003 [Nosema bombycis CQ1] |
| novel-m0035-5p | NBO_70g0007 | hypothetical protein NBO_70g0007 [Nosema bombycis CQ1] |
| novel-m0035-5p | NBO_71g0003 | hypothetical protein NBO_71g0003, partial [Nosema bombycis CQ1] |
| novel-m0035-5p | NBO_81g0012 | MADS domain containing protein, partial [Nosema bombycis CQ1] |
| novel-m0035-5p | NBO_8g0045 | signal peptidase like protein [Nosema bombycis CQ1] |
| novel-m0043-5p | NBO_1020g0001 | Eukaryotic translation initiation factor 2-alpha kinase 1 [Nosema bombycis CQ1] |
| novel-m0043-5p | NBO_1039g0001 | Proteasome component PUP1 [Nosema bombycis CQ1] |
| novel-m0043-5p | NBO_108g0004 | Heat shock protein 90, partial [Nosema bombycis CQ1] |
| novel-m0043-5p | NBO_1136g0001 | ricin b lectin [Nosema ceranae] |
| novel-m0043-5p | NBO_11g0003 | UTP-glucose-1-phosphate uridylyltransferase [Nosema bombycis CQ1] |
| novel-m0043-5p | NBO_11g0038 | Protein kinase kin1 [Nosema bombycis CQ1] |
| novel-m0043-5p | NBO_11g0064 | Guanine nucleotide-binding protein-like 3 [Nosema bombycis CQ1] |
| novel-m0043-5p | NBO_15g0003 | GPI mannosyltransferase 2 [Nosema bombycis CQ1] |
| novel-m0043-5p | NBO_16g0003 | hypothetical protein NBO_16g0003 [Nosema bombycis CQ1] |
| novel-m0043-5p | NBO_16g0023 | Chaperone protein dnaK [Nosema bombycis CQ1] |
| novel-m0043-5p | NBO_16g0027 | Heat shock 70 kDa protein cognate 4 [Nosema bombycis CQ1] |
| novel-m0043-5p | NBO_19g0026 | U3 small nucleolar ribonucleoprotein imp4 [Nosema bombycis CQ1] |
| novel-m0043-5p | NBO_204g0002 | hypothetical protein NBO_204g0002 [Nosema bombycis CQ1] |
| novel-m0043-5p | NBO_32g0030 | hypothetical protein NBO_32g0030 [Nosema bombycis CQ1] |
| novel-m0043-5p | NBO_366g0002 | Heat shock protein HSP 90-alpha 1 [Nosema bombycis CQ1] |
| novel-m0043-5p | NBO_38g0034 | protein kinase domain-containing protein [Nosema pernyi] |
| novel-m0043-5p | NBO_452g0002 | Sld5 domain-containing protein [Rhizoctonia solani AG-1 IA] |
| novel-m0043-5p | NBO_462g0003 | hypothetical protein NBO_462g0003 [Nosema bombycis CQ1] |
| novel-m0043-5p | NBO_468g0005 | Heat shock protein 90 [Nosema bombycis CQ1] |
| novel-m0043-5p | NBO_4g0017 | Heat shock protein 101 [Nosema bombycis CQ1] |
| novel-m0043-5p | NBO_546g0001 | hypothetical protein NBO_546g0001 [Nosema bombycis CQ1] |
| novel-m0043-5p | NBO_55g0009 | hypothetical protein NBO_55g0009 [Nosema bombycis CQ1] |
| novel-m0043-5p | NBO_586gi001 | valyl trna synthetase [Nosema bombycis CQ1] |
| novel-m0043-5p | NBO_59g0003 | Large subunit GTPase 1 [Nosema bombycis CQ1] |
| novel-m0043-5p | NBO_60g0017 | Transcription-associated protein 1 [Nosema bombycis CQ1] |
| novel-m0043-5p | NBO_648gi001 | serine/threonine protein kinase, partial [Nosema bombycis CQ1] |
| novel-m0043-5p | NBO_6g0060 | hypothetical protein NBO_6g0060 [Nosema bombycis CQ1] |
| novel-m0043-5p | NBO_73g0023 | hypothetical protein NBO_73g0023 [Nosema bombycis CQ1] |
| novel-m0043-5p | NBO_742g0003 | hypothetical protein NBO_742g0003 [Nosema bombycis CQ1] |
| novel-m0043-5p | NBO_76g0013 | hypothetical protein NBO_76g0013 [Nosema bombycis CQ1] |
| novel-m0043-5p | NBO_7g0016 | Polar tube protein 1 [Nosema bombycis CQ1] |
| novel-m0043-5p | NBO_920g0001 | Myosin heavy chain kinase B [Nosema bombycis CQ1] |
| novel-m0043-5p | NBO_925g0001 | Myosin heavy chain kinase B [Nosema bombycis CQ1] |
| novel-m0043-5p | NBO_943g0004 | Polar tube protein 1 [Nosema bombycis CQ1] |
| novel-m0043-5p | NBO_985g0001 | hypothetical protein NBO_985g0001 [Nosema bombycis CQ1] |
| novel-m0050-5p | NBO_1020g0001 | Eukaryotic translation initiation factor 2-alpha kinase 1 [Nosema bombycis CQ1] |
| novel-m0050-5p | NBO_1084g0001 | 78 kDa glucose-regulated protein [Nosema bombycis CQ1] |
| novel-m0050-5p | NBO_10g0001 | dna-directed rna polymerase ii subunit rpb7 [Nosema ceranae] |
| novel-m0050-5p | NBO_10g0052 | Polar tube protein 3 [Nosema bombycis CQ1] |
| novel-m0050-5p | NBO_1118gi001 | glucose/Na cotransporter, partial [Nosema bombycis CQ1] |
| novel-m0050-5p | NBO_111g0002 | Serine palmitoyltransferase 1 [Nosema bombycis CQ1] |
| novel-m0050-5p | NBO_1170gi001 | HSP 101 related protein, partial [Nosema bombycis CQ1] |
| novel-m0050-5p | NBO_11g0046 | Xaa-Pro aminopeptidase 1 [Nosema bombycis CQ1] |
| novel-m0050-5p | NBO_11g0061 | Serine/threonine-protein kinase crk1 [Nosema bombycis CQ1] |
| novel-m0050-5p | NBO_1302g0002 | Superkiller viralicidic activity 2-like 2 [Nosema bombycis CQ1] |
| novel-m0050-5p | NBO_16g0028 | hypothetical protein NBO_16g0028 [Nosema bombycis CQ1] |
| novel-m0050-5p | NBO_16g0047 | Chitin synthase export chaperone [Nosema bombycis CQ1] |
| novel-m0050-5p | NBO_174g0001 | hypothetical protein NBO_174g0001 [Nosema bombycis CQ1] |
| novel-m0050-5p | NBO_18g0028 | sugar permease [Nosema bombycis CQ1] |
| novel-m0050-5p | NBO_20g0004 | Bifunctional xylanase/deacetylase [Nosema bombycis CQ1] |
| novel-m0050-5p | NBO_24g0003 | hypothetical protein NBO_24g0003 [Nosema bombycis CQ1] |
| novel-m0050-5p | NBO_256g0003 | hypothetical protein NBO_256g0003 [Nosema bombycis CQ1] |
| novel-m0050-5p | NBO_261g0001 | endonuclease [Nosema bombycis CQ1] |
| novel-m0050-5p | NBO_26g0013 | hypothetical spore wall protein [Nosema bombycis] |
| novel-m0050-5p | NBO_27g0020 | hypothetical protein NBO_27g0020 [Nosema bombycis CQ1] |
| novel-m0050-5p | NBO_27gi003 | hypothetical protein NBO_27gi003 [Nosema bombycis CQ1] |
| novel-m0050-5p | NBO_31gi002 | small-conductance mechanosensitive channel protein [Nosema ceranae] |
| novel-m0050-5p | NBO_33g0018 | 78 kDa glucose-regulated protein [Nosema bombycis CQ1] |
| novel-m0050-5p | NBO_366g0002 | Heat shock protein HSP 90-alpha 1 [Nosema bombycis CQ1] |
| novel-m0050-5p | NBO_374g0007 | Glucosamine--fructose-6-phosphate aminotransferase 1 [Nosema bombycis CQ1] |
| novel-m0050-5p | NBO_37g0005 | Cystine/glutamate transporter [Nosema bombycis CQ1] |
| novel-m0050-5p | NBO_38g0017 | hypothetical protein NBO_38g0017 [Nosema bombycis CQ1] |
| novel-m0050-5p | NBO_3g0006 | 60S ribosomal protein L6, partial [Nosema bombycis] |
| novel-m0050-5p | NBO_411gi001 | GTP-binding protein [Nosema bombycis CQ1] |
| novel-m0050-5p | NBO_460g0011 | hypothetical protein NBO_460g0011 [Nosema bombycis CQ1] |
| novel-m0050-5p | NBO_46g0002 | hypothetical protein NBO_46g0002 [Nosema bombycis CQ1] |
| novel-m0050-5p | NBO_4g0029 | polar tube protein 3 [Nosema bombycis] |
| novel-m0050-5p | NBO_520g0003 | N(2),N(2)-dimethylguanosine tRNA methyltransferase [Nosema bombycis CQ1] |
| novel-m0050-5p | NBO_53g0017 | glucose-6-phosphate isomerase [Nosema bombycis CQ1] |
| novel-m0050-5p | NBO_55g0009 | hypothetical protein NBO_55g0009 [Nosema bombycis CQ1] |
| novel-m0050-5p | NBO_55g0017 | integral membrane protein [Nosema bombycis CQ1] |
| novel-m0050-5p | NBO_602gi001 | serine palmitoyltransferase subunit 1 [Nosema bombycis CQ1] |
| novel-m0050-5p | NBO_6g0076 | dna-directed rna polymerase ii subunit rpb7 [Nosema ceranae] |
| novel-m0050-5p | NBO_6gi003 | hypothetical protein NBO_6gi003 [Nosema bombycis CQ1] |
| novel-m0050-5p | NBO_72g0013 | Rab GTPase interacting factor Golgi membrane protein [Encephalitozoon romaleae SJ-2008] |
| novel-m0050-5p | NBO_80g0017 | Integrator complex subunit 11 [Nosema bombycis CQ1] |
| novel-m0070-3p | NBO_1084g0001 | 78 kDa glucose-regulated protein [Nosema bombycis CQ1] |
| novel-m0070-3p | NBO_10g0007 | 3-hydroxy-3-methylglutaryl-coenzyme A reductase, partial [Nosema bombycis CQ1] |
| novel-m0070-3p | NBO_11g0064 | Guanine nucleotide-binding protein-like 3 [Nosema bombycis CQ1] |
| novel-m0070-3p | NBO_1232g0001 | hypothetical protein NBO_1232g0001 [Nosema bombycis CQ1] |
| novel-m0070-3p | NBO_153g0004 | zinc metalloprotease [Nosema ceranae] |
| novel-m0070-3p | NBO_24g0003 | hypothetical protein NBO_24g0003 [Nosema bombycis CQ1] |
| novel-m0070-3p | NBO_26g0013 | hypothetical spore wall protein [Nosema bombycis] |
| novel-m0070-3p | NBO_28g0053 | Forkhead box protein K2 [Nosema bombycis CQ1] |
| novel-m0070-3p | NBO_29g0015 | Phosphoacetylglucosamine mutase [Nosema bombycis CQ1] |
| novel-m0070-3p | NBO_38g0012 | hypothetical protein NBO_38g0012 [Nosema bombycis CQ1] |
| novel-m0070-3p | NBO_444g0002 | Chaperone protein dnaJ [Nosema bombycis CQ1] |
| novel-m0070-3p | NBO_48g0008 | Glycerophosphodiester phosphodiesterase gde1 [Nosema bombycis CQ1] |
| novel-m0070-3p | NBO_49g0003 | Fructose-bisphosphate aldolase B [Nosema bombycis CQ1] |
| novel-m0070-3p | NBO_59g0003 | Large subunit GTPase 1 [Nosema bombycis CQ1] |
| novel-m0070-3p | NBO_606g0001 | hypothetical protein NBO_606g0001 [Nosema bombycis CQ1] |
| novel-m0070-3p | NBO_696g0002 | Tryptophanyl-tRNA synthetase [Nosema bombycis CQ1] |
| novel-m0070-3p | NBO_80g0034 | Zinc finger C2H2 protein [Nosema bombycis CQ1] |
| novel-m0070-3p | NBO_92g0005 | Beta-lactamase, class A [Nosema bombycis CQ1] |
| novel-m0080-3p | NBO_19g0018 | Chromosome segregation protein [Nosema bombycis CQ1] |
| novel-m0080-3p | NBO_23g0004 | hypothetical protein NBO_23g0004 [Nosema bombycis CQ1] |
| novel-m0080-3p | NBO_33g0020 | Beta tubulin, autoregulation binding site [Nosema bombycis CQ1] |
| novel-m0080-3p | NBO_401g0005 | YjeF family domain-containing protein [Anncaliia algerae PRA339] |
| novel-m0080-3p | NBO_508g0035 | WD repeat-containing protein, partial [Nosema bombycis CQ1] |
| novel-m0080-3p | NBO_55g0007 | Zinc finger CCCH domain-containing protein 15 [Nosema bombycis CQ1] |
| novel-m0080-3p | NBO_648gi001 | serine/threonine protein kinase, partial [Nosema bombycis CQ1] |
| novel-m0080-3p | NBO_64g0032 | YjeF family domain-containing protein [Anncaliia algerae PRA339] |
| novel-m0080-3p | NBO_6g0088 | hypothetical protein NBO_6g0088 [Nosema bombycis CQ1] |
| novel-m0080-3p | NBO_7g0015 | polar tube protein 2 [Nosema bombycis] |
| novel-m0080-3p | NBO_7g0016 | Polar tube protein 1 [Nosema bombycis CQ1] |
| novel-m0100-5p | NBO_1000gi001 | Protein PNS1, partial [Nosema bombycis CQ1] |
| novel-m0100-5p | NBO_1024g0002 | N(5)-glutamine methyltransferase MTQ2 [Nosema bombycis CQ1] |
| novel-m0100-5p | NBO_1053g0001 | glucose transporter type 3 [Nosema bombycis CQ1] |
| novel-m0100-5p | NBO_10g0094 | hypothetical protein NBO_10g0094, partial [Nosema bombycis CQ1] |
| novel-m0100-5p | NBO_1110g0001 | SEC31-like protein invovled in vesicular transport from ER to Golgi [Nosema bombycis CQ1] |
| novel-m0100-5p | NBO_1110g0002 | Protein transport protein sec31 [Nosema bombycis CQ1] |
| novel-m0100-5p | NBO_114g0004 | Zinc phosphodiesterase ELAC protein 2 [Nosema bombycis CQ1] |
| novel-m0100-5p | NBO_11g0003 | UTP-glucose-1-phosphate uridylyltransferase [Nosema bombycis CQ1] |
| novel-m0100-5p | NBO_1320g0001 | Hexokinase-2 [Nosema bombycis CQ1] |
| novel-m0100-5p | NBO_1517g0004 | Rac-like GTP-binding protein ARAC7 [Nosema bombycis CQ1] |
| novel-m0100-5p | NBO_153g0004 | zinc metalloprotease [Nosema ceranae] |
| novel-m0100-5p | NBO_15g0003 | GPI mannosyltransferase 2 [Nosema bombycis CQ1] |
| novel-m0100-5p | NBO_1608g0001 | Isoleucyl-tRNA synthetase, cytoplasmic [Nosema bombycis CQ1] |
| novel-m0100-5p | NBO_166g0002 | Transcription-associated protein 1, partial [Nosema bombycis CQ1] |
| novel-m0100-5p | NBO_18g0007 | hypothetical protein NBO_18g0007 [Nosema bombycis CQ1] |
| novel-m0100-5p | NBO_20g0002 | Pescadillo like protein [Nosema bombycis CQ1] |
| novel-m0100-5p | NBO_20g0004 | Bifunctional xylanase/deacetylase [Nosema bombycis CQ1] |
| novel-m0100-5p | NBO_20g0008 | peptide chain release factor 2 [Nosema ceranae] |
| novel-m0100-5p | NBO_24g0003 | hypothetical protein NBO_24g0003 [Nosema bombycis CQ1] |
| novel-m0100-5p | NBO_26g0007 | hypothetical protein NBO_26g0007 [Nosema bombycis CQ1] |
| novel-m0100-5p | NBO_27gi003 | hypothetical protein NBO_27gi003 [Nosema bombycis CQ1] |
| novel-m0100-5p | NBO_29g0016 | hypothetical protein NBO_29g0016, partial [Nosema bombycis CQ1] |
| novel-m0100-5p | NBO_29g0031 | Solute carrier family 2, facilitated glucose transporter member 3 [Nosema bombycis CQ1] |
| novel-m0100-5p | NBO_2g0012 | Thymidine kinase [Nosema bombycis CQ1] |
| novel-m0100-5p | NBO_318g0001 | hypothetical protein NBO_318g0001 [Nosema bombycis CQ1] |
| novel-m0100-5p | NBO_33gi001 | Protein PNS1, partial [Nosema bombycis CQ1] |
| novel-m0100-5p | NBO_350g0001 | Protein PNS1, partial [Nosema bombycis CQ1] |
| novel-m0100-5p | NBO_35g0002 | serpin-like protein [Nosema bombycis CQ1] |
| novel-m0100-5p | NBO_367g0001 | hypothetical protein NBO_367g0001 [Nosema bombycis CQ1] |
| novel-m0100-5p | NBO_375g0010 | 2,3-bisphosphoglycerate-independent phosphoglycerate mutase [Nosema bombycis CQ1] |
| novel-m0100-5p | NBO_376g0007 | hypothetical protein NBO_376g0007 [Nosema bombycis CQ1] |
| novel-m0100-5p | NBO_37g0005 | Cystine/glutamate transporter [Nosema bombycis CQ1] |
| novel-m0100-5p | NBO_417g0011 | Solute carrier family 35 member C2, partial [Nosema bombycis CQ1] |
| novel-m0100-5p | NBO_417g0013 | ATP-binding protein [Ordospora colligata OC4] |
| novel-m0100-5p | NBO_418gi001 | integral membrane protein [Nosema bombycis CQ1] |
| novel-m0100-5p | NBO_41g0014 | Vacuolar amino acid transporter 5 [Nosema bombycis CQ1] |
| novel-m0100-5p | NBO_41g0024 | Vacuolar amino acid transporter 5 [Nosema bombycis CQ1] |
| novel-m0100-5p | NBO_423g0001 | NADPH-dependent diflavin oxidoreductase 1 [Nosema bombycis CQ1] |
| novel-m0100-5p | NBO_448g0002 | hypothetical protein NBO_448g0002 [Nosema bombycis CQ1] |
| novel-m0100-5p | NBO_44g0010 | KH domain-containing protein [Nosema bombycis CQ1] |
| novel-m0100-5p | NBO_452g0002 | Sld5 domain-containing protein [Rhizoctonia solani AG-1 IA] |
| novel-m0100-5p | NBO_464g0008 | Isoleucyl-tRNA synthetase [Nosema bombycis CQ1] |
| novel-m0100-5p | NBO_480g0001 | hypothetical protein NBO_480g0001 [Nosema bombycis CQ1] |
| novel-m0100-5p | NBO_48g0004 | mevalonate kinase [Nosema bombycis CQ1] |
| novel-m0100-5p | NBO_490g0001 | hypothetical protein NBO_490g0001 [Nosema bombycis CQ1] |
| novel-m0100-5p | NBO_508g0021 | Rac-like GTP-binding protein ARAC7 [Nosema bombycis CQ1] |
| novel-m0100-5p | NBO_53g0017 | glucose-6-phosphate isomerase [Nosema bombycis CQ1] |
| novel-m0100-5p | NBO_549g0007 | DNA polymerase epsilon catalytic subunit A [Nosema bombycis CQ1] |
| novel-m0100-5p | NBO_55g0012 | hypothetical protein NBO_55g0012 [Nosema bombycis CQ1] |
| novel-m0100-5p | NBO_58g0022 | hypothetical protein NBO_58g0022 [Nosema bombycis CQ1] |
| novel-m0100-5p | NBO_59g0003 | Large subunit GTPase 1 [Nosema bombycis CQ1] |
| novel-m0100-5p | NBO_63g0015 | hypothetical protein NBO_63g0015, partial [Nosema bombycis CQ1] |
| novel-m0100-5p | NBO_63g0022 | hypothetical protein NBO_63g0022 [Nosema bombycis CQ1] |
| novel-m0100-5p | NBO_71g0002 | isoleucyl-tRNA synthetase, cytoplasmic [Nosema bombycis CQ1] |
| novel-m0100-5p | NBO_71g0003 | hypothetical protein NBO_71g0003, partial [Nosema bombycis CQ1] |
| novel-m0100-5p | NBO_72g0013 | Rab GTPase interacting factor Golgi membrane protein [Encephalitozoon romaleae SJ-2008] |
| novel-m0100-5p | NBO_742g0003 | hypothetical protein NBO_742g0003 [Nosema bombycis CQ1] |
| novel-m0100-5p | NBO_76gi003 | actin-like 53kDa protein [Nosema bombycis CQ1] |
| novel-m0100-5p | NBO_78g0012 | ATP-binding cassette sub-family B member 7, mitochondrial [Nosema bombycis CQ1] |
| novel-m0100-5p | NBO_7g0040 | hypothetical protein NBO_7g0040, partial [Nosema bombycis CQ1] |
| novel-m0100-5p | NBO_80g0017 | Integrator complex subunit 11 [Nosema bombycis CQ1] |
| novel-m0100-5p | NBO_80g0024 | Major facilitator superfamily domain-containing protein 1 [Nosema bombycis CQ1] |
| novel-m0100-5p | NBO_8g0003 | hypothetical protein NBO_8g0003 [Nosema bombycis CQ1] |
| novel-m0100-5p | NBO_8g0045 | signal peptidase like protein [Nosema bombycis CQ1] |
| novel-m0100-5p | NBO_915g0002 | Cell division control protein 48 E, partial [Nosema bombycis CQ1] |
| novel-m0100-5p | NBO_937g0002 | Sec61beta [Nosema bombycis CQ1] |
| novel-m0129-3p | NBO_19g0018 | Chromosome segregation protein [Nosema bombycis CQ1] |
| novel-m0129-3p | NBO_23g0004 | hypothetical protein NBO_23g0004 [Nosema bombycis CQ1] |
| novel-m0129-3p | NBO_33g0020 | Beta tubulin, autoregulation binding site [Nosema bombycis CQ1] |
| novel-m0129-3p | NBO_401g0005 | YjeF family domain-containing protein [Anncaliia algerae PRA339] |
| novel-m0129-3p | NBO_508g0035 | WD repeat-containing protein, partial [Nosema bombycis CQ1] |
| novel-m0129-3p | NBO_55g0007 | Zinc finger CCCH domain-containing protein 15 [Nosema bombycis CQ1] |
| novel-m0129-3p | NBO_648gi001 | serine/threonine protein kinase, partial [Nosema bombycis CQ1] |
| novel-m0129-3p | NBO_64g0032 | YjeF family domain-containing protein [Anncaliia algerae PRA339] |
| novel-m0129-3p | NBO_6g0088 | hypothetical protein NBO_6g0088 [Nosema bombycis CQ1] |
| novel-m0129-3p | NBO_7g0015 | polar tube protein 2 [Nosema bombycis] |
| novel-m0129-3p | NBO_7g0016 | Polar tube protein 1 [Nosema bombycis CQ1] |
| novel-m0148-3p | NBO_1053g0002 | Nuclear pore complex protein Nup98-Nup96 [Nosema bombycis CQ1] |
| novel-m0148-3p | NBO_1133g0001 | Ricin B lectin [Nosema bombycis CQ1] |
| novel-m0148-3p | NBO_114g0004 | Zinc phosphodiesterase ELAC protein 2 [Nosema bombycis CQ1] |
| novel-m0148-3p | NBO_11g0038 | Protein kinase kin1 [Nosema bombycis CQ1] |
| novel-m0148-3p | NBO_11g0045 | hypothetical protein NBO_11g0045 [Nosema bombycis CQ1] |
| novel-m0148-3p | NBO_16g0023 | Chaperone protein dnaK [Nosema bombycis CQ1] |
| novel-m0148-3p | NBO_16g0027 | Heat shock 70 kDa protein cognate 4 [Nosema bombycis CQ1] |
| novel-m0148-3p | NBO_24g0011 | hypothetical protein NBO_24g0011 [Nosema bombycis CQ1] |
| novel-m0148-3p | NBO_26g0007 | hypothetical protein NBO_26g0007 [Nosema bombycis CQ1] |
| novel-m0148-3p | NBO_27g0027 | ribosomal protein hs6-type [Nosema apis BRL 01] |
| novel-m0148-3p | NBO_28g0050 | hypothetical protein NBO_28g0050 [Nosema bombycis CQ1] |
| novel-m0148-3p | NBO_293g0001 | hypothetical protein NBO_293g0001 [Nosema bombycis CQ1] |
| novel-m0148-3p | NBO_30g0002 | hypothetical protein NBO_30g0002 [Nosema bombycis CQ1] |
| novel-m0148-3p | NBO_34g0010 | mitochondrial protein import protein MAS5 [Nosema pernyi] |
| novel-m0148-3p | NBO_36g0002 | Homeobox protein HD-8 [Nosema bombycis CQ1] |
| novel-m0148-3p | NBO_38g0002 | hypothetical protein NBO_38g0002 [Nosema bombycis CQ1] |
| novel-m0148-3p | NBO_423g0006 | Iron-sulfur clusters transporter atm1, mitochondrial, partial [Nosema bombycis CQ1] |
| novel-m0148-3p | NBO_447g0002 | Meiosis-specific protein HOP1 [Nosema bombycis CQ1] |
| novel-m0148-3p | NBO_448g0002 | hypothetical protein NBO_448g0002 [Nosema bombycis CQ1] |
| novel-m0148-3p | NBO_459g0003 | Meiosis-specific protein HOP1 [Nosema bombycis CQ1] |
| novel-m0148-3p | NBO_463g0002 | Ricin B lectin [Nosema bombycis CQ1] |
| novel-m0148-3p | NBO_499g0002 | SKP1 component [Nosema bombycis CQ1] |
| novel-m0148-3p | NBO_4g0031 | elongation factor 2 [Nosema bombycis CQ1] |
| novel-m0148-3p | NBO_53g0017 | glucose-6-phosphate isomerase [Nosema bombycis CQ1] |
| novel-m0148-3p | NBO_547g0003 | hypothetical protein NBO_547g0003 [Nosema bombycis CQ1] |
| novel-m0148-3p | NBO_55g0005 | mitochondrial pyruvate dehydrogenase E1 component subunit alpha [Nosema bombycis] |
| novel-m0148-3p | NBO_63g0022 | hypothetical protein NBO_63g0022 [Nosema bombycis CQ1] |
| novel-m0148-3p | NBO_6g0004 | hypothetical protein NBO_6g0004 [Nosema bombycis CQ1] |
| novel-m0148-3p | NBO_6g0048 | Ricin B lectin [Nosema bombycis CQ1] |
| novel-m0148-3p | NBO_6g0049 | Ricin B lectin [Nosema bombycis CQ1] |
| novel-m0148-3p | NBO_6g0085 | 40S ribosomal protein S10 [Amphiamblys sp. WSBS2006] |
| novel-m0148-3p | NBO_7g0040 | hypothetical protein NBO_7g0040, partial [Nosema bombycis CQ1] |
| novel-m0148-3p | NBO_920g0001 | Myosin heavy chain kinase B [Nosema bombycis CQ1] |
| novel-m0167-5p | NBO_1024g0002 | N(5)-glutamine methyltransferase MTQ2 [Nosema bombycis CQ1] |
| novel-m0167-5p | NBO_1054gi001 | T complex protein 1 subunit beta [Nosema bombycis CQ1] |
| novel-m0167-5p | NBO_11g0038 | Protein kinase kin1 [Nosema bombycis CQ1] |
| novel-m0167-5p | NBO_11g0069 | hypothetical protein NBO_11g0069 [Nosema bombycis CQ1] |
| novel-m0167-5p | NBO_1302g0001 | hypothetical protein NBO_1302g0001 [Nosema bombycis CQ1] |
| novel-m0167-5p | NBO_1314g0001 | hypothetical protein NBO_1314g0001 [Nosema bombycis CQ1] |
| novel-m0167-5p | NBO_13g0057 | transcription factor TAU-like protein [Nosema bombycis CQ1] |
| novel-m0167-5p | NBO_13g0072 | hypothetical protein NBO_13g0072 [Nosema bombycis CQ1] |
| novel-m0167-5p | NBO_198gi002 | phosphate-transporting ATPase [Trachipleistophora hominis] |
| novel-m0167-5p | NBO_20g0024 | cdc73-like rna polymerase ii accessory factor [Nosema ceranae] |
| novel-m0167-5p | NBO_24g0011 | hypothetical protein NBO_24g0011 [Nosema bombycis CQ1] |
| novel-m0167-5p | NBO_27g0006 | integral membrane [Nosema apis BRL 01] |
| novel-m0167-5p | NBO_28g0053 | Forkhead box protein K2 [Nosema bombycis CQ1] |
| novel-m0167-5p | NBO_29gi002 | alpha,alpha trehalose-phosphate synthase [Nosema bombycis CQ1] |
| novel-m0167-5p | NBO_32g0024 | General transcriptional corepressor ssn6 [Nosema bombycis CQ1] |
| novel-m0167-5p | NBO_32g0030 | hypothetical protein NBO_32g0030 [Nosema bombycis CQ1] |
| novel-m0167-5p | NBO_366gi001 | phosphate-transporting ATPase [Trachipleistophora hominis] |
| novel-m0167-5p | NBO_36g0002 | Homeobox protein HD-8 [Nosema bombycis CQ1] |
| novel-m0167-5p | NBO_37g0007 | KH domain-containing protein [Nosema bombycis CQ1] |
| novel-m0167-5p | NBO_380g0001 | hypothetical protein NBO_380g0001 [Nosema bombycis CQ1] |
| novel-m0167-5p | NBO_41g0019 | eukaryotic translation initiation factor 4a [Nosema ceranae] |
| novel-m0167-5p | NBO_41g0030 | hypothetical protein NBO_41g0030 [Nosema bombycis CQ1] |
| novel-m0167-5p | NBO_43g0006 | ATP-dependent RNA helicase DHX37 [Nosema bombycis CQ1] |
| novel-m0167-5p | NBO_44g0010 | KH domain-containing protein [Nosema bombycis CQ1] |
| novel-m0167-5p | NBO_46g0007 | hypothetical protein NBO_380g0001 [Nosema bombycis CQ1] |
| novel-m0167-5p | NBO_483g0001 | hypothetical protein NBO_483g0001 [Nosema bombycis CQ1] |
| novel-m0167-5p | NBO_4g0050 | Insulin-degrading enzyme [Nosema bombycis CQ1] |
| novel-m0167-5p | NBO_508g0035 | WD repeat-containing protein, partial [Nosema bombycis CQ1] |
| novel-m0167-5p | NBO_520g0003 | N(2),N(2)-dimethylguanosine tRNA methyltransferase [Nosema bombycis CQ1] |
| novel-m0167-5p | NBO_53g0008 | Ubiquitin thioesterase otubain-like protein [Nosema bombycis CQ1] |
| novel-m0167-5p | NBO_555g0001 | hypothetical protein NBO_555g0001 [Nosema bombycis CQ1] |
| novel-m0167-5p | NBO_568g0004 | Alpha,alpha-trehalose-phosphate synthase, partial [Nosema bombycis CQ1] |
| novel-m0167-5p | NBO_582gi001 | alpha,alpha trehalose-phosphate synthase, partial [Nosema bombycis CQ1] |
| novel-m0167-5p | NBO_58g0009 | DNA repair protein RAD16 [Nosema bombycis CQ1] |
| novel-m0167-5p | NBO_591g0001 | Transketolase 1, partial [Nosema bombycis CQ1] |
| novel-m0167-5p | NBO_67g0015 | hypothetical protein NBO_67g0015 [Nosema bombycis CQ1] |
| novel-m0167-5p | NBO_696g0002 | Tryptophanyl-tRNA synthetase [Nosema bombycis CQ1] |
| novel-m0167-5p | NBO_6g0015 | Ricin B lectin [Nosema bombycis CQ1] |
| novel-m0167-5p | NBO_6g0030 | ATP-dependent DNA helicase hus2/rqh1 [Nosema bombycis CQ1] |
| novel-m0167-5p | NBO_6g0112 | hypothetical protein NBO_6g0112 [Nosema bombycis CQ1] |
| novel-m0167-5p | NBO_71g0002 | isoleucyl-tRNA synthetase, cytoplasmic [Nosema bombycis CQ1] |
| novel-m0167-5p | NBO_73g0023 | hypothetical protein NBO_73g0023 [Nosema bombycis CQ1] |
| novel-m0167-5p | NBO_74g0004 | small-conductance mechanosensitive channel protein [Nosema ceranae] |
| novel-m0167-5p | NBO_770g0002 | Ubiquitin thioesterase otubain-like protein [Nosema bombycis CQ1] |
| novel-m0167-5p | NBO_7g0016 | Polar tube protein 1 [Nosema bombycis CQ1] |
| novel-m0167-5p | NBO_7g0040 | hypothetical protein NBO_7g0040, partial [Nosema bombycis CQ1] |
| novel-m0167-5p | NBO_943g0004 | Polar tube protein 1 [Nosema bombycis CQ1] |
| novel-m0167-5p | NBO_985g0001 | hypothetical protein NBO_985g0001 [Nosema bombycis CQ1] |
| novel-m0167-5p | NBO_9g0005 | 60S ribosomal protein L10 [Nosema bombycis] |
| novel-m0192-5p | NBO_1020g0001 | Eukaryotic translation initiation factor 2-alpha kinase 1 [Nosema bombycis CQ1] |
| novel-m0192-5p | NBO_1084g0001 | 78 kDa glucose-regulated protein [Nosema bombycis CQ1] |
| novel-m0192-5p | NBO_10g0001 | dna-directed rna polymerase ii subunit rpb7 [Nosema ceranae] |
| novel-m0192-5p | NBO_10g0052 | Polar tube protein 3 [Nosema bombycis CQ1] |
| novel-m0192-5p | NBO_1118gi001 | glucose/Na cotransporter, partial [Nosema bombycis CQ1] |
| novel-m0192-5p | NBO_111g0002 | Serine palmitoyltransferase 1 [Nosema bombycis CQ1] |
| novel-m0192-5p | NBO_1170gi001 | HSP 101 related protein, partial [Nosema bombycis CQ1] |
| novel-m0192-5p | NBO_11g0046 | Xaa-Pro aminopeptidase 1 [Nosema bombycis CQ1] |
| novel-m0192-5p | NBO_11g0061 | Serine/threonine-protein kinase crk1 [Nosema bombycis CQ1] |
| novel-m0192-5p | NBO_1302g0002 | Superkiller viralicidic activity 2-like 2 [Nosema bombycis CQ1] |
| novel-m0192-5p | NBO_16g0028 | hypothetical protein NBO_16g0028 [Nosema bombycis CQ1] |
| novel-m0192-5p | NBO_16g0047 | Chitin synthase export chaperone [Nosema bombycis CQ1] |
| novel-m0192-5p | NBO_174g0001 | hypothetical protein NBO_174g0001 [Nosema bombycis CQ1] |
| novel-m0192-5p | NBO_18g0028 | sugar permease [Nosema bombycis CQ1] |
| novel-m0192-5p | NBO_20g0004 | Bifunctional xylanase/deacetylase [Nosema bombycis CQ1] |
| novel-m0192-5p | NBO_24g0003 | hypothetical protein NBO_24g0003 [Nosema bombycis CQ1] |
| novel-m0192-5p | NBO_256g0003 | hypothetical protein NBO_256g0003 [Nosema bombycis CQ1] |
| novel-m0192-5p | NBO_261g0001 | endonuclease [Nosema bombycis CQ1] |
| novel-m0192-5p | NBO_26g0013 | hypothetical spore wall protein [Nosema bombycis] |
| novel-m0192-5p | NBO_27g0020 | hypothetical protein NBO_27g0020 [Nosema bombycis CQ1] |
| novel-m0192-5p | NBO_27gi003 | hypothetical protein NBO_27gi003 [Nosema bombycis CQ1] |
| novel-m0192-5p | NBO_31gi002 | small-conductance mechanosensitive channel protein [Nosema ceranae] |
| novel-m0192-5p | NBO_33g0018 | 78 kDa glucose-regulated protein [Nosema bombycis CQ1] |
| novel-m0192-5p | NBO_366g0002 | Heat shock protein HSP 90-alpha 1 [Nosema bombycis CQ1] |
| novel-m0192-5p | NBO_374g0007 | Glucosamine--fructose-6-phosphate aminotransferase 1 [Nosema bombycis CQ1] |
| novel-m0192-5p | NBO_37g0005 | Cystine/glutamate transporter [Nosema bombycis CQ1] |
| novel-m0192-5p | NBO_38g0017 | hypothetical protein NBO_38g0017 [Nosema bombycis CQ1] |
| novel-m0192-5p | NBO_3g0006 | 60S ribosomal protein L6, partial [Nosema bombycis] |
| novel-m0192-5p | NBO_411gi001 | GTP-binding protein [Nosema bombycis CQ1] |
| novel-m0192-5p | NBO_460g0011 | hypothetical protein NBO_460g0011 [Nosema bombycis CQ1] |
| novel-m0192-5p | NBO_46g0002 | hypothetical protein NBO_46g0002 [Nosema bombycis CQ1] |
| novel-m0192-5p | NBO_4g0029 | polar tube protein 3 [Nosema bombycis] |
| novel-m0192-5p | NBO_520g0003 | N(2),N(2)-dimethylguanosine tRNA methyltransferase [Nosema bombycis CQ1] |
| novel-m0192-5p | NBO_53g0017 | glucose-6-phosphate isomerase [Nosema bombycis CQ1] |
| novel-m0192-5p | NBO_55g0009 | hypothetical protein NBO_55g0009 [Nosema bombycis CQ1] |
| novel-m0192-5p | NBO_55g0017 | integral membrane protein [Nosema bombycis CQ1] |
| novel-m0192-5p | NBO_602gi001 | serine palmitoyltransferase subunit 1 [Nosema bombycis CQ1] |
| novel-m0192-5p | NBO_6g0076 | dna-directed rna polymerase ii subunit rpb7 [Nosema ceranae] |
| novel-m0192-5p | NBO_6gi003 | hypothetical protein NBO_6gi003 [Nosema bombycis CQ1] |
| novel-m0192-5p | NBO_72g0013 | Rab GTPase interacting factor Golgi membrane protein [Encephalitozoon romaleae SJ-2008] |
| novel-m0192-5p | NBO_80g0017 | Integrator complex subunit 11 [Nosema bombycis CQ1] |
| novel-m0205-5p | NBO_11g0012 | Homeobox protein HD-10 [Nosema bombycis CQ1] |
| novel-m0205-5p | NBO_24g0011 | hypothetical protein NBO_24g0011 [Nosema bombycis CQ1] |
| novel-m0205-5p | NBO_85g0005 | Homeobox protein HD-10 [Nosema bombycis CQ1] |
| novel-m0222-3p | NBO_12g0010 | pseudouridylate synthase [Nosema ceranae] |
| novel-m0080-5p | NBO_1302g0002 | Superkiller viralicidic activity 2-like 2 [Nosema bombycis CQ1] |
| novel-m0080-5p | NBO_9g0005 | 60S ribosomal protein L10 [Nosema bombycis] |
| novel-m0086-5p | NBO_1203g0001 | hypothetical protein NBO_1203g0001 [Nosema bombycis CQ1] |
| novel-m0086-5p | NBO_41g0024 | Vacuolar amino acid transporter 5 [Nosema bombycis CQ1] |
| novel-m0086-5p | NBO_54g0012 | hypothetical protein NBO_54g0012 [Nosema bombycis CQ1] |
| novel-m0094-3p | NBO_1010g0001 | Pescadillo-like protein [Nosema bombycis CQ1] |
| novel-m0094-3p | NBO_1066g0001 | hypothetical protein NBO_1066g0001 [Nosema bombycis CQ1] |
| novel-m0094-3p | NBO_1118gi001 | glucose/Na cotransporter, partial [Nosema bombycis CQ1] |
| novel-m0094-3p | NBO_1303g0002 | hypothetical protein NBO_1303g0002 [Nosema bombycis CQ1] |
| novel-m0094-3p | NBO_1344g0003 | magnesium transporter alr2 [Nosema ceranae] |
| novel-m0094-3p | NBO_137g0001 | hypothetical protein NBO_137g0001 [Nosema bombycis CQ1] |
| novel-m0094-3p | NBO_1530gi001 | tryptophanyl tRNA synthetase [Nosema bombycis CQ1] |
| novel-m0094-3p | NBO_153g0004 | zinc metalloprotease [Nosema ceranae] |
| novel-m0094-3p | NBO_16g0047 | Chitin synthase export chaperone [Nosema bombycis CQ1] |
| novel-m0094-3p | NBO_204g0002 | hypothetical protein NBO_204g0002 [Nosema bombycis CQ1] |
| novel-m0094-3p | NBO_20g0002 | Pescadillo like protein [Nosema bombycis CQ1] |
| novel-m0094-3p | NBO_24g0003 | hypothetical protein NBO_24g0003 [Nosema bombycis CQ1] |
| novel-m0094-3p | NBO_24g0012 | nucleotide excision repair factor TFIIH/TFIIK subunit cyclin H-like protein [Encephalitozoon intestinalis ATCC 50506] |
| novel-m0094-3p | NBO_29g0027 | trehalose-phosphatase, partial [Nosema bombycis CQ1] |
| novel-m0094-3p | NBO_29g0031 | Solute carrier family 2, facilitated glucose transporter member 3 [Nosema bombycis CQ1] |
| novel-m0094-3p | NBO_33g0017 | hypothetical protein NBO_33g0017 [Nosema bombycis CQ1] |
| novel-m0094-3p | NBO_34g0010 | mitochondrial protein import protein MAS5 [Nosema pernyi] |
| novel-m0094-3p | NBO_362g0011 | CDP-diacylglycerol--inositol 3-phosphatidyltransferase [Nosema bombycis CQ1] |
| novel-m0094-3p | NBO_389g0001 | U3 small nucleolar RNA-associated protein 12 [Nosema bombycis CQ1] |
| novel-m0094-3p | NBO_38g0025 | eukaryotic translation initiation factor 2C 2 [Nosema bombycis CQ1] |
| novel-m0094-3p | NBO_462g0003 | hypothetical protein NBO_462g0003 [Nosema bombycis CQ1] |
| novel-m0094-3p | NBO_64g0051 | hypothetical protein NBO_64g0051 [Nosema bombycis CQ1] |
| novel-m0094-3p | NBO_6g0011 | hypothetical protein NBO_6g0011 [Nosema bombycis CQ1] |
| novel-m0094-3p | NBO_800gi001 | dopey-like leucine zipper transcription factor [Nosema ceranae] |
| novel-m0094-3p | NBO_80g0024 | Major facilitator superfamily domain-containing protein 1 [Nosema bombycis CQ1] |
| novel-m0100-3p | NBO_1084g0001 | 78 kDa glucose-regulated protein [Nosema bombycis CQ1] |
| novel-m0100-3p | NBO_108g0004 | Heat shock protein 90, partial [Nosema bombycis CQ1] |
| novel-m0100-3p | NBO_10g0053 | DNA mismatch repair protein Mlh1 [Nosema bombycis CQ1] |
| novel-m0100-3p | NBO_1230g0003 | swi5-like protein [Nosema ceranae] |
| novel-m0100-3p | NBO_13g0057 | transcription factor TAU-like protein [Nosema bombycis CQ1] |
| novel-m0100-3p | NBO_15g0003 | GPI mannosyltransferase 2 [Nosema bombycis CQ1] |
| novel-m0100-3p | NBO_16g0051 | hypothetical protein NBO_16g0051 [Nosema bombycis CQ1] |
| novel-m0100-3p | NBO_20g0002 | Pescadillo like protein [Nosema bombycis CQ1] |
| novel-m0100-3p | NBO_20g0008 | peptide chain release factor 2 [Nosema ceranae] |
| novel-m0100-3p | NBO_24g0012 | nucleotide excision repair factor TFIIH/TFIIK subunit cyclin H-like protein [Encephalitozoon intestinalis ATCC 50506] |
| novel-m0100-3p | NBO_261g0001 | endonuclease [Nosema bombycis CQ1] |
| novel-m0100-3p | NBO_26g0007 | hypothetical protein NBO_26g0007 [Nosema bombycis CQ1] |
| novel-m0100-3p | NBO_28g0052 | Nucleolar essential protein 1 [Nosema bombycis CQ1] |
| novel-m0100-3p | NBO_28g0061 | Cleavage and polyadenylation specificity factor subunit 4 [Nosema bombycis CQ1] |
| novel-m0100-3p | NBO_29g0027 | trehalose-phosphatase, partial [Nosema bombycis CQ1] |
| novel-m0100-3p | NBO_29gi002 | alpha,alpha trehalose-phosphate synthase [Nosema bombycis CQ1] |
| novel-m0100-3p | NBO_318g0001 | hypothetical protein NBO_318g0001 [Nosema bombycis CQ1] |
| novel-m0100-3p | NBO_31gi002 | small-conductance mechanosensitive channel protein [Nosema ceranae] |
| novel-m0100-3p | NBO_364g0016 | hypothetical protein NBO_364g0016 [Nosema bombycis CQ1] |
| novel-m0100-3p | NBO_366g0002 | Heat shock protein HSP 90-alpha 1 [Nosema bombycis CQ1] |
| novel-m0100-3p | NBO_377g0003 | Heat shock 70 kDa protein 6 [Nosema bombycis CQ1] |
| novel-m0100-3p | NBO_389g0002 | hypothetical protein NBO_389g0002, partial [Nosema bombycis CQ1] |
| novel-m0100-3p | NBO_38g0002 | hypothetical protein NBO_38g0002 [Nosema bombycis CQ1] |
| novel-m0100-3p | NBO_3g0016 | hypothetical protein NBO_3g0016 [Nosema bombycis CQ1] |
| novel-m0100-3p | NBO_41g0019 | eukaryotic translation initiation factor 4a [Nosema ceranae] |
| novel-m0100-3p | NBO_41g0030 | hypothetical protein NBO_41g0030 [Nosema bombycis CQ1] |
| novel-m0100-3p | NBO_444g0008 | Cysteinyl-tRNA synthetase, cytoplasmic [Nosema bombycis CQ1] |
| novel-m0100-3p | NBO_508g0035 | WD repeat-containing protein, partial [Nosema bombycis CQ1] |
| novel-m0100-3p | NBO_547g0003 | hypothetical protein NBO_547g0003 [Nosema bombycis CQ1] |
| novel-m0100-3p | NBO_568g0004 | Alpha,alpha-trehalose-phosphate synthase, partial [Nosema bombycis CQ1] |
| novel-m0100-3p | NBO_582gi001 | alpha,alpha trehalose-phosphate synthase, partial [Nosema bombycis CQ1] |
| novel-m0100-3p | NBO_58g0022 | hypothetical protein NBO_58g0022 [Nosema bombycis CQ1] |
| novel-m0100-3p | NBO_60g0027 | histone-binding protein N1/N2 [Nosema bombycis CQ1] |
| novel-m0100-3p | NBO_63g0015 | hypothetical protein NBO_63g0015, partial [Nosema bombycis CQ1] |
| novel-m0100-3p | NBO_63g0018 | hypothetical protein NBO_63g0018 [Nosema bombycis CQ1] |
| novel-m0100-3p | NBO_66g0005 | hypothetical protein NBO_66g0005 [Nosema bombycis CQ1] |
| novel-m0100-3p | NBO_6g0088 | hypothetical protein NBO_6g0088 [Nosema bombycis CQ1] |
| novel-m0100-3p | NBO_71g0003 | hypothetical protein NBO_71g0003, partial [Nosema bombycis CQ1] |
| novel-m0100-3p | NBO_73g0026 | hypothetical protein NBO_73g0026 [Nosema bombycis CQ1] |
| novel-m0100-3p | NBO_73g0028 | hypothetical protein NBO_73g0028 [Nosema bombycis CQ1] |
| novel-m0100-3p | NBO_915g0002 | Cell division control protein 48 E, partial [Nosema bombycis CQ1] |
| novel-m0103-5p | NBO_10g0037 | hypothetical protein NBO_10g0037 [Nosema bombycis CQ1] |
| novel-m0103-5p | NBO_12g0014 | ribosome biogenesis regulatory protein [Nosema ceranae] |
| novel-m0103-5p | NBO_480g0001 | hypothetical protein NBO_480g0001 [Nosema bombycis CQ1] |
| novel-m0103-5p | NBO_63g0020 | Glutamate synthase NADPH small chain [Nosema bombycis CQ1] |
| novel-m0120-3p | NBO_27g0019 | hypothetical protein NBO_27g0019, partial [Nosema bombycis CQ1] |
| novel-m0120-3p | NBO_586gi001 | valyl trna synthetase [Nosema bombycis CQ1] |
| novel-m0120-3p | NBO_63g0020 | Glutamate synthase NADPH small chain [Nosema bombycis CQ1] |
| novel-m0120-3p | NBO_6g0062 | Ricin B lectin [Nosema bombycis CQ1] |
| novel-m0122-5p | NBO_10g0037 | hypothetical protein NBO_10g0037 [Nosema bombycis CQ1] |
| novel-m0122-5p | NBO_12g0014 | ribosome biogenesis regulatory protein [Nosema ceranae] |
| novel-m0122-5p | NBO_480g0001 | hypothetical protein NBO_480g0001 [Nosema bombycis CQ1] |
| novel-m0122-5p | NBO_63g0020 | Glutamate synthase NADPH small chain [Nosema bombycis CQ1] |
| novel-m0133-3p | NBO_27g0019 | hypothetical protein NBO_27g0019, partial [Nosema bombycis CQ1] |
| novel-m0133-3p | NBO_586gi001 | valyl trna synthetase [Nosema bombycis CQ1] |
| novel-m0133-3p | NBO_63g0020 | Glutamate synthase NADPH small chain [Nosema bombycis CQ1] |
| novel-m0133-3p | NBO_6g0062 | Ricin B lectin [Nosema bombycis CQ1] |
| novel-m0138-5p | NBO_10g0037 | hypothetical protein NBO_10g0037 [Nosema bombycis CQ1] |
| novel-m0138-5p | NBO_12g0014 | ribosome biogenesis regulatory protein [Nosema ceranae] |
| novel-m0138-5p | NBO_480g0001 | hypothetical protein NBO_480g0001 [Nosema bombycis CQ1] |
| novel-m0138-5p | NBO_63g0020 | Glutamate synthase NADPH small chain [Nosema bombycis CQ1] |
| novel-m0145-3p | NBO_27g0019 | hypothetical protein NBO_27g0019, partial [Nosema bombycis CQ1] |
| novel-m0145-3p | NBO_586gi001 | valyl trna synthetase [Nosema bombycis CQ1] |
| novel-m0145-3p | NBO_63g0020 | Glutamate synthase NADPH small chain [Nosema bombycis CQ1] |
| novel-m0145-3p | NBO_6g0062 | Ricin B lectin [Nosema bombycis CQ1] |
| novel-m0152-3p | NBO_27g0019 | hypothetical protein NBO_27g0019, partial [Nosema bombycis CQ1] |
| novel-m0152-3p | NBO_586gi001 | valyl trna synthetase [Nosema bombycis CQ1] |
| novel-m0152-3p | NBO_63g0020 | Glutamate synthase NADPH small chain [Nosema bombycis CQ1] |
| novel-m0152-3p | NBO_6g0062 | Ricin B lectin [Nosema bombycis CQ1] |
| novel-m0161-3p | NBO_27g0019 | hypothetical protein NBO_27g0019, partial [Nosema bombycis CQ1] |
| novel-m0161-3p | NBO_586gi001 | valyl trna synthetase [Nosema bombycis CQ1] |
| novel-m0161-3p | NBO_63g0020 | Glutamate synthase NADPH small chain [Nosema bombycis CQ1] |
| novel-m0161-3p | NBO_6g0062 | Ricin B lectin [Nosema bombycis CQ1] |
| novel-m0170-5p | NBO_10g0037 | hypothetical protein NBO_10g0037 [Nosema bombycis CQ1] |
| novel-m0170-5p | NBO_12g0014 | ribosome biogenesis regulatory protein [Nosema ceranae] |
| novel-m0170-5p | NBO_480g0001 | hypothetical protein NBO_480g0001 [Nosema bombycis CQ1] |
| novel-m0170-5p | NBO_63g0020 | Glutamate synthase NADPH small chain [Nosema bombycis CQ1] |
| novel-m0197-3p | NBO_27g0019 | hypothetical protein NBO_27g0019, partial [Nosema bombycis CQ1] |
| novel-m0197-3p | NBO_586gi001 | valyl trna synthetase [Nosema bombycis CQ1] |
| novel-m0197-3p | NBO_63g0020 | Glutamate synthase NADPH small chain [Nosema bombycis CQ1] |
| novel-m0197-3p | NBO_6g0062 | Ricin B lectin [Nosema bombycis CQ1] |
| novel-m0199-5p | NBO_1203g0001 | hypothetical protein NBO_1203g0001 [Nosema bombycis CQ1] |
| novel-m0199-5p | NBO_41g0024 | Vacuolar amino acid transporter 5 [Nosema bombycis CQ1] |
| novel-m0199-5p | NBO_54g0012 | hypothetical protein NBO_54g0012 [Nosema bombycis CQ1] |
| novel-m0025-3p | NBO_10g0053 | DNA mismatch repair protein Mlh1 [Nosema bombycis CQ1] |
| novel-m0025-3p | NBO_13g0067 | f-box domain-containing protein [Nosema ceranae] |
| novel-m0025-3p | NBO_16g0027 | Heat shock 70 kDa protein cognate 4 [Nosema bombycis CQ1] |
| novel-m0025-3p | NBO_338g0001 | hypothetical protein NBO_338g0001 [Nosema bombycis CQ1] |
| novel-m0025-3p | NBO_375g0010 | 2,3-bisphosphoglycerate-independent phosphoglycerate mutase [Nosema bombycis CQ1] |
| novel-m0025-3p | NBO_3g0004 | hypothetical protein NBO_3g0004 [Nosema bombycis CQ1] |
| novel-m0025-3p | NBO_53g0003 | hypothetical protein NBO_53g0003 [Nosema bombycis CQ1] |
| novel-m0025-3p | NBO_55g0006 | Cyclin-A1-2, partial [Nosema bombycis CQ1] |
| novel-m0025-3p | NBO_62g0012 | hypothetical protein NBO_62g0012 [Nosema bombycis CQ1] |
| novel-m0025-3p | NBO_63g0026 | hypothetical spore wall protein [Nosema bombycis] |
| novel-m0025-3p | NBO_64gi001 | tRNA adenilyl transferase [Nosema bombycis CQ1] |
| novel-m0025-3p | NBO_76g0012 | type 2 peptidyl-tRNA hydrolase [Encephalitozoon romaleae SJ-2008] |
| novel-m0025-3p | NBO_85g0011 | Solute carrier family 2, facilitated glucose transporter member 2 [Nosema bombycis CQ1] |
| novel-m0061-3p | NBO_16g0028 | hypothetical protein NBO_16g0028 [Nosema bombycis CQ1] |
| novel-m0061-3p | NBO_32g0021 | hypothetical protein NBO_32g0021 [Nosema bombycis CQ1] |
| novel-m0061-3p | NBO_4g0050 | Insulin-degrading enzyme [Nosema bombycis CQ1] |
| novel-m0061-3p | NBO_63g0006 | Triosephosphate isomerase [Nosema bombycis CQ1] |
| novel-m0091-5p | NBO_1332gi002 | threonyl-tRNA synthetase [Nosema bombycis CQ1] |
| novel-m0091-5p | NBO_13g0057 | transcription factor TAU-like protein [Nosema bombycis CQ1] |
| novel-m0091-5p | NBO_365g0001 | Threonyl-tRNA synthetase, partial [Nosema bombycis CQ1] |
| novel-m0091-5p | NBO_63g0025 | Serine/threonine-protein kinase ppk23 [Nosema bombycis CQ1] |
| novel-m0091-5p | NBO_671gi001 | hiv1-tat interacting protein [Nosema bombycis CQ1] |
| novel-m0091-5p | NBO_76gi004 | threonyl-tRNA synthetase [Nosema bombycis CQ1] |
| novel-m0091-5p | NBO_81g0012 | MADS domain containing protein, partial [Nosema bombycis CQ1] |
| novel-m0099-3p | NBO_380g0001 | hypothetical protein NBO_380g0001 [Nosema bombycis CQ1] |
| novel-m0099-3p | NBO_46g0007 | hypothetical protein NBO_380g0001 [Nosema bombycis CQ1] |
| novel-m0099-3p | NBO_937g0002 | Sec61beta [Nosema bombycis CQ1] |
| novel-m0138-3p | NBO_380g0001 | hypothetical protein NBO_380g0001 [Nosema bombycis CQ1] |
| novel-m0138-3p | NBO_46g0007 | hypothetical protein NBO_380g0001 [Nosema bombycis CQ1] |
| novel-m0138-3p | NBO_937g0002 | Sec61beta [Nosema bombycis CQ1] |
| novel-m0196-3p | NBO_1054gi001 | T complex protein 1 subunit beta [Nosema bombycis CQ1] |
| novel-m0196-3p | NBO_10g0007 | 3-hydroxy-3-methylglutaryl-coenzyme A reductase, partial [Nosema bombycis CQ1] |
| novel-m0196-3p | NBO_1116g0004 | hypothetical protein NBO_1116g0004 [Nosema bombycis CQ1] |
| novel-m0196-3p | NBO_111g0002 | Serine palmitoyltransferase 1 [Nosema bombycis CQ1] |
| novel-m0196-3p | NBO_11g0004 | hypothetical protein NBO_11g0004 [Nosema bombycis CQ1] |
| novel-m0196-3p | NBO_11g0032 | Peptidase S8 and S53, subtilisin, kexin, sedolisin [Nosema bombycis CQ1] |
| novel-m0196-3p | NBO_11g0046 | Xaa-Pro aminopeptidase 1 [Nosema bombycis CQ1] |
| novel-m0196-3p | NBO_1276g0001 | tRNA acetyltransferase TAN1 [Nematocida displodere] |
| novel-m0196-3p | NBO_1332gi002 | threonyl-tRNA synthetase [Nosema bombycis CQ1] |
| novel-m0196-3p | NBO_1345gi001 | enolase [Nosema bombycis CQ1] |
| novel-m0196-3p | NBO_13g0057 | transcription factor TAU-like protein [Nosema bombycis CQ1] |
| novel-m0196-3p | NBO_16g0041 | 40S ribosomal protein SA [Phytophthora nicotianae] |
| novel-m0196-3p | NBO_19g0026 | U3 small nucleolar ribonucleoprotein imp4 [Nosema bombycis CQ1] |
| novel-m0196-3p | NBO_27g0051 | hypothetical protein NBO_27g0051 [Nosema bombycis CQ1] |
| novel-m0196-3p | NBO_29g0027 | trehalose-phosphatase, partial [Nosema bombycis CQ1] |
| novel-m0196-3p | NBO_2g0058 | RpeA, partial [Nosema bombycis CQ1] |
| novel-m0196-3p | NBO_31gi002 | small-conductance mechanosensitive channel protein [Nosema ceranae] |
| novel-m0196-3p | NBO_389g0002 | hypothetical protein NBO_389g0002, partial [Nosema bombycis CQ1] |
| novel-m0196-3p | NBO_38g0002 | hypothetical protein NBO_38g0002 [Nosema bombycis CQ1] |
| novel-m0196-3p | NBO_39g0002 | hypothetical protein NBO_39g0002 [Nosema bombycis CQ1] |
| novel-m0196-3p | NBO_423g0003 | hypothetical protein NBO_423g0003 [Nosema bombycis CQ1] |
| novel-m0196-3p | NBO_546g0001 | hypothetical protein NBO_546g0001 [Nosema bombycis CQ1] |
| novel-m0196-3p | NBO_58g0009 | DNA repair protein RAD16 [Nosema bombycis CQ1] |
| novel-m0196-3p | NBO_64g0003 | Putative membrane protein ycf1 [Nosema bombycis CQ1] |
| novel-m0196-3p | NBO_67g0015 | hypothetical protein NBO_67g0015 [Nosema bombycis CQ1] |
| novel-m0196-3p | NBO_69g0015 | serine threonine protein kinase [Nosema ceranae] |
| novel-m0196-3p | NBO_6g0004 | hypothetical protein NBO_6g0004 [Nosema bombycis CQ1] |
| novel-m0196-3p | NBO_6g0062 | Ricin B lectin [Nosema bombycis CQ1] |
| novel-m0196-3p | NBO_73g0028 | hypothetical protein NBO_73g0028 [Nosema bombycis CQ1] |
| novel-m0196-3p | NBO_7g0005 | hypothetical protein NBO_7g0005 [Nosema bombycis CQ1] |
| novel-m0196-3p | NBO_845g0001 | hypothetical protein NBO_845g0001 [Nosema bombycis CQ1] |
| novel-m0196-3p | NBO_84g0004 | Glycerol-3-phosphate dehydrogenase, mitochondrial [Nosema bombycis CQ1] |
| novel-m0228-3p | NBO_1054gi001 | T complex protein 1 subunit beta [Nosema bombycis CQ1] |
| novel-m0228-3p | NBO_10g0007 | 3-hydroxy-3-methylglutaryl-coenzyme A reductase, partial [Nosema bombycis CQ1] |
| novel-m0228-3p | NBO_1116g0004 | hypothetical protein NBO_1116g0004 [Nosema bombycis CQ1] |
| novel-m0228-3p | NBO_111g0002 | Serine palmitoyltransferase 1 [Nosema bombycis CQ1] |
| novel-m0228-3p | NBO_11g0004 | hypothetical protein NBO_11g0004 [Nosema bombycis CQ1] |
| novel-m0228-3p | NBO_11g0032 | Peptidase S8 and S53, subtilisin, kexin, sedolisin [Nosema bombycis CQ1] |
| novel-m0228-3p | NBO_11g0046 | Xaa-Pro aminopeptidase 1 [Nosema bombycis CQ1] |
| novel-m0228-3p | NBO_1276g0001 | tRNA acetyltransferase TAN1 [Nematocida displodere] |
| novel-m0228-3p | NBO_1332gi002 | threonyl-tRNA synthetase [Nosema bombycis CQ1] |
| novel-m0228-3p | NBO_1345gi001 | enolase [Nosema bombycis CQ1] |
| novel-m0228-3p | NBO_13g0057 | transcription factor TAU-like protein [Nosema bombycis CQ1] |
| novel-m0228-3p | NBO_16g0041 | 40S ribosomal protein SA [Phytophthora nicotianae] |
| novel-m0228-3p | NBO_19g0026 | U3 small nucleolar ribonucleoprotein imp4 [Nosema bombycis CQ1] |
| novel-m0228-3p | NBO_27g0051 | hypothetical protein NBO_27g0051 [Nosema bombycis CQ1] |
| novel-m0228-3p | NBO_29g0027 | trehalose-phosphatase, partial [Nosema bombycis CQ1] |
| novel-m0228-3p | NBO_2g0058 | RpeA, partial [Nosema bombycis CQ1] |
| novel-m0228-3p | NBO_31gi002 | small-conductance mechanosensitive channel protein [Nosema ceranae] |
| novel-m0228-3p | NBO_389g0002 | hypothetical protein NBO_389g0002, partial [Nosema bombycis CQ1] |
| novel-m0228-3p | NBO_38g0002 | hypothetical protein NBO_38g0002 [Nosema bombycis CQ1] |
| novel-m0228-3p | NBO_39g0002 | hypothetical protein NBO_39g0002 [Nosema bombycis CQ1] |
| novel-m0228-3p | NBO_423g0003 | hypothetical protein NBO_423g0003 [Nosema bombycis CQ1] |
| novel-m0228-3p | NBO_546g0001 | hypothetical protein NBO_546g0001 [Nosema bombycis CQ1] |
| novel-m0228-3p | NBO_58g0009 | DNA repair protein RAD16 [Nosema bombycis CQ1] |
| novel-m0228-3p | NBO_64g0003 | Putative membrane protein ycf1 [Nosema bombycis CQ1] |
| novel-m0228-3p | NBO_67g0015 | hypothetical protein NBO_67g0015 [Nosema bombycis CQ1] |
| novel-m0228-3p | NBO_69g0015 | serine threonine protein kinase [Nosema ceranae] |
| novel-m0228-3p | NBO_6g0004 | hypothetical protein NBO_6g0004 [Nosema bombycis CQ1] |
| novel-m0228-3p | NBO_6g0062 | Ricin B lectin [Nosema bombycis CQ1] |
| novel-m0228-3p | NBO_73g0028 | hypothetical protein NBO_73g0028 [Nosema bombycis CQ1] |
| novel-m0228-3p | NBO_7g0005 | hypothetical protein NBO_7g0005 [Nosema bombycis CQ1] |
| novel-m0228-3p | NBO_845g0001 | hypothetical protein NBO_845g0001 [Nosema bombycis CQ1] |
| novel-m0228-3p | NBO_84g0004 | Glycerol-3-phosphate dehydrogenase, mitochondrial [Nosema bombycis CQ1] |
| novel-m0229-3p | NBO_1054gi001 | T complex protein 1 subunit beta [Nosema bombycis CQ1] |
| novel-m0229-3p | NBO_10g0007 | 3-hydroxy-3-methylglutaryl-coenzyme A reductase, partial [Nosema bombycis CQ1] |
| novel-m0229-3p | NBO_1116g0004 | hypothetical protein NBO_1116g0004 [Nosema bombycis CQ1] |
| novel-m0229-3p | NBO_111g0002 | Serine palmitoyltransferase 1 [Nosema bombycis CQ1] |
| novel-m0229-3p | NBO_11g0004 | hypothetical protein NBO_11g0004 [Nosema bombycis CQ1] |
| novel-m0229-3p | NBO_11g0032 | Peptidase S8 and S53, subtilisin, kexin, sedolisin [Nosema bombycis CQ1] |
| novel-m0229-3p | NBO_11g0046 | Xaa-Pro aminopeptidase 1 [Nosema bombycis CQ1] |
| novel-m0229-3p | NBO_1276g0001 | tRNA acetyltransferase TAN1 [Nematocida displodere] |
| novel-m0229-3p | NBO_1332gi002 | threonyl-tRNA synthetase [Nosema bombycis CQ1] |
| novel-m0229-3p | NBO_1345gi001 | enolase [Nosema bombycis CQ1] |
| novel-m0229-3p | NBO_13g0057 | transcription factor TAU-like protein [Nosema bombycis CQ1] |
| novel-m0229-3p | NBO_16g0041 | 40S ribosomal protein SA [Phytophthora nicotianae] |
| novel-m0229-3p | NBO_19g0026 | U3 small nucleolar ribonucleoprotein imp4 [Nosema bombycis CQ1] |
| novel-m0229-3p | NBO_27g0051 | hypothetical protein NBO_27g0051 [Nosema bombycis CQ1] |
| novel-m0229-3p | NBO_29g0027 | trehalose-phosphatase, partial [Nosema bombycis CQ1] |
| novel-m0229-3p | NBO_2g0058 | RpeA, partial [Nosema bombycis CQ1] |
| novel-m0229-3p | NBO_31gi002 | small-conductance mechanosensitive channel protein [Nosema ceranae] |
| novel-m0229-3p | NBO_389g0002 | hypothetical protein NBO_389g0002, partial [Nosema bombycis CQ1] |
| novel-m0229-3p | NBO_38g0002 | hypothetical protein NBO_38g0002 [Nosema bombycis CQ1] |
| novel-m0229-3p | NBO_39g0002 | hypothetical protein NBO_39g0002 [Nosema bombycis CQ1] |
| novel-m0229-3p | NBO_423g0003 | hypothetical protein NBO_423g0003 [Nosema bombycis CQ1] |
| novel-m0229-3p | NBO_546g0001 | hypothetical protein NBO_546g0001 [Nosema bombycis CQ1] |
| novel-m0229-3p | NBO_58g0009 | DNA repair protein RAD16 [Nosema bombycis CQ1] |
| novel-m0229-3p | NBO_64g0003 | Putative membrane protein ycf1 [Nosema bombycis CQ1] |
| novel-m0229-3p | NBO_67g0015 | hypothetical protein NBO_67g0015 [Nosema bombycis CQ1] |
| novel-m0229-3p | NBO_69g0015 | serine threonine protein kinase [Nosema ceranae] |
| novel-m0229-3p | NBO_6g0004 | hypothetical protein NBO_6g0004 [Nosema bombycis CQ1] |
| novel-m0229-3p | NBO_6g0062 | Ricin B lectin [Nosema bombycis CQ1] |
| novel-m0229-3p | NBO_73g0028 | hypothetical protein NBO_73g0028 [Nosema bombycis CQ1] |
| novel-m0229-3p | NBO_7g0005 | hypothetical protein NBO_7g0005 [Nosema bombycis CQ1] |
| novel-m0229-3p | NBO_845g0001 | hypothetical protein NBO_845g0001 [Nosema bombycis CQ1] |
| novel-m0229-3p | NBO_84g0004 | Glycerol-3-phosphate dehydrogenase, mitochondrial [Nosema bombycis CQ1] |
| novel-m0003-3p | NBO_1024g0001 | DNA-directed RNA polymerase II subunit RPB3 [Nosema bombycis CQ1] |
| novel-m0003-3p | NBO_1039g0001 | Proteasome component PUP1 [Nosema bombycis CQ1] |
| novel-m0003-3p | NBO_108g0004 | Heat shock protein 90, partial [Nosema bombycis CQ1] |
| novel-m0003-3p | NBO_1230g0001 | hypothetical protein NBO_1230g0001 [Nosema bombycis CQ1] |
| novel-m0003-3p | NBO_16g0013 | Structural maintenance of chromosomes protein 4, partial [Nosema bombycis CQ1] |
| novel-m0003-3p | NBO_16g0051 | hypothetical protein NBO_16g0051 [Nosema bombycis CQ1] |
| novel-m0003-3p | NBO_26g0013 | hypothetical spore wall protein [Nosema bombycis] |
| novel-m0003-3p | NBO_27g0006 | integral membrane [Nosema apis BRL 01] |
| novel-m0003-3p | NBO_27g0025 | skt5-like protein [Nosema apis BRL 01] |
| novel-m0003-3p | NBO_28g0019 | DNA-directed RNA polymerases I and III subunit RPAC1 [Nosema bombycis CQ1] |
| novel-m0003-3p | NBO_366g0002 | Heat shock protein HSP 90-alpha 1 [Nosema bombycis CQ1] |
| novel-m0003-3p | NBO_374g0007 | Glucosamine--fructose-6-phosphate aminotransferase 1 [Nosema bombycis CQ1] |
| novel-m0003-3p | NBO_377g0003 | Heat shock 70 kDa protein 6 [Nosema bombycis CQ1] |
| novel-m0003-3p | NBO_38g0009 | hypothetical protein NBO_38g0009 [Nosema bombycis CQ1] |
| novel-m0003-3p | NBO_460g0011 | hypothetical protein NBO_460g0011 [Nosema bombycis CQ1] |
| novel-m0003-3p | NBO_490g0001 | hypothetical protein NBO_490g0001 [Nosema bombycis CQ1] |
| novel-m0003-3p | NBO_72g0014 | hypothetical protein NBO_72g0014 [Nosema bombycis CQ1] |
| novel-m0003-3p | NBO_740g0001 | ribosomal protein l12e-l44-l45-rpp1-rpp2 [Nosema apis BRL 01] |
| novel-m0003-3p | NBO_78g0012 | ATP-binding cassette sub-family B member 7, mitochondrial [Nosema bombycis CQ1] |
| novel-m0003-3p | NBO_80g0024 | Major facilitator superfamily domain-containing protein 1 [Nosema bombycis CQ1] |
| novel-m0003-3p | NBO_841g0001 | hypothetical protein NBO_841g0001 [Nosema bombycis CQ1] |
| novel-m0003-3p | NBO_979g0001 | transcription initiation factor brf1 subunit-like protein [Nosema ceranae] |
| novel-m0010-5p | NBO_28g0026 | hypothetical protein NBO_28g0026 [Nosema bombycis CQ1] |
| novel-m0010-5p | NBO_34g0043 | Serine/threonine-protein phosphatase 4 catalytic subunit [Nosema bombycis CQ1] |
| novel-m0010-5p | NBO_389g0002 | hypothetical protein NBO_389g0002, partial [Nosema bombycis CQ1] |
| novel-m0010-5p | NBO_4g0009 | 3-ketodihydrosphingosine reductase [Nosema bombycis CQ1] |
| novel-m0010-5p | NBO_549g0007 | DNA polymerase epsilon catalytic subunit A [Nosema bombycis CQ1] |
| novel-m0010-5p | NBO_560g0001 | DNA-binding protein SMUBP-2 [Nosema bombycis CQ1] |
| novel-m0010-5p | NBO_64gi001 | tRNA adenilyl transferase [Nosema bombycis CQ1] |
| novel-m0010-5p | NBO_671gi001 | hiv1-tat interacting protein [Nosema bombycis CQ1] |
| novel-m0015-3p | NBO_1024g0001 | DNA-directed RNA polymerase II subunit RPB3 [Nosema bombycis CQ1] |
| novel-m0015-3p | NBO_1140g0001 | Synaptobrevin-like protein [Nosema bombycis CQ1] |
| novel-m0015-3p | NBO_11gi001 | actin-like 53kDa protein [Nosema bombycis CQ1] |
| novel-m0015-3p | NBO_15g0005 | negative regulator of transcription [Encephalitozoon cuniculi GB-M1] |
| novel-m0015-3p | NBO_24g0003 | hypothetical protein NBO_24g0003 [Nosema bombycis CQ1] |
| novel-m0015-3p | NBO_34g0043 | Serine/threonine-protein phosphatase 4 catalytic subunit [Nosema bombycis CQ1] |
| novel-m0015-3p | NBO_362g0011 | CDP-diacylglycerol--inositol 3-phosphatidyltransferase [Nosema bombycis CQ1] |
| novel-m0015-3p | NBO_616g0003 | Heat shock factor protein 1 [Nosema bombycis CQ1] |
| novel-m0015-3p | NBO_9g0005 | 60S ribosomal protein L10 [Nosema bombycis] |
| novel-m0065-3p | NBO_16g0013 | Structural maintenance of chromosomes protein 4, partial [Nosema bombycis CQ1] |
| novel-m0065-3p | NBO_2g0012 | Thymidine kinase [Nosema bombycis CQ1] |
| novel-m0065-3p | NBO_34g0043 | Serine/threonine-protein phosphatase 4 catalytic subunit [Nosema bombycis CQ1] |
| novel-m0065-3p | NBO_362g0011 | CDP-diacylglycerol--inositol 3-phosphatidyltransferase [Nosema bombycis CQ1] |
| novel-m0065-3p | NBO_411gi001 | GTP-binding protein [Nosema bombycis CQ1] |
| novel-m0065-3p | NBO_41g0019 | eukaryotic translation initiation factor 4a [Nosema ceranae] |
| novel-m0065-3p | NBO_423g0006 | Iron-sulfur clusters transporter atm1, mitochondrial, partial [Nosema bombycis CQ1] |
| novel-m0065-3p | NBO_444g0002 | Chaperone protein dnaJ [Nosema bombycis CQ1] |
| novel-m0065-3p | NBO_520g0003 | N(2),N(2)-dimethylguanosine tRNA methyltransferase [Nosema bombycis CQ1] |
| novel-m0065-3p | NBO_58g0010 | Chitin synthase 7 [Nosema bombycis CQ1] |
| novel-m0065-3p | NBO_69g0004 | DNA polymerase alpha subunit B, partial [Nosema bombycis CQ1] |
| novel-m0065-3p | NBO_9g0005 | 60S ribosomal protein L10 [Nosema bombycis] |
| novel-m0072-5p | NBO_10g0052 | Polar tube protein 3 [Nosema bombycis CQ1] |
| novel-m0072-5p | NBO_1136g0001 | ricin b lectin [Nosema ceranae] |
| novel-m0072-5p | NBO_114g0004 | Zinc phosphodiesterase ELAC protein 2 [Nosema bombycis CQ1] |
| novel-m0072-5p | NBO_13g0046 | hypothetical protein NBO_13g0046 [Nosema bombycis CQ1] |
| novel-m0072-5p | NBO_13g0067 | f-box domain-containing protein [Nosema ceranae] |
| novel-m0072-5p | NBO_166g0002 | Transcription-associated protein 1, partial [Nosema bombycis CQ1] |
| novel-m0072-5p | NBO_16g0013 | Structural maintenance of chromosomes protein 4, partial [Nosema bombycis CQ1] |
| novel-m0072-5p | NBO_20g0001 | exocyst complex subunit sec6 [Nosema apis BRL 01] |
| novel-m0072-5p | NBO_28g0056 | hypothetical protein NBO_28g0056 [Nosema bombycis CQ1] |
| novel-m0072-5p | NBO_29g0021 | Spore wall and anchoring disk complex protein [Nosema bombycis CQ1] |
| novel-m0072-5p | NBO_32g0048 | Elongation factor 1-alpha [Nosema bombycis CQ1] |
| novel-m0072-5p | NBO_33g0018 | 78 kDa glucose-regulated protein [Nosema bombycis CQ1] |
| novel-m0072-5p | NBO_378g0002 | hypothetical protein NBO_378g0002 [Nosema bombycis CQ1] |
| novel-m0072-5p | NBO_4g0029 | polar tube protein 3 [Nosema bombycis] |
| novel-m0072-5p | NBO_4g0042 | TBC1 domain family member 13 [Nosema bombycis CQ1] |
| novel-m0072-5p | NBO_4g0050 | Insulin-degrading enzyme [Nosema bombycis CQ1] |
| novel-m0072-5p | NBO_552g0005 | surface-antigen protein P30.4, partial [Nosema bombycis] |
| novel-m0072-5p | NBO_568g0002 | Spore wall and anchoring disk complex protein [Nosema bombycis CQ1] |
| novel-m0072-5p | NBO_60g0017 | Transcription-associated protein 1 [Nosema bombycis CQ1] |
| novel-m0072-5p | NBO_6g0040 | hypothetical protein NBO_6g0040 [Nosema bombycis CQ1] |
| novel-m0072-5p | NBO_7g0005 | hypothetical protein NBO_7g0005 [Nosema bombycis CQ1] |
| novel-m0072-5p | NBO_7g0040 | hypothetical protein NBO_7g0040, partial [Nosema bombycis CQ1] |
| novel-m0104-3p | NBO_1078g0004 | Zinc finger C2H2 protein [Nosema bombycis CQ1] |
| novel-m0104-3p | NBO_1168gi001 | SCF ubiquitin ligase and anaphase-promoting complex protein [Encephalitozoon intestinalis ATCC 50506] |
| novel-m0104-3p | NBO_11g0004 | hypothetical protein NBO_11g0004 [Nosema bombycis CQ1] |
| novel-m0104-3p | NBO_1271g0002 | hypothetical protein NBO_1271g0002 [Nosema bombycis CQ1] |
| novel-m0104-3p | NBO_1291g0001 | Deoxyribodipyrimidine photo-lyase [Nosema bombycis CQ1] |
| novel-m0104-3p | NBO_1314g0001 | hypothetical protein NBO_1314g0001 [Nosema bombycis CQ1] |
| novel-m0104-3p | NBO_1320g0001 | Hexokinase-2 [Nosema bombycis CQ1] |
| novel-m0104-3p | NBO_149g0002 | Glutamate-cysteine ligase, partial [Nosema bombycis CQ1] |
| novel-m0104-3p | NBO_16g0010 | 2Fe-2S ferredoxin [Nosema bombycis CQ1] |
| novel-m0104-3p | NBO_16g0013 | Structural maintenance of chromosomes protein 4, partial [Nosema bombycis CQ1] |
| novel-m0104-3p | NBO_204g0002 | hypothetical protein NBO_204g0002 [Nosema bombycis CQ1] |
| novel-m0104-3p | NBO_22g0008 | ormdl family protein [Nosema ceranae] |
| novel-m0104-3p | NBO_24g0011 | hypothetical protein NBO_24g0011 [Nosema bombycis CQ1] |
| novel-m0104-3p | NBO_27g0024 | Chitin synthase regulatory factor 4 [Nosema bombycis CQ1] |
| novel-m0104-3p | NBO_27g0035 | spore wall protein 5 [Nosema bombycis] |
| novel-m0104-3p | NBO_28g0012 | Transcription elongation factor SPT4 [Nosema bombycis CQ1] |
| novel-m0104-3p | NBO_2g0071 | hypothetical protein NBO_2g0071 [Nosema bombycis CQ1] |
| novel-m0104-3p | NBO_31gi002 | small-conductance mechanosensitive channel protein [Nosema ceranae] |
| novel-m0104-3p | NBO_32g0024 | General transcriptional corepressor ssn6 [Nosema bombycis CQ1] |
| novel-m0104-3p | NBO_34g0043 | Serine/threonine-protein phosphatase 4 catalytic subunit [Nosema bombycis CQ1] |
| novel-m0104-3p | NBO_377g0003 | Heat shock 70 kDa protein 6 [Nosema bombycis CQ1] |
| novel-m0104-3p | NBO_384g0005 | hypothetical protein NBO_384g0005 [Nosema bombycis CQ1] |
| novel-m0104-3p | NBO_389g0002 | hypothetical protein NBO_389g0002, partial [Nosema bombycis CQ1] |
| novel-m0104-3p | NBO_38g0002 | hypothetical protein NBO_38g0002 [Nosema bombycis CQ1] |
| novel-m0104-3p | NBO_3g0006 | 60S ribosomal protein L6, partial [Nosema bombycis] |
| novel-m0104-3p | NBO_419g0005 | hypothetical protein NBO_419g0005 [Nosema bombycis CQ1] |
| novel-m0104-3p | NBO_423g0001 | NADPH-dependent diflavin oxidoreductase 1 [Nosema bombycis CQ1] |
| novel-m0104-3p | NBO_429g0002 | hypothetical protein NBO_429g0002 [Nosema bombycis CQ1] |
| novel-m0104-3p | NBO_48g0007 | hypothetical protein NBO_48g0007 [Nosema bombycis CQ1] |
| novel-m0104-3p | NBO_546g0001 | hypothetical protein NBO_546g0001 [Nosema bombycis CQ1] |
| novel-m0104-3p | NBO_549g0007 | DNA polymerase epsilon catalytic subunit A [Nosema bombycis CQ1] |
| novel-m0104-3p | NBO_54g0012 | hypothetical protein NBO_54g0012 [Nosema bombycis CQ1] |
| novel-m0104-3p | NBO_552g0002 | spore wall protein 5 [Nosema bombycis] |
| novel-m0104-3p | NBO_55g0012 | hypothetical protein NBO_55g0012 [Nosema bombycis CQ1] |
| novel-m0104-3p | NBO_633g0001 | DNA mismatch repair protein mutS [Nosema bombycis CQ1] |
| novel-m0104-3p | NBO_63g0005 | Triosephosphate isomerase [Nosema bombycis CQ1] |
| novel-m0104-3p | NBO_63g0006 | Triosephosphate isomerase [Nosema bombycis CQ1] |
| novel-m0104-3p | NBO_64g0003 | Putative membrane protein ycf1 [Nosema bombycis CQ1] |
| novel-m0104-3p | NBO_69g0004 | DNA polymerase alpha subunit B, partial [Nosema bombycis CQ1] |
| novel-m0104-3p | NBO_71g0002 | isoleucyl-tRNA synthetase, cytoplasmic [Nosema bombycis CQ1] |
| novel-m0104-3p | NBO_76g0003 | hypothetical protein NBO_76g0003 [Nosema bombycis CQ1] |
| novel-m0104-3p | NBO_78g0013 | hypothetical protein NBO_78g0013 [Nosema bombycis CQ1] |
| novel-m0104-3p | NBO_81g0012 | MADS domain containing protein, partial [Nosema bombycis CQ1] |
| novel-m0104-3p | NBO_841g0001 | hypothetical protein NBO_841g0001 [Nosema bombycis CQ1] |
| novel-m0104-3p | NBO_9g0004 | hypothetical protein NBO_9g0004 [Nosema bombycis CQ1] |
| novel-m0104-5p | NBO_1054gi001 | T complex protein 1 subunit beta [Nosema bombycis CQ1] |
| novel-m0104-5p | NBO_1552gi001 | poly(A) polymerase type 1, partial [Nosema bombycis CQ1] |
| novel-m0104-5p | NBO_16g0010 | 2Fe-2S ferredoxin [Nosema bombycis CQ1] |
| novel-m0104-5p | NBO_6g0015 | Ricin B lectin [Nosema bombycis CQ1] |
| novel-m0118-3p | NBO_1024g0001 | DNA-directed RNA polymerase II subunit RPB3 [Nosema bombycis CQ1] |
| novel-m0118-3p | NBO_1039g0001 | Proteasome component PUP1 [Nosema bombycis CQ1] |
| novel-m0118-3p | NBO_108g0004 | Heat shock protein 90, partial [Nosema bombycis CQ1] |
| novel-m0118-3p | NBO_1230g0001 | hypothetical protein NBO_1230g0001 [Nosema bombycis CQ1] |
| novel-m0118-3p | NBO_16g0013 | Structural maintenance of chromosomes protein 4, partial [Nosema bombycis CQ1] |
| novel-m0118-3p | NBO_16g0051 | hypothetical protein NBO_16g0051 [Nosema bombycis CQ1] |
| novel-m0118-3p | NBO_26g0013 | hypothetical spore wall protein [Nosema bombycis] |
| novel-m0118-3p | NBO_27g0006 | integral membrane [Nosema apis BRL 01] |
| novel-m0118-3p | NBO_27g0025 | skt5-like protein [Nosema apis BRL 01] |
| novel-m0118-3p | NBO_28g0019 | DNA-directed RNA polymerases I and III subunit RPAC1 [Nosema bombycis CQ1] |
| novel-m0118-3p | NBO_366g0002 | Heat shock protein HSP 90-alpha 1 [Nosema bombycis CQ1] |
| novel-m0118-3p | NBO_374g0007 | Glucosamine--fructose-6-phosphate aminotransferase 1 [Nosema bombycis CQ1] |
| novel-m0118-3p | NBO_377g0003 | Heat shock 70 kDa protein 6 [Nosema bombycis CQ1] |
| novel-m0118-3p | NBO_38g0009 | hypothetical protein NBO_38g0009 [Nosema bombycis CQ1] |
| novel-m0118-3p | NBO_460g0011 | hypothetical protein NBO_460g0011 [Nosema bombycis CQ1] |
| novel-m0118-3p | NBO_490g0001 | hypothetical protein NBO_490g0001 [Nosema bombycis CQ1] |
| novel-m0118-3p | NBO_72g0014 | hypothetical protein NBO_72g0014 [Nosema bombycis CQ1] |
| novel-m0118-3p | NBO_740g0001 | ribosomal protein l12e-l44-l45-rpp1-rpp2 [Nosema apis BRL 01] |
| novel-m0118-3p | NBO_78g0012 | ATP-binding cassette sub-family B member 7, mitochondrial [Nosema bombycis CQ1] |
| novel-m0118-3p | NBO_80g0024 | Major facilitator superfamily domain-containing protein 1 [Nosema bombycis CQ1] |
| novel-m0118-3p | NBO_841g0001 | hypothetical protein NBO_841g0001 [Nosema bombycis CQ1] |
| novel-m0118-3p | NBO_979g0001 | transcription initiation factor brf1 subunit-like protein [Nosema ceranae] |
| novel-m0174-5p | NBO_1010g0001 | Pescadillo-like protein [Nosema bombycis CQ1] |
| novel-m0174-5p | NBO_1078g0004 | Zinc finger C2H2 protein [Nosema bombycis CQ1] |
| novel-m0174-5p | NBO_1170gi001 | HSP 101 related protein, partial [Nosema bombycis CQ1] |
| novel-m0174-5p | NBO_11g0003 | UTP-glucose-1-phosphate uridylyltransferase [Nosema bombycis CQ1] |
| novel-m0174-5p | NBO_11g0038 | Protein kinase kin1 [Nosema bombycis CQ1] |
| novel-m0174-5p | NBO_1230gi001 | hypothetical protein NBO_1230gi001 [Nosema bombycis CQ1] |
| novel-m0174-5p | NBO_1232g0001 | hypothetical protein NBO_1232g0001 [Nosema bombycis CQ1] |
| novel-m0174-5p | NBO_1271g0002 | hypothetical protein NBO_1271g0002 [Nosema bombycis CQ1] |
| novel-m0174-5p | NBO_1302g0001 | hypothetical protein NBO_1302g0001 [Nosema bombycis CQ1] |
| novel-m0174-5p | NBO_13g0057 | transcription factor TAU-like protein [Nosema bombycis CQ1] |
| novel-m0174-5p | NBO_13g0067 | f-box domain-containing protein [Nosema ceranae] |
| novel-m0174-5p | NBO_19g0014 | hypothetical protein NBO_19g0014 [Nosema bombycis CQ1] |
| novel-m0174-5p | NBO_20g0002 | Pescadillo like protein [Nosema bombycis CQ1] |
| novel-m0174-5p | NBO_32g0024 | General transcriptional corepressor ssn6 [Nosema bombycis CQ1] |
| novel-m0174-5p | NBO_32g0025 | DNA-directed RNA polymerase I subunit RPA12 [Nosema bombycis CQ1] |
| novel-m0174-5p | NBO_375g0009 | 2,3-bisphosphoglycerate-independent phosphoglycerate mutase [Nosema bombycis CQ1] |
| novel-m0174-5p | NBO_389g0002 | hypothetical protein NBO_389g0002, partial [Nosema bombycis CQ1] |
| novel-m0174-5p | NBO_38g0012 | hypothetical protein NBO_38g0012 [Nosema bombycis CQ1] |
| novel-m0174-5p | NBO_3g0004 | hypothetical protein NBO_3g0004 [Nosema bombycis CQ1] |
| novel-m0174-5p | NBO_43g0006 | ATP-dependent RNA helicase DHX37 [Nosema bombycis CQ1] |
| novel-m0174-5p | NBO_468g0001 | hypothetical protein NBO_468g0001 [Nosema bombycis CQ1] |
| novel-m0174-5p | NBO_48g0004 | mevalonate kinase [Nosema bombycis CQ1] |
| novel-m0174-5p | NBO_490g0001 | hypothetical protein NBO_490g0001 [Nosema bombycis CQ1] |
| novel-m0174-5p | NBO_499g0002 | SKP1 component [Nosema bombycis CQ1] |
| novel-m0174-5p | NBO_49g0001 | hypothetical protein NBO_49g0001 [Nosema bombycis CQ1] |
| novel-m0174-5p | NBO_4g0017 | Heat shock protein 101 [Nosema bombycis CQ1] |
| novel-m0174-5p | NBO_4g0031 | elongation factor 2 [Nosema bombycis CQ1] |
| novel-m0174-5p | NBO_4g0050 | Insulin-degrading enzyme [Nosema bombycis CQ1] |
| novel-m0174-5p | NBO_54g0012 | hypothetical protein NBO_54g0012 [Nosema bombycis CQ1] |
| novel-m0174-5p | NBO_60g0027 | histone-binding protein N1/N2 [Nosema bombycis CQ1] |
| novel-m0174-5p | NBO_63g0018 | hypothetical protein NBO_63g0018 [Nosema bombycis CQ1] |
| novel-m0174-5p | NBO_63g0026 | hypothetical spore wall protein [Nosema bombycis] |
| novel-m0174-5p | NBO_64g0028 | E3 ubiquitin-protein ligase [Nosema bombycis CQ1] |
| novel-m0174-5p | NBO_69g0015 | serine threonine protein kinase [Nosema ceranae] |
| novel-m0174-5p | NBO_6g0004 | hypothetical protein NBO_6g0004 [Nosema bombycis CQ1] |
| novel-m0174-5p | NBO_6g0033 | NEDD8-conjugating enzyme UBC12 [Nosema bombycis CQ1] |
| novel-m0174-5p | NBO_80g0034 | Zinc finger C2H2 protein [Nosema bombycis CQ1] |
| novel-m0174-5p | NBO_84g0004 | Glycerol-3-phosphate dehydrogenase, mitochondrial [Nosema bombycis CQ1] |
| novel-m0174-5p | NBO_8g0045 | signal peptidase like protein [Nosema bombycis CQ1] |
| novel-m0174-5p | NBO_915g0002 | Cell division control protein 48 E, partial [Nosema bombycis CQ1] |
| novel-m0204-3p | NBO_1024g0001 | DNA-directed RNA polymerase II subunit RPB3 [Nosema bombycis CQ1] |
| novel-m0204-3p | NBO_1140g0001 | Synaptobrevin-like protein [Nosema bombycis CQ1] |
| novel-m0204-3p | NBO_11gi001 | actin-like 53kDa protein [Nosema bombycis CQ1] |
| novel-m0204-3p | NBO_15g0005 | negative regulator of transcription [Encephalitozoon cuniculi GB-M1] |
| novel-m0204-3p | NBO_24g0003 | hypothetical protein NBO_24g0003 [Nosema bombycis CQ1] |
| novel-m0204-3p | NBO_34g0043 | Serine/threonine-protein phosphatase 4 catalytic subunit [Nosema bombycis CQ1] |
| novel-m0204-3p | NBO_362g0011 | CDP-diacylglycerol--inositol 3-phosphatidyltransferase [Nosema bombycis CQ1] |
| novel-m0204-3p | NBO_616g0003 | Heat shock factor protein 1 [Nosema bombycis CQ1] |
| novel-m0204-3p | NBO_9g0005 | 60S ribosomal protein L10 [Nosema bombycis] |
| novel-m0206-5p | NBO_10g0052 | Polar tube protein 3 [Nosema bombycis CQ1] |
| novel-m0206-5p | NBO_10g0110 | DNA-directed RNA polymerases I and III subunit RPAC2 [Nosema bombycis CQ1] |
| novel-m0206-5p | NBO_1314g0001 | hypothetical protein NBO_1314g0001 [Nosema bombycis CQ1] |
| novel-m0206-5p | NBO_13g0072 | hypothetical protein NBO_13g0072 [Nosema bombycis CQ1] |
| novel-m0206-5p | NBO_16g0013 | Structural maintenance of chromosomes protein 4, partial [Nosema bombycis CQ1] |
| novel-m0206-5p | NBO_19g0014 | hypothetical protein NBO_19g0014 [Nosema bombycis CQ1] |
| novel-m0206-5p | NBO_28g0019 | DNA-directed RNA polymerases I and III subunit RPAC1 [Nosema bombycis CQ1] |
| novel-m0206-5p | NBO_2gi003 | DNA-directed RNA polymerases I and III subunit RPAC2 [Nosema bombycis CQ1] |
| novel-m0206-5p | NBO_3g0041 | DNA-directed RNA polymerase III subunit RPC6 [Nosema bombycis CQ1] |
| novel-m0206-5p | NBO_423g0001 | NADPH-dependent diflavin oxidoreductase 1 [Nosema bombycis CQ1] |
| novel-m0206-5p | NBO_483g0001 | hypothetical protein NBO_483g0001 [Nosema bombycis CQ1] |
| novel-m0206-5p | NBO_4g0029 | polar tube protein 3 [Nosema bombycis] |
| novel-m0211-3p | NBO_16g0013 | Structural maintenance of chromosomes protein 4, partial [Nosema bombycis CQ1] |
| novel-m0211-3p | NBO_2g0012 | Thymidine kinase [Nosema bombycis CQ1] |
| novel-m0211-3p | NBO_34g0043 | Serine/threonine-protein phosphatase 4 catalytic subunit [Nosema bombycis CQ1] |
| novel-m0211-3p | NBO_362g0011 | CDP-diacylglycerol--inositol 3-phosphatidyltransferase [Nosema bombycis CQ1] |
| novel-m0211-3p | NBO_411gi001 | GTP-binding protein [Nosema bombycis CQ1] |
| novel-m0211-3p | NBO_41g0019 | eukaryotic translation initiation factor 4a [Nosema ceranae] |
| novel-m0211-3p | NBO_423g0006 | Iron-sulfur clusters transporter atm1, mitochondrial, partial [Nosema bombycis CQ1] |
| novel-m0211-3p | NBO_444g0002 | Chaperone protein dnaJ [Nosema bombycis CQ1] |
| novel-m0211-3p | NBO_520g0003 | N(2),N(2)-dimethylguanosine tRNA methyltransferase [Nosema bombycis CQ1] |
| novel-m0211-3p | NBO_58g0010 | Chitin synthase 7 [Nosema bombycis CQ1] |
| novel-m0211-3p | NBO_69g0004 | DNA polymerase alpha subunit B, partial [Nosema bombycis CQ1] |
| novel-m0211-3p | NBO_9g0005 | 60S ribosomal protein L10 [Nosema bombycis] |
| novel-m0212-3p | NBO_1078g0004 | Zinc finger C2H2 protein [Nosema bombycis CQ1] |
| novel-m0212-3p | NBO_1168gi001 | SCF ubiquitin ligase and anaphase-promoting complex protein [Encephalitozoon intestinalis ATCC 50506] |
| novel-m0212-3p | NBO_11g0004 | hypothetical protein NBO_11g0004 [Nosema bombycis CQ1] |
| novel-m0212-3p | NBO_1271g0002 | hypothetical protein NBO_1271g0002 [Nosema bombycis CQ1] |
| novel-m0212-3p | NBO_1291g0001 | Deoxyribodipyrimidine photo-lyase [Nosema bombycis CQ1] |
| novel-m0212-3p | NBO_1314g0001 | hypothetical protein NBO_1314g0001 [Nosema bombycis CQ1] |
| novel-m0212-3p | NBO_1320g0001 | Hexokinase-2 [Nosema bombycis CQ1] |
| novel-m0212-3p | NBO_149g0002 | Glutamate-cysteine ligase, partial [Nosema bombycis CQ1] |
| novel-m0212-3p | NBO_16g0010 | 2Fe-2S ferredoxin [Nosema bombycis CQ1] |
| novel-m0212-3p | NBO_16g0013 | Structural maintenance of chromosomes protein 4, partial [Nosema bombycis CQ1] |
| novel-m0212-3p | NBO_204g0002 | hypothetical protein NBO_204g0002 [Nosema bombycis CQ1] |
| novel-m0212-3p | NBO_22g0008 | ormdl family protein [Nosema ceranae] |
| novel-m0212-3p | NBO_24g0011 | hypothetical protein NBO_24g0011 [Nosema bombycis CQ1] |
| novel-m0212-3p | NBO_27g0024 | Chitin synthase regulatory factor 4 [Nosema bombycis CQ1] |
| novel-m0212-3p | NBO_27g0035 | spore wall protein 5 [Nosema bombycis] |
| novel-m0212-3p | NBO_28g0012 | Transcription elongation factor SPT4 [Nosema bombycis CQ1] |
| novel-m0212-3p | NBO_2g0071 | hypothetical protein NBO_2g0071 [Nosema bombycis CQ1] |
| novel-m0212-3p | NBO_31gi002 | small-conductance mechanosensitive channel protein [Nosema ceranae] |
| novel-m0212-3p | NBO_32g0024 | General transcriptional corepressor ssn6 [Nosema bombycis CQ1] |
| novel-m0212-3p | NBO_34g0043 | Serine/threonine-protein phosphatase 4 catalytic subunit [Nosema bombycis CQ1] |
| novel-m0212-3p | NBO_377g0003 | Heat shock 70 kDa protein 6 [Nosema bombycis CQ1] |
| novel-m0212-3p | NBO_384g0005 | hypothetical protein NBO_384g0005 [Nosema bombycis CQ1] |
| novel-m0212-3p | NBO_389g0002 | hypothetical protein NBO_389g0002, partial [Nosema bombycis CQ1] |
| novel-m0212-3p | NBO_38g0002 | hypothetical protein NBO_38g0002 [Nosema bombycis CQ1] |
| novel-m0212-3p | NBO_3g0006 | 60S ribosomal protein L6, partial [Nosema bombycis] |
| novel-m0212-3p | NBO_419g0005 | hypothetical protein NBO_419g0005 [Nosema bombycis CQ1] |
| novel-m0212-3p | NBO_423g0001 | NADPH-dependent diflavin oxidoreductase 1 [Nosema bombycis CQ1] |
| novel-m0212-3p | NBO_429g0002 | hypothetical protein NBO_429g0002 [Nosema bombycis CQ1] |
| novel-m0212-3p | NBO_48g0007 | hypothetical protein NBO_48g0007 [Nosema bombycis CQ1] |
| novel-m0212-3p | NBO_546g0001 | hypothetical protein NBO_546g0001 [Nosema bombycis CQ1] |
| novel-m0212-3p | NBO_549g0007 | DNA polymerase epsilon catalytic subunit A [Nosema bombycis CQ1] |
| novel-m0212-3p | NBO_54g0012 | hypothetical protein NBO_54g0012 [Nosema bombycis CQ1] |
| novel-m0212-3p | NBO_552g0002 | spore wall protein 5 [Nosema bombycis] |
| novel-m0212-3p | NBO_55g0012 | hypothetical protein NBO_55g0012 [Nosema bombycis CQ1] |
| novel-m0212-3p | NBO_633g0001 | DNA mismatch repair protein mutS [Nosema bombycis CQ1] |
| novel-m0212-3p | NBO_63g0005 | Triosephosphate isomerase [Nosema bombycis CQ1] |
| novel-m0212-3p | NBO_63g0006 | Triosephosphate isomerase [Nosema bombycis CQ1] |
| novel-m0212-3p | NBO_64g0003 | Putative membrane protein ycf1 [Nosema bombycis CQ1] |
| novel-m0212-3p | NBO_69g0004 | DNA polymerase alpha subunit B, partial [Nosema bombycis CQ1] |
| novel-m0212-3p | NBO_71g0002 | isoleucyl-tRNA synthetase, cytoplasmic [Nosema bombycis CQ1] |
| novel-m0212-3p | NBO_76g0003 | hypothetical protein NBO_76g0003 [Nosema bombycis CQ1] |
| novel-m0212-3p | NBO_78g0013 | hypothetical protein NBO_78g0013 [Nosema bombycis CQ1] |
| novel-m0212-3p | NBO_81g0012 | MADS domain containing protein, partial [Nosema bombycis CQ1] |
| novel-m0212-3p | NBO_841g0001 | hypothetical protein NBO_841g0001 [Nosema bombycis CQ1] |
| novel-m0212-3p | NBO_9g0004 | hypothetical protein NBO_9g0004 [Nosema bombycis CQ1] |
| novel-m0212-5p | NBO_1054gi001 | T complex protein 1 subunit beta [Nosema bombycis CQ1] |
| novel-m0212-5p | NBO_1552gi001 | poly(A) polymerase type 1, partial [Nosema bombycis CQ1] |
| novel-m0212-5p | NBO_16g0010 | 2Fe-2S ferredoxin [Nosema bombycis CQ1] |
| novel-m0212-5p | NBO_6g0015 | Ricin B lectin [Nosema bombycis CQ1] |
| novel-m0022-5p | NBO_13g0031 | eukaryotic translation initiation factor 4E [Encephalitozoon romaleae SJ-2008] |
| novel-m0022-5p | NBO_34g0043 | Serine/threonine-protein phosphatase 4 catalytic subunit [Nosema bombycis CQ1] |
| novel-m0022-5p | NBO_3g0006 | 60S ribosomal protein L6, partial [Nosema bombycis] |
| novel-m0022-5p | NBO_443g0001 | nucleic acid-binding protein [Nosema ceranae] |
| novel-m0022-5p | NBO_55g0009 | hypothetical protein NBO_55g0009 [Nosema bombycis CQ1] |
| novel-m0022-5p | NBO_63g0005 | Triosephosphate isomerase [Nosema bombycis CQ1] |
| novel-m0022-5p | NBO_63g0006 | Triosephosphate isomerase [Nosema bombycis CQ1] |
| novel-m0050-3p | NBO_1066g0001 | hypothetical protein NBO_1066g0001 [Nosema bombycis CQ1] |
| novel-m0050-3p | NBO_10g0052 | Polar tube protein 3 [Nosema bombycis CQ1] |
| novel-m0050-3p | NBO_12g0008 | hypothetical protein NBO_12g0008 [Nosema bombycis CQ1] |
| novel-m0050-3p | NBO_29g0016 | hypothetical protein NBO_29g0016, partial [Nosema bombycis CQ1] |
| novel-m0050-3p | NBO_452g0003 | Peptidyl-prolyl cis-trans isomerase NIMA-interacting 1 [Nosema bombycis CQ1] |
| novel-m0050-3p | NBO_48g0007 | hypothetical protein NBO_48g0007 [Nosema bombycis CQ1] |
| novel-m0050-3p | NBO_55g0005 | mitochondrial pyruvate dehydrogenase E1 component subunit alpha [Nosema bombycis] |
| novel-m0050-3p | NBO_64g0003 | Putative membrane protein ycf1 [Nosema bombycis CQ1] |
| novel-m0050-3p | NBO_80g0024 | Major facilitator superfamily domain-containing protein 1 [Nosema bombycis CQ1] |
| novel-m0050-3p | NBO_845g0001 | hypothetical protein NBO_845g0001 [Nosema bombycis CQ1] |
| novel-m0079-5p | NBO_1053g0002 | Nuclear pore complex protein Nup98-Nup96 [Nosema bombycis CQ1] |
| novel-m0079-5p | NBO_1133g0001 | Ricin B lectin [Nosema bombycis CQ1] |
| novel-m0079-5p | NBO_1168gi001 | SCF ubiquitin ligase and anaphase-promoting complex protein [Encephalitozoon intestinalis ATCC 50506] |
| novel-m0079-5p | NBO_1320g0001 | Hexokinase-2 [Nosema bombycis CQ1] |
| novel-m0079-5p | NBO_13g0067 | f-box domain-containing protein [Nosema ceranae] |
| novel-m0079-5p | NBO_19g0026 | U3 small nucleolar ribonucleoprotein imp4 [Nosema bombycis CQ1] |
| novel-m0079-5p | NBO_29g0030 | Nuclear pore complex protein Nup98-Nup96 [Nosema bombycis CQ1] |
| novel-m0079-5p | NBO_32g0022 | Glutaredoxin [Nosema bombycis CQ1] |
| novel-m0079-5p | NBO_360g0003 | Deoxyuridine 5'-triphosphate nucleotidohydrolase [Nosema bombycis CQ1] |
| novel-m0079-5p | NBO_6g0030 | ATP-dependent DNA helicase hus2/rqh1 [Nosema bombycis CQ1] |
| novel-m0079-5p | NBO_6gi003 | hypothetical protein NBO_6gi003 [Nosema bombycis CQ1] |
| novel-m0079-5p | NBO_81g0012 | MADS domain containing protein, partial [Nosema bombycis CQ1] |
| novel-m0082-3p | NBO_1024g0001 | DNA-directed RNA polymerase II subunit RPB3 [Nosema bombycis CQ1] |
| novel-m0082-3p | NBO_452g0002 | Sld5 domain-containing protein [Rhizoctonia solani AG-1 IA] |
| novel-m0082-3p | NBO_76g0012 | type 2 peptidyl-tRNA hydrolase [Encephalitozoon romaleae SJ-2008] |
| novel-m0140-3p | NBO_10g0052 | Polar tube protein 3 [Nosema bombycis CQ1] |
| novel-m0140-3p | NBO_4g0029 | polar tube protein 3 [Nosema bombycis] |
| novel-m0169-3p | NBO_1024g0001 | DNA-directed RNA polymerase II subunit RPB3 [Nosema bombycis CQ1] |
| novel-m0169-3p | NBO_452g0002 | Sld5 domain-containing protein [Rhizoctonia solani AG-1 IA] |
| novel-m0169-3p | NBO_76g0012 | type 2 peptidyl-tRNA hydrolase [Encephalitozoon romaleae SJ-2008] |
| novel-m0174-3p | NBO_10g0052 | Polar tube protein 3 [Nosema bombycis CQ1] |
| novel-m0174-3p | NBO_16g0027 | Heat shock 70 kDa protein cognate 4 [Nosema bombycis CQ1] |
| novel-m0174-3p | NBO_28g0065 | hypothetical protein NBO_28g0065 [Nosema bombycis CQ1] |
| novel-m0174-3p | NBO_32g0024 | General transcriptional corepressor ssn6 [Nosema bombycis CQ1] |
| novel-m0174-3p | NBO_33g0018 | 78 kDa glucose-regulated protein [Nosema bombycis CQ1] |
| novel-m0174-3p | NBO_38g0025 | eukaryotic translation initiation factor 2C 2 [Nosema bombycis CQ1] |
| novel-m0174-3p | NBO_419g0005 | hypothetical protein NBO_419g0005 [Nosema bombycis CQ1] |
| novel-m0174-3p | NBO_452g0003 | Peptidyl-prolyl cis-trans isomerase NIMA-interacting 1 [Nosema bombycis CQ1] |
| novel-m0174-3p | NBO_4g0029 | polar tube protein 3 [Nosema bombycis] |
| novel-m0174-3p | NBO_508g0021 | Rac-like GTP-binding protein ARAC7 [Nosema bombycis CQ1] |
| novel-m0174-3p | NBO_6g0028 | hypothetical protein NBO_6g0028 [Nosema bombycis CQ1] |
| novel-m0174-3p | NBO_80g0017 | Integrator complex subunit 11 [Nosema bombycis CQ1] |
| novel-m0192-3p | NBO_1066g0001 | hypothetical protein NBO_1066g0001 [Nosema bombycis CQ1] |
| novel-m0192-3p | NBO_10g0052 | Polar tube protein 3 [Nosema bombycis CQ1] |
| novel-m0192-3p | NBO_12g0008 | hypothetical protein NBO_12g0008 [Nosema bombycis CQ1] |
| novel-m0192-3p | NBO_29g0016 | hypothetical protein NBO_29g0016, partial [Nosema bombycis CQ1] |
| novel-m0192-3p | NBO_452g0003 | Peptidyl-prolyl cis-trans isomerase NIMA-interacting 1 [Nosema bombycis CQ1] |
| novel-m0192-3p | NBO_48g0007 | hypothetical protein NBO_48g0007 [Nosema bombycis CQ1] |
| novel-m0192-3p | NBO_55g0005 | mitochondrial pyruvate dehydrogenase E1 component subunit alpha [Nosema bombycis] |
| novel-m0192-3p | NBO_64g0003 | Putative membrane protein ycf1 [Nosema bombycis CQ1] |
| novel-m0192-3p | NBO_80g0024 | Major facilitator superfamily domain-containing protein 1 [Nosema bombycis CQ1] |
| novel-m0192-3p | NBO_845g0001 | hypothetical protein NBO_845g0001 [Nosema bombycis CQ1] |
| novel-m0071-5p | NBO_1232g0001 | hypothetical protein NBO_1232g0001 [Nosema bombycis CQ1] |
| novel-m0071-5p | NBO_369g0001 | exosome complex exonuclease rrp4, partial [Nosema bombycis CQ1] |
| novel-m0071-5p | NBO_53g0022 | DRAP deaminase [Nosema bombycis CQ1] |
| novel-m0093-3p | NBO_16g0003 | hypothetical protein NBO_16g0003 [Nosema bombycis CQ1] |
| novel-m0093-3p | NBO_18g0007 | hypothetical protein NBO_18g0007 [Nosema bombycis CQ1] |
| novel-m0093-3p | NBO_29g0021 | Spore wall and anchoring disk complex protein [Nosema bombycis CQ1] |
| novel-m0093-3p | NBO_364g0016 | hypothetical protein NBO_364g0016 [Nosema bombycis CQ1] |
| novel-m0093-3p | NBO_375g0010 | 2,3-bisphosphoglycerate-independent phosphoglycerate mutase [Nosema bombycis CQ1] |
| novel-m0093-3p | NBO_4g0049 | clathrin coat assembly protein [Nosema bombycis CQ1] |
| novel-m0093-3p | NBO_560g0001 | DNA-binding protein SMUBP-2 [Nosema bombycis CQ1] |
| novel-m0093-3p | NBO_568g0002 | Spore wall and anchoring disk complex protein [Nosema bombycis CQ1] |
| novel-m0093-3p | NBO_6g0015 | Ricin B lectin [Nosema bombycis CQ1] |
| novel-m0093-3p | NBO_6g0086 | hypothetical protein NBO_6g0086 [Nosema bombycis CQ1] |
| novel-m0093-3p | NBO_73g0026 | hypothetical protein NBO_73g0026 [Nosema bombycis CQ1] |
| novel-m0112-3p | NBO_1131g0001 | hypothetical protein NBO_1131g0001 [Nosema bombycis CQ1] |
| novel-m0112-3p | NBO_27g0006 | integral membrane [Nosema apis BRL 01] |
| novel-m0112-3p | NBO_29gi002 | alpha,alpha trehalose-phosphate synthase [Nosema bombycis CQ1] |
| novel-m0112-3p | NBO_374g0007 | Glucosamine--fructose-6-phosphate aminotransferase 1 [Nosema bombycis CQ1] |
| novel-m0112-3p | NBO_38g0014 | hypothetical protein NBO_38g0014 [Nosema bombycis CQ1] |
| novel-m0112-3p | NBO_417g0011 | Solute carrier family 35 member C2, partial [Nosema bombycis CQ1] |
| novel-m0112-3p | NBO_418gi001 | integral membrane protein [Nosema bombycis CQ1] |
| novel-m0112-3p | NBO_41g0019 | eukaryotic translation initiation factor 4a [Nosema ceranae] |
| novel-m0112-3p | NBO_568g0004 | Alpha,alpha-trehalose-phosphate synthase, partial [Nosema bombycis CQ1] |
| novel-m0112-3p | NBO_582gi001 | alpha,alpha trehalose-phosphate synthase, partial [Nosema bombycis CQ1] |
| novel-m0112-3p | NBO_6g0048 | Ricin B lectin [Nosema bombycis CQ1] |
| novel-m0112-3p | NBO_800gi001 | dopey-like leucine zipper transcription factor [Nosema ceranae] |
| novel-m0112-3p | NBO_9g0005 | 60S ribosomal protein L10 [Nosema bombycis] |
| novel-m0147-5p | NBO_256g0003 | hypothetical protein NBO_256g0003 [Nosema bombycis CQ1] |
| novel-m0147-5p | NBO_6g0015 | Ricin B lectin [Nosema bombycis CQ1] |
| novel-m0147-5p | NBO_76g0003 | hypothetical protein NBO_76g0003 [Nosema bombycis CQ1] |
| novel-m0213-3p | NBO_404g0002 | hypothetical protein NBO_404g0002 [Nosema bombycis CQ1] |
| novel-m0213-3p | NBO_55g0006 | Cyclin-A1-2, partial [Nosema bombycis CQ1] |
| novel-m0213-3p | NBO_60g0017 | Transcription-associated protein 1 [Nosema bombycis CQ1] |
| novel-m0213-3p | NBO_64g0051 | hypothetical protein NBO_64g0051 [Nosema bombycis CQ1] |
| novel-m0213-3p | NBO_70g0007 | hypothetical protein NBO_70g0007 [Nosema bombycis CQ1] |
| novel-m0213-3p | NBO_81g0012 | MADS domain containing protein, partial [Nosema bombycis CQ1] |
| novel-m0102-5p | NBO_1024g0001 | DNA-directed RNA polymerase II subunit RPB3 [Nosema bombycis CQ1] |
| novel-m0102-5p | NBO_1059gi002 | glycerol 3-phosphate dehydrogenase, partial [Nosema bombycis CQ1] |
| novel-m0102-5p | NBO_111g0002 | Serine palmitoyltransferase 1 [Nosema bombycis CQ1] |
| novel-m0102-5p | NBO_1230gi001 | hypothetical protein NBO_1230gi001 [Nosema bombycis CQ1] |
| novel-m0102-5p | NBO_1232g0001 | hypothetical protein NBO_1232g0001 [Nosema bombycis CQ1] |
| novel-m0102-5p | NBO_153g0004 | zinc metalloprotease [Nosema ceranae] |
| novel-m0102-5p | NBO_1552gi001 | poly(A) polymerase type 1, partial [Nosema bombycis CQ1] |
| novel-m0102-5p | NBO_166g0002 | Transcription-associated protein 1, partial [Nosema bombycis CQ1] |
| novel-m0102-5p | NBO_20g0004 | Bifunctional xylanase/deacetylase [Nosema bombycis CQ1] |
| novel-m0102-5p | NBO_20g0008 | peptide chain release factor 2 [Nosema ceranae] |
| novel-m0102-5p | NBO_27g0020 | hypothetical protein NBO_27g0020 [Nosema bombycis CQ1] |
| novel-m0102-5p | NBO_29g0021 | Spore wall and anchoring disk complex protein [Nosema bombycis CQ1] |
| novel-m0102-5p | NBO_2g0026 | deoxyribodipyrimidine photo-lyase [Nosema bombycis CQ1] |
| novel-m0102-5p | NBO_2g0074 | Nuclear elongation and deformation protein 1 [Nosema bombycis CQ1] |
| novel-m0102-5p | NBO_33g0017 | hypothetical protein NBO_33g0017 [Nosema bombycis CQ1] |
| novel-m0102-5p | NBO_34g0010 | mitochondrial protein import protein MAS5 [Nosema pernyi] |
| novel-m0102-5p | NBO_374g0007 | Glucosamine--fructose-6-phosphate aminotransferase 1 [Nosema bombycis CQ1] |
| novel-m0102-5p | NBO_384g0005 | hypothetical protein NBO_384g0005 [Nosema bombycis CQ1] |
| novel-m0102-5p | NBO_386g0004 | Meiotic expression up-regulated protein 26 [Nosema bombycis CQ1] |
| novel-m0102-5p | NBO_38g0012 | hypothetical protein NBO_38g0012 [Nosema bombycis CQ1] |
| novel-m0102-5p | NBO_396g0002 | TAF4 transcription initiation factor TFIID component [Encephalitozoon intestinalis ATCC 50506] |
| novel-m0102-5p | NBO_3g0033 | hypothetical protein NBO_3g0033 [Nosema bombycis CQ1] |
| novel-m0102-5p | NBO_463g0002 | Ricin B lectin [Nosema bombycis CQ1] |
| novel-m0102-5p | NBO_468g0001 | hypothetical protein NBO_468g0001 [Nosema bombycis CQ1] |
| novel-m0102-5p | NBO_48g0007 | hypothetical protein NBO_48g0007 [Nosema bombycis CQ1] |
| novel-m0102-5p | NBO_4g0031 | elongation factor 2 [Nosema bombycis CQ1] |
| novel-m0102-5p | NBO_568g0002 | Spore wall and anchoring disk complex protein [Nosema bombycis CQ1] |
| novel-m0102-5p | NBO_582g0002 | Tristetraproline [Nosema bombycis CQ1] |
| novel-m0102-5p | NBO_58g0010 | Chitin synthase 7 [Nosema bombycis CQ1] |
| novel-m0102-5p | NBO_602gi001 | serine palmitoyltransferase subunit 1 [Nosema bombycis CQ1] |
| novel-m0102-5p | NBO_60g0017 | Transcription-associated protein 1 [Nosema bombycis CQ1] |
| novel-m0102-5p | NBO_63g0022 | hypothetical protein NBO_63g0022 [Nosema bombycis CQ1] |
| novel-m0102-5p | NBO_671gi001 | hiv1-tat interacting protein [Nosema bombycis CQ1] |
| novel-m0102-5p | NBO_6g0086 | hypothetical protein NBO_6g0086 [Nosema bombycis CQ1] |
| novel-m0102-5p | NBO_7g0016 | Polar tube protein 1 [Nosema bombycis CQ1] |
| novel-m0102-5p | NBO_80g0024 | Major facilitator superfamily domain-containing protein 1 [Nosema bombycis CQ1] |
| novel-m0102-5p | NBO_92g0005 | Beta-lactamase, class A [Nosema bombycis CQ1] |
| novel-m0102-5p | NBO_943g0004 | Polar tube protein 1 [Nosema bombycis CQ1] |
| novel-m0114-3p | NBO_10g0052 | Polar tube protein 3 [Nosema bombycis CQ1] |
| novel-m0114-3p | NBO_1110g0001 | SEC31-like protein invovled in vesicular transport from ER to Golgi [Nosema bombycis CQ1] |
| novel-m0114-3p | NBO_11g0052 | Flap endonuclease 1-A [Nosema bombycis CQ1] |
| novel-m0114-3p | NBO_1203g0001 | hypothetical protein NBO_1203g0001 [Nosema bombycis CQ1] |
| novel-m0114-3p | NBO_1246g0001 | Serine/threonine-protein phosphatase PP2A catalytic subunit [Nosema bombycis CQ1] |
| novel-m0114-3p | NBO_1522g0001 | spore wall protein 8 [Nosema antheraeae] |
| novel-m0114-3p | NBO_16g0024 | hypothetical protein NBO_16g0024 [Nosema bombycis CQ1] |
| novel-m0114-3p | NBO_16g0028 | hypothetical protein NBO_16g0028 [Nosema bombycis CQ1] |
| novel-m0114-3p | NBO_28g0074 | u3 small nucleolar ribonucleoprotein component mpp10 [Nosema ceranae] |
| novel-m0114-3p | NBO_2g0058 | RpeA, partial [Nosema bombycis CQ1] |
| novel-m0114-3p | NBO_374g0007 | Glucosamine--fructose-6-phosphate aminotransferase 1 [Nosema bombycis CQ1] |
| novel-m0114-3p | NBO_386g0004 | Meiotic expression up-regulated protein 26 [Nosema bombycis CQ1] |
| novel-m0114-3p | NBO_41g0030 | hypothetical protein NBO_41g0030 [Nosema bombycis CQ1] |
| novel-m0114-3p | NBO_437g0002 | hypothetical protein NBO_437g0002 [Nosema bombycis CQ1] |
| novel-m0114-3p | NBO_447g0002 | Meiosis-specific protein HOP1 [Nosema bombycis CQ1] |
| novel-m0114-3p | NBO_459g0003 | Meiosis-specific protein HOP1 [Nosema bombycis CQ1] |
| novel-m0114-3p | NBO_46g0002 | hypothetical protein NBO_46g0002 [Nosema bombycis CQ1] |
| novel-m0114-3p | NBO_4g0029 | polar tube protein 3 [Nosema bombycis] |
| novel-m0114-3p | NBO_4g0042 | TBC1 domain family member 13 [Nosema bombycis CQ1] |
| novel-m0114-3p | NBO_602gi001 | serine palmitoyltransferase subunit 1 [Nosema bombycis CQ1] |
| novel-m0114-3p | NBO_6g0040 | hypothetical protein NBO_6g0040 [Nosema bombycis CQ1] |
| novel-m0114-3p | NBO_73g0016 | hypothetical protein NBO_73g0016 [Nosema bombycis CQ1] |
| novel-m0114-3p | NBO_80g0024 | Major facilitator superfamily domain-containing protein 1 [Nosema bombycis CQ1] |
| novel-m0114-3p | NBO_80g0028 | proteasome subunit beta type-6 [Nosema bombycis CQ1] |
| novel-m0114-3p | NBO_81g0002 | MADS domain containing protein [Nosema pernyi] |
| novel-m0114-3p | NBO_81g0012 | MADS domain containing protein, partial [Nosema bombycis CQ1] |
| novel-m0114-3p | NBO_979g0001 | transcription initiation factor brf1 subunit-like protein [Nosema ceranae] |
| novel-m0114-3p | NBO_9g0005 | 60S ribosomal protein L10 [Nosema bombycis] |
| novel-m0116-3p | NBO_1000gi001 | Protein PNS1, partial [Nosema bombycis CQ1] |
| novel-m0116-3p | NBO_1086g0001 | hypothetical protein NBO_1086g0001 [Nosema bombycis CQ1] |
| novel-m0116-3p | NBO_11g0069 | hypothetical protein NBO_11g0069 [Nosema bombycis CQ1] |
| novel-m0116-3p | NBO_13g0067 | f-box domain-containing protein [Nosema ceranae] |
| novel-m0116-3p | NBO_16g0047 | Chitin synthase export chaperone [Nosema bombycis CQ1] |
| novel-m0116-3p | NBO_20g0008 | peptide chain release factor 2 [Nosema ceranae] |
| novel-m0116-3p | NBO_24g0011 | hypothetical protein NBO_24g0011 [Nosema bombycis CQ1] |
| novel-m0116-3p | NBO_2g0058 | RpeA, partial [Nosema bombycis CQ1] |
| novel-m0116-3p | NBO_33gi001 | Protein PNS1, partial [Nosema bombycis CQ1] |
| novel-m0116-3p | NBO_350g0001 | Protein PNS1, partial [Nosema bombycis CQ1] |
| novel-m0116-3p | NBO_3g0004 | hypothetical protein NBO_3g0004 [Nosema bombycis CQ1] |
| novel-m0116-3p | NBO_404g0004 | Serum response factor [Nosema bombycis CQ1] |
| novel-m0116-3p | NBO_462g0003 | hypothetical protein NBO_462g0003 [Nosema bombycis CQ1] |
| novel-m0116-3p | NBO_547g0003 | hypothetical protein NBO_547g0003 [Nosema bombycis CQ1] |
| novel-m0116-3p | NBO_54g0012 | hypothetical protein NBO_54g0012 [Nosema bombycis CQ1] |
| novel-m0116-3p | NBO_63g0005 | Triosephosphate isomerase [Nosema bombycis CQ1] |
| novel-m0116-3p | NBO_63g0006 | Triosephosphate isomerase [Nosema bombycis CQ1] |
| novel-m0116-3p | NBO_64gi001 | tRNA adenilyl transferase [Nosema bombycis CQ1] |
| novel-m0116-3p | NBO_6g0011 | hypothetical protein NBO_6g0011 [Nosema bombycis CQ1] |
| novel-m0116-3p | NBO_6g0112 | hypothetical protein NBO_6g0112 [Nosema bombycis CQ1] |
| novel-m0116-3p | NBO_71g0002 | isoleucyl-tRNA synthetase, cytoplasmic [Nosema bombycis CQ1] |
| novel-m0116-3p | NBO_71g0003 | hypothetical protein NBO_71g0003, partial [Nosema bombycis CQ1] |
| novel-m0116-3p | NBO_81g0015 | Serum response factor [Nosema bombycis CQ1] |
| novel-m0116-3p | NBO_841g0001 | hypothetical protein NBO_841g0001 [Nosema bombycis CQ1] |
| novel-m0116-3p | NBO_911gi001 | XPF/ERCC4/RAD1 family like protein, partial [Nosema bombycis CQ1] |
| novel-m0117-5p | NBO_1000gi001 | Protein PNS1, partial [Nosema bombycis CQ1] |
| novel-m0117-5p | NBO_1086g0001 | hypothetical protein NBO_1086g0001 [Nosema bombycis CQ1] |
| novel-m0117-5p | NBO_11g0069 | hypothetical protein NBO_11g0069 [Nosema bombycis CQ1] |
| novel-m0117-5p | NBO_13g0067 | f-box domain-containing protein [Nosema ceranae] |
| novel-m0117-5p | NBO_16g0047 | Chitin synthase export chaperone [Nosema bombycis CQ1] |
| novel-m0117-5p | NBO_20g0008 | peptide chain release factor 2 [Nosema ceranae] |
| novel-m0117-5p | NBO_24g0011 | hypothetical protein NBO_24g0011 [Nosema bombycis CQ1] |
| novel-m0117-5p | NBO_2g0058 | RpeA, partial [Nosema bombycis CQ1] |
| novel-m0117-5p | NBO_33gi001 | Protein PNS1, partial [Nosema bombycis CQ1] |
| novel-m0117-5p | NBO_350g0001 | Protein PNS1, partial [Nosema bombycis CQ1] |
| novel-m0117-5p | NBO_3g0004 | hypothetical protein NBO_3g0004 [Nosema bombycis CQ1] |
| novel-m0117-5p | NBO_404g0004 | Serum response factor [Nosema bombycis CQ1] |
| novel-m0117-5p | NBO_462g0003 | hypothetical protein NBO_462g0003 [Nosema bombycis CQ1] |
| novel-m0117-5p | NBO_547g0003 | hypothetical protein NBO_547g0003 [Nosema bombycis CQ1] |
| novel-m0117-5p | NBO_54g0012 | hypothetical protein NBO_54g0012 [Nosema bombycis CQ1] |
| novel-m0117-5p | NBO_63g0005 | Triosephosphate isomerase [Nosema bombycis CQ1] |
| novel-m0117-5p | NBO_63g0006 | Triosephosphate isomerase [Nosema bombycis CQ1] |
| novel-m0117-5p | NBO_64gi001 | tRNA adenilyl transferase [Nosema bombycis CQ1] |
| novel-m0117-5p | NBO_6g0011 | hypothetical protein NBO_6g0011 [Nosema bombycis CQ1] |
| novel-m0117-5p | NBO_6g0112 | hypothetical protein NBO_6g0112 [Nosema bombycis CQ1] |
| novel-m0117-5p | NBO_71g0002 | isoleucyl-tRNA synthetase, cytoplasmic [Nosema bombycis CQ1] |
| novel-m0117-5p | NBO_71g0003 | hypothetical protein NBO_71g0003, partial [Nosema bombycis CQ1] |
| novel-m0117-5p | NBO_81g0015 | Serum response factor [Nosema bombycis CQ1] |
| novel-m0117-5p | NBO_841g0001 | hypothetical protein NBO_841g0001 [Nosema bombycis CQ1] |
| novel-m0117-5p | NBO_911gi001 | XPF/ERCC4/RAD1 family like protein, partial [Nosema bombycis CQ1] |
| novel-m0131-5p | NBO_1024g0001 | DNA-directed RNA polymerase II subunit RPB3 [Nosema bombycis CQ1] |
| novel-m0131-5p | NBO_1059gi002 | glycerol 3-phosphate dehydrogenase, partial [Nosema bombycis CQ1] |
| novel-m0131-5p | NBO_111g0002 | Serine palmitoyltransferase 1 [Nosema bombycis CQ1] |
| novel-m0131-5p | NBO_1230gi001 | hypothetical protein NBO_1230gi001 [Nosema bombycis CQ1] |
| novel-m0131-5p | NBO_1232g0001 | hypothetical protein NBO_1232g0001 [Nosema bombycis CQ1] |
| novel-m0131-5p | NBO_153g0004 | zinc metalloprotease [Nosema ceranae] |
| novel-m0131-5p | NBO_1552gi001 | poly(A) polymerase type 1, partial [Nosema bombycis CQ1] |
| novel-m0131-5p | NBO_166g0002 | Transcription-associated protein 1, partial [Nosema bombycis CQ1] |
| novel-m0131-5p | NBO_20g0004 | Bifunctional xylanase/deacetylase [Nosema bombycis CQ1] |
| novel-m0131-5p | NBO_20g0008 | peptide chain release factor 2 [Nosema ceranae] |
| novel-m0131-5p | NBO_27g0020 | hypothetical protein NBO_27g0020 [Nosema bombycis CQ1] |
| novel-m0131-5p | NBO_29g0021 | Spore wall and anchoring disk complex protein [Nosema bombycis CQ1] |
| novel-m0131-5p | NBO_2g0026 | deoxyribodipyrimidine photo-lyase [Nosema bombycis CQ1] |
| novel-m0131-5p | NBO_2g0074 | Nuclear elongation and deformation protein 1 [Nosema bombycis CQ1] |
| novel-m0131-5p | NBO_33g0017 | hypothetical protein NBO_33g0017 [Nosema bombycis CQ1] |
| novel-m0131-5p | NBO_34g0010 | mitochondrial protein import protein MAS5 [Nosema pernyi] |
| novel-m0131-5p | NBO_374g0007 | Glucosamine--fructose-6-phosphate aminotransferase 1 [Nosema bombycis CQ1] |
| novel-m0131-5p | NBO_384g0005 | hypothetical protein NBO_384g0005 [Nosema bombycis CQ1] |
| novel-m0131-5p | NBO_386g0004 | Meiotic expression up-regulated protein 26 [Nosema bombycis CQ1] |
| novel-m0131-5p | NBO_38g0012 | hypothetical protein NBO_38g0012 [Nosema bombycis CQ1] |
| novel-m0131-5p | NBO_396g0002 | TAF4 transcription initiation factor TFIID component [Encephalitozoon intestinalis ATCC 50506] |
| novel-m0131-5p | NBO_3g0033 | hypothetical protein NBO_3g0033 [Nosema bombycis CQ1] |
| novel-m0131-5p | NBO_463g0002 | Ricin B lectin [Nosema bombycis CQ1] |
| novel-m0131-5p | NBO_468g0001 | hypothetical protein NBO_468g0001 [Nosema bombycis CQ1] |
| novel-m0131-5p | NBO_48g0007 | hypothetical protein NBO_48g0007 [Nosema bombycis CQ1] |
| novel-m0131-5p | NBO_4g0031 | elongation factor 2 [Nosema bombycis CQ1] |
| novel-m0131-5p | NBO_568g0002 | Spore wall and anchoring disk complex protein [Nosema bombycis CQ1] |
| novel-m0131-5p | NBO_582g0002 | Tristetraproline [Nosema bombycis CQ1] |
| novel-m0131-5p | NBO_58g0010 | Chitin synthase 7 [Nosema bombycis CQ1] |
| novel-m0131-5p | NBO_602gi001 | serine palmitoyltransferase subunit 1 [Nosema bombycis CQ1] |
| novel-m0131-5p | NBO_60g0017 | Transcription-associated protein 1 [Nosema bombycis CQ1] |
| novel-m0131-5p | NBO_63g0022 | hypothetical protein NBO_63g0022 [Nosema bombycis CQ1] |
| novel-m0131-5p | NBO_671gi001 | hiv1-tat interacting protein [Nosema bombycis CQ1] |
| novel-m0131-5p | NBO_6g0086 | hypothetical protein NBO_6g0086 [Nosema bombycis CQ1] |
| novel-m0131-5p | NBO_7g0016 | Polar tube protein 1 [Nosema bombycis CQ1] |
| novel-m0131-5p | NBO_80g0024 | Major facilitator superfamily domain-containing protein 1 [Nosema bombycis CQ1] |
| novel-m0131-5p | NBO_92g0005 | Beta-lactamase, class A [Nosema bombycis CQ1] |
| novel-m0131-5p | NBO_943g0004 | Polar tube protein 1 [Nosema bombycis CQ1] |
| novel-m0136-5p | NBO_1024g0001 | DNA-directed RNA polymerase II subunit RPB3 [Nosema bombycis CQ1] |
| novel-m0136-5p | NBO_1059gi002 | glycerol 3-phosphate dehydrogenase, partial [Nosema bombycis CQ1] |
| novel-m0136-5p | NBO_111g0002 | Serine palmitoyltransferase 1 [Nosema bombycis CQ1] |
| novel-m0136-5p | NBO_1230gi001 | hypothetical protein NBO_1230gi001 [Nosema bombycis CQ1] |
| novel-m0136-5p | NBO_1232g0001 | hypothetical protein NBO_1232g0001 [Nosema bombycis CQ1] |
| novel-m0136-5p | NBO_153g0004 | zinc metalloprotease [Nosema ceranae] |
| novel-m0136-5p | NBO_1552gi001 | poly(A) polymerase type 1, partial [Nosema bombycis CQ1] |
| novel-m0136-5p | NBO_166g0002 | Transcription-associated protein 1, partial [Nosema bombycis CQ1] |
| novel-m0136-5p | NBO_20g0004 | Bifunctional xylanase/deacetylase [Nosema bombycis CQ1] |
| novel-m0136-5p | NBO_20g0008 | peptide chain release factor 2 [Nosema ceranae] |
| novel-m0136-5p | NBO_27g0020 | hypothetical protein NBO_27g0020 [Nosema bombycis CQ1] |
| novel-m0136-5p | NBO_29g0021 | Spore wall and anchoring disk complex protein [Nosema bombycis CQ1] |
| novel-m0136-5p | NBO_2g0026 | deoxyribodipyrimidine photo-lyase [Nosema bombycis CQ1] |
| novel-m0136-5p | NBO_2g0074 | Nuclear elongation and deformation protein 1 [Nosema bombycis CQ1] |
| novel-m0136-5p | NBO_33g0017 | hypothetical protein NBO_33g0017 [Nosema bombycis CQ1] |
| novel-m0136-5p | NBO_34g0010 | mitochondrial protein import protein MAS5 [Nosema pernyi] |
| novel-m0136-5p | NBO_374g0007 | Glucosamine--fructose-6-phosphate aminotransferase 1 [Nosema bombycis CQ1] |
| novel-m0136-5p | NBO_384g0005 | hypothetical protein NBO_384g0005 [Nosema bombycis CQ1] |
| novel-m0136-5p | NBO_386g0004 | Meiotic expression up-regulated protein 26 [Nosema bombycis CQ1] |
| novel-m0136-5p | NBO_38g0012 | hypothetical protein NBO_38g0012 [Nosema bombycis CQ1] |
| novel-m0136-5p | NBO_396g0002 | TAF4 transcription initiation factor TFIID component [Encephalitozoon intestinalis ATCC 50506] |
| novel-m0136-5p | NBO_3g0033 | hypothetical protein NBO_3g0033 [Nosema bombycis CQ1] |
| novel-m0136-5p | NBO_463g0002 | Ricin B lectin [Nosema bombycis CQ1] |
| novel-m0136-5p | NBO_468g0001 | hypothetical protein NBO_468g0001 [Nosema bombycis CQ1] |
| novel-m0136-5p | NBO_48g0007 | hypothetical protein NBO_48g0007 [Nosema bombycis CQ1] |
| novel-m0136-5p | NBO_4g0031 | elongation factor 2 [Nosema bombycis CQ1] |
| novel-m0136-5p | NBO_568g0002 | Spore wall and anchoring disk complex protein [Nosema bombycis CQ1] |
| novel-m0136-5p | NBO_582g0002 | Tristetraproline [Nosema bombycis CQ1] |
| novel-m0136-5p | NBO_58g0010 | Chitin synthase 7 [Nosema bombycis CQ1] |
| novel-m0136-5p | NBO_602gi001 | serine palmitoyltransferase subunit 1 [Nosema bombycis CQ1] |
| novel-m0136-5p | NBO_60g0017 | Transcription-associated protein 1 [Nosema bombycis CQ1] |
| novel-m0136-5p | NBO_63g0022 | hypothetical protein NBO_63g0022 [Nosema bombycis CQ1] |
| novel-m0136-5p | NBO_671gi001 | hiv1-tat interacting protein [Nosema bombycis CQ1] |
| novel-m0136-5p | NBO_6g0086 | hypothetical protein NBO_6g0086 [Nosema bombycis CQ1] |
| novel-m0136-5p | NBO_7g0016 | Polar tube protein 1 [Nosema bombycis CQ1] |
| novel-m0136-5p | NBO_80g0024 | Major facilitator superfamily domain-containing protein 1 [Nosema bombycis CQ1] |
| novel-m0136-5p | NBO_92g0005 | Beta-lactamase, class A [Nosema bombycis CQ1] |
| novel-m0136-5p | NBO_943g0004 | Polar tube protein 1 [Nosema bombycis CQ1] |
| novel-m0149-3p | NBO_16g0023 | Chaperone protein dnaK [Nosema bombycis CQ1] |
| novel-m0149-3p | NBO_18g0012 | Cyclin-dependent kinases regulatory subunit [Nosema bombycis CQ1] |
| novel-m0149-3p | NBO_32gi001 | threonyl-tRNA synthetase [Nosema bombycis CQ1] |
| novel-m0149-3p | NBO_462g0004 | 60S ribosomal protein L37a [Nosema bombycis] |
| novel-m0149-3p | NBO_63g0022 | hypothetical protein NBO_63g0022 [Nosema bombycis CQ1] |
| novel-m0149-3p | NBO_6gi002 | 60S ribosomal protein L37a [Nosema bombycis] |
| novel-m0149-3p | NBO_76gi004 | threonyl-tRNA synthetase [Nosema bombycis CQ1] |
| novel-m0158-5p | NBO_1000gi001 | Protein PNS1, partial [Nosema bombycis CQ1] |
| novel-m0158-5p | NBO_108g0004 | Heat shock protein 90, partial [Nosema bombycis CQ1] |
| novel-m0158-5p | NBO_10g0052 | Polar tube protein 3 [Nosema bombycis CQ1] |
| novel-m0158-5p | NBO_153g0006 | 20s proteasome subunit [Nosema apis BRL 01] |
| novel-m0158-5p | NBO_1574g0001 | hypothetical protein NBO_1574g0001 [Nosema bombycis CQ1] |
| novel-m0158-5p | NBO_24g0004 | myosin heavy chain [Nosema ceranae] |
| novel-m0158-5p | NBO_32g0012 | hypothetical protein NBO_32g0012 [Nosema bombycis CQ1] |
| novel-m0158-5p | NBO_350g0001 | Protein PNS1, partial [Nosema bombycis CQ1] |
| novel-m0158-5p | NBO_366g0002 | Heat shock protein HSP 90-alpha 1 [Nosema bombycis CQ1] |
| novel-m0158-5p | NBO_463g0002 | Ricin B lectin [Nosema bombycis CQ1] |
| novel-m0158-5p | NBO_468g0005 | Heat shock protein 90 [Nosema bombycis CQ1] |
| novel-m0158-5p | NBO_46g0005 | hypothetical protein NBO_46g0005 [Nosema bombycis CQ1] |
| novel-m0158-5p | NBO_481g0001 | hypothetical protein NBO_936g0001 [Nosema bombycis CQ1] |
| novel-m0158-5p | NBO_49g0003 | Fructose-bisphosphate aldolase B [Nosema bombycis CQ1] |
| novel-m0158-5p | NBO_4g0029 | polar tube protein 3 [Nosema bombycis] |
| novel-m0158-5p | NBO_4g0061 | hypothetical protein NBO_4g0061 [Nosema bombycis CQ1] |
| novel-m0158-5p | NBO_53g0003 | hypothetical protein NBO_53g0003 [Nosema bombycis CQ1] |
| novel-m0158-5p | NBO_597g0004 | hypothetical protein NBO_597g0004 [Nosema bombycis CQ1] |
| novel-m0158-5p | NBO_7g0015 | polar tube protein 2 [Nosema bombycis] |
| novel-m0158-5p | NBO_80g0024 | Major facilitator superfamily domain-containing protein 1 [Nosema bombycis CQ1] |
| novel-m0158-5p | NBO_8g0003 | hypothetical protein NBO_8g0003 [Nosema bombycis CQ1] |
| novel-m0158-5p | NBO_936g0001 | hypothetical protein NBO_936g0001 [Nosema bombycis CQ1] |
| novel-m0158-5p | NBO_943g0003 | Polar tube protein 2 [Nosema bombycis CQ1] |
| novel-m0158-5p | NBO_9g0005 | 60S ribosomal protein L10 [Nosema bombycis] |
| novel-m0168-3p | NBO_10g0052 | Polar tube protein 3 [Nosema bombycis CQ1] |
| novel-m0168-3p | NBO_1110g0001 | SEC31-like protein invovled in vesicular transport from ER to Golgi [Nosema bombycis CQ1] |
| novel-m0168-3p | NBO_11g0052 | Flap endonuclease 1-A [Nosema bombycis CQ1] |
| novel-m0168-3p | NBO_1203g0001 | hypothetical protein NBO_1203g0001 [Nosema bombycis CQ1] |
| novel-m0168-3p | NBO_1246g0001 | Serine/threonine-protein phosphatase PP2A catalytic subunit [Nosema bombycis CQ1] |
| novel-m0168-3p | NBO_1522g0001 | spore wall protein 8 [Nosema antheraeae] |
| novel-m0168-3p | NBO_16g0024 | hypothetical protein NBO_16g0024 [Nosema bombycis CQ1] |
| novel-m0168-3p | NBO_16g0028 | hypothetical protein NBO_16g0028 [Nosema bombycis CQ1] |
| novel-m0168-3p | NBO_28g0074 | u3 small nucleolar ribonucleoprotein component mpp10 [Nosema ceranae] |
| novel-m0168-3p | NBO_2g0058 | RpeA, partial [Nosema bombycis CQ1] |
| novel-m0168-3p | NBO_374g0007 | Glucosamine--fructose-6-phosphate aminotransferase 1 [Nosema bombycis CQ1] |
| novel-m0168-3p | NBO_386g0004 | Meiotic expression up-regulated protein 26 [Nosema bombycis CQ1] |
| novel-m0168-3p | NBO_41g0030 | hypothetical protein NBO_41g0030 [Nosema bombycis CQ1] |
| novel-m0168-3p | NBO_437g0002 | hypothetical protein NBO_437g0002 [Nosema bombycis CQ1] |
| novel-m0168-3p | NBO_447g0002 | Meiosis-specific protein HOP1 [Nosema bombycis CQ1] |
| novel-m0168-3p | NBO_459g0003 | Meiosis-specific protein HOP1 [Nosema bombycis CQ1] |
| novel-m0168-3p | NBO_46g0002 | hypothetical protein NBO_46g0002 [Nosema bombycis CQ1] |
| novel-m0168-3p | NBO_4g0029 | polar tube protein 3 [Nosema bombycis] |
| novel-m0168-3p | NBO_4g0042 | TBC1 domain family member 13 [Nosema bombycis CQ1] |
| novel-m0168-3p | NBO_602gi001 | serine palmitoyltransferase subunit 1 [Nosema bombycis CQ1] |
| novel-m0168-3p | NBO_6g0040 | hypothetical protein NBO_6g0040 [Nosema bombycis CQ1] |
| novel-m0168-3p | NBO_73g0016 | hypothetical protein NBO_73g0016 [Nosema bombycis CQ1] |
| novel-m0168-3p | NBO_80g0024 | Major facilitator superfamily domain-containing protein 1 [Nosema bombycis CQ1] |
| novel-m0168-3p | NBO_80g0028 | proteasome subunit beta type-6 [Nosema bombycis CQ1] |
| novel-m0168-3p | NBO_81g0002 | MADS domain containing protein [Nosema pernyi] |
| novel-m0168-3p | NBO_81g0012 | MADS domain containing protein, partial [Nosema bombycis CQ1] |
| novel-m0168-3p | NBO_979g0001 | transcription initiation factor brf1 subunit-like protein [Nosema ceranae] |
| novel-m0168-3p | NBO_9g0005 | 60S ribosomal protein L10 [Nosema bombycis] |
| novel-m0179-3p | NBO_10g0052 | Polar tube protein 3 [Nosema bombycis CQ1] |
| novel-m0179-3p | NBO_1110g0001 | SEC31-like protein invovled in vesicular transport from ER to Golgi [Nosema bombycis CQ1] |
| novel-m0179-3p | NBO_11g0052 | Flap endonuclease 1-A [Nosema bombycis CQ1] |
| novel-m0179-3p | NBO_1203g0001 | hypothetical protein NBO_1203g0001 [Nosema bombycis CQ1] |
| novel-m0179-3p | NBO_1246g0001 | Serine/threonine-protein phosphatase PP2A catalytic subunit [Nosema bombycis CQ1] |
| novel-m0179-3p | NBO_1522g0001 | spore wall protein 8 [Nosema antheraeae] |
| novel-m0179-3p | NBO_16g0024 | hypothetical protein NBO_16g0024 [Nosema bombycis CQ1] |
| novel-m0179-3p | NBO_16g0028 | hypothetical protein NBO_16g0028 [Nosema bombycis CQ1] |
| novel-m0179-3p | NBO_28g0074 | u3 small nucleolar ribonucleoprotein component mpp10 [Nosema ceranae] |
| novel-m0179-3p | NBO_2g0058 | RpeA, partial [Nosema bombycis CQ1] |
| novel-m0179-3p | NBO_374g0007 | Glucosamine--fructose-6-phosphate aminotransferase 1 [Nosema bombycis CQ1] |
| novel-m0179-3p | NBO_386g0004 | Meiotic expression up-regulated protein 26 [Nosema bombycis CQ1] |
| novel-m0179-3p | NBO_41g0030 | hypothetical protein NBO_41g0030 [Nosema bombycis CQ1] |
| novel-m0179-3p | NBO_437g0002 | hypothetical protein NBO_437g0002 [Nosema bombycis CQ1] |
| novel-m0179-3p | NBO_447g0002 | Meiosis-specific protein HOP1 [Nosema bombycis CQ1] |
| novel-m0179-3p | NBO_459g0003 | Meiosis-specific protein HOP1 [Nosema bombycis CQ1] |
| novel-m0179-3p | NBO_46g0002 | hypothetical protein NBO_46g0002 [Nosema bombycis CQ1] |
| novel-m0179-3p | NBO_4g0029 | polar tube protein 3 [Nosema bombycis] |
| novel-m0179-3p | NBO_4g0042 | TBC1 domain family member 13 [Nosema bombycis CQ1] |
| novel-m0179-3p | NBO_602gi001 | serine palmitoyltransferase subunit 1 [Nosema bombycis CQ1] |
| novel-m0179-3p | NBO_6g0040 | hypothetical protein NBO_6g0040 [Nosema bombycis CQ1] |
| novel-m0179-3p | NBO_73g0016 | hypothetical protein NBO_73g0016 [Nosema bombycis CQ1] |
| novel-m0179-3p | NBO_80g0024 | Major facilitator superfamily domain-containing protein 1 [Nosema bombycis CQ1] |
| novel-m0179-3p | NBO_80g0028 | proteasome subunit beta type-6 [Nosema bombycis CQ1] |
| novel-m0179-3p | NBO_81g0002 | MADS domain containing protein [Nosema pernyi] |
| novel-m0179-3p | NBO_81g0012 | MADS domain containing protein, partial [Nosema bombycis CQ1] |
| novel-m0179-3p | NBO_979g0001 | transcription initiation factor brf1 subunit-like protein [Nosema ceranae] |
| novel-m0179-3p | NBO_9g0005 | 60S ribosomal protein L10 [Nosema bombycis] |
| novel-m0191-3p | NBO_10g0052 | Polar tube protein 3 [Nosema bombycis CQ1] |
| novel-m0191-3p | NBO_1110g0001 | SEC31-like protein invovled in vesicular transport from ER to Golgi [Nosema bombycis CQ1] |
| novel-m0191-3p | NBO_11g0052 | Flap endonuclease 1-A [Nosema bombycis CQ1] |
| novel-m0191-3p | NBO_1203g0001 | hypothetical protein NBO_1203g0001 [Nosema bombycis CQ1] |
| novel-m0191-3p | NBO_1246g0001 | Serine/threonine-protein phosphatase PP2A catalytic subunit [Nosema bombycis CQ1] |
| novel-m0191-3p | NBO_1522g0001 | spore wall protein 8 [Nosema antheraeae] |
| novel-m0191-3p | NBO_16g0024 | hypothetical protein NBO_16g0024 [Nosema bombycis CQ1] |
| novel-m0191-3p | NBO_16g0028 | hypothetical protein NBO_16g0028 [Nosema bombycis CQ1] |
| novel-m0191-3p | NBO_28g0074 | u3 small nucleolar ribonucleoprotein component mpp10 [Nosema ceranae] |
| novel-m0191-3p | NBO_2g0058 | RpeA, partial [Nosema bombycis CQ1] |
| novel-m0191-3p | NBO_374g0007 | Glucosamine--fructose-6-phosphate aminotransferase 1 [Nosema bombycis CQ1] |
| novel-m0191-3p | NBO_386g0004 | Meiotic expression up-regulated protein 26 [Nosema bombycis CQ1] |
| novel-m0191-3p | NBO_41g0030 | hypothetical protein NBO_41g0030 [Nosema bombycis CQ1] |
| novel-m0191-3p | NBO_437g0002 | hypothetical protein NBO_437g0002 [Nosema bombycis CQ1] |
| novel-m0191-3p | NBO_447g0002 | Meiosis-specific protein HOP1 [Nosema bombycis CQ1] |
| novel-m0191-3p | NBO_459g0003 | Meiosis-specific protein HOP1 [Nosema bombycis CQ1] |
| novel-m0191-3p | NBO_46g0002 | hypothetical protein NBO_46g0002 [Nosema bombycis CQ1] |
| novel-m0191-3p | NBO_4g0029 | polar tube protein 3 [Nosema bombycis] |
| novel-m0191-3p | NBO_4g0042 | TBC1 domain family member 13 [Nosema bombycis CQ1] |
| novel-m0191-3p | NBO_602gi001 | serine palmitoyltransferase subunit 1 [Nosema bombycis CQ1] |
| novel-m0191-3p | NBO_6g0040 | hypothetical protein NBO_6g0040 [Nosema bombycis CQ1] |
| novel-m0191-3p | NBO_73g0016 | hypothetical protein NBO_73g0016 [Nosema bombycis CQ1] |
| novel-m0191-3p | NBO_80g0024 | Major facilitator superfamily domain-containing protein 1 [Nosema bombycis CQ1] |
| novel-m0191-3p | NBO_80g0028 | proteasome subunit beta type-6 [Nosema bombycis CQ1] |
| novel-m0191-3p | NBO_81g0002 | MADS domain containing protein [Nosema pernyi] |
| novel-m0191-3p | NBO_81g0012 | MADS domain containing protein, partial [Nosema bombycis CQ1] |
| novel-m0191-3p | NBO_979g0001 | transcription initiation factor brf1 subunit-like protein [Nosema ceranae] |
| novel-m0191-3p | NBO_9g0005 | 60S ribosomal protein L10 [Nosema bombycis] |
| novel-m0201-3p | NBO_549g0007 | DNA polymerase epsilon catalytic subunit A [Nosema bombycis CQ1] |
| novel-m0029-5p | NBO_447g0003 | Protein of unknown function GLTT, partial [Nosema bombycis CQ1] |
| novel-m0044-3p | NBO_1000gi001 | Protein PNS1, partial [Nosema bombycis CQ1] |
| novel-m0044-3p | NBO_11g0012 | Homeobox protein HD-10 [Nosema bombycis CQ1] |
| novel-m0044-3p | NBO_33gi001 | Protein PNS1, partial [Nosema bombycis CQ1] |
| novel-m0044-3p | NBO_350g0001 | Protein PNS1, partial [Nosema bombycis CQ1] |
| novel-m0044-3p | NBO_38g0025 | eukaryotic translation initiation factor 2C 2 [Nosema bombycis CQ1] |
| novel-m0044-3p | NBO_401g0005 | YjeF family domain-containing protein [Anncaliia algerae PRA339] |
| novel-m0044-3p | NBO_429g0002 | hypothetical protein NBO_429g0002 [Nosema bombycis CQ1] |
| novel-m0044-3p | NBO_458g0008 | hypothetical protein NBO_458g0008 [Nosema bombycis CQ1] |
| novel-m0044-3p | NBO_4g0048 | hypothetical protein NBO_4g0048 [Nosema bombycis CQ1] |
| novel-m0044-3p | NBO_63g0015 | hypothetical protein NBO_63g0015, partial [Nosema bombycis CQ1] |
| novel-m0044-3p | NBO_64g0032 | YjeF family domain-containing protein [Anncaliia algerae PRA339] |
| novel-m0044-3p | NBO_76g0003 | hypothetical protein NBO_76g0003 [Nosema bombycis CQ1] |
| novel-m0044-3p | NBO_85g0005 | Homeobox protein HD-10 [Nosema bombycis CQ1] |
| novel-m0046-3p | NBO_1000gi001 | Protein PNS1, partial [Nosema bombycis CQ1] |
| novel-m0046-3p | NBO_11g0012 | Homeobox protein HD-10 [Nosema bombycis CQ1] |
| novel-m0046-3p | NBO_33gi001 | Protein PNS1, partial [Nosema bombycis CQ1] |
| novel-m0046-3p | NBO_350g0001 | Protein PNS1, partial [Nosema bombycis CQ1] |
| novel-m0046-3p | NBO_38g0025 | eukaryotic translation initiation factor 2C 2 [Nosema bombycis CQ1] |
| novel-m0046-3p | NBO_401g0005 | YjeF family domain-containing protein [Anncaliia algerae PRA339] |
| novel-m0046-3p | NBO_429g0002 | hypothetical protein NBO_429g0002 [Nosema bombycis CQ1] |
| novel-m0046-3p | NBO_458g0008 | hypothetical protein NBO_458g0008 [Nosema bombycis CQ1] |
| novel-m0046-3p | NBO_4g0048 | hypothetical protein NBO_4g0048 [Nosema bombycis CQ1] |
| novel-m0046-3p | NBO_63g0015 | hypothetical protein NBO_63g0015, partial [Nosema bombycis CQ1] |
| novel-m0046-3p | NBO_64g0032 | YjeF family domain-containing protein [Anncaliia algerae PRA339] |
| novel-m0046-3p | NBO_76g0003 | hypothetical protein NBO_76g0003 [Nosema bombycis CQ1] |
| novel-m0046-3p | NBO_85g0005 | Homeobox protein HD-10 [Nosema bombycis CQ1] |
| novel-m0076-3p | NBO_1057g0002 | histone h3 [Nosema apis BRL 01] |
| novel-m0076-3p | NBO_1066g0001 | hypothetical protein NBO_1066g0001 [Nosema bombycis CQ1] |
| novel-m0076-3p | NBO_1302g0001 | hypothetical protein NBO_1302g0001 [Nosema bombycis CQ1] |
| novel-m0076-3p | NBO_1345gi001 | enolase [Nosema bombycis CQ1] |
| novel-m0076-3p | NBO_174g0001 | hypothetical protein NBO_174g0001 [Nosema bombycis CQ1] |
| novel-m0076-3p | NBO_376g0001 | hypothetical protein NBO_376g0001 [Nosema bombycis CQ1] |
| novel-m0076-3p | NBO_38g0025 | eukaryotic translation initiation factor 2C 2 [Nosema bombycis CQ1] |
| novel-m0076-3p | NBO_3g0033 | hypothetical protein NBO_3g0033 [Nosema bombycis CQ1] |
| novel-m0076-3p | NBO_507g0012 | hypothetical protein NBO_507g0012 [Nosema bombycis CQ1] |
| novel-m0076-3p | NBO_537g0001 | DNA replication fork-blocking protein FOB1, partial [Nosema bombycis CQ1] |
| novel-m0076-3p | NBO_555g0001 | hypothetical protein NBO_555g0001 [Nosema bombycis CQ1] |
| novel-m0076-3p | NBO_69g0015 | serine threonine protein kinase [Nosema ceranae] |
| novel-m0076-3p | NBO_985g0001 | hypothetical protein NBO_985g0001 [Nosema bombycis CQ1] |
| novel-m0076-3p | NBO_9g0004 | hypothetical protein NBO_9g0004 [Nosema bombycis CQ1] |
| novel-m0107-5p | NBO_10g0052 | Polar tube protein 3 [Nosema bombycis CQ1] |
| novel-m0107-5p | NBO_423g0001 | NADPH-dependent diflavin oxidoreductase 1 [Nosema bombycis CQ1] |
| novel-m0107-5p | NBO_43g0006 | ATP-dependent RNA helicase DHX37 [Nosema bombycis CQ1] |
| novel-m0107-5p | NBO_490g0001 | hypothetical protein NBO_490g0001 [Nosema bombycis CQ1] |
| novel-m0107-5p | NBO_4g0029 | polar tube protein 3 [Nosema bombycis] |
| novel-m0108-3p | NBO_264g0001 | hypothetical protein NBO_264g0001, partial [Nosema bombycis CQ1] |
| novel-m0108-3p | NBO_2g0071 | hypothetical protein NBO_2g0071 [Nosema bombycis CQ1] |
| novel-m0109-3p | NBO_1057g0002 | histone h3 [Nosema apis BRL 01] |
| novel-m0109-3p | NBO_1066g0001 | hypothetical protein NBO_1066g0001 [Nosema bombycis CQ1] |
| novel-m0109-3p | NBO_1302g0001 | hypothetical protein NBO_1302g0001 [Nosema bombycis CQ1] |
| novel-m0109-3p | NBO_1345gi001 | enolase [Nosema bombycis CQ1] |
| novel-m0109-3p | NBO_174g0001 | hypothetical protein NBO_174g0001 [Nosema bombycis CQ1] |
| novel-m0109-3p | NBO_376g0001 | hypothetical protein NBO_376g0001 [Nosema bombycis CQ1] |
| novel-m0109-3p | NBO_38g0025 | eukaryotic translation initiation factor 2C 2 [Nosema bombycis CQ1] |
| novel-m0109-3p | NBO_3g0033 | hypothetical protein NBO_3g0033 [Nosema bombycis CQ1] |
| novel-m0109-3p | NBO_507g0012 | hypothetical protein NBO_507g0012 [Nosema bombycis CQ1] |
| novel-m0109-3p | NBO_537g0001 | DNA replication fork-blocking protein FOB1, partial [Nosema bombycis CQ1] |
| novel-m0109-3p | NBO_555g0001 | hypothetical protein NBO_555g0001 [Nosema bombycis CQ1] |
| novel-m0109-3p | NBO_69g0015 | serine threonine protein kinase [Nosema ceranae] |
| novel-m0109-3p | NBO_985g0001 | hypothetical protein NBO_985g0001 [Nosema bombycis CQ1] |
| novel-m0109-3p | NBO_9g0004 | hypothetical protein NBO_9g0004 [Nosema bombycis CQ1] |
| novel-m0119-3p | NBO_264g0001 | hypothetical protein NBO_264g0001, partial [Nosema bombycis CQ1] |
| novel-m0119-3p | NBO_2g0071 | hypothetical protein NBO_2g0071 [Nosema bombycis CQ1] |
| novel-m0121-3p | NBO_264g0001 | hypothetical protein NBO_264g0001, partial [Nosema bombycis CQ1] |
| novel-m0121-3p | NBO_2g0071 | hypothetical protein NBO_2g0071 [Nosema bombycis CQ1] |
| novel-m0142-5p | NBO_1024g0001 | DNA-directed RNA polymerase II subunit RPB3 [Nosema bombycis CQ1] |
| novel-m0142-5p | NBO_1059gi002 | glycerol 3-phosphate dehydrogenase, partial [Nosema bombycis CQ1] |
| novel-m0142-5p | NBO_111g0002 | Serine palmitoyltransferase 1 [Nosema bombycis CQ1] |
| novel-m0142-5p | NBO_1230gi001 | hypothetical protein NBO_1230gi001 [Nosema bombycis CQ1] |
| novel-m0142-5p | NBO_1232g0001 | hypothetical protein NBO_1232g0001 [Nosema bombycis CQ1] |
| novel-m0142-5p | NBO_153g0004 | zinc metalloprotease [Nosema ceranae] |
| novel-m0142-5p | NBO_1552gi001 | poly(A) polymerase type 1, partial [Nosema bombycis CQ1] |
| novel-m0142-5p | NBO_166g0002 | Transcription-associated protein 1, partial [Nosema bombycis CQ1] |
| novel-m0142-5p | NBO_20g0004 | Bifunctional xylanase/deacetylase [Nosema bombycis CQ1] |
| novel-m0142-5p | NBO_20g0008 | peptide chain release factor 2 [Nosema ceranae] |
| novel-m0142-5p | NBO_27g0020 | hypothetical protein NBO_27g0020 [Nosema bombycis CQ1] |
| novel-m0142-5p | NBO_29g0021 | Spore wall and anchoring disk complex protein [Nosema bombycis CQ1] |
| novel-m0142-5p | NBO_2g0026 | deoxyribodipyrimidine photo-lyase [Nosema bombycis CQ1] |
| novel-m0142-5p | NBO_2g0074 | Nuclear elongation and deformation protein 1 [Nosema bombycis CQ1] |
| novel-m0142-5p | NBO_33g0017 | hypothetical protein NBO_33g0017 [Nosema bombycis CQ1] |
| novel-m0142-5p | NBO_34g0010 | mitochondrial protein import protein MAS5 [Nosema pernyi] |
| novel-m0142-5p | NBO_374g0007 | Glucosamine--fructose-6-phosphate aminotransferase 1 [Nosema bombycis CQ1] |
| novel-m0142-5p | NBO_384g0005 | hypothetical protein NBO_384g0005 [Nosema bombycis CQ1] |
| novel-m0142-5p | NBO_386g0004 | Meiotic expression up-regulated protein 26 [Nosema bombycis CQ1] |
| novel-m0142-5p | NBO_38g0012 | hypothetical protein NBO_38g0012 [Nosema bombycis CQ1] |
| novel-m0142-5p | NBO_396g0002 | TAF4 transcription initiation factor TFIID component [Encephalitozoon intestinalis ATCC 50506] |
| novel-m0142-5p | NBO_3g0033 | hypothetical protein NBO_3g0033 [Nosema bombycis CQ1] |
| novel-m0142-5p | NBO_463g0002 | Ricin B lectin [Nosema bombycis CQ1] |
| novel-m0142-5p | NBO_468g0001 | hypothetical protein NBO_468g0001 [Nosema bombycis CQ1] |
| novel-m0142-5p | NBO_48g0007 | hypothetical protein NBO_48g0007 [Nosema bombycis CQ1] |
| novel-m0142-5p | NBO_4g0031 | elongation factor 2 [Nosema bombycis CQ1] |
| novel-m0142-5p | NBO_568g0002 | Spore wall and anchoring disk complex protein [Nosema bombycis CQ1] |
| novel-m0142-5p | NBO_582g0002 | Tristetraproline [Nosema bombycis CQ1] |
| novel-m0142-5p | NBO_58g0010 | Chitin synthase 7 [Nosema bombycis CQ1] |
| novel-m0142-5p | NBO_602gi001 | serine palmitoyltransferase subunit 1 [Nosema bombycis CQ1] |
| novel-m0142-5p | NBO_60g0017 | Transcription-associated protein 1 [Nosema bombycis CQ1] |
| novel-m0142-5p | NBO_63g0022 | hypothetical protein NBO_63g0022 [Nosema bombycis CQ1] |
| novel-m0142-5p | NBO_671gi001 | hiv1-tat interacting protein [Nosema bombycis CQ1] |
| novel-m0142-5p | NBO_6g0086 | hypothetical protein NBO_6g0086 [Nosema bombycis CQ1] |
| novel-m0142-5p | NBO_7g0016 | Polar tube protein 1 [Nosema bombycis CQ1] |
| novel-m0142-5p | NBO_80g0024 | Major facilitator superfamily domain-containing protein 1 [Nosema bombycis CQ1] |
| novel-m0142-5p | NBO_92g0005 | Beta-lactamase, class A [Nosema bombycis CQ1] |
| novel-m0142-5p | NBO_943g0004 | Polar tube protein 1 [Nosema bombycis CQ1] |
| novel-m0164-3p | NBO_264g0001 | hypothetical protein NBO_264g0001, partial [Nosema bombycis CQ1] |
| novel-m0164-3p | NBO_2g0071 | hypothetical protein NBO_2g0071 [Nosema bombycis CQ1] |
| novel-m0165-3p | NBO_1230gi001 | hypothetical protein NBO_1230gi001 [Nosema bombycis CQ1] |
| novel-m0165-3p | NBO_1232g0001 | hypothetical protein NBO_1232g0001 [Nosema bombycis CQ1] |
| novel-m0165-3p | NBO_1246g0001 | Serine/threonine-protein phosphatase PP2A catalytic subunit [Nosema bombycis CQ1] |
| novel-m0165-3p | NBO_166g0002 | Transcription-associated protein 1, partial [Nosema bombycis CQ1] |
| novel-m0165-3p | NBO_24g0012 | nucleotide excision repair factor TFIIH/TFIIK subunit cyclin H-like protein [Encephalitozoon intestinalis ATCC 50506] |
| novel-m0165-3p | NBO_29gi002 | alpha,alpha trehalose-phosphate synthase [Nosema bombycis CQ1] |
| novel-m0165-3p | NBO_38g0012 | hypothetical protein NBO_38g0012 [Nosema bombycis CQ1] |
| novel-m0165-3p | NBO_3g0028 | vacuolar atp synthase subunit d [Nosema ceranae] |
| novel-m0165-3p | NBO_41g0014 | Vacuolar amino acid transporter 5 [Nosema bombycis CQ1] |
| novel-m0165-3p | NBO_41g0024 | Vacuolar amino acid transporter 5 [Nosema bombycis CQ1] |
| novel-m0165-3p | NBO_41g0047 | thioltransferase [Nosema bombycis CQ1] |
| novel-m0165-3p | NBO_483g0001 | hypothetical protein NBO_483g0001 [Nosema bombycis CQ1] |
| novel-m0165-3p | NBO_508g0014 | hypothetical protein NBO_508g0014 [Nosema bombycis CQ1] |
| novel-m0165-3p | NBO_508g0035 | WD repeat-containing protein, partial [Nosema bombycis CQ1] |
| novel-m0165-3p | NBO_546g0001 | hypothetical protein NBO_546g0001 [Nosema bombycis CQ1] |
| novel-m0165-3p | NBO_60g0017 | Transcription-associated protein 1 [Nosema bombycis CQ1] |
| novel-m0165-3p | NBO_71g0003 | hypothetical protein NBO_71g0003, partial [Nosema bombycis CQ1] |
| novel-m0165-3p | NBO_73g0026 | hypothetical protein NBO_73g0026 [Nosema bombycis CQ1] |
| novel-m0165-3p | NBO_74g0004 | small-conductance mechanosensitive channel protein [Nosema ceranae] |
| novel-m0165-3p | NBO_974g0002 | Transcription-associated protein 1 [Nosema bombycis CQ1] |
| novel-m0224-3p | NBO_10g0027 | hypothetical protein NBO_10g0027 [Nosema bombycis CQ1] |
| novel-m0224-3p | NBO_10g0041 | hypothetical protein NBO_10g0041 [Nosema bombycis CQ1] |
| novel-m0224-3p | NBO_10g0052 | Polar tube protein 3 [Nosema bombycis CQ1] |
| novel-m0224-3p | NBO_10g0053 | DNA mismatch repair protein Mlh1 [Nosema bombycis CQ1] |
| novel-m0224-3p | NBO_1116g0004 | hypothetical protein NBO_1116g0004 [Nosema bombycis CQ1] |
| novel-m0224-3p | NBO_11g0041 | small-conductance mechanosensitive channel protein [Nosema ceranae] |
| novel-m0224-3p | NBO_11g0045 | hypothetical protein NBO_11g0045 [Nosema bombycis CQ1] |
| novel-m0224-3p | NBO_1203g0001 | hypothetical protein NBO_1203g0001 [Nosema bombycis CQ1] |
| novel-m0224-3p | NBO_1209gi001 | chromatin structure modulator [Nosema bombycis CQ1] |
| novel-m0224-3p | NBO_1246g0001 | Serine/threonine-protein phosphatase PP2A catalytic subunit [Nosema bombycis CQ1] |
| novel-m0224-3p | NBO_1276g0001 | tRNA acetyltransferase TAN1 [Nematocida displodere] |
| novel-m0224-3p | NBO_1291g0001 | Deoxyribodipyrimidine photo-lyase [Nosema bombycis CQ1] |
| novel-m0224-3p | NBO_1332gi002 | threonyl-tRNA synthetase [Nosema bombycis CQ1] |
| novel-m0224-3p | NBO_13g0067 | f-box domain-containing protein [Nosema ceranae] |
| novel-m0224-3p | NBO_16g0023 | Chaperone protein dnaK [Nosema bombycis CQ1] |
| novel-m0224-3p | NBO_20g0008 | peptide chain release factor 2 [Nosema ceranae] |
| novel-m0224-3p | NBO_28g0019 | DNA-directed RNA polymerases I and III subunit RPAC1 [Nosema bombycis CQ1] |
| novel-m0224-3p | NBO_293g0001 | hypothetical protein NBO_293g0001 [Nosema bombycis CQ1] |
| novel-m0224-3p | NBO_2g0026 | deoxyribodipyrimidine photo-lyase [Nosema bombycis CQ1] |
| novel-m0224-3p | NBO_2g0074 | Nuclear elongation and deformation protein 1 [Nosema bombycis CQ1] |
| novel-m0224-3p | NBO_31gi002 | small-conductance mechanosensitive channel protein [Nosema ceranae] |
| novel-m0224-3p | NBO_321g0001 | Phosphomannomutase 2 [Nosema bombycis CQ1] |
| novel-m0224-3p | NBO_32g0024 | General transcriptional corepressor ssn6 [Nosema bombycis CQ1] |
| novel-m0224-3p | NBO_33g0017 | hypothetical protein NBO_33g0017 [Nosema bombycis CQ1] |
| novel-m0224-3p | NBO_375g0009 | 2,3-bisphosphoglycerate-independent phosphoglycerate mutase [Nosema bombycis CQ1] |
| novel-m0224-3p | NBO_377g0003 | Heat shock 70 kDa protein 6 [Nosema bombycis CQ1] |
| novel-m0224-3p | NBO_37g0007 | KH domain-containing protein [Nosema bombycis CQ1] |
| novel-m0224-3p | NBO_389g0002 | hypothetical protein NBO_389g0002, partial [Nosema bombycis CQ1] |
| novel-m0224-3p | NBO_38g0009 | hypothetical protein NBO_38g0009 [Nosema bombycis CQ1] |
| novel-m0224-3p | NBO_39g0002 | hypothetical protein NBO_39g0002 [Nosema bombycis CQ1] |
| novel-m0224-3p | NBO_3g0004 | hypothetical protein NBO_3g0004 [Nosema bombycis CQ1] |
| novel-m0224-3p | NBO_3g0006 | 60S ribosomal protein L6, partial [Nosema bombycis] |
| novel-m0224-3p | NBO_3g0033 | hypothetical protein NBO_3g0033 [Nosema bombycis CQ1] |
| novel-m0224-3p | NBO_41g0030 | hypothetical protein NBO_41g0030 [Nosema bombycis CQ1] |
| novel-m0224-3p | NBO_423g0006 | Iron-sulfur clusters transporter atm1, mitochondrial, partial [Nosema bombycis CQ1] |
| novel-m0224-3p | NBO_444g0008 | Cysteinyl-tRNA synthetase, cytoplasmic [Nosema bombycis CQ1] |
| novel-m0224-3p | NBO_447g0002 | Meiosis-specific protein HOP1 [Nosema bombycis CQ1] |
| novel-m0224-3p | NBO_448g0002 | hypothetical protein NBO_448g0002 [Nosema bombycis CQ1] |
| novel-m0224-3p | NBO_458g0008 | hypothetical protein NBO_458g0008 [Nosema bombycis CQ1] |
| novel-m0224-3p | NBO_468g0001 | hypothetical protein NBO_468g0001 [Nosema bombycis CQ1] |
| novel-m0224-3p | NBO_4g0029 | polar tube protein 3 [Nosema bombycis] |
| novel-m0224-3p | NBO_4g0031 | elongation factor 2 [Nosema bombycis CQ1] |
| novel-m0224-3p | NBO_4g0050 | Insulin-degrading enzyme [Nosema bombycis CQ1] |
| novel-m0224-3p | NBO_507g0012 | hypothetical protein NBO_507g0012 [Nosema bombycis CQ1] |
| novel-m0224-3p | NBO_53g0003 | hypothetical protein NBO_53g0003 [Nosema bombycis CQ1] |
| novel-m0224-3p | NBO_560g0001 | DNA-binding protein SMUBP-2 [Nosema bombycis CQ1] |
| novel-m0224-3p | NBO_58g0026 | GPN-loop GTPase 1 [Nosema bombycis CQ1] |
| novel-m0224-3p | NBO_606g0001 | hypothetical protein NBO_606g0001 [Nosema bombycis CQ1] |
| novel-m0224-3p | NBO_63g0018 | hypothetical protein NBO_63g0018 [Nosema bombycis CQ1] |
| novel-m0224-3p | NBO_69g0015 | serine threonine protein kinase [Nosema ceranae] |
| novel-m0224-3p | NBO_6g0004 | hypothetical protein NBO_6g0004 [Nosema bombycis CQ1] |
| novel-m0224-3p | NBO_73g0016 | hypothetical protein NBO_73g0016 [Nosema bombycis CQ1] |
| novel-m0224-3p | NBO_76g0014 | hypothetical protein NBO_76g0014 [Nosema bombycis CQ1] |
| novel-m0224-3p | NBO_7g0006 | hypothetical protein NBO_7g0006 [Nosema bombycis CQ1] |
| novel-m0224-3p | NBO_800gi001 | dopey-like leucine zipper transcription factor [Nosema ceranae] |
| novel-m0224-3p | NBO_84g0004 | Glycerol-3-phosphate dehydrogenase, mitochondrial [Nosema bombycis CQ1] |
| novel-m0224-3p | NBO_915g0002 | Cell division control protein 48 E, partial [Nosema bombycis CQ1] |
| novel-m0224-3p | NBO_9g0004 | hypothetical protein NBO_9g0004 [Nosema bombycis CQ1] |

| **Table S6 LncRNA-miRNA-mRNA ceRNA networks of *N. bombycis* in the microsporidia congenitally infected silkworm embryos and larvae** | | | | | | | | | | | | | | | |
| --- | --- | --- | --- | --- | --- | --- | --- | --- | --- | --- | --- | --- | --- | --- | --- |
| mRNA | lncRNA | miRNA | | | | | | | | cor | | description | | | |
| MSTRG.1990 | MSTRG.2923.1 | novel-m0174-5p | | | | | | | | 0.985336078 | | ATP-dependent DNA helicase PIF1 [Hypsizygus marmoreus] | | | |
| MSTRG.8131 | MSTRG.1660.2 | novel-m0065-3p; novel-m0211-3p | | | | | | | | 0.968674691 | | pol polyprotein [Nosema bombycis] | | | |
| MSTRG.2926 | MSTRG.2923.1 | novel-m0224-3p | | | | | | | | 0.96755973 | | transposase [Nosema bombycis] | | | |
| MSTRG.8131 | MSTRG.4289.1 | novel-m0065-3p; novel-m0211-3p | | | | | | | | 0.958578439 | | pol polyprotein [Nosema bombycis] | | | |
| MSTRG.8131 | MSTRG.2923.1 | novel-m0211-3p; novel-m0065-3p | | | | | | | | 0.943529983 | | pol polyprotein [Nosema bombycis] | | | |
| MSTRG.5960 | MSTRG.2923.1 | novel-m0224-3p | | | | | | | | 0.933603307 | | pol polyprotein, partial [Nosema apis BRL 01] | | | |
| NBO_1203g0001 | MSTRG.5153.1 | novel-m0114-3p; novel-m0179-3p; novel-m0191-3p; novel-m0168-3p | | | | | | | | 0.932419796 | | hypothetical protein NBO_1203g0001 [Nosema bombycis CQ1] | | | |
| MSTRG.1369 | MSTRG.2923.1 | novel-m0029-5p | | | | | | | | 0.921848528 | | pol polyprotein [Nosema bombycis] | | | |
| MSTRG.1990 | MSTRG.4851.1 | novel-m0174-5p | | | | | | | | 0.918241476 | | ATP-dependent DNA helicase PIF1 [Hypsizygus marmoreus] | | | |
| NBO_34g0010 | MSTRG.5153.2 | novel-m0021-5p | | | | | | | | 0.916199717 | | mitochondrial protein import protein MAS5 [Nosema pernyi] | | | |
| NBO_8g0045 | MSTRG.2013.1 | novel-m0174-5p | | | | | | | | 0.914764126 | | signal peptidase like protein [Nosema bombycis CQ1] | | | |
| NBO_1116g0004 | MSTRG.7750.2 | novel-m0224-3p | | | | | | | | 0.90004157 | | hypothetical protein NBO_1116g0004 [Nosema bombycis CQ1] | | | |
| MSTRG.128 | MSTRG.4851.1 | novel-m0174-5p | | | | | | | | 0.899413044 | | transposase [Nosema bombycis] | | | |
| MSTRG.8131 | MSTRG.3258.1 | novel-m0211-3p; novel-m0065-3p | | | | | | | | 0.898187592 | | pol polyprotein [Nosema bombycis] | | | |
| NBO_4g0029 | MSTRG.7750.2 | novel-m0224-3p | | | | | | | | 0.894658807 | | polar tube protein 3 [Nosema bombycis] | | | |
| NBO_10g0052 | MSTRG.7750.2 | novel-m0224-3p | | | | | | | | 0.888798421 | | Polar tube protein 3 [Nosema bombycis CQ1] | | | |
| MSTRG.7603 | MSTRG.2923.1 | novel-m0174-5p | | | | | | | | 0.886035496 | | - | | | |
| MSTRG.1990 | MSTRG.2013.1 | novel-m0174-5p | | | | | | | | 0.878224277 | | ATP-dependent DNA helicase PIF1 [Hypsizygus marmoreus] | | | |
| MSTRG.7603 | MSTRG.2013.1 | novel-m0174-5p | | | | | | | | 0.876526036 | | - | | | |
| NBO_31gi002 | MSTRG.7750.2 | novel-m0224-3p | | | | | | | | 0.876241168 | | small-conductance mechanosensitive channel protein [Nosema ceranae] | | | |
| NBO_3g0004 | MSTRG.7750.2 | novel-m0224-3p | | | | | | | | 0.864333417 | | hypothetical protein NBO_3g0004 [Nosema bombycis CQ1] | | | |
| NBO_1332gi002 | MSTRG.7750.2 | novel-m0224-3p | | | | | | | | 0.862826449 | | threonyl-tRNA synthetase [Nosema bombycis CQ1] | | | |
| MSTRG.7603 | MSTRG.4851.1 | novel-m0174-5p | | | | | | | | 0.862033601 | | - | | | |
| NBO_20g0002 | MSTRG.5153.1 | novel-m0100-3p | | | | | | | | 0.848244081 | | Pescadillo like protein [Nosema bombycis CQ1] | | | |
| MSTRG.128 | MSTRG.2013.1 | novel-m0174-5p | | | | | | | | 0.845385811 | | transposase [Nosema bombycis] | |
| NBO_64g0028 | MSTRG.2013.1 | novel-m0174-5p | | | | | | | | 0.83676606 | | E3 ubiquitin-protein ligase [Nosema bombycis CQ1] | |
| NBO_11g0041 | MSTRG.7750.2 | novel-m0224-3p | | | | | | | | 0.834774646 | | small-conductance mechanosensitive channel protein [Nosema ceranae] | |
| NBO_34g0010 | MSTRG.10794.1 | novel-m0148-3p | | | | | | | | 0.833621844 | | mitochondrial protein import protein MAS5 [Nosema pernyi] | |
| MSTRG.128 | MSTRG.2923.1 | novel-m0174-5p | | | | | | | | 0.826007602 | | transposase [Nosema bombycis] | |
| NBO_8g0045 | MSTRG.4851.1 | novel-m0174-5p | | | | | | | | 0.823705004 | | signal peptidase like protein [Nosema bombycis CQ1] | |
| NBO_468g0005 | MSTRG.5153.3 | novel-m0158-5p | | | | | | | | 0.822671522 | | Heat shock protein 90 [Nosema bombycis CQ1] | |
| NBO_26g0007 | MSTRG.5153.1 | novel-m0100-3p | | | | | | | | 0.82153511 | | hypothetical protein NBO_26g0007 [Nosema bombycis CQ1] | |
| MSTRG.7066 | MSTRG.5153.2 | novel-m0158-5p | | | | | | | | 0.820067459 | | - | |
| NBO_28g0046 | MSTRG.5153.1 | novel-m0021-5p | | | | | | | | 0.818829361 | |  | |
| NBO_33g0017 | MSTRG.7750.2 | novel-m0224-3p | | | | | | | | 0.811237263 | | hypothetical protein NBO_33g0017 [Nosema bombycis CQ1] | |
| NBO_547g0003 | MSTRG.5153.1 | novel-m0100-3p | | | | | | | | 0.811208648 | | hypothetical protein NBO_547g0003 [Nosema bombycis CQ1] | |
| NBO_166g0002 | MSTRG.11899.2 | novel-m0072-5p; novel-m0165-3p | | | | | | | | 0.807092174 | | Transcription-associated protein 1, partial [Nosema bombycis CQ1] | |
| NBO_16g0023 | MSTRG.5153.1 | novel-m0021-5p | | | | | | | | 0.80527354 | | Chaperone protein dnaK [Nosema bombycis CQ1] | |
| NBO_114g0004 | MSTRG.12084.1 | novel-m0148-3p | | | | | | | | 0.804654883 | | Zinc phosphodiesterase ELAC protein 2 [Nosema bombycis CQ1] | |
| NBO_64g0028 | MSTRG.4851.1 | novel-m0174-5p | | | | | | | | 0.803304089 | | E3 ubiquitin-protein ligase [Nosema bombycis CQ1] | |
| NBO_547g0003 | MSTRG.12418.1 | novel-m0100-3p | | | | | | | | 0.803209444 | | hypothetical protein NBO_547g0003 [Nosema bombycis CQ1] | |
| NBO_1203g0001 | MSTRG.5153.2 | novel-m0191-3p; novel-m0179-3p; novel-m0114-3p; novel-m0168-3p | | | | | | | | 0.802027963 | | hypothetical protein NBO_1203g0001 [Nosema bombycis CQ1] | |
| NBO_108g0004 | MSTRG.5153.3 | novel-m0100-3p; novel-m0158-5p | | | | | | | | 0.801089964 | | Heat shock protein 90, partial [Nosema bombycis CQ1] | |
| NBO_3g0006 | MSTRG.7750.2 | novel-m0224-3p | | | | | | | | 0.799755193 | | 60S ribosomal protein L6, partial [Nosema bombycis] | |
| NBO_4g0017 | MSTRG.5153.1 | novel-m0021-5p | | | | | | | | 0.798667405 | | Heat shock protein 101 [Nosema bombycis CQ1] | |
| NBO_108g0004 | MSTRG.5153.1 | novel-m0100-3p; novel-m0158-5p | | | | | | | | 0.796941013 | | Heat shock protein 90, partial [Nosema bombycis CQ1] | |
| NBO_468g0005 | MSTRG.5153.1 | novel-m0158-5p | | | | | | | | 0.79649461 | | Heat shock protein 90 [Nosema bombycis CQ1] | |
| NBO_63g0022 | MSTRG.12084.1 | novel-m0148-3p | | | | | | | | 0.795395106 | | hypothetical protein NBO_63g0022 [Nosema bombycis CQ1] | |
| NBO_444g0008 | MSTRG.7750.2 | novel-m0224-3p | | | | | | | | 0.795127452 | | Cysteinyl-tRNA synthetase, cytoplasmic [Nosema bombycis CQ1] | |
| NBO_69g0015 | MSTRG.7750.2 | novel-m0224-3p | | | | | | | | 0.79485485 | | serine threonine protein kinase [Nosema ceranae] | |
| NBO_6g0049 | MSTRG.10794.1 | novel-m0148-3p | | | | | | | | 0.789020032 | | Ricin B lectin [Nosema bombycis CQ1] | |
| NBO_1170gi001 | MSTRG.5153.1 | novel-m0021-5p | | | | | | | | 0.787610543 | | HSP 101 related protein, partial [Nosema bombycis CQ1] | |
| MSTRG.63 | MSTRG.2173.1 | novel-m0065-3p; novel-m0211-3p | | | | | | | | 0.786168325 | | hypothetical spore wall protein 14 [Nosema bombycis] | |
| MSTRG.7066 | MSTRG.5153.3 | novel-m0158-5p | | | | | | | | 0.78358418 | | - | |
| NBO_4g0029 | MSTRG.10054.3 | novel-m0134-3p | | | | | | | | 0.782281811 | | polar tube protein 3 [Nosema bombycis] | |
| NBO_800gi001 | MSTRG.7750.2 | novel-m0224-3p | | | | | | | | 0.781701133 | | dopey-like leucine zipper transcription factor [Nosema ceranae] | |
| NBO_547g0003 | MSTRG.5153.3 | novel-m0100-3p | | | | | | | | 0.776806913 | | hypothetical protein NBO_547g0003 [Nosema bombycis CQ1] | |
| NBO_10g0052 | MSTRG.10054.3 | novel-m0134-3p | | | | | | | | 0.775925897 | | Polar tube protein 3 [Nosema bombycis CQ1] | |
| NBO_1170gi001 | MSTRG.5153.2 | novel-m0021-5p | | | | | | | | 0.773609711 | | HSP 101 related protein, partial [Nosema bombycis CQ1] | |
| NBO_34g0010 | MSTRG.5153.1 | novel-m0021-5p | | | | | | | | 0.772935253 | | mitochondrial protein import protein MAS5 [Nosema pernyi] | |
| NBO_66g0005 | MSTRG.5153.1 | novel-m0100-3p | | | | | | | 0.766785044 | | | hypothetical protein NBO_66g0005 [Nosema bombycis CQ1] |
| NBO_4g0017 | MSTRG.5153.2 | novel-m0021-5p | | | | | | | 0.761397366 | | | Heat shock protein 101 [Nosema bombycis CQ1] |
| NBO_60g0017 | MSTRG.11899.2 | novel-m0165-3p; novel-m0072-5p | | | | | | | 0.761252564 | | | Transcription-associated protein 1 [Nosema bombycis CQ1] |
| NBO_974g0002 | MSTRG.11899.2 | novel-m0165-3p | | | | | | | 0.761037651 | | | Transcription-associated protein 1 [Nosema bombycis CQ1] |
| NBO_547g0003 | MSTRG.5153.2 | novel-m0100-3p | | | | | | | 0.759387281 | | | hypothetical protein NBO_547g0003 [Nosema bombycis CQ1] |
| NBO_423g0006 | MSTRG.2173.1 | novel-m0211-3p; novel-m0065-3p | | | | | | | 0.758056579 | | | Iron-sulfur clusters transporter atm1, mitochondrial, partial [Nosema bombycis CQ1] |
| NBO_468g0005 | MSTRG.5153.2 | novel-m0158-5p | | | | | | | 0.754476423 | | | Heat shock protein 90 [Nosema bombycis CQ1] |
| NBO_114g0004 | MSTRG.11899.2 | novel-m0072-5p | | | | | | | 0.747095122 | | | Zinc phosphodiesterase ELAC protein 2 [Nosema bombycis CQ1] |
| NBO_8g0045 | MSTRG.2923.1 | novel-m0174-5p | | | | | | | 0.744341719 | | | signal peptidase like protein [Nosema bombycis CQ1] |
| NBO_29gi002 | MSTRG.11899.2 | novel-m0165-3p | | | | | | | 0.741596137 | | | alpha,alpha trehalose-phosphate synthase [Nosema bombycis CQ1] |
| NBO_16g0027 | MSTRG.10794.1 | novel-m0148-3p | | | | | | | 0.740582031 | | | Heat shock 70 kDa protein cognate 4 [Nosema bombycis CQ1] |
| NBO_16g0023 | MSTRG.10794.1 | novel-m0148-3p | | | | | | | 0.739852447 | | | Chaperone protein dnaK [Nosema bombycis CQ1] |
| NBO_66g0005 | MSTRG.5153.3 | novel-m0100-3p | | | | | | | 0.739041843 | | | hypothetical protein NBO_66g0005 [Nosema bombycis CQ1] |
| NBO_62g0012 | MSTRG.12418.1 | novel-m0087-5p | | | | | | | 0.735978063 | | | hypothetical protein NBO_62g0012 [Nosema bombycis CQ1] |
| NBO_423g0006 | MSTRG.12418.1 | novel-m0065-3p; novel-m0211-3p | | | | | | | 0.732105664 | | | Iron-sulfur clusters transporter atm1, mitochondrial, partial [Nosema bombycis CQ1] |
| NBO_1203g0001 | MSTRG.5153.3 | novel-m0179-3p; novel-m0114-3p; novel-m0168-3p; novel-m0191-3p | | | | | | | 0.727212431 | | | hypothetical protein NBO_1203g0001 [Nosema bombycis CQ1] |
| NBO_108g0004 | MSTRG.5153.2 | novel-m0158-5p; novel-m0100-3p | | | | | | | 0.724579477 | | | Heat shock protein 90, partial [Nosema bombycis CQ1] |
| NBO_71g0003 | MSTRG.5153.1 | novel-m0100-3p; novel-m0021-5p | | | | | | | 0.721431237 | | | hypothetical protein NBO_71g0003, partial [Nosema bombycis CQ1] |
| NBO_20g0002 | MSTRG.12418.1 | novel-m0100-3p | | | | | | | 0.718000155 | | | Pescadillo like protein [Nosema bombycis CQ1] |
| NBO_915g0002 | MSTRG.5153.1 | novel-m0100-3p; novel-m0021-5p | | | | | | | 0.714006892 | | | Cell division control protein 48 E, partial [Nosema bombycis CQ1] |
| MSTRG.63 | MSTRG.12418.1 | novel-m0211-3p; novel-m0087-5p; novel-m0065-3p | | | | | | | 0.70579221 | | | hypothetical spore wall protein 14 [Nosema bombycis] |
| NBO_547g0003 | MSTRG.10794.1 | novel-m0148-3p | | | | | | | 0.704845911 | | | hypothetical protein NBO_547g0003 [Nosema bombycis CQ1] |
| NBO_6g0118 | MSTRG.10054.3 | novel-m0134-3p | | | | | | | 0.698195951 | | | hypothetical protein NBO_6g0118 [Nosema bombycis CQ1] |
| NBO_507g0012 | MSTRG.5153.1 | novel-m0109-3p; novel-m0076-3p | | | | | | | 0.696567912 | | | hypothetical protein NBO_507g0012 [Nosema bombycis CQ1] |
| NBO_20g0008 | MSTRG.5153.1 | novel-m0100-3p | | | | | | | 0.695503279 | | | peptide chain release factor 2 [Nosema ceranae] |
| NBO_16g0023 | MSTRG.5153.2 | novel-m0021-5p | | | | | | | 0.690549933 | | |  |
| NBO_66g0005 | MSTRG.5153.2 | novel-m0100-3p | | | | | | | 0.689986688 | | | hypothetical protein NBO_66g0005 [Nosema bombycis CQ1] |
| NBO_423g0006 | MSTRG.10794.1 | novel-m0148-3p | | | | | | | 0.688454803 | | | Iron-sulfur clusters transporter atm1, mitochondrial, partial [Nosema bombycis CQ1] |
| NBO_64g0028 | MSTRG.2923.1 | novel-m0174-5p | | | | | | | 0.687532634 | | | E3 ubiquitin-protein ligase [Nosema bombycis CQ1] |
| NBO_26g0007 | MSTRG.5153.2 | novel-m0100-3p | | | | | | | 0.686781389 | | | hypothetical protein NBO_26g0007 [Nosema bombycis CQ1] |
| NBO_4g0029 | MSTRG.11899.2 | novel-m0072-5p | | | | | | | 0.686314788 | | | polar tube protein 3 [Nosema bombycis] |
| NBO_26g0007 | MSTRG.5153.3 | novel-m0100-3p | | | | | | 0.685183441 | | | | hypothetical protein NBO_26g0007 [Nosema bombycis CQ1] | | |
| NBO_1291g0001 | MSTRG.7750.2 | novel-m0224-3p | | | | | | 0.68052383 | | | | Deoxyribodipyrimidine photo-lyase [Nosema bombycis CQ1] | | |
| NBO_63g0015 | MSTRG.5153.3 | novel-m0100-3p | | | | | | 0.675693959 | | | | hypothetical protein NBO_63g0015, partial [Nosema bombycis CQ1] | | |
| NBO_26g0007 | MSTRG.10794.1 | novel-m0148-3p | | | | | | 0.666911053 | | | | hypothetical protein NBO_26g0007 [Nosema bombycis CQ1] | | |
| NBO_73g0016 | MSTRG.5153.3 | novel-m0168-3p; novel-m0179-3p; novel-m0114-3p; novel-m0191-3p | | | | | | 0.664485174 | | | | hypothetical protein NBO_73g0016 [Nosema bombycis CQ1] | | |
| NBO_108g0004 | MSTRG.12418.1 | novel-m0100-3p | | | | | | 0.661752895 | | | | Heat shock protein 90, partial [Nosema bombycis CQ1] | | |
| NBO_10g0052 | MSTRG.11899.2 | novel-m0072-5p | | | | | | 0.661103693 | | | | Polar tube protein 3 [Nosema bombycis CQ1] | | |
| NBO_63g0018 | MSTRG.5153.1 | novel-m0100-3p | | | | | | 0.66031521 | | | | hypothetical protein NBO_63g0018 [Nosema bombycis CQ1] | | |
| MSTRG.63 | MSTRG.2923.1 | novel-m0065-3p; novel-m0224-3p; novel-m0211-3p | | | | | | 0.659089342 | | | | hypothetical spore wall protein 14 [Nosema bombycis] | | |
| NBO_20g0002 | MSTRG.5153.2 | novel-m0100-3p | | | | | | 0.652507511 | | | | Pescadillo like protein [Nosema bombycis CQ1] | | |
| NBO_19g0026 | MSTRG.5153.1 | novel-m0079-5p | | | | | | 0.650652131 | | | | U3 small nucleolar ribonucleoprotein imp4 [Nosema bombycis CQ1] | | |
| NBO_33gi001 | MSTRG.10054.3 | novel-m0134-3p | | | | | | 0.650412501 | | | | Protein PNS1, partial [Nosema bombycis CQ1] | | |
| NBO_53g0003 | MSTRG.5153.2 | novel-m0158-5p | | | | | | 0.650002848 | | | | hypothetical protein NBO_53g0003 [Nosema bombycis CQ1] | | |
| NBO_20g0008 | MSTRG.5153.3 | novel-m0100-3p | | | | | | 0.634731492 | | | | peptide chain release factor 2 [Nosema ceranae] | | |
| NBO_33g0017 | MSTRG.10054.3 | novel-m0134-3p | | | | | | 0.634103526 | | | | hypothetical protein NBO_33g0017 [Nosema bombycis CQ1] | | |
| NBO_9g0004 | MSTRG.2923.1 | novel-m0224-3p | | | | | | 0.633517326 | | | | hypothetical protein NBO_9g0004 [Nosema bombycis CQ1] | | |
| MSTRG.8023 | MSTRG.10054.3 | novel-m0040-5p | | | | | | 0.631486864 | | | | trans-sialidase [Cordyceps brongniartii RCEF 3172] | | |
| NBO_448g0002 | MSTRG.10794.1 | novel-m0148-3p | | | | | | 0.63063858 | | | | hypothetical protein NBO_448g0002 [Nosema bombycis CQ1] | | |
| NBO_974g0002 | MSTRG.10054.3 | novel-m0134-3p | | | | | | 0.628911386 | | | | Transcription-associated protein 1 [Nosema bombycis CQ1] | | |
| NBO_11g0038 | MSTRG.2013.1 | novel-m0174-5p | | | | | | 0.623677121 | | | | Protein kinase kin1 [Nosema bombycis CQ1] | | |
| NBO_84g0004 | MSTRG.7750.2 | novel-m0224-3p | | | | | | 0.62293595 | | | | Glycerol-3-phosphate dehydrogenase, mitochondrial [Nosema bombycis CQ1] | | |
| NBO_1015g0001 | MSTRG.5153.2 | novel-m0021-5p | | | | | | 0.621450015 | | | | U3 small nucleolar RNA-associated protein 15 [Nosema bombycis CQ1] | | |
| NBO_26g0007 | MSTRG.12418.1 | novel-m0100-3p | | | | | | 0.616728878 | | | | hypothetical protein NBO_26g0007 [Nosema bombycis CQ1] | | |
| NBO_800gi001 | MSTRG.11875.2 | novel-m0224-3p | | | | | | 0.611112686 | | | | dopey-like leucine zipper transcription factor [Nosema ceranae] | | |
| NBO_16g0051 | MSTRG.5153.1 | novel-m0100-3p | | | | | | 0.609772383 | | | | hypothetical protein NBO_16g0051 [Nosema bombycis CQ1] | | |
| NBO_915g0002 | MSTRG.5153.3 | novel-m0100-3p | | | | | | 0.606342205 | | | | Cell division control protein 48 E, partial [Nosema bombycis CQ1] | | |
| NBO_63g0015 | MSTRG.5153.2 | novel-m0021-5p; novel-m0100-3p | | | | | | 0.606292611 | | | | hypothetical protein NBO_63g0015, partial [Nosema bombycis CQ1] | | |
| NBO_73g0016 | MSTRG.5153.2 | novel-m0191-3p; novel-m0168-3p; novel-m0114-3p; novel-m0179-3p | | | | | | 0.606158814 | | | | hypothetical protein NBO_73g0016 [Nosema bombycis CQ1] | | |
| NBO_20g0002 | MSTRG.5153.3 | novel-m0100-3p | | | | | | 0.596316154 | | | | Pescadillo like protein [Nosema bombycis CQ1] | | |
| NBO_1078g0004 | MSTRG.4851.1 | novel-m0174-5p | | | | | | 0.596295523 | | | | Zinc finger C2H2 protein [Nosema bombycis CQ1] | | |
| MSTRG.7066 | MSTRG.585.1 | novel-m0158-5p | | | | | | 0.595628413 | | | | - | | |
| NBO_1015g0001 | MSTRG.5153.1 | novel-m0021-5p | | | | | | 0.592528839 | | | | U3 small nucleolar RNA-associated protein 15 [Nosema bombycis CQ1] | | |
| NBO_33g0017 | MSTRG.11875.2 | novel-m0224-3p | | | | | | 0.591695381 | | | | hypothetical protein NBO_33g0017 [Nosema bombycis CQ1] | | |
| NBO_66g0005 | MSTRG.12418.1 | novel-m0100-3p | | | | | | 0.587657516 | | | | hypothetical protein NBO_66g0005 [Nosema bombycis CQ1] | | |
| NBO_915g0002 | MSTRG.5153.2 | novel-m0100-3p; novel-m0021-5p | | | | | 0.583700571 | | | | | Cell division control protein 48 E, partial [Nosema bombycis CQ1] |
| NBO_20g0008 | MSTRG.5153.2 | novel-m0100-3p | | | | | 0.580955548 | | | | | peptide chain release factor 2 [Nosema ceranae] |
| NBO_591g0001 | MSTRG.10054.3 | novel-m0134-3p | | | | | 0.576502472 | | | | | Transketolase 1, partial [Nosema bombycis CQ1] |
| NBO_63g0018 | MSTRG.5153.3 | novel-m0100-3p | | | | | 0.57621798 | | | | | hypothetical protein NBO_63g0018 [Nosema bombycis CQ1] |
| NBO_1320g0001 | MSTRG.11875.2 | novel-m0079-5p | | | | | 0.575437716 | | | | | Hexokinase-2 [Nosema bombycis CQ1] |
| NBO_13g0067 | MSTRG.5153.1 | novel-m0079-5p | | | | | 0.57370476 | | | | | f-box domain-containing protein [Nosema ceranae] |
| NBO_389g0002 | MSTRG.2013.1 | novel-m0174-5p | | | | | 0.571095349 | | | | | hypothetical protein NBO_389g0002, partial [Nosema bombycis CQ1] |
| NBO_26g0013 | MSTRG.10054.3 | novel-m0134-3p | | | | | 0.568113978 | | | | | hypothetical spore wall protein [Nosema bombycis] |
| NBO_11g0038 | MSTRG.4851.1 | novel-m0174-5p | | | | | 0.56687483 | | | | | Protein kinase kin1 [Nosema bombycis CQ1] |
| NBO_20g0008 | MSTRG.12418.1 | novel-m0100-3p; novel-m0087-5p | | | | | 0.565012977 | | | | | peptide chain release factor 2 [Nosema ceranae] |
| NBO_80g0034 | MSTRG.4851.1 | novel-m0174-5p | | | | | 0.564622816 | | | | | Zinc finger C2H2 protein [Nosema bombycis CQ1] |
| NBO_6g0048 | MSTRG.10794.1 | novel-m0148-3p | | | | | 0.563978943 | | | | | Ricin B lectin [Nosema bombycis CQ1] |
| NBO_1078g0004 | MSTRG.2013.1 | novel-m0174-5p | | | | | 0.551598617 | | | | | Zinc finger C2H2 protein [Nosema bombycis CQ1] |
| NBO_73g0016 | MSTRG.5153.1 | novel-m0168-3p; novel-m0179-3p; novel-m0114-3p; novel-m0191-3p | | | | | 0.547862569 | | | | | hypothetical protein NBO_73g0016 [Nosema bombycis CQ1] |
| NBO_80g0034 | MSTRG.2013.1 | novel-m0174-5p | | | | | 0.546570232 | | | | | Zinc finger C2H2 protein [Nosema bombycis CQ1] |
| NBO_204g0002 | MSTRG.12418.1 | novel-m0087-5p | | | | | 0.546235306 | | | | |  |
| NBO_979g0001 | MSTRG.5153.2 | novel-m0179-3p; novel-m0114-3p; novel-m0168-3p; novel-m0191-3p | | | | | 0.529123908 | | | | | transcription initiation factor brf1 subunit-like protein [Nosema ceranae] |
| NBO_63g0015 | MSTRG.5153.1 | novel-m0021-5p; novel-m0100-3p | | | | | 0.524928401 | | | | | hypothetical protein NBO_63g0015, partial [Nosema bombycis CQ1] |
| NBO_53g0003 | MSTRG.5153.1 | novel-m0158-5p | | | | | 0.52235126 | | | | | hypothetical protein NBO_53g0003 [Nosema bombycis CQ1] |
| NBO_63g0018 | MSTRG.5153.2 | novel-m0100-3p | | | | | 0.521147967 | | | | | hypothetical protein NBO_63g0018 [Nosema bombycis CQ1] |
| NBO_1276g0001 | MSTRG.2923.1 | novel-m0224-3p | | | | | 0.518879356 | | | | | tRNA acetyltransferase TAN1 [Nematocida displodere] |
| NBO_16g0051 | MSTRG.5153.3 | novel-m0100-3p | | | | | 0.518170291 | | | | | hypothetical protein NBO_16g0051 [Nosema bombycis CQ1] |
| NBO_71g0003 | MSTRG.5153.2 | novel-m0100-3p; novel-m0021-5p | | | | | 0.517757185 | | | | | hypothetical protein NBO_71g0003, partial [Nosema bombycis CQ1] |
| NBO_915g0002 | MSTRG.12418.1 | novel-m0100-3p | | | | | 0.517211352 | | | | | Cell division control protein 48 E, partial [Nosema bombycis CQ1] |
| MSTRG.63 | MSTRG.1660.2 | novel-m0211-3p; novel-m0065-3p | | | | | 0.510488986 | | | | | hypothetical spore wall protein 14 [Nosema bombycis] |
| NBO_1000gi001 | MSTRG.10054.3 | novel-m0134-3p | | | | | 0.509581499 | | | | | Protein PNS1, partial [Nosema bombycis CQ1] |
| NBO_920g0001 | MSTRG.10794.1 | novel-m0148-3p | | | | | 0.505903503 | | | | | Myosin heavy chain kinase B [Nosema bombycis CQ1] |
| NBO_166g0002 | MSTRG.10054.3 | novel-m0134-3p | | | | | 0.505412651 | | | | | Transcription-associated protein 1, partial [Nosema bombycis CQ1] |
| MSTRG.63 | MSTRG.4289.1 | novel-m0211-3p; novel-m0065-3p | | | | | 0.504941323 | | | | | hypothetical spore wall protein 14 [Nosema bombycis] |
| NBO_10g0052 | MSTRG.11875.2 | novel-m0224-3p; novel-m0206-5p | | | | | 0.502831044 | | | | | Polar tube protein 3 [Nosema bombycis CQ1] |
| MSTRG.7066 | MSTRG.2923.1 | novel-m0224-3p | | | | 0.499244424 | | | | | | - |
| NBO_71g0003 | MSTRG.12418.1 | novel-m0100-3p | | | | 0.499110758 | | | | | | hypothetical protein NBO_71g0003, partial [Nosema bombycis CQ1] |
| NBO_1010g0001 | MSTRG.5153.1 | novel-m0021-5p | | | | 0.496381929 | | | | | | Pescadillo-like protein [Nosema bombycis CQ1] |
| NBO_53g0003 | MSTRG.5153.3 | novel-m0158-5p | | | | 0.494935611 | | | | | | hypothetical protein NBO_53g0003 [Nosema bombycis CQ1] |
| MSTRG.2926 | MSTRG.5153.2 | novel-m0191-3p; novel-m0114-3p; novel-m0179-3p; novel-m0168-3p | | | | 0.494289362 | | | | | | transposase [Nosema bombycis] |
| NBO_979g0001 | MSTRG.5153.3 | novel-m0179-3p; novel-m0114-3p; novel-m0168-3p; novel-m0191-3p | | | | 0.493408844 | | | | | | transcription initiation factor brf1 subunit-like protein [Nosema ceranae] |
| NBO_4g0048 | MSTRG.10054.3 | novel-m0134-3p | | | | 0.48946041 | | | | | | hypothetical protein NBO_4g0048 [Nosema bombycis CQ1] |
| NBO_71g0003 | MSTRG.5153.3 | novel-m0100-3p | | | | 0.487979608 | | | | | | hypothetical protein NBO_71g0003, partial [Nosema bombycis CQ1] |
| NBO_4g0029 | MSTRG.11875.2 | novel-m0206-5p; novel-m0224-3p | | | | 0.485722645 | | | | | | polar tube protein 3 [Nosema bombycis] |
| NBO_1010g0001 | MSTRG.5153.2 | novel-m0021-5p | | | | 0.484431083 | | | | | | Pescadillo-like protein [Nosema bombycis CQ1] |
| NBO_16g0051 | MSTRG.5153.2 | novel-m0100-3p | | | | 0.474384457 | | | | | | hypothetical protein NBO_16g0051 [Nosema bombycis CQ1] |
| NBO_28g0046 | MSTRG.5153.2 | novel-m0021-5p | | | | 0.470359267 | | | | | |  |
| NBO_389g0002 | MSTRG.4851.1 | novel-m0174-5p | | | | 0.468026038 | | | | | | hypothetical protein NBO_389g0002, partial [Nosema bombycis CQ1] |
| NBO_423g0006 | MSTRG.2923.1 | novel-m0224-3p; novel-m0065-3p; novel-m0211-3p | | | | 0.462058278 | | | | | | Iron-sulfur clusters transporter atm1, mitochondrial, partial [Nosema bombycis CQ1] |
| NBO_6g0040 | MSTRG.5153.3 | novel-m0179-3p; novel-m0114-3p; novel-m0168-3p; novel-m0191-3p | | | | 0.459497411 | | | | | | hypothetical protein NBO_6g0040 [Nosema bombycis CQ1] |
| NBO_3g0006 | MSTRG.11875.2 | novel-m0224-3p | | | | 0.454489996 | | | | | | 60S ribosomal protein L6, partial [Nosema bombycis] |
| NBO_1116g0004 | MSTRG.11875.2 | novel-m0224-3p | | | | 0.453581737 | | | | | | hypothetical protein NBO_1116g0004 [Nosema bombycis CQ1] |
| NBO_375g0009 | MSTRG.4851.1 | novel-m0174-5p | | | | 0.453026464 | | | | | | 2,3-bisphosphoglycerate-independent phosphoglycerate mutase [Nosema bombycis CQ1] |
| NBO_36g0002 | MSTRG.10794.1 | novel-m0148-3p | | | | 0.451212975 | | | | | | Homeobox protein HD-8 [Nosema bombycis CQ1] |
| NBO_375g0009 | MSTRG.2013.1 | novel-m0174-5p | | | | 0.44520967 | | | | | | 2,3-bisphosphoglycerate-independent phosphoglycerate mutase [Nosema bombycis CQ1] |
| NBO_11g0038 | MSTRG.2923.1 | novel-m0174-5p | | | | 0.438326218 | | | | | | Protein kinase kin1 [Nosema bombycis CQ1] |
| NBO_4g0048 | MSTRG.5153.1 | novel-m0021-5p | | | | 0.437757773 | | | | | | hypothetical protein NBO_4g0048 [Nosema bombycis CQ1] |
| MSTRG.7066 | MSTRG.5153.1 | novel-m0158-5p | | | | 0.433766167 | | | | | | - |
| NBO_63g0018 | MSTRG.12418.1 | novel-m0100-3p | | | | 0.431697191 | | | | | | hypothetical protein NBO_63g0018 [Nosema bombycis CQ1] |
| NBO_6g0040 | MSTRG.11899.2 | novel-m0072-5p | | | | 0.427112233 | | | | | | hypothetical protein NBO_6g0040 [Nosema bombycis CQ1] |
| NBO_80g0034 | MSTRG.2923.1 | novel-m0174-5p | | | | 0.42290471 | | | | | | Zinc finger C2H2 protein [Nosema bombycis CQ1] |
| NBO_16g0051 | MSTRG.12418.1 | novel-m0100-3p | | | | 0.417981701 | | | | | | hypothetical protein NBO_16g0051 [Nosema bombycis CQ1] |
| MSTRG.63 | MSTRG.3258.1 | novel-m0211-3p; novel-m0065-3p | | | | 0.417311984 | | | | | | hypothetical spore wall protein 14 [Nosema bombycis] |
| NBO_1078g0004 | MSTRG.2923.1 | novel-m0174-5p | | | | 0.417184504 | | | | | | Zinc finger C2H2 protein [Nosema bombycis CQ1] |
| NBO_6g0030 | MSTRG.5153.1 | novel-m0079-5p | | | | 0.409681009 | | | | | | ATP-dependent DNA helicase hus2/rqh1 [Nosema bombycis CQ1] |
| NBO_979g0001 | MSTRG.5153.1 | novel-m0191-3p; novel-m0168-3p; novel-m0114-3p; novel-m0179-3p | | | 0.404634101 | | | | | | | transcription initiation factor brf1 subunit-like protein [Nosema ceranae] |
| NBO_444g0008 | MSTRG.11875.2 | novel-m0224-3p | | | 0.404566596 | | | | | | | Cysteinyl-tRNA synthetase, cytoplasmic [Nosema bombycis CQ1] |
| NBO_6g0049 | MSTRG.2236.1 | novel-m0177-3p | | | 0.404388143 | | | | | | | Ricin B lectin [Nosema bombycis CQ1] |
| NBO_19g0014 | MSTRG.11875.2 | novel-m0206-5p | | | 0.382753883 | | | | | | | hypothetical protein NBO_19g0014 [Nosema bombycis CQ1] |
| NBO_13g0057 | MSTRG.5153.3 | novel-m0100-3p | | | 0.380327804 | | | | | | | transcription factor TAU-like protein [Nosema bombycis CQ1] |
| NBO_3g0004 | MSTRG.11875.2 | novel-m0224-3p | | | 0.380018734 | | | | | | | hypothetical protein NBO_3g0004 [Nosema bombycis CQ1] |
| NBO_3g0033 | MSTRG.2923.1 | novel-m0224-3p | | | 0.376098424 | | | | | | | hypothetical protein NBO_3g0033 [Nosema bombycis CQ1] |
| MSTRG.2926 | MSTRG.5153.3 | novel-m0191-3p; novel-m0168-3p; novel-m0114-3p; novel-m0179-3p | | | 0.367334854 | | | | | | | transposase [Nosema bombycis] |
| NBO_389g0002 | MSTRG.2923.1 | novel-m0174-5p; novel-m0224-3p | | | 0.357879937 | | | | | | | hypothetical protein NBO_389g0002, partial [Nosema bombycis CQ1] |
| NBO_13g0057 | MSTRG.5153.1 | novel-m0100-3p | | | 0.35773902 | | | | | | | transcription factor TAU-like protein [Nosema bombycis CQ1] |
| NBO_11g0041 | MSTRG.11875.2 | novel-m0224-3p | | | 0.356781155 | | | | | | small-conductance mechanosensitive channel protein [Nosema ceranae] | |
| NBO_6g0004 | MSTRG.2923.1 | novel-m0224-3p | | | 0.354316954 | | | | | | hypothetical protein NBO_6g0004 [Nosema bombycis CQ1] | |
| NBO_78g0012 | MSTRG.10054.3 | novel-m0134-3p | | | 0.353879686 | | | | | | ATP-binding cassette sub-family B member 7, mitochondrial [Nosema bombycis CQ1] | |
| NBO_389g0001 | MSTRG.10054.3 | novel-m0134-3p | | | 0.351588326 | | | | | | U3 small nucleolar RNA-associated protein 12 [Nosema bombycis CQ1] | |
| MSTRG.4051 | MSTRG.12418.1 | novel-m0087-5p | | | 0.348965337 | | | | | | - | |
| NBO_375g0009 | MSTRG.7750.2 | novel-m0224-3p | | | 0.342695857 | | | | | | 2,3-bisphosphoglycerate-independent phosphoglycerate mutase [Nosema bombycis CQ1] | |
| NBO_63g0015 | MSTRG.12418.1 | novel-m0100-3p | | | 0.34191359 | | | | | | hypothetical protein NBO_63g0015, partial [Nosema bombycis CQ1] | |
| MSTRG.8131 | MSTRG.12418.1 | novel-m0211-3p; novel-m0065-3p | | | 0.341780808 | | | | | | pol polyprotein [Nosema bombycis] | |
| NBO_423g0006 | MSTRG.1660.2 | novel-m0065-3p; novel-m0211-3p | | | 0.341326337 | | | | | | Iron-sulfur clusters transporter atm1, mitochondrial, partial [Nosema bombycis CQ1] | |
| NBO_1133g0001 | MSTRG.11875.2 | novel-m0079-5p | | | 0.334784034 | | | | | | Ricin B lectin [Nosema bombycis CQ1] | |
| NBO_1332gi002 | MSTRG.11875.2 | novel-m0224-3p | | | 0.327937301 | | | | | | threonyl-tRNA synthetase [Nosema bombycis CQ1] | |
| NBO_71g0002 | MSTRG.10054.3 | novel-m0134-3p | | | 0.319360042 | | | | | | isoleucyl-tRNA synthetase, cytoplasmic [Nosema bombycis CQ1] | |
| NBO_423g0006 | MSTRG.4289.1 | novel-m0211-3p; novel-m0065-3p | | | 0.316176276 | | | | | | Iron-sulfur clusters transporter atm1, mitochondrial, partial [Nosema bombycis CQ1] | |
| NBO_84g0004 | MSTRG.11875.2 | novel-m0224-3p | | | 0.313625617 | | | | | | Glycerol-3-phosphate dehydrogenase, mitochondrial [Nosema bombycis CQ1] | |
| MSTRG.14183 | MSTRG.11875.2 | novel-m0206-5p | | | 0.309928628 | | | | | | - | |
| NBO_375g0009 | MSTRG.2923.1 | novel-m0224-3p; novel-m0174-5p | | | 0.305478627 | | | | | | 2,3-bisphosphoglycerate-independent phosphoglycerate mutase [Nosema bombycis CQ1] | |
| NBO_31gi002 | MSTRG.11875.2 | novel-m0224-3p | | | 0.303930212 | | | | | | small-conductance mechanosensitive channel protein [Nosema ceranae] | |
| NBO_362g0011 | MSTRG.10054.3 | novel-m0134-3p | | | 0.303839352 | | | | | | CDP-diacylglycerol--inositol 3-phosphatidyltransferase [Nosema bombycis CQ1] | |
| NBO_1291g0001 | MSTRG.11875.2 | novel-m0224-3p | | | 0.301203288 | | | | | | Deoxyribodipyrimidine photo-lyase [Nosema bombycis CQ1] | |
| NBO_568g0001 | MSTRG.10054.3 | novel-m0134-3p | | | 0.292479629 | | | | | | Tristetraproline [Nosema bombycis CQ1] | |
| NBO_69g0015 | MSTRG.11875.2 | novel-m0224-3p | | | 0.28459273 | | | | | | serine threonine protein kinase [Nosema ceranae] | |
| NBO_76g0014 | MSTRG.7750.2 | novel-m0224-3p | | | 0.276820192 | | | | | | hypothetical protein NBO_76g0014 [Nosema bombycis CQ1] | |
| NBO_76g0014 | MSTRG.5153.1 | novel-m0021-5p | 0.27601896 | | | | | | | | hypothetical protein NBO_76g0014 [Nosema bombycis CQ1] | |
| NBO_13g0057 | MSTRG.5153.2 | novel-m0100-3p | 0.267734994 | | | | | | | | transcription factor TAU-like protein [Nosema bombycis CQ1] | |
| NBO_1566g0002 | MSTRG.5153.1 | novel-m0021-5p | 0.264885106 | | | | | | | | Deoxyhypusine hydroxylase [Nosema bombycis CQ1] | |
| NBO_10g0053 | MSTRG.2923.1 | novel-m0224-3p | 0.263612977 | | | | | | | | DNA mismatch repair protein Mlh1 [Nosema bombycis CQ1] | |
| NBO_4g0050 | MSTRG.10054.3 | novel-m0134-3p | 0.261763043 | | | | | | | | Insulin-degrading enzyme [Nosema bombycis CQ1] | |
| NBO_3g0016 | MSTRG.5153.3 | novel-m0100-3p | 0.258593007 | | | | | | | | hypothetical protein NBO_3g0016 [Nosema bombycis CQ1] | |
| NBO_448g0002 | MSTRG.11875.2 | novel-m0224-3p | 0.244674566 | | | | | | | | hypothetical protein NBO_448g0002 [Nosema bombycis CQ1] | |
| NBO_468g0005 | MSTRG.585.1 | novel-m0158-5p | | 0.241347621 | | | | | | | Heat shock protein 90 [Nosema bombycis CQ1] | |
| NBO_19g0014 | MSTRG.4851.1 | novel-m0174-5p | | 0.238900982 | | | | | | | hypothetical protein NBO_19g0014 [Nosema bombycis CQ1] | |
| MSTRG.8131 | MSTRG.2173.1 | novel-m0065-3p; novel-m0211-3p | | 0.235894609 | | | | | | | pol polyprotein [Nosema bombycis] | |
| MSTRG.2926 | MSTRG.5153.1 | novel-m0191-3p; novel-m0179-3p; novel-m0114-3p; novel-m0168-3p | | 0.234777978 | | | | | | | transposase [Nosema bombycis] | |
| NBO_108g0004 | MSTRG.585.1 | novel-m0158-5p | | 0.230476754 | | | | | | | Heat shock protein 90, partial [Nosema bombycis CQ1] | |
| NBO_53g0003 | MSTRG.11875.2 | novel-m0224-3p | | 0.225263249 | | | | | | | hypothetical protein NBO_53g0003 [Nosema bombycis CQ1] | |
| NBO_6g0040 | MSTRG.5153.2 | novel-m0191-3p; novel-m0114-3p; novel-m0179-3p; novel-m0168-3p | | 0.224353923 | | | | | | | hypothetical protein NBO_6g0040 [Nosema bombycis CQ1] | |
| NBO_6g0040 | MSTRG.5153.1 | novel-m0179-3p; novel-m0114-3p; novel-m0168-3p; novel-m0191-3p | | 0.223254328 | | | | | | | hypothetical protein NBO_6g0040 [Nosema bombycis CQ1] | |
| NBO_19g0014 | MSTRG.2013.1 | novel-m0174-5p | | 0.213700357 | | | | | | | hypothetical protein NBO_19g0014 [Nosema bombycis CQ1] | |
| NBO_423g0006 | MSTRG.3258.1 | novel-m0211-3p; novel-m0065-3p | | 0.205324811 | | | | | | | Iron-sulfur clusters transporter atm1, mitochondrial, partial [Nosema bombycis CQ1] | |
| NBO_13g0057 | MSTRG.12418.1 | novel-m0100-3p; novel-m0087-5p | | 0.170662737 | | | | | | | transcription factor TAU-like protein [Nosema bombycis CQ1] | |
| NBO_76g0014 | MSTRG.5153.2 | novel-m0021-5p | | 0.16868183 | | | | | | | hypothetical protein NBO_76g0014 [Nosema bombycis CQ1] | |
| NBO_3g0016 | MSTRG.5153.2 | novel-m0100-3p | | 0.168394803 | | | | | | | hypothetical protein NBO_3g0016 [Nosema bombycis CQ1] | |
| NBO_9g0004 | MSTRG.5153.1 | novel-m0076-3p; novel-m0109-3p | | 0.157428938 | | | | | | | hypothetical protein NBO_9g0004 [Nosema bombycis CQ1] | |
| NBO_464g0008 | MSTRG.10054.3 | novel-m0134-3p | | 0.149851566 | | | | | | | Isoleucyl-tRNA synthetase [Nosema bombycis CQ1] | |
| NBO_84g0004 | MSTRG.4851.1 | novel-m0174-5p | | 0.147299503 | | | | | | | Glycerol-3-phosphate dehydrogenase, mitochondrial [Nosema bombycis CQ1] | |
| NBO_71g0003 | MSTRG.11899.2 | novel-m0165-3p | | 0.138508687 | | | | | | | hypothetical protein NBO_71g0003, partial [Nosema bombycis CQ1] | |
| NBO_43g0006 | MSTRG.5153.1 | novel-m0021-5p | | 0.136138374 | | | | | | | ATP-dependent RNA helicase DHX37 [Nosema bombycis CQ1] | |
| NBO_58g0026 | MSTRG.2923.1 | novel-m0224-3p | | 0.133935062 | | | | | | | GPN-loop GTPase 1 [Nosema bombycis CQ1] | |
| NBO_28g0056 | MSTRG.10054.3 | novel-m0134-3p | | 0.130234236 | | | | | | | hypothetical protein NBO_28g0056 [Nosema bombycis CQ1] | |
| NBO_1170gi001 | MSTRG.2923.1 | novel-m0174-5p | | 0.128024766 | | | | | | | HSP 101 related protein, partial [Nosema bombycis CQ1] | |
| NBO_6g0112 | MSTRG.10054.3 | novel-m0134-3p | | 0.122990603 | | | | | | | hypothetical protein NBO_6g0112 [Nosema bombycis CQ1] | |
| NBO_417g0015 | MSTRG.12418.1 | novel-m0087-5p | | 0.117168445 | | | | | | | ADP-ribosylation factor 1 [Nosema bombycis CQ1] | |
| NBO_1302g0001 | MSTRG.4851.1 | novel-m0174-5p | | 0.116581185 | | | | | | | hypothetical protein NBO_1302g0001 [Nosema bombycis CQ1] | |
| NBO_19g0014 | MSTRG.2923.1 | novel-m0174-5p | | 0.11377276 | | | | | | | hypothetical protein NBO_19g0014 [Nosema bombycis CQ1] | |
| NBO_6g0085 | MSTRG.12084.1 | novel-m0148-3p | | 0.110697171 | | | | | | | 40S ribosomal protein S10 [Amphiamblys sp. WSBS2006] | |
| NBO_1566g0002 | MSTRG.5153.2 | novel-m0021-5p | | 0.10587058 | | | | | | | Deoxyhypusine hydroxylase [Nosema bombycis CQ1] | |
| NBO_1203g0001 | MSTRG.2923.1 | novel-m0224-3p | | 0.096039845 | | | | | | | hypothetical protein NBO_1203g0001 [Nosema bombycis CQ1] | |
| NBO_46g0002 | MSTRG.5153.2 | novel-m0168-3p; novel-m0179-3p; novel-m0114-3p; novel-m0191-3p | | 0.09004279 | | | | | | | hypothetical protein NBO_46g0002 [Nosema bombycis CQ1] | |
| NBO_1302g0001 | MSTRG.2013.1 | novel-m0174-5p | | 0.087681362 | | | | | | | hypothetical protein NBO_1302g0001 [Nosema bombycis CQ1] | |
| NBO_84g0004 | MSTRG.2013.1 | novel-m0174-5p | | 0.084900521 | | | | | | | Glycerol-3-phosphate dehydrogenase, mitochondrial [Nosema bombycis CQ1] | |
| NBO_4g0017 | MSTRG.2923.1 | novel-m0174-5p | | 0.084303252 | | | | | | | Heat shock protein 101 [Nosema bombycis CQ1] | |
| NBO_937g0002 | MSTRG.5153.1 | novel-m0021-5p | | 0.081486665 | | | | | | | Sec61beta [Nosema bombycis CQ1] | |
| NBO_53g0003 | MSTRG.7750.2 | novel-m0224-3p | | 0.075231345 | | | | | | | hypothetical protein NBO_53g0003 [Nosema bombycis CQ1] | |
| NBO_46g0002 | MSTRG.5153.3 | novel-m0114-3p; novel-m0179-3p; novel-m0168-3p; novel-m0191-3p | | 0.063034547 | | | | | | | hypothetical protein NBO_46g0002 [Nosema bombycis CQ1] | |
| NBO_396g0002 | MSTRG.12418.1 | novel-m0087-5p | | 0.055783407 | | | | | | | TAF4 transcription initiation factor TFIID component [Encephalitozoon intestinalis ATCC 50506] | |
| NBO_16g0023 | MSTRG.2923.1 | novel-m0224-3p | | 0.054700103 | | | | | | | Chaperone protein dnaK [Nosema bombycis CQ1] | |
| NBO_4g0048 | MSTRG.5153.2 | novel-m0021-5p | | 0.049341643 | | | | | | | hypothetical protein NBO_4g0048 [Nosema bombycis CQ1] | |
| NBO_76g0014 | MSTRG.11875.2 | novel-m0224-3p | | 0.028949666 | | | | | | | hypothetical protein NBO_76g0014 [Nosema bombycis CQ1] | |
| NBO_3g0016 | MSTRG.12418.1 | novel-m0100-3p | | 0.028067938 | | | | | | | hypothetical protein NBO_3g0016 [Nosema bombycis CQ1] | |
| NBO_1302g0001 | MSTRG.2923.1 | novel-m0174-5p | | 0.026528032 | | | | | | | hypothetical protein NBO_1302g0001 [Nosema bombycis CQ1] | |
| NBO_63g0018 | MSTRG.7750.2 | novel-m0224-3p | | 0.025201479 | | | | | | | hypothetical protein NBO_63g0018 [Nosema bombycis CQ1] | |
| NBO_28g0025 | MSTRG.10054.3 | novel-m0134-3p | | 0.02023751 | | | | | | | Uridine kinase | |
| NBO_20g0008 | MSTRG.10054.3 | novel-m0134-3p | | 0.015264043 | | | | | | | peptide chain release factor 2 [Nosema ceranae] | |
| NBO_13g0067 | MSTRG.2923.1 | novel-m0224-3p; novel-m0174-5p | | 0.009719837 | | | | | | | f-box domain-containing protein [Nosema ceranae] | |
| NBO_53g0003 | MSTRG.585.1 | novel-m0158-5p | | 0.002121078 | | | | | | | hypothetical protein NBO_53g0003 [Nosema bombycis CQ1] | |

| Table S7 **CircRNA -miRNA-mRNA ceRNA networks of *N. bombycis* in the microsporidia congenitally infected silkworm embryos and larvae** | | | | |
| --- | --- | --- | --- | --- |
| mRNA | circRNA | miRNA | cor | description |
| NBO_28g0019 | novel_circ_000006 | novel-m0209-5p; novel-m0185-5p; novel-m0221-5p | 0.447208462 | DNA-directed RNA polymerases I and III subunit RPAC1 [Nosema bombycis CQ1] |
| NBO_4g0048 | novel_circ_000006 | novel-m0044-3p; novel-m0046-3p | 0.039738076 | hypothetical protein NBO_4g0048 [Nosema bombycis CQ1] |

| Table S8 **The connection degree of each gene of *N. bombycis* in the ceRNA networks** | | |
| --- | --- | --- |
| ID | Type | Connection degree |
| novel-m0224-3p | miRNA | 8 |
| novel-m0211-3p | miRNA | 8 |
| novel-m0065-3p | miRNA | 8 |
| novel-m0158-5p | miRNA | 6 |
| novel-m0134-3p | miRNA | 5 |
| novel-m0100-3p | miRNA | 5 |
| novel-m0191-3p | miRNA | 4 |
| novel-m0179-3p | miRNA | 4 |
| novel-m0174-5p | miRNA | 4 |
| novel-m0168-3p | miRNA | 4 |
| novel-m0148-3p | miRNA | 4 |
| novel-m0114-3p | miRNA | 4 |
| novel-m0206-5p | miRNA | 3 |
| novel-m0072-5p | miRNA | 3 |
| novel-m0221-5p | miRNA | 2 |
| novel-m0209-5p | miRNA | 2 |
| novel-m0185-5p | miRNA | 2 |
| novel-m0177-3p | miRNA | 2 |
| novel-m0087-5p | miRNA | 2 |
| novel-m0079-5p | miRNA | 2 |
| MSTRG.2926 | mRNA | 5 |
| NBO_4g0029 | mRNA | 4 |
| NBO_423g0006 | mRNA | 4 |
| NBO_10g0052 | mRNA | 4 |
| MSTRG.63 | mRNA | 4 |
| NBO_28g0019 | mRNA | 3 |
| NBO_6g0049 | mRNA | 2 |
| NBO_108g0004 | mRNA | 2 |
| NBO_78g0012 | mRNA | 1 |
| NBO_6g0048 | mRNA | 1 |
| NBO_468g0005 | mRNA | 1 |
| NBO_26g0013 | mRNA | 1 |
| NBO_1133g0001 | mRNA | 1 |
| MSTRG.128 | mRNA | 1 |
| MSTRG.5153.3 | lncRNA | 6 |
| MSTRG.5153.2 | lncRNA | 6 |
| MSTRG.5153.1 | lncRNA | 6 |
| MSTRG.2923.1 | lncRNA | 4 |
| MSTRG.12418.1 | lncRNA | 4 |
| novel_circ_000006 | lncRNA | 3 |
| MSTRG.11875.2 | lncRNA | 3 |
| MSTRG.4289.1 | lncRNA | 2 |
| MSTRG.3258.1 | lncRNA | 2 |
| MSTRG.2173.1 | lncRNA | 2 |
| MSTRG.1660.2 | lncRNA | 2 |
| MSTRG.7750.2 | lncRNA | 1 |
| MSTRG.585.1 | lncRNA | 1 |
| MSTRG.4851.1 | lncRNA | 1 |
| MSTRG.2236.1 | lncRNA | 1 |
| MSTRG.2013.1 | lncRNA | 1 |
| MSTRG.11899.2 | lncRNA | 1 |
| MSTRG.10794.1 | lncRNA | 1 |
| MSTRG.10054.3 | lncRNA | 1 |

| **Table S9 Novel genes of silkworm were identified in the microsporidia congenital infection in silkworm embryos and larvae** | | | | | | | | | | | | | | | | | | | |
| --- | --- | --- | --- | --- | --- | --- | --- | --- | --- | --- | --- | --- | --- | --- | --- | --- | --- | --- | --- |
| GeneID | Symbol | Description | | | | | | | | | | | | | | | | | |
| MSTRG.10016 | -- | PREDICTED: uncharacterized protein LOC105841941 [Bombyx mori] | | | | | | | | | | | | | | | | | |
| MSTRG.10031 | -- | PREDICTED: mitochondrial ribonuclease P protein 3 [Bombyx mori] | | | | | | | | | | | | | | | | | |
| MSTRG.10036 | -- | PREDICTED: dual specificity protein kinase splB-like [Bombyx mori] | | | | | | | | | | | | | | | | | |
| MSTRG.10043 | -- | - | | | | | | | | | | | | | | | | | |
| MSTRG.10044 | -- | - | | | | | | | | | | | | | | | | | |
| MSTRG.10072 | -- | PREDICTED: general odorant-binding protein 99a isoform X1 [Bombyx mori] | | | | | | | | | | | | | | | | | |
| MSTRG.10074 | -- | odorant-binding protein 1 [Bombyx mori] | | | | | | | | | | | | | | | | | |
| MSTRG.10077 | -- | PREDICTED: odorant-binding protein 4 isoform X1 [Bombyx mori] | | | | | | | | | | | | | | | | | |
| MSTRG.10093 | Tubgcp6 | PREDICTED: gamma-tubulin complex component 6 [Amyelois transitella] | | | | | | | | | | | | | | | | | |
| MSTRG.10102 | -- | uncharacterized LOC106101566 precursor [Papilio polytes] | | | | | | | | | | | | | | | | | |
| MSTRG.10115 | Psma3 | proteasome alpha 3 subunit [Bombyx mori] | | | | | | | | | | | | | | | | | |
| MSTRG.10120 | fend | PREDICTED: uncharacterized protein LOC101746296 [Bombyx mori] | | | | | | | | | | | | | | | | | |
| MSTRG.10131 | -- | PREDICTED: uncharacterized protein LOC101746574 [Bombyx mori] | | | | | | | | | | | | | | | | | |
| MSTRG.10133 | -- | PREDICTED: uncharacterized protein LOC101746715 [Bombyx mori] | | | | | | | | | | | | | | | | | |
| MSTRG.10138 | ALKBH1 | PREDICTED: alkylated DNA repair protein alkB homolog 1 isoform X1 [Bombyx mori] | | | | | | | | | | | | | | | | | |
| MSTRG.10141 | Mthfr | PREDICTED: methylenetetrahydrofolate reductase [Amyelois transitella] | | | | | | | | | | | | | | | | | |
| MSTRG.10149 | Drosha | PREDICTED: ribonuclease 3 [Bombyx mori] | | | | | | | | | | | | | | | | | |
| MSTRG.10150 | Drosha | PREDICTED: ribonuclease 3 [Bombyx mori] | | | | | | | | | | | | | | | | | |
| MSTRG.10157 | Bahd1 | Bromo adjacent-likey domain-containing 1 protein [Papilio machaon] | | | | | | | | | | | | | | | | | |
| MSTRG.10158 | Bahd1 | Bromo adjacent-likey domain-containing 1 protein [Papilio machaon] | | | | | | | | | | | | | | | | | |
| MSTRG.10167 | AAEL001134 | PREDICTED: probable methylmalonate-semialdehyde dehydrogenase [acylating], mitochondrial isoform X1 [Bombyx mori] | | | | | | | | | | | | | | | | | |
| MSTRG.10178 | nkx2.2a | PREDICTED: homeobox protein Nkx-2.2a isoform X2 [Bombyx mori] | | | | | | | | | | | | | | | | | |
| MSTRG.10182 | VPS39 | PREDICTED: vam6/Vps39-like protein [Bombyx mori] | | | | | | | | | | | | | | | | | |
| MSTRG.10196 | PSMC4 | 26S protease regulatory subunit 6B [Bombyx mori] | | | | | | | | | | | | | | | | | |
| MSTRG.10207 | Jon99Cii | PREDICTED: collagenase-like [Bombyx mori] | | | | | | | | | | | | | | | | | |
| MSTRG.10215 | gag-pro-pol | Transposon Ty3-G Gag-Pol polyprotein [Exaiptasia pallida] | | | | | | | | | | | | | | | | | |
| MSTRG.10235 | -- | PREDICTED: uncharacterized protein LOC101736009 [Bombyx mori] | | | | | | | | | | | | | | | | | |
| MSTRG.10238 | -- | PREDICTED: myb-like protein I [Amyelois transitella] | | | | | | | | | | | | | | | | | |
| MSTRG.10249 | -- | PREDICTED: proline-rich protein 11-like [Amyelois transitella] | | | | | | | | | | | | | | | | | |
| MSTRG.10254 | Nprl3 | PREDICTED: nitrogen permease regulator 3-like protein [Bombyx mori] | | | | | | | | | | | | | | | | | |
| MSTRG.10267 | TY3B-I | PREDICTED: uncharacterized protein K02A2.6-like [Papilio polytes] | | | | | | | | | | | | | | | | | |
| MSTRG.10273 | GlcAT-I | PREDICTED: galactosylgalactosylxylosylprotein 3-beta-glucuronosyltransferase I [Bombyx mori] | | | | | | | | | | | | | | | | | |
| MSTRG.10278 | Fas2 | PREDICTED: fasciclin-2-like [Bombyx mori] | | | | | | | | | | | | | | | | | |
| MSTRG.10291 | SORL1 | PREDICTED: sortilin-related receptor-like isoform X2 [Papilio xuthus] | | | | | | | | | | | | | | | | | |
| MSTRG.10292 | -- | PREDICTED: ankyrin-3-like [Amyelois transitella] | | | | | | | | | | | | | | | | | |
| MSTRG.10296 | RpS19a | ribosomal protein S19 [Bombyx mori] | | | | | | | | | | | | | | | | | |
| MSTRG.10308 | pol | Transposon Tf2-9 polyprotein-like Protein [Tribolium castaneum] | | | | | | | | | | | | | | | | | |
| MSTRG.10325 | DnaJ-60 | DnaJ (Hsp40) homolog 13 [Bombyx mori] | | | | | | | | | | | | | | | | | |
| MSTRG.10332 | CTRL | PREDICTED: brachyurin-like [Bombyx mori] | | | | | | | | | | | | | | | | | |
| MSTRG.10347 | -- | PREDICTED: zinc finger protein 808-like isoform X1 [Amyelois transitella] | | | | | | | | | | | | | | | | | |
| MSTRG.10398 | pol | Transposon Tf2-9 polyprotein-like Protein [Tribolium castaneum] | | | | | | | | | | | | | | | | | |
| MSTRG.10399 | -- | Pro-Pol polyprotein [Habropoda laboriosa] | | | | | | | | | | | | | | | | | |
| MSTRG.10413 | -- | single VWC domain protein 2, partial [Pararge aegeria] | | | | | | | | | | | | | | | | | |
| MSTRG.10514 | RTase | reverse transcriptase [Bombyx mori] | | | | | | | | | | | | | | | | | |
| MSTRG.1056 | pol | PREDICTED: aquaporin-like isoform X2 [Bombyx mori] | | | | | | | | | | | | | | | | | |
| MSTRG.10599 | PCP36 | cuticular protein RR-1 motif 52 [Bombyx mori] | | | | | | | | | | | | | | | | | |
| MSTRG.1060 | Bap60 | PREDICTED: brahma-associated protein of 60 kDa, partial [Bombyx mori] | | | | | | | | | | | | | | | | | |
| MSTRG.10609 | ACAA2 | PREDICTED: 3-ketoacyl-CoA thiolase, mitochondrial-like [Bombyx mori] | | | | | | | | | | | | | | | | | |
| MSTRG.10610 | ACAA2 | PREDICTED: 3-ketoacyl-CoA thiolase, mitochondrial-like [Bombyx mori] | | | | | | | | | | | | | | | | | |
| MSTRG.10611 | brk1-a | endonuclease and reverse transcriptase-like protein [Bombyx mori] | | | | | | | | | | | | | | | | | |
| MSTRG.10612 | brk1-a | endonuclease and reverse transcriptase-like protein [Bombyx mori] | | | | | | | | | | | | | | | | | |
| MSTRG.10625 | -- | PREDICTED: alpha-2-macroglobulin-like [Bombyx mori] | | | | | | | | | | | | | | | | | |
| MSTRG.10642 | -- | PREDICTED: putative nuclease HARBI1 [Bombyx mori] | | | | | | | | | | | | | | | | | |
| MSTRG.10643 | Rpap3 | TPR-repeat protein [Bombyx mori] | | | | | | | | | | | | | | | | | |
| MSTRG.10644 | Rpap3 | TPR-repeat protein [Bombyx mori] | | | | | | | | | | | | | | | | | |
| MSTRG.10647 | PCP36 | cuticular protein RR-1 motif 11 precursor [Bombyx mori] | | | | | | | | | | | | | | | | | |
| MSTRG.10648 | PCP36 | cuticular protein RR-1 motif 11 precursor [Bombyx mori] | | | | | | | | | | | | | | | | | |
| MSTRG.1072 | -- | PREDICTED: RNA-directed DNA polymerase from mobile element jockey-like [Papilio polytes] | | | | | | | | | | | | | | | | | |
| MSTRG.1074 | Madm | PREDICTED: nuclear receptor-binding protein homolog [Bombyx mori] | | | | | | | | | | | | | | | | | |
| MSTRG.10782 | Cks30A | PREDICTED: cyclin-dependent kinases regulatory subunit [Bombyx mori] | | | | | | | | | | | | | | | | | |
| MSTRG.10792 | Faf1 | PREDICTED: FAS-associated factor 1 [Bombyx mori] | | | | | | | | | | | | | | | | | |
| MSTRG.10915 | tric-1B.1 | PREDICTED: trimeric intracellular cation channel type B [Papilio polytes] | | | | | | | | | | | | | | | | | |
| MSTRG.10933 | PABPN1 | PREDICTED: polyadenylate binding protein 2 isoform X1 [Bombyx mori] | | | | | | | | | | | | | | | | | |
| MSTRG.1095 | Traf4 | PREDICTED: TNF receptor-associated factor 4 isoform X1 [Bombyx mori] | | | | | | | | | | | | | | | | | |
| MSTRG.10979 | -- | PREDICTED: uncharacterized protein LOC105842596 [Bombyx mori] | | | | | | | | | | | | | | | | | |
| MSTRG.11035 | PGBD4 | PiggyBac transposable element-derived protein 4 [Papilio machaon] | | | | | | | | | | | | | | | | | |
| MSTRG.11052 | nhl-1 | PREDICTED: RING finger protein nhl-1-like [Bombyx mori] | | | | | | | | | | | | | | | | | |
| MSTRG.11071 | -- | CG8818, partial [Drosophila busckii] | | | | | | | | | | | | | | | | | |
| MSTRG.11110 | glpK2 | PREDICTED: putative glycerol kinase 5 [Bombyx mori] | | | | | | | | | | | | | | | | | |
| MSTRG.11111 | -- | PREDICTED: putative glycerol kinase 5 [Bombyx mori] | | | | | | | | | | | | | | | | | |
| MSTRG.11150 | -- | tRNA 5-methylaminomethyl-2-thiouridine biosynthesis bifunctional protein MnmC [Operophtera brumata] | | | | | | | | | | | | | | | | | |
| MSTRG.11222 | GTF3C1 | General transcription factor 3C polypeptide 1 [Papilio xuthus] | | | | | | | | | | | | | | | | | |
| MSTRG.11254 | col4a3bp | PREDICTED: collagen type IV alpha-3-binding protein isoform X2 [Bombyx mori] | | | | | | | | | | | | | | | | | |
| MSTRG.11283 | -- | PREDICTED: actin cytoskeleton-regulatory complex protein PAN1-like [Papilio polytes] | | | | | | | | | | | | | | | | | |
| MSTRG.11284 | -- | PREDICTED: actin cytoskeleton-regulatory complex protein PAN1-like [Papilio polytes] | | | | | | | | | | | | | | | | | |
| MSTRG.11293 | -- | - | | | | | | | | | | | | | | | | | |
| MSTRG.11341 | EbpIII | PREDICTED: chemosensory protein 9 isoform X1 [Bombyx mori] | | | | | | | | | | | | | | | | | |
| MSTRG.11399 | -- | Retrovirus-related Pol polyprotein from transposon 17.6 [Operophtera brumata] | | | | | | | | | | | | | | | | |
| MSTRG.1140 | -- | PREDICTED: ankyrin-3-like [Amyelois transitella] | | | | | | | | | | | | | | | | |
| MSTRG.11408 | -- | PREDICTED: leucine-rich repeats and immunoglobulin-like domains protein 2 [Bombyx mori] | | | | | | | | | | | | | | | | |
| MSTRG.11417 | -- | PREDICTED: receptor-like protein kinase BRI1-like 3 [Bombyx mori] | | | | | | | | | | | | | | | | |
| MSTRG.11419 | RTase | Retrotransposable element, partial [Anoplophora glabripennis] | | | | | | | | | | | | | | | | |
| MSTRG.11430 | osbpl9 | PREDICTED: oxysterol-binding protein-related protein 9 [Bombyx mori] | | | | | | | | | | | | | | | | |
| MSTRG.11557 | cactin | PREDICTED: LOW QUALITY PROTEIN: cactin [Bombyx mori] | | | | | | | | | | | | | | | | |
| MSTRG.11561 | -- | PREDICTED: tudor domain-containing protein 5 [Amyelois transitella] | | | | | | | | | | | | | | | | |
| MSTRG.11567 | -- | PREDICTED: uncharacterized protein LOC106137743 [Amyelois transitella] | | | | | | | | | | | | | | | | |
| MSTRG.11587 | MED16 | PREDICTED: mediator of RNA polymerase II transcription subunit 16 [Bombyx mori] | | | | | | | | | | | | | | | | |
| MSTRG.11599 | RTase | endonuclease and reverse transcriptase-like protein, partial [Pararge aegeria] | | | | | | | | | | | | | | | | |
| MSTRG.11656 | sel | PREDICTED: protein canopy homolog 1 [Bombyx mori] | | | | | | | | | | | | | | | | |
| MSTRG.11663 | RAB31 | PREDICTED: ras-related protein Rab-31 [Bombyx mori] | | | | | | | | | | | | | | | | |
| MSTRG.11664 | RAB31 | PREDICTED: ras-related protein Rab-31 [Bombyx mori] | | | | | | | | | | | | | | | | |
| MSTRG.11684 | UBXN4 | PREDICTED: UBX domain-containing protein 4 [Bombyx mori] | | | | | | | | | | | | | | | | |
| MSTRG.11792 | -- | - | | | | | | | | | | | | | | | | |
| MSTRG.11796 | SV2C | PREDICTED: synaptic vesicle glycoprotein 2C-like [Bombyx mori] | | | | | | | | | | | | | | | | |
| MSTRG.11868 | -- | PREDICTED: androgen-dependent TFPI-regulating protein-like [Bombyx mori] | | | | | | | | | | | | | | | | |
| MSTRG.11908 | tsu | RNA binding motif protein Y14 [Bombyx mori] | | | | | | | | | | | | | | | | |
| MSTRG.11960 | -- | cytochrome P450, partial [Bombyx mori] | | | | | | | | | | | | | | | | |
| MSTRG.12055 | -- | MLN64 N-terminal domain-like [Papilio machaon] | | | | | | | | | | | | | | | | |
| MSTRG.12056 | -- | endonuclease-reverse transcriptase [Bombyx mori] | | | | | | | | | | | | | | | | |
| MSTRG.1208 | -- | PREDICTED: C-type lectin 11 isoform X1 [Bombyx mori] | | | | | | | | | | | | | | | | |
| MSTRG.12150 | -- | transposase [Bombyx mori] | | | | | | | | | | | | | | | | |
| MSTRG.12197 | chb | PREDICTED: CLIP-associating protein [Amyelois transitella] | | | | | | | | | | | | | | | | |
| MSTRG.12198 | chb | PREDICTED: CLIP-associating protein [Amyelois transitella] | | | | | | | | | | | | | | | | |
| MSTRG.12228 | -- | transposase [Danaus plexippus] | | | | | | | | | | | | | | | | |
| MSTRG.12263 | jockey\pol | endonuclease and reverse transcriptase-like protein [Bombyx mori] | | | | | | | | | | | | | | | | |
| MSTRG.12306 | Tret1 | PREDICTED: facilitated trehalose transporter Tret1 [Bombyx mori] | | | | | | | | | | | | | | | | |
| MSTRG.12310 | PGBD4 | PREDICTED: piggyBac transposable element-derived protein 4-like isoform X4 [Bombyx mori] | | | | | | | | | | | | | | | | |
| MSTRG.12361 | SELENOI | PREDICTED: ethanolaminephosphotransferase 1-like [Bombyx mori] | | | | | | | | | | | | | | | | |
| MSTRG.12376 | -- | PREDICTED: protein Cep89 homolog isoform X1 [Bombyx mori] | | | | | | | | | | | | | | | | |
| MSTRG.12427 | -- | - | | | | | | | | | | | | | | | | |
| MSTRG.12505 | -- | Pogo transposable element with KRAB domain [Papilio machaon] | | | | | | | | | | | | | | | | |
| MSTRG.1251 | -- | PREDICTED: protein piccolo-like [Bombyx mori] | | | | | | | | | | | | | | | | |
| MSTRG.1252 | -- | PREDICTED: protein piccolo-like [Bombyx mori] | | | | | | | | | | | | | | | | |
| MSTRG.12520 | -- | Diguanylate cyclase with PAS sensor [Operophtera brumata] | | | | | | | | | | | | | | | | |
| MSTRG.12531 | Ime4 | PREDICTED: N6-adenosine-methyltransferase 70 kDa subunit isoform X1 [Bombyx mori] | | | | | | | | | | | | | | | | |
| MSTRG.12532 | Ime4 | PREDICTED: N6-adenosine-methyltransferase 70 kDa subunit isoform X1 [Bombyx mori] | | | | | | | | | | | | | | | | |
| MSTRG.1259 | -- | RNA-directed DNA polymerase from mobile element jockey [Papilio machaon] | | | | | | | | | | | | | | | |
| MSTRG.12603 | Tmem165 | PREDICTED: GDT1-like protein 5 isoform X2 [Amyelois transitella] | | | | | | | | | | | | | | | |
| MSTRG.1262 | -- | PREDICTED: zinc finger protein 711-like [Halyomorpha halys] | | | | | | | | | | | | | | | |
| MSTRG.12653 | cps-6 | PREDICTED: endonuclease G, mitochondrial, partial [Bombyx mori] | | | | | | | | | | | | | | | |
| MSTRG.12728 | scra | PREDICTED: actin-binding protein anillin-like isoform X1 [Papilio machaon] | | | | | | | | | | | | | | | |
| MSTRG.12763 | NFYC | nuclear Y/CCAAT-box binding factor C subunit NF/YC [Bombyx mori] | | | | | | | | | | | | | | | |
| MSTRG.12764 | -- | PREDICTED: uncharacterized protein LOC101736344 [Bombyx mori] | | | | | | | | | | | | | | | |
| MSTRG.12765 | -- | PREDICTED: uncharacterized protein LOC101745265 [Bombyx mori] | | | | | | | | | | | | | | | |
| MSTRG.12803 | GIP | Retrovirus-related Pol polyprotein from transposon TNT 1-94 [Papilio machaon] | | | | | | | | | | | | | | | |
| MSTRG.1281 | Ca-P60A | sarco/endoplasmic reticulum calcium ATPase [Bombyx mori] | | | | | | | | | | | | | | | |
| MSTRG.1282 | Ca-P60A | sarco/endoplasmic reticulum calcium ATPase [Bombyx mori] | | | | | | | | | | | | | | | |
| MSTRG.12850 | -- | PREDICTED: prolow-density lipoprotein receptor-related protein 1-like [Bombyx mori] | | | | | | | | | | | | | | | |
| MSTRG.12876 | -- | E3 ubiquitin-protein ligase MARCH2 [Papilio machaon] | | | | | | | | | | | | | | | |
| MSTRG.1294 | PGBD4 | PiggyBac transposable element-derived protein 4 [Papilio machaon] | | | | | | | | | | | | | | | |
| MSTRG.12945 | -- | Gag-pol polyprotein [Operophtera brumata] | | | | | | | | | | | | | | | |
| MSTRG.12947 | -- | PREDICTED: uncharacterized protein LOC106713229 [Papilio machaon] | | | | | | | | | | | | | | | |
| MSTRG.12989 | -- | lish domain-containing [Lasius niger] | | | | | | | | | | | | | | | |
| MSTRG.13069 | Vps28 | PREDICTED: vacuolar protein sorting-associated protein 28 homolog [Bombyx mori] | | | | | | | | | | | | | | | |
| MSTRG.13148 | -- | olfactory receptor [Bombyx mori] | | | | | | | | | | | | | | | |
| MSTRG.13150 | -- | PREDICTED: snRNA-activating protein complex subunit 3 [Papilio machaon] | | | | | | | | | | | | | | | |
| MSTRG.13166 | -- | cuticular protein glycine-rich 20 precursor [Bombyx mori] | | | | | | | | | | | | | | | |
| MSTRG.13206 | SDH | PREDICTED: sorbitol dehydrogenase [Bombyx mori] | | | | | | | | | | | | | | | |
| MSTRG.13227 | Egfem1 | PREDICTED: collagen and calcium-binding EGF domain-containing protein 1-like [Bombyx mori] | | | | | | | | | | | | | | | |
| MSTRG.13247 | -- | PREDICTED: arrestin domain-containing protein 2-like isoform X1 [Bombyx mori] | | | | | | | | | | | | | | | |
| MSTRG.13248 | IPO11 | PREDICTED: importin-11 [Bombyx mori] | | | | | | | | | | | | | | | |
| MSTRG.13250 | -- | olfactory receptor [Bombyx mori] | | | | | | | | | | | | | | | |
| MSTRG.13277 | -- | PREDICTED: uncharacterized protein LOC101743025 [Bombyx mori] | | | | | | | | | | | | | | | |
| MSTRG.13278 | -- | PREDICTED: uncharacterized protein LOC106140183 [Amyelois transitella] | | | | | | | | | | | | | | | |
| MSTRG.13282 | -- | PREDICTED: uncharacterized protein LOC106143303 [Amyelois transitella] | | | | | | | | | | | | | | | |
| MSTRG.13300 | -- | PREDICTED: keratin-associated protein 19-2-like [Bombyx mori] | | | | | | | | | | | | | | | |
| MSTRG.13310 | -- | PREDICTED: probable maltase-glucoamylase-like protein, partial [Bombyx mori] | | | | | | | | | | | | | | | |
| MSTRG.13355 | cbd-1 | PREDICTED: chondroitin proteoglycan-2-like, partial [Bombyx mori] | | | | | | | | | | | | | | | |
| MSTRG.13378 | Ptpn9 | PREDICTED: tyrosine-protein phosphatase non-receptor type 9 isoform X2 [Bombyx mori] | | | | | | | | | | | | | | | |
| MSTRG.13397 | -- | chemosensory protein 5 [Athetis dissimilis] | | | | | | | | | | | | | | | |
| MSTRG.13415 | Hmcn2 | PREDICTED: lachesin-like [Bombyx mori] | | | | | | | | | | | | | | | |
| MSTRG.13426 | -- | transposase [Bombyx mori] | | | | | | | | | | | | | | | |
| MSTRG.13448 | chaf1a | PREDICTED: chromatin assembly factor 1 subunit A [Bombyx mori] | | | | | | | | | | | | | | | |
| MSTRG.13466 | RTase | PREDICTED: RNA-directed DNA polymerase from mobile element jockey-like [Amyelois transitella] | | | | | | | | | | | | | | | |
| MSTRG.13596 | PTAR1 | PREDICTED: protein prenyltransferase alpha subunit repeat-containing protein 1 [Bombyx mori] | | | | | | | | | | | | | | | |
| MSTRG.13618 | Dhx34 | PREDICTED: probable ATP-dependent RNA helicase DHX34 [Bombyx mori] | | | | | | | | | | | | | | |
| MSTRG.13627 | -- | PREDICTED: uncharacterized protein LOC105842123 [Bombyx mori] | | | | | | | | | | | | | | |
| MSTRG.13700 | -- | PREDICTED: uncharacterized protein LOC105842136 [Bombyx mori] | | | | | | | | | | | | | | |
| MSTRG.13773 | daao-1 | PREDICTED: D-amino-acid oxidase [Bombyx mori] | | | | | | | | | | | | | | |
| MSTRG.13842 | CLPX | PREDICTED: ATP-dependent Clp protease ATP-binding subunit clpX-like, mitochondrial [Bombyx mori] | | | | | | | | | | | | | | |
| MSTRG.13847 | CYP6K1 | PREDICTED: cytochrome P450 6k1-like [Bombyx mori] | | | | | | | | | | | | | | |
| MSTRG.13856 | TY3B-I | Transposon Tf2-9 polyprotein-like Protein [Tribolium castaneum] | | | | | | | | | | | | | | |
| MSTRG.1387 | CYB561A3 | PREDICTED: cytochrome b reductase 1 isoform X2 [Bombyx mori] | | | | | | | | | | | | | | |
| MSTRG.1388 | CYB561A3 | PREDICTED: cytochrome b reductase 1 isoform X2 [Bombyx mori] | | | | | | | | | | | | | | |
| MSTRG.13898 | RTase | endonuclease and reverse transcriptase-like protein, partial [Pararge aegeria] | | | | | | | | | | | | | | |
| MSTRG.13932 | -- | REPAT33 [Spodoptera exigua] | | | | | | | | | | | | | | |
| MSTRG.13964 | app | PREDICTED: palmitoyltransferase ZDHHC18 [Amyelois transitella] | | | | | | | | | | | | | | |
| MSTRG.14038 | pol | Retrovirus-related Pol polyprotein from transposon 412-like Protein [Tribolium castaneum] | | | | | | | | | | | | | | |
| MSTRG.14054 | -- | olfactory receptor [Bombyx mori] | | | | | | | | | | | | | | |
| MSTRG.14067 | -- | PREDICTED: 5'-3' exoribonuclease 1 isoform X2 [Bombyx mori] | | | | | | | | | | | | | | |
| MSTRG.14068 | -- | PREDICTED: 5'-3' exoribonuclease 1 isoform X2 [Bombyx mori] | | | | | | | | | | | | | | |
| MSTRG.14077 | -- | PREDICTED: spermatogenesis-associated protein 6 isoform X2 [Bombyx mori] | | | | | | | | | | | | | | |
| MSTRG.14138 | -- | - | | | | | | | | | | | | | | |
| MSTRG.14230 | MEAF6 | PREDICTED: chromatin modification-related protein MEAF6 [Amyelois transitella] | | | | | | | | | | | | | | |
| MSTRG.14280 | PNLIPRP2 | PREDICTED: pancreatic lipase-related protein 2-like [Bombyx mori] | | | | | | | | | | | | | | |
| MSTRG.14356 | -- | Protein odr-4-like [Papilio machaon] | | | | | | | | | | | | | | |
| MSTRG.14370 | lin-28 | PREDICTED: protein lin-28 homolog [Bombyx mori] | | | | | | | | | | | | | | |
| MSTRG.14409 | PCP20 | cuticular protein RR-1 motif 37 precursor [Bombyx mori] | | | | | | | | | | | | | | |
| MSTRG.14414 | Edg78E | cuticular protein RR-1 motif 34 precursor [Bombyx mori] | | | | | | | | | | | | | | |
| MSTRG.14416 | LCP17 | PREDICTED: cuticular protein RR-1 motif 33 isoform X1 [Bombyx mori] | | | | | | | | | | | | | | |
| MSTRG.1445 | -- | reverse transcriptase, partial [Bombyx mori] | | | | | | | | | | | | | | |
| MSTRG.14458 | BVES | PREDICTED: popeye domain-containing protein 3-like [Papilio machaon] | | | | | | | | | | | | | | |
| MSTRG.14474 | -- | PREDICTED: zinc finger protein 729-like isoform X2 [Bombyx mori] | | | | | | | | | | | | | | |
| MSTRG.14478 | -- | PREDICTED: myoneurin-like isoform X2 [Bombyx mori] | | | | | | | | | | | | | | |
| MSTRG.14479 | -- | PREDICTED: myoneurin-like isoform X1 [Bombyx mori] | | | | | | | | | | | | | | |
| MSTRG.1451 | SPR | myosuppressin receptor [Bombyx mori] | | | | | | | | | | | | | | |
| MSTRG.14530 | Zasp52 | PREDICTED: PDZ and LIM domain protein Zasp [Bombyx mori] | | | | | | | | | | | | | | |
| MSTRG.14531 | Zasp52 | PREDICTED: PDZ and LIM domain protein Zasp [Bombyx mori] | | | | | | | | | | | | | | |
| MSTRG.14532 | NOLC1 | PREDICTED: nucleolar and coiled-body phosphoprotein 1 [Bombyx mori] | | | | | | | | | | | | | | |
| MSTRG.14533 | NOLC1 | PREDICTED: nucleolar and coiled-body phosphoprotein 1 [Bombyx mori] | | | | | | | | | | | | | | |
| MSTRG.1455 | -- | polyprotein [Danaus plexippus] | | | | | | | | | | | | | | |
| MSTRG.14556 | -- | PREDICTED: aspartic and glutamic acid-rich protein-like isoform X2 [Bombyx mori] | | | | | | | | | | | | | | |
| MSTRG.14558 | -- | PREDICTED: aspartic and glutamic acid-rich protein-like isoform X2 [Bombyx mori] | | | | | | | | | | | | | | |
| MSTRG.14594 | -- | PREDICTED: uncharacterized protein LOC105390591 isoform X2 [Plutella xylostella] | | | | | | | | | | | | | | |
| MSTRG.14615 | Parp | PREDICTED: poly [ADP-ribose] polymerase [Bombyx mori] | | | | | | | | | | | | | | |
| MSTRG.1468 | caz | PREDICTED: RNA-binding protein cabeza-like isoform X1 [Bombyx mori] | | | | | | | | | | | | | | |
| MSTRG.1471 | Miro | PREDICTED: LOW QUALITY PROTEIN: mitochondrial Rho GTPase-like [Bombyx mori] | | | | | | | | | | | | | | |
| MSTRG.14711 | ELOVL4 | PREDICTED: elongation of very long chain fatty acids protein 4-like isoform X1 [Bombyx mori] | | | | | | | | | | | | | | |
| MSTRG.14736 | CG1542 | rRNA processing protein Ebp2 [Bombyx mori] | | | | | | | | | | | | | | |
| MSTRG.14777 | omd | PREDICTED: integrator complex subunit 5 [Bombyx mori] | | | | | | | | | | | | | | |
| MSTRG.14797 | mknk1 | PREDICTED: MAP kinase-interacting serine/threonine-protein kinase 1 [Bombyx mori] | | | | | | | | | | | | | | |
| MSTRG.14798 | mknk1 | PREDICTED: MAP kinase-interacting serine/threonine-protein kinase 1 [Bombyx mori] | | | | | | | | | | | | | | |
| MSTRG.1486 | -- | retroelement polyprotein [Glyptapanteles flavicoxis] | | | | | | | | | | | | | | |
| MSTRG.14870 | PUS7 | PREDICTED: pseudouridylate synthase 7 homolog [Amyelois transitella] | | | | | | | | | | | | | | |
| MSTRG.14877 | -- | reverse transcriptase, partial [Bombyx mori] | | | | | | | | | | | | | | |
| MSTRG.14884 | -- | Ferric-chelate reductase [Operophtera brumata] | | | | | | | | | | | | | | |
| MSTRG.14901 | -- | MOCS1A [Bombyx mori] | | | | | | | | | | | | | | |
| MSTRG.14924 | -- | PREDICTED: uncharacterized protein LOC105272169 isoform X1 [Fopius arisanus] | | | | | | | | | | | | | | |
| MSTRG.14931 | -- | UPF0439 protein C9orf30-like protein [Operophtera brumata] | | | | | | | | | | | | | | |
| MSTRG.14977 | RTase | PREDICTED: RNA-directed DNA polymerase from mobile element jockey-like [Amyelois transitella] | | | | | | | | | | | | | | |
| MSTRG.15012 | PBDC1 | UPF0368 protein Cxorf26-like protein [Pararge aegeria] | | | | | | | | | | | | | | |
| MSTRG.15013 | PBDC1 | UPF0368 protein Cxorf26-like protein [Pararge aegeria] | | | | | | | | | | | | | | |
| MSTRG.15041 | S2P | PREDICTED: membrane-bound transcription factor site-2 protease [Bombyx mori] | | | | | | | | | | | | | | |
| MSTRG.15087 | -- | polyprotein, partial [Operophtera brumata] | | | | | | | | | | | | | | |
| MSTRG.1509 | -- | gag pol polyprotein [Lasius niger] | | | | | | | | | | | | | | |
| MSTRG.1513 | X-element\ORF2 | PREDICTED: RNA-directed DNA polymerase from mobile element jockey-like isoform X3 [Papilio machaon] | | | | | | | | | | | | | | |
| MSTRG.15180 | anon-3B1.2 | PREDICTED: circadian clock-controlled protein-like [Amyelois transitella] | | | | | | | | | | | | | | |
| MSTRG.15181 | -- | PREDICTED: circadian clock-controlled protein-like [Bombyx mori] | | | | | | | | | | | | | | |
| MSTRG.15182 | -- | Glucosidase 2 subunit beta [Exaiptasia pallida] | | | | | | | | | | | | | | |
| MSTRG.15239 | -- | - | | | | | | | | | | | | | | |
| MSTRG.1525 | HDLBP | PREDICTED: vigilin [Amyelois transitella] | | | | | | | | | | | | | | |
| MSTRG.1526 | HDLBP | PREDICTED: vigilin [Amyelois transitella] | | | | | | | | | | | | | | |
| MSTRG.15273 | Orct | PREDICTED: organic cation transporter protein-like isoform X2 [Bombyx mori] | | | | | | | | | | | | | | |
| MSTRG.15289 | mgr | prefoldin subunit 3 [Bombyx mori] | | | | | | | | | | | | | | |
| MSTRG.15293 | Bdh1 | PREDICTED: D-beta-hydroxybutyrate dehydrogenase, mitochondrial-like [Bombyx mori] | | | | | | | | | | | | | | |
| MSTRG.15334 | SETMAR | PREDICTED: histone-lysine N-methyltransferase SETMAR-like [Camponotus floridanus] | | | | | | | | | | | | | | |
| MSTRG.15396 | HPS1 | PREDICTED: Hermansky-Pudlak syndrome 1 protein homolog [Bombyx mori] | | | | | | | | | | | | | | |
| MSTRG.15420 | -- | PREDICTED: prominin-like protein [Bombyx mori] | | | | | | | | | | | | | | |
| MSTRG.15514 | -- | PREDICTED: uncharacterized protein LOC105842795 [Bombyx mori] | | | | | | | | | | | | | | |
| MSTRG.15539 | Iscu | PREDICTED: iron-sulfur cluster assembly enzyme ISCU, mitochondrial [Bombyx mori] | | | | | | | | | | | | | | |
| MSTRG.1565 | FAM126A | PREDICTED: hyccin [Bombyx mori] | | | | | | | | | | | | |
| MSTRG.15658 | -- | olfactory receptor [Bombyx mori] | | | | | | | | | | | | |
| MSTRG.1570 | ABAT | PREDICTED: 4-aminobutyrate aminotransferase, mitochondrial [Bombyx mori] | | | | | | | | | | | | |
| MSTRG.15733 | AAEL006169 | cathepsin D precursor [Bombyx mori] | | | | | | | | | | | | |
| MSTRG.15755 | -- | endonuclease-reverse transcriptase [Bombyx mori] | | | | | | | | | | | | |
| MSTRG.15762 | CdGAPr | PREDICTED: GTPase-activating protein CdGAPr [Bombyx mori] | | | | | | | | | | | | |
| MSTRG.15764 | -- | PREDICTED: GTPase-activating protein CdGAPr [Amyelois transitella] | | | | | | | | | | | | |
| MSTRG.15799 | -- | Prolow-density lipoprotein receptor-related protein 1 [Papilio machaon] | | | | | | | | | | | | | |
| MSTRG.1588 | sdk | PREDICTED: LOW QUALITY PROTEIN: protein sidekick [Bombyx mori] | | | | | | | | | | | | | |
| MSTRG.1589 | sdk | PREDICTED: LOW QUALITY PROTEIN: protein sidekick [Bombyx mori] | | | | | | | | | | | | | |
| MSTRG.16014 | ANKRD50 | PREDICTED: ankyrin repeat domain-containing protein 50 [Amyelois transitella] | | | | | | | | | | | | | |
| MSTRG.16016 | ANKRD50 | PREDICTED: ankyrin repeat domain-containing protein 50 [Amyelois transitella] | | | | | | | | | | | | | |
| MSTRG.16035 | wat | PREDICTED: fatty acyl-CoA reductase 1-like [Bombyx mori] | | | | | | | | | | | | | |
| MSTRG.16038 | -- | uncharacterized LOC106124743 precursor [Papilio xuthus] | | | | | | | | | | | | | |
| MSTRG.1614 | CDK11B | cell division cycle 2 like-1 [Bombyx mori] | | | | | | | | | | | | | |
| MSTRG.1615 | CDK11B | PREDICTED: cell division cycle 2 like-1 isoform X1 [Bombyx mori] | | | | | | | | | | | | | |
| MSTRG.16151 | -- | - | | | | | | | | | | | | | |
| MSTRG.16159 | -- | olfactory receptor [Bombyx mori] | | | | | | | | | | | | | |
| MSTRG.16172 | -- | PREDICTED: gastrula zinc finger protein XlCGF46.1-like, partial [Bombyx mori] | | | | | | | | | | | | | |
| MSTRG.16173 | -- | PREDICTED: zinc finger protein 431-like [Bombyx mori] | | | | | | | | | | | | | |
| MSTRG.16209 | RhoGAPp190 | PREDICTED: rho GTPase-activating protein 190 isoform X2 [Bombyx mori] | | | | | | | | | | | | | |
| MSTRG.16214 | resilin | PREDICTED: pro-resilin [Bombyx mori] | | | | | | | | | | | | | |
| MSTRG.16224 | NANOS2 | zinc finger protein NANOS-P [Bombyx mori] | | | | | | | | | | | | | |
| MSTRG.16241 | -- | Zinc finger protein 26, partial [Pararge aegeria] | | | | | | | | | | | | | |
| MSTRG.16242 | FBXO28 | PREDICTED: F-box only protein 28 [Bombyx mori] | | | | | | | | | | | | | |
| MSTRG.16289 | -- | - | | | | | | | | | | | | | |
| MSTRG.16320 | -- | PREDICTED: ommochrome-binding protein-like [Papilio xuthus] | | | | | | | | | | | | | |
| MSTRG.16351 | -- | PREDICTED: THAP domain-containing protein 1-like isoform X1 [Bombyx mori] | | | | | | | | | | | | | |
| MSTRG.16372 | -- | PREDICTED: THAP domain-containing protein 1-like isoform X1 [Bombyx mori] | | | | | | | | | | | | | |
| MSTRG.16377 | -- | Irregular chiasm C-roughest protein [Papilio xuthus] | | | | | | | | | | | | | |
| MSTRG.16404 | rst | PREDICTED: irregular chiasm C-roughest protein-like isoform X1 [Bombyx mori] | | | | | | | | | | | | | |
| MSTRG.1644 | -- | PREDICTED: methylosome protein 50-like [Bombyx mori] | | | | | | | | | | | | | |
| MSTRG.16467 | -- | PREDICTED: stAR-related lipid transfer protein 7, mitochondrial-like isoform X1 [Papilio polytes] | | | | | | | | | | | | | |
| MSTRG.16499 | -- | Retrovirus-related Pol polyprotein from transposon TNT 1-94, partial [Ceratitis capitata] | | | | | | | | | | | | | |
| MSTRG.16537 | XPO7 | PREDICTED: exportin-7-B [Amyelois transitella] | | | | | | | | | | | | | |
| MSTRG.16539 | XPO7 | PREDICTED: exportin-7-B [Amyelois transitella] | | | | | | | | | | | | | |
| MSTRG.16557 | -- | PREDICTED: zinc finger protein 567-like [Bombyx mori] | | | | | | | | | | | | | |
| MSTRG.1662 | -- | Speract receptor [Papilio xuthus] | | | | | | | | | | | | | |
| MSTRG.16626 | Tf2-12 | Transposon Tf2-9 polyprotein-like Protein [Tribolium castaneum] | | | | | | | | | | | | | |
| MSTRG.16628 | -- | PREDICTED: biogenesis of lysosome-related organelles complex 1 subunit 3 isoform X2 [Aethina tumida] | | | | | | | | | | | | | |
| MSTRG.16768 | Rbm25 | PREDICTED: RNA-binding protein 25 [Bombyx mori] | | | | | | | | | | | | | |
| MSTRG.1677 | DNApol-alpha50 | PREDICTED: DNA primase small subunit [Bombyx mori] | | | | | | | | | | | | | |
| MSTRG.16771 | ROBO3 | PREDICTED: hemicentin-1 isoform X3 [Papilio xuthus] | | | | | | | | | | | | | |
| MSTRG.16800 | betA | PREDICTED: glucose dehydrogenase [FAD, quinone]-like [Bombyx mori] | | | | | | | | | | | | | |
| MSTRG.16808 | MAFF | PREDICTED: transcription factor MafK [Bombyx mori] | | | | | | | | | | | | | |
| MSTRG.1681 | CG9132 | PREDICTED: NECAP-like protein CG9132 [Bombyx mori] | | | | | | | | | | | | | |
| MSTRG.16825 | -- | PREDICTED: uncharacterized protein LOC101742436 isoform X1 [Bombyx mori] | | | | | | | | | | | | | |
| MSTRG.16855 | MAP4K5 | PREDICTED: mitogen-activated protein kinase kinase kinase kinase 5 isoform X1 [Bombyx mori] | | | | | | | | | | | | | |
| MSTRG.16857 | MAP4K3 | PREDICTED: mitogen-activated protein kinase kinase kinase kinase 5 isoform X2 [Bombyx mori] | | | | | | | | | | | | | |
| MSTRG.16882 | -- | PREDICTED: zinc finger protein 91-like [Amyelois transitella] | | | | | | | | | | | | | |
| MSTRG.1691 | MICU3 | PREDICTED: calcium uptake protein 3, mitochondrial isoform X4 [Amyelois transitella] | | | | | | | | | | | | | |
| MSTRG.1692 | Micu3 | PREDICTED: calcium uptake protein 3, mitochondrial isoform X4 [Amyelois transitella] | | | | | | | | | | | | | |
| MSTRG.1693 | Nop17l | PREDICTED: LOW QUALITY PROTEIN: protein kintoun [Bombyx mori] | | | | | | | | | | | | | |
| MSTRG.17014 | Slc25a3 | phosphate transport protein [Bombyx mori] | | | | | | | | | | | | | |
| MSTRG.17022 | -- | polyprotein, partial [Bombyx mori] | | | | | | | | | | | | | |
| MSTRG.17028 | -- | gag-like protein [Bombyx mori] | | | | | | | | | | | | | |
| MSTRG.17035 | -- | PREDICTED: THAP domain-containing protein 4-like [Bombyx mori] | | | | | | | | | | | | | |
| MSTRG.17111 | -- | Zinc finger protein 460 [Pararge aegeria] | | | | | | | | | | | | | |
| MSTRG.17117 | -- | PREDICTED: sugar transporter ERD6-like 2 [Papilio xuthus] | | | | | | | | | | | | | |
| MSTRG.17120 | -- | - | | | | | | | | | | | | | |
| MSTRG.17145 | -- | ATPase (PilT family) [Operophtera brumata] | | | | | | | | | | | | | |
| MSTRG.17162 | -- | PREDICTED: uncharacterized protein LOC105842541 [Bombyx mori] | | | | | | | | | | | | | |
| MSTRG.17177 | FDX1 | PREDICTED: adrenodoxin [Bombyx mori] | | | | | | | | | | | | | |
| MSTRG.17225 | LDB2 | PREDICTED: LIM domain-binding protein 2 [Bombyx mori] | | | | | | | | | | | | | |
| MSTRG.17258 | -- | Protein SERAC1 [Papilio machaon] | | | | | | | | | | | | | |
| MSTRG.17260 | -- | PREDICTED: protein SERAC1 [Papilio polytes] | | | | | | | | | | | | | |
| MSTRG.17267 | pol | Retrovirus-related Pol polyprotein from transposon 17.6 [Operophtera brumata] | | | | | | | | | | | | | |
| MSTRG.17339 | -- | reverse transcriptase [Bombyx mori] | | | | | | | | | | | | | |
| MSTRG.17340 | -- | kda protein in nof-fb transposable element [Lasius niger] | | | | | | | | | | | | | |
| MSTRG.17344 | -- | olfactory receptor [Bombyx mori] | | | | | | | | | | | | | |
| MSTRG.17352 | TCERG1 | PREDICTED: transcription elongation regulator 1-like, partial [Bombyx mori] | | | | | | | | | | | | | |
| MSTRG.17353 | TCERG1 | PREDICTED: transcription elongation regulator 1-like, partial [Bombyx mori] | | | | | | | | | | | | | |
| MSTRG.1742 | SWEET1B | PREDICTED: LOW QUALITY PROTEIN: sugar transporter SWEET1 [Bombyx mori] | | | | | | | | | | | | | |
| MSTRG.17448 | Exosc5 | PREDICTED: exosome complex component RRP46 [Bombyx mori] | | | | | | | | | | | | | |
| MSTRG.1745 | -- | Sortilin-related receptor [Papilio machaon] | | | | | | | | | | | | | |
| MSTRG.17464 | TTLL5 | PREDICTED: tubulin polyglutamylase ttll-4 [Bombyx mori] | | | | | | | | | | | | | |
| MSTRG.17480 | -- | PREDICTED: protein sel-1 homolog 1 isoform X3 [Bombyx mori] | | | | | | | | | | | | | |
| MSTRG.17482 | Sel1l | PREDICTED: protein sel-1 homolog 1 isoform X3 [Bombyx mori] | | | | | | | | | | | | | |
| MSTRG.17557 | Apod | chlorophyllide A binding protein precursor [Bombyx mori] | | | | | | | | | | | | | |
| MSTRG.17558 | Apod | PREDICTED: chlorophyllide A binding protein isoform X1 [Bombyx mori] | | | | | | | | | | | | |
| MSTRG.17575 | -- | PREDICTED: N-acetylneuraminate lyase-like isoform X1 [Bombyx mori] | | | | | | | | | | | | |
| MSTRG.17591 | SAYSD1 | PREDICTED: SAYSvFN domain-containing protein 1 [Bombyx mori] | | | | | | | | | | | | |
| MSTRG.17722 | Rpt2 | PREDICTED: 26S protease regulatory subunit 4 [Bombyx mori] | | | | | | | | | | | | |
| MSTRG.17770 | -- | PREDICTED: uncharacterized protein C15orf41 homolog [Bombyx mori] | | | | | | | | | | | | |
| MSTRG.17774 | -- | PREDICTED: zinc finger protein 711-like [Halyomorpha halys] | | | | | | | | | | | | |
| MSTRG.17778 | -- | PREDICTED: epididymal secretory protein E1-like isoform X1 [Bombyx mori] | | | | | | | | | | | | |
| MSTRG.17827 | Max | PREDICTED: protein max isoform X2 [Bombyx mori] | | | | | | | | | | | | |
| MSTRG.17862 | -- | PREDICTED: LOW QUALITY PROTEIN: GATA zinc finger domain-containing protein 10 [Acromyrmex echinatior] | | | | | | | | | | | | |
| MSTRG.17863 | -- | Hypothetical protein, partial [Pararge aegeria] | | | | | | | | | | | | |
| MSTRG.17886 | -- | PREDICTED: putative nuclease HARBI1 [Dendroctonus ponderosae] | | | | | | | | | | | | |
| MSTRG.1798 | -- | Transcription factor E2F4 [Papilio machaon] | | | | | | | | | | | | |
| MSTRG.18008 | alpha-Man-IIa | PREDICTED: alpha-mannosidase 2 [Bombyx mori] | | | | | | | | | | | | |
| MSTRG.18042 | -- | transposase [Bombyx mori] | | | | | | | | | | | | |
| MSTRG.18081 | prpf4B | PREDICTED: dual specificity protein kinase KNS1 [Bombyx mori] | | | | | | | | | | | | |
| MSTRG.18162 | Cpsf100 | PREDICTED: probable cleavage and polyadenylation specificity factor subunit 2, partial [Bombyx mori] | | | | | | | | | | | | |
| MSTRG.18175 | pol | reverse transcriptase-like protein [Bombyx mori] | | | | | | | | | | | | |
| MSTRG.1818 | -- | putative cuticle protein CPH43 [Bombyx mori] | | | | | | | | | | | | |
| MSTRG.18198 | SCP2D1 | PREDICTED: fatty acid-binding protein-like [Bombyx mori] | | | | | | | | | | | | |
| MSTRG.1820 | -- | putative cuticle protein CPH43 [Bombyx mori] | | | | | | | | | | | | |
| MSTRG.18230 | cfap36 | PREDICTED: cilia- and flagella-associated protein 36 [Bombyx mori] | | | | | | | | | | | | |
| MSTRG.18231 | -- | PREDICTED: phosphatidylinositol N-acetylglucosaminyltransferase subunit H isoform X2 [Bombyx mori] | | | | | | | | | | | | |
| MSTRG.18238 | Akr1e2 | PREDICTED: aldose reductase-like isoform X2 [Bombyx mori] | | | | | | | | | | | | |
| MSTRG.18240 | Mcm5 | PREDICTED: DNA replication licensing factor Mcm5-like [Plutella xylostella] | | | | | | | | | | | | |
| MSTRG.18241 | SDE3 | PREDICTED: probable RNA helicase SDE3 [Amyelois transitella] | | | | | | | | | | | | |
| MSTRG.18250 | Atpalpha | PREDICTED: sodium/potassium-transporting ATPase subunit alpha isoform X1 [Bombyx mori] | | | | | | | | | | | | |
| MSTRG.18253 | GluProRS | PREDICTED: bifunctional glutamate/proline--tRNA ligase isoform X1 [Bombyx mori] | | | | | | | | | | | | |
| MSTRG.18288 | -- | olfactory receptor [Bombyx mori] | | | | | | | | | | | | |
| MSTRG.18336 | Skeletor | PREDICTED: protein Skeletor, isoforms D/E isoform X1 [Bombyx mori] | | | | | | | | | | | | |
| MSTRG.18337 | Skeletor | PREDICTED: protein Skeletor, isoforms D/E isoform X1 [Bombyx mori] | | | | | | | | | | | | |
| MSTRG.1835 | HSD17B14 | PREDICTED: alcohol dehydrogenase 1-like [Bombyx mori] | | | | | | | | | | | | |
| MSTRG.18439 | Gprk2 | PREDICTED: G protein-coupled receptor kinase 2 isoform X1 [Bombyx mori] | | | | | | | | | | | | |
| MSTRG.1856 | GIP | PREDICTED: mucin-2-like [Amyelois transitella] | | | | | | | | | | | | |
| MSTRG.18568 | -- | cytochrome P450, partial [Bombyx mori] | | | | | | | | | | | | |
| MSTRG.18592 | -- | Zinc finger protein 41 [Pararge aegeria] | | | | | | | | | | | | |
| MSTRG.18648 | MELTF | PREDICTED: melanotransferrin [Bombyx mori] | | | | | | | | | | | | |
| MSTRG.18754 | -- | PREDICTED: B-cell linker protein-like [Halyomorpha halys] | | | | | | | | | | | | |
| MSTRG.18757 | Tret1 | PREDICTED: sugar transporter 4 isoform X1 [Bombyx mori] | | | | | | | | | | | | |
| MSTRG.18868 | maea | PREDICTED: macrophage erythroblast attacher isoform X1 [Bombyx mori] | | | | | | | | | | | | |
| MSTRG.18870 | CtBP | PREDICTED: C-terminal-binding protein isoform X1 [Bombyx mori] | | | | | | | | | | | |
| MSTRG.18872 | CFAP58 | PREDICTED: cilia- and flagella-associated protein 58-like [Bombyx mori] | | | | | | | | | | | |
| MSTRG.18895 | POL | Transposon Tf2-9 polyprotein-like Protein [Tribolium castaneum] | | | | | | | | | | | |
| MSTRG.18906 | -- | uncharacterized LOC106116239 precursor [Papilio xuthus] | | | | | | | | | | | |
| MSTRG.18926 | CECD | cytochrome P450, partial [Bombyx mori] | | | | | | | | | | | |
| MSTRG.1901 | TSPAN33 | PREDICTED: 23 kDa integral membrane protein-like [Bombyx mori] | | | | | | | | | | | |
| MSTRG.19016 | Ube2h | PREDICTED: ubiquitin-conjugating enzyme E2 H [Bombyx mori] | | | | | | | | | | | |
| MSTRG.19028 | SEC24C | PREDICTED: protein transport protein Sec24C isoform X2 [Bombyx mori] | | | | | | | | | | | |
| MSTRG.1903 | CD63 | PREDICTED: CD63 antigen-like [Bombyx mori] | | | | | | | | | | | |
| MSTRG.1904 | CD63 | PREDICTED: CD63 antigen-like [Bombyx mori] | | | | | | | | | | | |
| MSTRG.19051 | -- | olfactory receptor [Bombyx mori] | | | | | | | | | | | |
| MSTRG.1906 | -- | PREDICTED: CD63 antigen-like [Bombyx mori] | | | | | | | | | | | |
| MSTRG.19066 | -- | PREDICTED: sporozoite surface protein 2-like [Bombyx mori] | | | | | | | | | | | |
| MSTRG.19078 | -- | PREDICTED: uncharacterized protein LOC101744165 [Bombyx mori] | | | | | | | | | | | |
| MSTRG.1908 | -- | PREDICTED: leukocyte surface antigen CD53-like [Bombyx mori] | | | | | | | | | | | |
| MSTRG.19106 | MAGI3 | Membrane-associated guanylate kinase, WW and PDZ domain-containing protein 2 [Papilio xuthus] | | | | | | | | | | | |
| MSTRG.19107 | Magi1 | PREDICTED: membrane-associated guanylate kinase, WW and PDZ domain-containing protein 1 [Papilio xuthus] | | | | | | | | | | | |
| MSTRG.19137 | -- | PREDICTED: uncharacterized protein LOC106142350 [Amyelois transitella] | | | | | | | | | | | |
| MSTRG.19142 | -- | gag-pol polyprotein precursor, partial [Pararge aegeria] | | | | | | | | | | | |
| MSTRG.19159 | RhoGAPp190 | PREDICTED: rho GTPase-activating protein 190 isoform X2 [Bombyx mori] | | | | | | | | | | | |
| MSTRG.19169 | -- | PREDICTED: LOW QUALITY PROTEIN: ankyrin-2-like [Bombyx mori] | | | | | | | | | | | |
| MSTRG.19170 | Ank3 | Ankyrin-2 [Papilio xuthus] | | | | | | | | | | | |
| MSTRG.19171 | -- | PREDICTED: LOW QUALITY PROTEIN: ankyrin-2-like [Bombyx mori] | | | | | | | | | | | |
| MSTRG.19172 | -- | - | | | | | | | | | | | |
| MSTRG.19242 | W08D2.5 | PREDICTED: probable cation-transporting ATPase 13A3 [Bombyx mori] | | | | | | | | | | | |
| MSTRG.19244 | ATP13A3 | PREDICTED: probable cation-transporting ATPase 13A3 [Bombyx mori] | | | | | | | | | | | |
| MSTRG.1933 | CD63 | tetraspanin E [Bombyx mori] | | | | | | | | | | | |
| MSTRG.19334 | -- | reverse transcriptase, partial [Bombyx mori] | | | | | | | | | | | |
| MSTRG.1934 | CD63 | tetraspanin E [Bombyx mori] | | | | | | | | | | | |
| MSTRG.19357 | -- | PREDICTED: zinc finger protein 267-like [Amyelois transitella] | | | | | | | | | | | |
| MSTRG.19359 | GIP | Yokozuna [Bombyx mori] | | | | | | | | | | | |
| MSTRG.19412 | -- | olfactory receptor [Bombyx mori] | | | | | | | | | | | |
| MSTRG.19436 | ClC-a | PREDICTED: chloride channel protein 2 [Bombyx mori] | | | | | | | | | | | |
| MSTRG.19437 | ClC-a | PREDICTED: chloride channel protein 2 [Bombyx mori] | | | | | | | | | | | |
| MSTRG.19458 | Tret1-2 | PREDICTED: facilitated trehalose transporter Tret1-like [Bombyx mori] | | | | | | | | | | | |
| MSTRG.19463 | -- | cytochrome P450, partial [Bombyx mori] | | | | | | | | | | | |
| MSTRG.19528 | chrnb1 | nicotinic acetylcholine receptor subunit beta 3 precursor [Bombyx mori] | | | | | | | | | | | |
| MSTRG.19529 | chrnb1 | nicotinic acetylcholine receptor subunit beta 3 precursor [Bombyx mori] | | | | | | | | | | | |
| MSTRG.19593 | -- | PREDICTED: uncharacterized protein LOC106710902 [Papilio machaon] | | | | | | | | | | | |
| MSTRG.19651 | -- | PREDICTED: serine protease 42-like, partial [Ceratosolen solmsi marchali] | | | | | | | | | | | |
| MSTRG.1967 | Dbi | acyl-CoA binding protein [Bombyx mori] | | | | | | | | | | | |
| MSTRG.197 | Svep1 | PREDICTED: sushi, von Willebrand factor type A, EGF and pentraxin domain-containing protein 1 [Bombyx mori] | | | | | | | | | | | |
| MSTRG.19767 | -- | olfactory receptor [Bombyx mori] | | | | | | | | | | | |
| MSTRG.19799 | -- | PREDICTED: F-box only protein 33 [Bemisia tabaci] | | | | | | | | | | | |
| MSTRG.19822 | -- | olfactory receptor [Bombyx mori] | | | | | | | | | | | |
| MSTRG.19839 | Yaf2 | ring and YY1 binding protein [Bombyx mori] | | | | | | | | | | | |
| MSTRG.19847 | -- | PREDICTED: uncharacterized protein LOC101746177 [Bombyx mori] | | | | | | | | | | | |
| MSTRG.19860 | -- | juvenile hormone esterase binding protein [Bombyx mori] | | | | | | | | | | | |
| MSTRG.19861 | -- | juvenile hormone esterase binding protein [Bombyx mori] | | | | | | | | | | | |
| MSTRG.19881 | -- | olfactory receptor [Bombyx mori] | | | | | | | | | | | |
| MSTRG.19920 | Hmcn2 | PREDICTED: Down syndrome cell adhesion molecule-like protein Dscam2 [Bombyx mori] | | | | | | | | | | | |
| MSTRG.19934 | ea | PREDICTED: serine protease easter-like [Bombyx mori] | | | | | | | | | | | |
| MSTRG.19986 | At2g35920 | PREDICTED: ATP-dependent RNA helicase DHX36-like [Athalia rosae] | | | | | | | | | | | |
| MSTRG.20036 | -- | PREDICTED: glycine-rich cell wall structural protein 1.8-like [Bombyx mori] | | | | | | | | | | | |
| MSTRG.20048 | Hsc70-5 | PREDICTED: heat shock 70 kDa protein cognate 5-like [Bombyx mori] | | | | | | | | | | | |
| MSTRG.20123 | TY3B-I | PREDICTED: uncharacterized protein K02A2.6-like [Papilio polytes] | | | | | | | | | | | |
| MSTRG.20131 | -- | olfactory receptor [Bombyx mori] | | | | | | | | | | | |
| MSTRG.2018 | -- | UPF0439 protein C9orf30-like protein [Operophtera brumata] | | | | | | | | | | | |
| MSTRG.2021 | -- | Transposon Ty3-I Gag-Pol polyprotein [Papilio xuthus] | | | | | | | | | | | |
| MSTRG.20253 | Aaas | PREDICTED: aladin-like [Bombyx mori] | | | | | | | | | | | |
| MSTRG.2029 | ING1 | Chromatin modification-related protein png2 [Papilio xuthus] | | | | | | | | | | | |
| MSTRG.20318 | -- | ku P80 DNA helicase [Danaus plexippus] | | | | | | | | | | | |
| MSTRG.20363 | Nadk | PREDICTED: NAD kinase-like isoform X4 [Bombyx mori] | | | | | | | | | | | |
| MSTRG.20364 | Nadk | PREDICTED: NAD kinase-like isoform X2 [Bombyx mori] | | | | | | | | | | | |
| MSTRG.20380 | -- | Calcium signal-modulating cyclophilin ligand [Pararge aegeria] | | | | | | | | | | | |
| MSTRG.20382 | Caf1 | PREDICTED: probable histone-binding protein Caf1 [Bombyx mori] | | | | | | | | | | | |
| MSTRG.20438 | -- | endonuclease and reverse transcriptase-like protein [Bombyx mori] | | | | | | | | | | | |
| MSTRG.2048 | Rabgef1 | PREDICTED: rab5 GDP/GTP exchange factor [Bombyx mori] | | | | | | | | | | | |
| MSTRG.20483 | CG31760 | PREDICTED: probable G-protein coupled receptor CG31760 [Bombyx mori] | | | | | | | | | | | |
| MSTRG.20484 | CG31760 | PREDICTED: protein MIS12 homolog [Papilio polytes] | | | | | | | | | | | |
| MSTRG.2049 | Rabgef1 | PREDICTED: rab5 GDP/GTP exchange factor [Bombyx mori] | | | | | | | | | | | |
| MSTRG.20534 | -- | PREDICTED: myb-like protein X [Papilio polytes] | | | | | | | | | | | |
| MSTRG.20536 | -- | peritrophin type-A domain protein 2 [Danaus plexippus] | | | | | | | | | | | |
| MSTRG.20636 | DHX36 | PREDICTED: ATP-dependent RNA helicase DHX36 [Bombyx mori] | | | | | | | | | | | |
| MSTRG.20643 | ham | MDS1 and EVI1 complex locus protein EVI1 [Papilio machaon] | | | | | | | | | | | |
| MSTRG.20709 | Zc3h12a | PREDICTED: ribonuclease ZC3H12A [Bombyx mori] | | | | | | | | | | | |
| MSTRG.20710 | Zc3h12a | PREDICTED: ribonuclease ZC3H12A [Bombyx mori] | | | | | | | | | | | |
| MSTRG.208 | -- | - | | | | | | | | | | | |
| MSTRG.20888 | -- | PREDICTED: THAP domain-containing protein 2-like [Bombyx mori] | | | | | | | | | | | |
| MSTRG.20897 | -- | PREDICTED: myb/SANT-like DNA-binding domain-containing protein 3 [Anoplophora glabripennis] | | | | | | | | | | | |
| MSTRG.20954 | Shaw | PREDICTED: potassium voltage-gated channel protein Shaw-like, partial [Halyomorpha halys] | | | | | | | | | | | |
| MSTRG.20976 | Tollo | PREDICTED: toll-like receptor 4 [Bombyx mori] | | | | | | | | | | | |
| MSTRG.21029 | pol | reverse transcriptase [Bombyx mori] | | | | | | | | | | | |
| MSTRG.21039 | -- | PREDICTED: NACHT domain- and WD repeat-containing protein 1 [Papilio xuthus] | | | | | | | | | | | |
| MSTRG.21065 | -- | PREDICTED: jerky protein homolog, partial [Vollenhovia emeryi] | | | | | | | | | | | |
| MSTRG.21162 | -- | PREDICTED: piggyBac transposable element-derived protein 4-like [Amyelois transitella] | | | | | | | | | | | |
| MSTRG.21209 | casp3 | ICE protein [Bombyx mori] | | | | | | | | | | | |
| MSTRG.21271 | CPIJ005834 | PREDICTED: elongation factor G, mitochondrial-like [Bombyx mori] | | | | | | | | | | | |
| MSTRG.21272 | ico | PREDICTED: elongation factor G, mitochondrial [Amyelois transitella] | | | | | | | | | | | |
| MSTRG.21280 | WNT-1 | Wnt-1 [Dendrolimus punctatus] | | | | | | | | | | | |
| MSTRG.21284 | -- | Retrovirus-related Pol polyprotein from transposon 17.6 [Operophtera brumata] | | | | | | | | | | | |
| MSTRG.2129 | -- | reverse transcriptase, partial [Bombyx mori] | | | | | | | | | | | |
| MSTRG.21292 | itgb1-a | integrin beta2 [Bombyx mori] | | | | | | | | | | | |
| MSTRG.21303 | -- | PREDICTED: UPF0430 protein CG31712-like [Bombyx mori] | | | | | | | | | | | |
| MSTRG.21314 | RTase | PREDICTED: RNA-directed DNA polymerase from mobile element jockey-like [Amyelois transitella] | | | | | | | | | | | |
| MSTRG.21379 | NHLRC2 | PREDICTED: NHL repeat-containing protein 2 isoform X2 [Bombyx mori] | | | | | | | | | | | |
| MSTRG.2146 | dom | PREDICTED: LOW QUALITY PROTEIN: helicase domino [Bombyx mori] | | | | | | | | | | | |
| MSTRG.21473 | Gpcpd1 | PREDICTED: glycerophosphocholine phosphodiesterase GPCPD1-like [Papilio machaon] | | | | | | | | | | | |
| MSTRG.21478 | -- | PREDICTED: lisH domain-containing protein C1711.05-like, partial [Bombyx mori] | | | | | | | | | | | |
| MSTRG.21545 | -- | PREDICTED: zinc finger protein 711-like [Halyomorpha halys] | | | | | | | | | | | |
| MSTRG.21633 | -- | buffy [Bombyx mori] | | | | | | | | | | | |
| MSTRG.21634 | -- | buffy [Bombyx mori] | | | | | | | | | | | |
| MSTRG.21637 | ATM | PREDICTED: serine-protein kinase ATM isoform X1 [Amyelois transitella] | | | | | | | | | | | |
| MSTRG.21657 | NUFIP1 | PREDICTED: nuclear fragile X mental retardation-interacting protein 1 isoform X1 [Bombyx mori] | | | | | | | | | | | |
| MSTRG.21665 | salm | PREDICTED: homeotic protein spalt-major-like isoform X2 [Bombyx mori] | | | | | | | | | | | |
| MSTRG.2167 | -- | PREDICTED: uncharacterized protein LOC101739152 isoform X2 [Bombyx mori] | | | | | | | | | | | |
| MSTRG.21683 | -- | PREDICTED: ankyrin-3-like [Amyelois transitella] | | | | | | | | | | | |
| MSTRG.21733 | -- | PREDICTED: THAP domain-containing protein 2-like [Bombyx mori] | | | | | | | | | | | |
| MSTRG.21793 | mRpL10 | PREDICTED: 39S ribosomal protein L10, mitochondrial [Papilio machaon] | | | | | | | | | | | |
| MSTRG.21837 | -- | UPF0439 protein C9orf30-like protein [Operophtera brumata] | | | | | | | | | | | |
| MSTRG.21845 | Atpaf2 | PREDICTED: ATP synthase mitochondrial F1 complex assembly factor 2 isoform X1 [Bombyx mori] | | | | | | | | | | | |
| MSTRG.21990 | CNGA2 | PREDICTED: cyclic nucleotide-gated cation channel beta-1 isoform X2 [Bombyx mori] | | | | | | | | | | | |
| MSTRG.21999 | SP3 | PREDICTED: transcription factor Sp3-like isoform X3 [Bombyx mori] | | | | | | | | | | | |
| MSTRG.22014 | PGGHG | PREDICTED: acid trehalase-like protein 1 [Bombyx mori] | | | | | | | | | | | |
| MSTRG.2203 | ACO1 | PREDICTED: cytoplasmic aconitate hydratase-like isoform X1 [Bombyx mori] | | | | | | | | | | | |
| MSTRG.2204 | PGBD4 | PREDICTED: piggyBac transposable element-derived protein 4-like isoform X1 [Bombyx mori] | | | | | | | | | | | |
| MSTRG.22070 | -- | PREDICTED: serine/Arginine-related protein 53 isoform X2 [Bombyx mori] | | | | | | | | | | | |
| MSTRG.22071 | -- | PREDICTED: serine/Arginine-related protein 53 isoform X2 [Bombyx mori] | | | | | | | | | | | |
| MSTRG.22138 | ACSL4 | PREDICTED: long-chain-fatty-acid--CoA ligase 4 isoform X2 [Bombyx mori] | | | | | | | | | | | |
| MSTRG.22177 | CACNB1 | PREDICTED: voltage-dependent L-type calcium channel subunit beta-3 isoform X7 [Papilio machaon] | | | | | | | | | | | |
| MSTRG.22178 | CACNB1 | PREDICTED: voltage-dependent L-type calcium channel subunit beta-3 isoform X8 [Papilio machaon] | | | | | | | | | | | |
| MSTRG.22188 | -- | PREDICTED: signal transducing adapter molecule 2 [Bombyx mori] | | | | | | | | | | | |
| MSTRG.22235 | STK3 | HPO [Bombyx mori] | | | | | | | | | | | |
| MSTRG.22251 | -- | PREDICTED: uncharacterized protein LOC106111803 [Papilio polytes] | | | | | | | | | | | |
| MSTRG.22258 | -- | PREDICTED: uncharacterized protein LOC101747074 isoform X2 [Bombyx mori] | | | | | | | | | | | |
| MSTRG.22359 | -- | - | | | | | | | | | | | |
| MSTRG.22363 | -- | transposase [Bombyx mori] | | | | | | | | | | | |
| MSTRG.22373 | -- | transcription initiation factor TFIID subunit 2-like protein [Heliconius erato] | | | | | | | | | | | |
| MSTRG.22374 | -- | PREDICTED: uncharacterized protein LOC106136068 [Amyelois transitella] | | | | | | | | | | | |
| MSTRG.22375 | Cib3 | PREDICTED: calcium and integrin-binding family member 2 [Bombyx mori] | | | | | | | | | | | |
| MSTRG.22379 | lin-10 | PREDICTED: amyloid beta A4 precursor protein-binding family A member 2-like isoform X7 [Anoplophora glabripennis] | | | | | | | | | | | |
| MSTRG.2238 | SLC16A12 | PREDICTED: monocarboxylate transporter 12-like [Amyelois transitella] | | | | | | | | | | | |
| MSTRG.22380 | lin-10 | PREDICTED: amyloid beta A4 precursor protein-binding family A member 2-like isoform X7 [Anoplophora glabripennis] | | | | | | | | | | | |
| MSTRG.22435 | sdn-1 | PREDICTED: syndecan isoform X1 [Bombyx mori] | | | | | | | | | | | |
| MSTRG.22442 | RAB32 | Rab-related protein [Bombyx mori] | | | | | | | | | | | |
| MSTRG.22507 | stau2 | Double-stranded RNA-binding protein Staufen-like 2 [Papilio machaon] | | | | | | | | | | | |
| MSTRG.22526 | -- | PREDICTED: RNA-binding protein 12-like isoform X3 [Papilio polytes] | | | | | | | | | | | |
| MSTRG.22529 | -- | - | | | | | | | | | | | |
| MSTRG.22531 | -- | - | | | | | | | | | | | |
| MSTRG.22552 | CPAMD8 | uncharacterized LOC106111899 [Papilio polytes] | | | | | | | | | | | |
| MSTRG.22612 | -- | endonuclease-reverse transcriptase [Bombyx mori] | | | | | | | | | | | |
| MSTRG.22615 | -- | PREDICTED: general odorant-binding protein 83a-like [Bombyx mori] | | | | | | | | | | | |
| MSTRG.22642 | MFSD12 | PREDICTED: major facilitator superfamily domain-containing protein 12-like isoform X1 [Bombyx mori] | | | | | | | | | | | |
| MSTRG.22653 | Mrto4 | PREDICTED: mRNA turnover protein 4 homolog [Papilio polytes] | | | | | | | | | | | |
| MSTRG.22680 | Snupn | PREDICTED: snurportin-1 [Bombyx mori] | | | | | | | | | | | |
| MSTRG.22686 | slc16a12b | PREDICTED: monocarboxylate transporter 5-like [Bombyx mori] | | | | | | | | | | | |
| MSTRG.22708 | MYO3A | PREDICTED: myosin-IIIb-like isoform X2 [Bombyx mori] | | | | | | | | | | | |
| MSTRG.22714 | -- | Myosin-J heavy chain [Operophtera brumata] | | | | | | | | | | | |
| MSTRG.22732 | Sb | PREDICTED: LOW QUALITY PROTEIN: transmembrane protease serine 9 [Bombyx mori] | | | | | | | | | | | |
| MSTRG.22733 | MAST2 | PREDICTED: serine/threonine-protein kinase greatwall [Bombyx mori] | | | | | | | | | | | |
| MSTRG.22734 | mastl | PREDICTED: serine/threonine-protein kinase greatwall isoform X3 [Plutella xylostella] | | | | | | | | | | | |
| MSTRG.22737 | ifi30 | legumaturain [Bombyx mori] | | | | | | | | | | | |
| MSTRG.22740 | -- | AAEL011551-PA [Aedes aegypti] | | | | | | | | | | | |
| MSTRG.22788 | AAEL002056 | PREDICTED: cytoplasmic tRNA 2-thiolation protein 2 [Bombyx mori] | | | | | | | | | | | |
| MSTRG.22797 | -- | Low-density lipoprotein receptor-related protein [Papilio xuthus] | | | | | | | | | | | |
| MSTRG.22813 | RpII215 | PREDICTED: LOW QUALITY PROTEIN: DNA-directed RNA polymerase II subunit RPB1 [Bombyx mori] | | | | | | | | | | | |
| MSTRG.22821 | EPB41L4A | PREDICTED: band 4.1-like protein 4 isoform X1 [Bombyx mori] | | | | | | | | | | | |
| MSTRG.22824 | -- | PREDICTED: zinc finger protein 624-like [Bombyx mori] | | | | | | | | | | | |
| MSTRG.22856 | PIGN | PREDICTED: GPI ethanolamine phosphate transferase 1 [Bombyx mori] | | | | | | | | | | | |
| MSTRG.22883 | -- | Tyrosine-protein phosphatase non-receptor type 13 [Papilio machaon] | | | | | | | | | | | |
| MSTRG.2289 | -- | PREDICTED: zinc finger protein 583-like [Bombyx mori] | | | | | | | | | | | |
| MSTRG.22911 | -- | PREDICTED: uncharacterized protein LOC101736207 [Bombyx mori] | | | | | | | | | | | |
| MSTRG.22988 | armi | PREDICTED: probable RNA helicase armi, partial [Bombyx mori] | | | | | | | | | | | |
| MSTRG.22995 | -- | PREDICTED: receptor-interacting serine/threonine-protein kinase 1-like [Bombyx mori] | | | | | | | | | | | |
| MSTRG.22996 | GGCT | PREDICTED: gamma-glutamylcyclotransferase-like [Bombyx mori] | | | | | | | | | | | |
| MSTRG.23014 | ptc | PREDICTED: protein patched isoform X1 [Bombyx mori] | | | | | | | | | | | |
| MSTRG.23037 | ETFA | PREDICTED: electron transfer flavoprotein subunit alpha, mitochondrial [Bombyx mori] | | | | | | | | | | | |
| MSTRG.23045 | Cacna2d3 | PREDICTED: voltage-dependent calcium channel subunit alpha-2/delta-3 isoform X4 [Bombyx mori] | | | | | | | | | | | |
| MSTRG.23050 | -- | PREDICTED: protein rolling stone-like [Bombyx mori] | | | | | | | | | | | |
| MSTRG.23078 | CG11837 | PREDICTED: probable dimethyladenosine transferase [Amyelois transitella] | | | | | | | | | | | |
| MSTRG.23117 | -- | PREDICTED: monocarboxylate transporter 1-like [Amyelois transitella] | | | | | | | | | | | |
| MSTRG.23123 | GGCT | PREDICTED: gamma-glutamylcyclotransferase-like [Bombyx mori] | | | | | | | | | | | |
| MSTRG.23137 | -- | PREDICTED: uncharacterized protein LOC100101190 isoform X2 [Bombyx mori] | | | | | | | | | | | |
| MSTRG.2318 | yellow-f2 | yellow-fa precursor [Bombyx mori] | | | | | | | | | | | |
| MSTRG.2319 | yellow-f2 | yellow-fa precursor [Bombyx mori] | | | | | | | | | | | |
| MSTRG.232 | RERG | PREDICTED: ras-related and estrogen-regulated growth inhibitor [Bombyx mori] | | | | | | | | | | | |
| MSTRG.23201 | PGBD4 | PREDICTED: piggyBac transposable element-derived protein 4-like isoform X1 [Bombyx mori] | | | | | | | | | | | |
| MSTRG.23206 | -- | PREDICTED: exportin-5-like [Plutella xylostella] | | | | | | | | | | | |
| MSTRG.23210 | -- | PREDICTED: piggyBac transposable element-derived protein 4-like isoform X4 [Bombyx mori] | | | | | | | | | | | |
| MSTRG.23218 | -- | PREDICTED: cilia- and flagella-associated protein 61 [Bombyx mori] | | | | | | | | | | | |
| MSTRG.23330 | OSBPL1A | PREDICTED: oxysterol-binding protein-related protein 1-like [Bombyx mori] | | | | | | | | | | | |
| MSTRG.234 | Gpr107 | PREDICTED: protein GPR107 [Bombyx mori] | | | | | | | | | | | |
| MSTRG.23401 | -- | PREDICTED: ankyrin-3-like [Amyelois transitella] | | | | | | | | | | | |
| MSTRG.23403 | Boc | PREDICTED: hemicentin-2-like [Bombyx mori] | | | | | | | | | | | |
| MSTRG.23440 | G3BP2 | PREDICTED: ras GTPase-activating protein-binding protein 2 [Bombyx mori] | | | | | | | | | | | |
| MSTRG.23444 | H2A.F/Z | H2A histone family member V [Bombyx mori] | | | | | | | | | | | |
| MSTRG.23489 | Pxt | PREDICTED: chorion peroxidase [Bombyx mori] | | | | | | | | | | |
| MSTRG.23490 | Pxt | PREDICTED: chorion peroxidase [Bombyx mori] | | | | | | | | | | |
| MSTRG.23529 | SV2B | PREDICTED: synaptic vesicle glycoprotein 2B-like [Bombyx mori] | | | | | | | | | | |
| MSTRG.23538 | TY3B-I | Transposon Ty3-I Gag-Pol polyprotein, partial [Anoplophora glabripennis] | | | | | | | | | | |
| MSTRG.23586 | Eif4e2 | eukaryotic initiation factor 4E-2 [Bombyx mori] | | | | | | | | | | |
| MSTRG.23612 | Cdk5rap3 | CDK5 regulatory subunit-associated protein 3 [Operophtera brumata] | | | | | | | | | | |
| MSTRG.23618 | -- | PREDICTED: uncharacterized protein LOC101738181 isoform X1 [Bombyx mori] | | | | | | | | | | |
| MSTRG.2365 | -- | PREDICTED: zinc finger protein 62 homolog isoform X5 [Bombyx mori] | | | | | | | | | | |
| MSTRG.23663 | FER | PREDICTED: tyrosine-protein kinase Fps85D isoform X2 [Bombyx mori] | | | | | | | | | | |
| MSTRG.2367 | -- | PREDICTED: uncharacterized protein LOC107044482 [Diachasma alloeum] | | | | | | | | | | |
| MSTRG.2369 | -- | PREDICTED: LOW QUALITY PROTEIN: uncharacterized protein LOC100899489 [Galendromus occidentalis] | | | | | | | | | | |
| MSTRG.23738 | vent1 | TPA_inf: Hox cluster protein Shx11 [Bombyx mori] | | | | | | | | | | |
| MSTRG.23778 | -- | Kynurenine formamidase [Papilio xuthus] | | | | | | | | | | |
| MSTRG.23780 | -- | PREDICTED: TAF5-like RNA polymerase II p300/CBP-associated factor-associated factor 65 kDa subunit 5L [Amyelois transitella] | | | | | | | | | | |
| MSTRG.23790 | TY3B-I | Transposon Ty3-I Gag-Pol polyprotein, partial [Anoplophora glabripennis] | | | | | | | | | | |
| MSTRG.23798 | -- | olfactory receptor [Bombyx mori] | | | | | | | | | | |
| MSTRG.2380 | -- | PREDICTED: zinc finger protein 16-like isoform X2 [Bombyx mori] | | | | | | | | | | |
| MSTRG.23855 | Pxd | PREDICTED: LOW QUALITY PROTEIN: peroxidase-like [Bombyx mori] | | | | | | | | | | |
| MSTRG.23910 | -- | PREDICTED: probable salivary secreted peptide [Papilio xuthus] | | | | | | | | | | |
| MSTRG.23920 | -- | PREDICTED: uncharacterized protein LOC101737832 [Bombyx mori] | | | | | | | | | | |
| MSTRG.23977 | -- | PREDICTED: balbiani ring protein 3-like isoform X2 [Bombyx mori] | | | | | | | | | | |
| MSTRG.24097 | KLKB1 | 37-kDa protease precursor [Bombyx mori] | | | | | | | | | | |
| MSTRG.2419 | -- | PREDICTED: uncharacterized protein LOC105841987 [Bombyx mori] | | | | | | | | | | |
| MSTRG.24194 | rpl1 | 39S ribosomal protein L1, mitochondrial [Papilio xuthus] | | | | | | | | | | |
| MSTRG.24208 | -- | PREDICTED: centromere protein F isoform X2 [Bombyx mori] | | | | | | | | | | |
| MSTRG.24215 | fxr1-a | PREDICTED: fragile X mental retardation syndrome-related protein 1-like [Bombyx mori] | | | | | | | | | | |
| MSTRG.24266 | -- | PREDICTED: uncharacterized protein LOC105842324 [Bombyx mori] | | | | | | | | | | |
| MSTRG.24286 | CG14701 | PREDICTED: DPH3 homolog [Bombyx mori] | | | | | | | | | | |
| MSTRG.24338 | CG1785 | CG1785-like protein [Helicoverpa armigera] | | | | | | | | | | |
| MSTRG.24349 | -- | olfactory receptor [Bombyx mori] | | | | | | | | | | |
| MSTRG.24385 | -- | PREDICTED: E3 ubiquitin-protein ligase TRAIP-like [Bombyx mori] | | | | | | | | | | |
| MSTRG.24387 | -- | PREDICTED: postreplication repair E3 ubiquitin-protein ligase RAD18-like isoform X1 [Bombyx mori] | | | | | | | | | | |
| MSTRG.244 | -- | PREDICTED: nuclear pore complex protein Nup153-like [Bombyx mori] | | | | | | | | | | |
| MSTRG.24404 | -- | PREDICTED: proline-rich extensin-like protein EPR1 [Bombyx mori] | | | | | | | | | | |
| MSTRG.24446 | -- | hypothetical protein KGM_13352 [Danaus plexippus] | | | | | | | | | | |
| MSTRG.24451 | STK40 | PREDICTED: serine/threonine-protein kinase 40-like [Bombyx mori] | | | | | | | | | | |
| MSTRG.24452 | STK40 | PREDICTED: serine/threonine-protein kinase 40-like [Bombyx mori] | | | | | | | | | | |
| MSTRG.24481 | ARHGEF17 | PREDICTED: rho guanine nucleotide exchange factor 17 isoform X2 [Bombyx mori] | | | | | | | | | | |
| MSTRG.245 | -- | PREDICTED: nuclear pore complex protein Nup153-like [Bombyx mori] | | | | | | | | | | |
| MSTRG.24509 | -- | olfactory receptor [Bombyx mori] | | | | | | | | | |
| MSTRG.24605 | CCDC174 | PREDICTED: coiled-coil domain-containing protein 174 [Bombyx mori] | | | | | | | | | |
| MSTRG.24609 | -- | Uncharacterized protein OBRU01_07814 [Operophtera brumata] | | | | | | | | | |
| MSTRG.24639 | Ccp84Ab | cuticular protein RR-2 motif 72 [Bombyx mori] | | | | | | | | | |
| MSTRG.24643 | SH3D19 | PREDICTED: SH3 domain-containing protein 19 isoform X1 [Papilio xuthus] | | | | | | | | | |
| MSTRG.24646 | -- | PREDICTED: F-box/WD repeat-containing protein 4-like isoform X1 [Bombyx mori] | | | | | | | | | |
| MSTRG.24732 | -- | Uncharacterized protein OBRU01_14843 [Operophtera brumata] | | | | | | | | | |
| MSTRG.24739 | Dlc90F | dynein molecular motor protein light chain 1 [Bombyx mori] | | | | | | | | | |
| MSTRG.2476 | -- | PREDICTED: uncharacterized protein LOC101745508 [Bombyx mori] | | | | | | | | | |
| MSTRG.24764 | -- | PREDICTED: uncharacterized protein LOC101744165 [Bombyx mori] | | | | | | | | | |
| MSTRG.24859 | GST1 | glutathione S-transferase epsilon 1 [Bombyx mori] | | | | | | | | | |
| MSTRG.24906 | FAM160B1 | PREDICTED: protein FAM160B1-like [Amyelois transitella] | | | | | | | | | |
| MSTRG.24931 | Micu3 | PREDICTED: calcium uptake protein 3, mitochondrial isoform X2 [Amyelois transitella] | | | | | | | | | |
| MSTRG.25007 | ttk | PREDICTED: zinc finger and BTB domain-containing protein 14 [Bombyx mori] | | | | | | | | | |
| MSTRG.25032 | CG1234 | PREDICTED: nucleolar complex protein 3 homolog [Bombyx mori] | | | | | | | | | |
| MSTRG.25034 | -- | Kinetochore protein NDC80-like [Papilio machaon] | | | | | | | | | |
| MSTRG.25052 | -- | olfactory receptor [Bombyx mori] | | | | | | | | | |
| MSTRG.25065 | UGP2 | PREDICTED: UTP--glucose-1-phosphate uridylyltransferase isoform X4 [Bombyx mori] | | | | | | | | | |
| MSTRG.25085 | fgf1 | PREDICTED: fibroblast growth factor 5 [Bombyx mori] | | | | | | | | | |
| MSTRG.25088 | pnc1 | PREDICTED: nicotinamidase isoform X1 [Bombyx mori] | | | | | | | | | |
| MSTRG.25112 | INF2 | PREDICTED: inverted formin-2-like [Bombyx mori] | | | | | | | | | |
| MSTRG.25157 | C38C10.2 | PREDICTED: inorganic phosphate transporter 1 isoform X1 [Bombyx mori] | | | | | | | | | |
| MSTRG.25158 | eat-4 | PREDICTED: inorganic phosphate transporter 1 isoform X1 [Bombyx mori] | | | | | | | | | |
| MSTRG.25168 | -- | PREDICTED: importin subunit alpha-2 isoform X1 [Bombyx mori] | | | | | | | | | |
| MSTRG.25170 | -- | Coiled-coil domain-containing protein 74B [Papilio machaon] | | | | | | | | | |
| MSTRG.25214 | -- | PREDICTED: multiple inositol polyphosphate phosphatase 1-like [Amyelois transitella] | | | | | | | | | |
| MSTRG.25220 | -- | PREDICTED: phosphatidylinositol-glycan biosynthesis class X protein isoform X1 [Bombyx mori] | | | | | | | | | |
| MSTRG.25264 | X-element\ORF2 | reverse transcriptase [Lasius niger] | | | | | | | | | |
| MSTRG.25400 | -- | PREDICTED: leucine-rich repeat-containing protein 1-like [Bombyx mori] | | | | | | | | | |
| MSTRG.25447 | TY3B-I | pol polyprotein [Lasius niger] | | | | | | | | | |
| MSTRG.25460 | -- | MyD88 [Spodoptera frugiperda] | | | | | | | | | |
| MSTRG.25461 | -- | geminin [Bombyx mori] | | | | | | | | | |
| MSTRG.25473 | Tf2-12 | Transposon Tf2-9 polyprotein-like Protein [Tribolium castaneum] | | | | | | | | | |
| MSTRG.25476 | -- | - | | | | | | | | | |
| MSTRG.25497 | -- | - | | | | | | | | | |
| MSTRG.25521 | Tret1 | PREDICTED: facilitated trehalose transporter Tret1 [Bombyx mori] | | | | | | | | | |
| MSTRG.25569 | UGT2B19 | UDP-glycosyltransferase UGT39C1 precursor [Bombyx mori] | | | | | | | | | |
| MSTRG.2558 | -- | PREDICTED: microtubule-associated protein futsch-like [Amyelois transitella] | | | | | | | | | |
| MSTRG.25588 | -- | olfactory receptor [Bombyx mori] | | | | | | | | | |
| MSTRG.25589 | RCJMB04_18o22 | PREDICTED: UPF0454 protein C12orf49 homolog [Bombyx mori] | | | | | | | | |
| MSTRG.2559 | -- | PREDICTED: uncharacterized protein LOC101736082 [Bombyx mori] | | | | | | | | |
| MSTRG.25703 | TCF15 | PREDICTED: transcription factor 15 [Bombyx mori] | | | | | | | | |
| MSTRG.25751 | PSMD2 | PREDICTED: 26S proteasome non-ATPase regulatory subunit 2 isoform X1 [Bombyx mori] | | | | | | | | |
| MSTRG.25752 | PSMD2 | PREDICTED: 26S proteasome non-ATPase regulatory subunit 2 isoform X1 [Bombyx mori] | | | | | | | | |
| MSTRG.25774 | Lis-1 | PREDICTED: LOW QUALITY PROTEIN: lissencephaly-1 homolog [Bombyx mori] | | | | | | | | |
| MSTRG.25783 | -- | reverse transcriptase, partial [Bombyx mori] | | | | | | | | |
| MSTRG.25786 | -- | PREDICTED: RNA-directed DNA polymerase from mobile element jockey-like isoform X5 [Papilio machaon] | | | | | | | | |
| MSTRG.25859 | -- | olfactory receptor [Bombyx mori] | | | | | | | | |
| MSTRG.25860 | PSPH | PREDICTED: phosphoserine phosphatase isoform X1 [Bombyx mori] | | | | | | | | |
| MSTRG.25862 | PSPH | PREDICTED: phosphoserine phosphatase isoform X1 [Bombyx mori] | | | | | | | | |
| MSTRG.25875 | -- | PREDICTED: probable G-protein coupled receptor Mth-like 2 [Bombyx mori] | | | | | | | | |
| MSTRG.25877 | mthl3 | PREDICTED: G-protein coupled receptor Mth2-like [Amyelois transitella] | | | | | | | | |
| MSTRG.26011 | pol | Transposon Tf2-9 polyprotein-like Protein [Tribolium castaneum] | | | | | | | | |
| MSTRG.26012 | chif | chiffon, isoform A [Danaus plexippus] | | | | | | | | |
| MSTRG.26013 | chif | chiffon, isoform A [Danaus plexippus] | | | | | | | | |
| MSTRG.26144 | allc | PREDICTED: allantoicase-like, partial [Bombyx mori] | | | | | | | | |
| MSTRG.2619 | -- | PREDICTED: ribonuclease H2 subunit C [Plutella xylostella] | | | | | | | | |
| MSTRG.2620 | -- | PREDICTED: PCNA-associated factor-like [Bombyx mori] | | | | | | | | |
| MSTRG.2622 | Gtpbp2 | GTP-binding protein 2 [Papilio xuthus] | | | | | | | | |
| MSTRG.26220 | pol | reverse transcriptase-like protein [Bombyx mori] | | | | | | | | |
| MSTRG.2623 | Gtpbp2 | GTP-binding protein 2 [Papilio xuthus] | | | | | | | | |
| MSTRG.26240 | -- | PREDICTED: meiosis-specific nuclear structural protein 1-like [Bombyx mori] | | | | | | | | |
| MSTRG.26241 | -- | PREDICTED: meiosis-specific nuclear structural protein 1-like [Bombyx mori] | | | | | | | | |
| MSTRG.26244 | -- | olfactory receptor [Bombyx mori] | | | | | | | | |
| MSTRG.26250 | -- | hypothetical protein L798_04777 [Zootermopsis nevadensis] | | | | | | | | |
| MSTRG.26253 | ppk28 | PREDICTED: pickpocket protein 28-like [Bombyx mori] | | | | | | | | |
| MSTRG.26310 | -- | acetylcholinesterase (ace) gene [Pararge aegeria] | | | | | | | | |
| MSTRG.26373 | Vps13 | PREDICTED: vacuolar protein sorting-associated protein 13C [Bombyx mori] | | | | | | | | |
| MSTRG.26374 | Vps13 | PREDICTED: vacuolar protein sorting-associated protein 13C [Bombyx mori] | | | | | | | | |
| MSTRG.26448 | MTFMT | PREDICTED: methionyl-tRNA formyltransferase, mitochondrial [Bombyx mori] | | | | | | | | |
| MSTRG.26467 | CCDC58 | PREDICTED: UPF0389 protein CG9231 [Bombyx mori] | | | | | | | | |
| MSTRG.26493 | -- | PREDICTED: uncharacterized protein LOC106134622 [Amyelois transitella] | | | | | | | | |
| MSTRG.26496 | Ace | acetylcholinesterase type 2 [Bombyx mori] | | | | | | | | |
| MSTRG.26499 | -- | PREDICTED: muscle M-line assembly protein unc-89-like [Amyelois transitella] | | | | | | | | |
| MSTRG.26500 | FAM76A | PREDICTED: LOW QUALITY PROTEIN: protein FAM76A [Bombyx mori] | | | | | | | | |
| MSTRG.26518 | Arsj | PREDICTED: arylsulfatase B-like [Bombyx mori] | | | | | | | | |
| MSTRG.26541 | mal | molybdenum cofactor sulfurase [Bombyx mori] | | | | | | | | |
| MSTRG.26581 | BBXB3 | insulin-like precursor polypeptide 2 [Spodoptera littoralis] | | | | | | | | |
| MSTRG.26582 | -- | PREDICTED: bombyxin A-1 homolog [Bombyx mori] | | | | | | | | |
| MSTRG.26585 | -- | PREDICTED: bombyxin A-1 homolog [Bombyx mori] | | | | | | | |
| MSTRG.26676 | C15orf61 | olfactory receptor 65 [Bombyx mori] | | | | | | | |
| MSTRG.26719 | -- | PREDICTED: major facilitator superfamily domain-containing protein 9-like [Bombyx mori] | | | | | | | |
| MSTRG.26758 | -- | PREDICTED: TELO2-interacting protein 2-like [Bombyx mori] | | | | | | | |
| MSTRG.26759 | Rev3l | PREDICTED: DNA polymerase zeta catalytic subunit isoform X1 [Bombyx mori] | | | | | | | |
| MSTRG.26824 | -- | - | | | | | | | |
| MSTRG.26873 | -- | olfactory receptor [Bombyx mori] | | | | | | | |
| MSTRG.26874 | CenG1A | PREDICTED: centaurin-gamma-1A [Bombyx mori] | | | | | | | |
| MSTRG.26897 | -- | nimrod B precursor [Bombyx mori] | | | | | | | |
| MSTRG.26946 | zfh1 | PREDICTED: zinc finger protein 1 isoform X2 [Bombyx mori] | | | | | | | |
| MSTRG.2701 | ADH5 | PREDICTED: alcohol dehydrogenase isoform X1 [Bombyx mori] | | | | | | | |
| MSTRG.2874 | -- | PREDICTED: uncharacterized protein LOC106717337 [Papilio machaon] | | | | | | | |
| MSTRG.2912 | -- | PREDICTED: acidic leucine-rich nuclear phosphoprotein 32-related protein-like [Bombyx mori] | | | | | | | |
| MSTRG.2958 | fdl | PREDICTED: beta-N-acetylglucosaminidase 1 isoform X1 [Bombyx mori] | | | | | | | |
| MSTRG.2967 | -- | olfactory receptor [Bombyx mori] | | | | | | | |
| MSTRG.297 | AAEL003481 | PREDICTED: glycosyltransferase 25 family member isoform X1 [Amyelois transitella] | | | | | | | |
| MSTRG.2980 | TMEM184B | PREDICTED: transmembrane protein 184B isoform X2 [Bombyx mori] | | | | | | | |
| MSTRG.2981 | TMEM184B | PREDICTED: transmembrane protein 184B isoform X2 [Bombyx mori] | | | | | | | |
| MSTRG.2989 | DUSP22 | PREDICTED: dual specificity protein phosphatase 22 isoform X2 [Bombyx mori] | | | | | | | |
| MSTRG.3028 | CNIH4 | PREDICTED: protein cornichon homolog 4 [Bombyx mori] | | | | | | | |
| MSTRG.3072 | -- | PREDICTED: putative nuclease HARBI1, partial [Bombyx mori] | | | | | | | |
| MSTRG.3086 | SLC25A17 | PREDICTED: peroxisomal membrane protein PMP34-like isoform X2 [Bombyx mori] | | | | | | | |
| MSTRG.3174 | CYP15C1 | RecName: Full=Farnesoate epoxidase; AltName: Full=Cytochrome P450 15C1; AltName: Full=Protein dimolting; Short=mod; Flags: Precursor | | | | | | | |
| MSTRG.3177 | elg1 | PREDICTED: ATPase family AAA domain-containing protein 5 [Amyelois transitella] | | | | | | | |
| MSTRG.3182 | DIS3L2 | PREDICTED: DIS3-like exonuclease 2 [Plutella xylostella] | | | | | | | |
| MSTRG.3247 | ATFC | activating transcription factor of chaperone [Bombyx mori] | | | | | | | |
| MSTRG.3410 | Lar | PREDICTED: tyrosine-protein phosphatase Lar [Amyelois transitella] | | | | | | | |
| MSTRG.3442 | CG7457 | PREDICTED: tonsoku-like protein [Amyelois transitella] | | | | | | | |
| MSTRG.3451 | COL15A1 | PREDICTED: collagen alpha-1(XVIII) chain-like [Bombyx mori] | | | | | | | |
| MSTRG.3480 | RIOK1 | PREDICTED: serine/threonine-protein kinase RIO1 [Bombyx mori] | | | | | | | |
| MSTRG.3495 | -- | uncharacterized LOC106121242 [Papilio xuthus] | | | | | | | |
| MSTRG.351 | RTase | PREDICTED: RNA-directed DNA polymerase from mobile element jockey-like [Amyelois transitella] | | | | | | | |
| MSTRG.3566 | knrl | PREDICTED: knirps-related protein-like [Papilio machaon] | | | | | | | |
| MSTRG.3604 | -- | serpin-24, partial [Bombyx mori] | | | | | | | |
| MSTRG.3692 | Tnpo3 | PREDICTED: importin-13 [Amyelois transitella] | | | | | | | |
| MSTRG.3723 | -- | cuticular protein RR-1 motif 55 precursor [Bombyx mori] | | | | | | | |
| MSTRG.3730 | -- | PREDICTED: uncharacterized protein LOC101737213 [Bombyx mori] | | | | | | |
| MSTRG.376 | -- | PREDICTED: uncharacterized protein LOC106111934 [Papilio polytes] | | | | | | |
| MSTRG.3805 | -- | reverse transcriptase, partial [Bombyx mori] | | | | | | |
| MSTRG.3832 | Acyp2 | PREDICTED: acylphosphatase-2 [Bombyx mori] | | | | | | |
| MSTRG.3908 | -- | PREDICTED: serine/arginine repetitive matrix protein 1 [Papilio xuthus] | | | | | | |
| MSTRG.3948 | Mp20 | PREDICTED: myophilin [Bombyx mori] | | | | | | |
| MSTRG.3953 | -- | hypothetical protein RR48_13816 [Papilio machaon] | | | | | | |
| MSTRG.3989 | -- | PREDICTED: androgen-dependent TFPI-regulating protein-like [Bombyx mori] | | | | | | |
| MSTRG.4043 | Mrpl46 | PREDICTED: 39S ribosomal protein L46, mitochondrial [Bombyx mori] | | | | | | |
| MSTRG.4045 | dpp | PREDICTED: decapentaplegic isoform X1 [Bombyx mori] | | | | | | |
| MSTRG.4060 | -- | chitin deacetylase 4 [Cnaphalocrocis medinalis] | | | | | | |
| MSTRG.4132 | -- | PREDICTED: galectin-9-like isoform X1 [Bombyx mori] | | | | | | |
| MSTRG.4133 | -- | PREDICTED: PR domain zinc finger protein 10-like isoform X2 [Bombyx mori] | | | | | | |
| MSTRG.4208 | clptm1 | PREDICTED: cleft lip and palate transmembrane protein 1 homolog [Amyelois transitella] | | | | | | |
| MSTRG.4238 | -- | - | | | | | | |
| MSTRG.425 | -- | transposase [Bombyx mori] | | | | | | |
| MSTRG.4255 | Washc4 | PREDICTED: WASH complex subunit 7-like [Plutella xylostella] | | | | | | |
| MSTRG.4271 | X-element\ORF2 | reverse transcriptase, partial [Bombyx mori] | | | | | | |
| MSTRG.4284 | desat1 | stearoyl-CoA desaturase 5 [Bombyx mori] | | | | | | |
| MSTRG.4285 | desat1 | stearoyl-CoA desaturase 5 [Bombyx mori] | | | | | | |
| MSTRG.4307 | wdpcp | PREDICTED: WD repeat-containing and planar cell polarity effector protein fritz homolog isoform X2 [Bombyx mori] | | | | | | |
| MSTRG.4333 | TFAP4 | transcription factor AP-4 [Bombyx mori] | | | | | | |
| MSTRG.4335 | -- | PREDICTED: FK506-binding protein 4 [Bombyx mori] | | | | | | |
| MSTRG.4336 | -- | PREDICTED: TNF receptor-associated factor family protein DDB_G0272098-like, partial [Bombyx mori] | | | | | | |
| MSTRG.441 | Prss16 | PREDICTED: putative serine protease K12H4.7 [Bombyx mori] | | | | | | |
| MSTRG.4442 | -- | TAF6-like RNA polymerase II p300/CBP-associated factor-associated factor 65 kDa subunit 6L [Papilio xuthus] | | | | | | |
| MSTRG.4472 | -- | PREDICTED: uncharacterized protein LOC106717337 [Papilio machaon] | | | | | | |
| MSTRG.4483 | -- | PREDICTED: fibrinogen silencer-binding protein-like [Anoplophora glabripennis] | | | | | | |
| MSTRG.45 | ab | PREDICTED: protein tramtrack, beta isoform-like isoform X3 [Bombyx mori] | | | | | | |
| MSTRG.4510 | ACSF2 | PREDICTED: acyl-CoA synthetase family member 2, mitochondrial [Bombyx mori] | | | | | | |
| MSTRG.4511 | ACSF2 | PREDICTED: acyl-CoA synthetase family member 2, mitochondrial [Bombyx mori] | | | | | | |
| MSTRG.4521 | EI24 | PREDICTED: etoposide-induced protein 2.4 homolog [Bombyx mori] | | | | | | |
| MSTRG.4550 | br | PREDICTED: protein bric-a-brac 1-like isoform X3 [Bombyx mori] | | | | | | |
| MSTRG.4555 | -- | PREDICTED: ubiquitin-like-conjugating enzyme ATG10 [Bombyx mori] | | | | | | |
| MSTRG.4581 | CELF4 | PREDICTED: CUGBP Elav-like family member 5 [Papilio polytes] | | | | | | |
| MSTRG.4583 | -- | UPF0439 protein C9orf30-like protein [Operophtera brumata] | | | | | | |
| MSTRG.4628 | -- | neurotransmitter gated ion channel [Danaus plexippus] | | | | | | |
| MSTRG.4662 | -- | PREDICTED: uncharacterized protein LOC106708887 [Papilio machaon] | | | | | | |
| MSTRG.4687 | CG7137 | PREDICTED: ribosomal RNA-processing protein 8 [Bombyx mori] | | | | | | |
| MSTRG.4712 | -- | PREDICTED: ankyrin-3-like [Amyelois transitella] | | | | | |
| MSTRG.4746 | Pwp2 | PREDICTED: periodic tryptophan protein 2 homolog [Bombyx mori] | | | | | |
| MSTRG.4772 | -- | PREDICTED: mucin-17-like isoform X9 [Bombyx mori] | | | | | |
| MSTRG.4826 | PGRMC1 | PREDICTED: membrane-associated progesterone receptor component 1-like [Papilio machaon] | | | | | |
| MSTRG.4859 | -- | Retrotransposable element Tf2 155 kDa protein type 3, partial [Pararge aegeria] | | | | | |
| MSTRG.4881 | Tmlhe | PREDICTED: trimethyllysine dioxygenase, mitochondrial [Bombyx mori] | | | | | |
| MSTRG.4922 | -- | olfactory receptor [Bombyx mori] | | | | | |
| MSTRG.4923 | -- | PREDICTED: lipopolysaccharide-induced tumor necrosis factor-alpha factor homolog [Bombyx mori] | | | | | |
| MSTRG.4968 | VAMP7 | PREDICTED: vesicle-associated membrane protein 7-like isoform X1 [Bombyx mori] | | | | | |
| MSTRG.4990 | -- | PREDICTED: uncharacterized protein LOC105842795 [Bombyx mori] | | | | | |
| MSTRG.5028 | Ift88 | Intraflagellar transport protein 88-like [Papilio machaon] | | | | | |
| MSTRG.5081 | -- | PREDICTED: FAST kinase domain-containing protein 5 [Bombyx mori] | | | | | |
| MSTRG.514 | -- | endonuclease-reverse transcriptase [Bombyx mori] | | | | | |
| MSTRG.518 | CES1 | beta-esterase 2 precursor [Bombyx mori] | | | | | |
| MSTRG.5232 | -- | UPF0439 protein C9orf30-like protein [Operophtera brumata] | | | | | |
| MSTRG.5233 | -- | PREDICTED: alkaline nuclease isoform X1 [Bombyx mori] | | | | | |
| MSTRG.5250 | -- | PREDICTED: zinc finger protein 711-like [Halyomorpha halys] | | | | | |
| MSTRG.5257 | -- | endonuclease-reverse transcriptase [Bombyx mori] | | | | | |
| MSTRG.5283 | -- | olfactory receptor [Bombyx mori] | | | | | |
| MSTRG.5302 | -- | - | | | | | |
| MSTRG.5310 | Nup50 | PREDICTED: nuclear pore complex protein Nup50 [Bombyx mori] | | | | | |
| MSTRG.5363 | -- | PREDICTED: probable endochitinase [Bombyx mori] | | | | | |
| MSTRG.538 | Pdp1 | PREDICTED: pyruvate dehydrogenase [acetyl-transferring]-phosphatase 1, mitochondrial isoform X1 [Bombyx mori] | | | | | |
| MSTRG.5388 | -- | PREDICTED: gastrula zinc finger protein XlCGF17.1-like [Bombyx mori] | | | | | |
| MSTRG.5397 | -- | Signal peptidase complex subunit 3, partial [Operophtera brumata] | | | | | |
| MSTRG.5429 | CG7990 | PREDICTED: post-GPI attachment to proteins factor 2-like [Bombyx mori] | | | | | |
| MSTRG.5466 | -- | m7GpppN-mRNA hydrolase [Bombyx mori] | | | | | |
| MSTRG.5517 | -- | olfactory receptor [Bombyx mori] | | | | | |
| MSTRG.5598 | prkra | PREDICTED: R2D2 protein isoform X1 [Bombyx mori] | | | | | |
| MSTRG.5606 | -- | PREDICTED: RNA-directed DNA polymerase from mobile element jockey-like [Amyelois transitella] | | | | | |
| MSTRG.562 | CG12262 | PREDICTED: probable medium-chain specific acyl-CoA dehydrogenase, mitochondrial isoform X2 [Bombyx mori] | | | | | |
| MSTRG.563 | CG12262 | PREDICTED: probable medium-chain specific acyl-CoA dehydrogenase, mitochondrial isoform X1 [Bombyx mori] | | | | | |
| MSTRG.5647 | pol | non-LTR retrotransposon CATS, partial [Bombyx mori] | | | | | |
| MSTRG.5704 | AIFM1 | PREDICTED: apoptosis-inducing factor 1, mitochondrial-like [Bombyx mori] | | | | | |
| MSTRG.5707 | -- | PREDICTED: hamartin [Bombyx mori] | | | | | |
| MSTRG.5807 | VTC5 | PREDICTED: GDP-D-glucose phosphorylase 1 [Bombyx mori] | | | | | |
| MSTRG.5812 | -- | PREDICTED: mucin-2-like isoform X1 [Papilio xuthus] | | | | |
| MSTRG.5852 | tsl | PREDICTED: torso-like protein [Bombyx mori] | | | | |
| MSTRG.5853 | tsl | PREDICTED: torso-like protein [Bombyx mori] | | | | |
| MSTRG.5871 | gol | PREDICTED: protein goliath isoform X1 [Bombyx mori] | | | | |
| MSTRG.5896 | -- | - | | | | |
| MSTRG.5937 | -- | PREDICTED: cyclin-dependent kinase inhibitor 1 isoform X2 [Harpegnathos saltator] | | | | |
| MSTRG.597 | zdhhc16a | PREDICTED: probable palmitoyltransferase ZDHHC16 [Bombyx mori] | | | | |
| MSTRG.6022 | pol | Retrovirus-related Pol polyprotein from transposon 412-like Protein [Tribolium castaneum] | | | | |
| MSTRG.6023 | RTase | reverse transcriptase [Bombyx mori] | | | | |
| MSTRG.6024 | THAP9 | mariner transposase [Bombyx mori] | | | | |
| MSTRG.6025 | THAP9 | Retrotransposable element Tf2 155 kDa protein type 1 [Papilio machaon] | | | | |
| MSTRG.6030 | -- | olfactory receptor [Bombyx mori] | | | | |
| MSTRG.6033 | pol | Retrovirus-related Pol polyprotein from transposon 17.6 [Operophtera brumata] | | | | |
| MSTRG.6214 | -- | reverse transcriptase, partial [Bombyx mori] | | | | |
| MSTRG.6215 | -- | reverse transcriptase, partial [Bombyx mori] | | | | |
| MSTRG.6224 | -- | PREDICTED: retrovirus-related Pol polyprotein from transposon TNT 1-94 [Bombyx mori] | | | | |
| MSTRG.6225 | PGBD4 | PREDICTED: piggyBac transposable element-derived protein 4-like isoform X1 [Bombyx mori] | | | | |
| MSTRG.6241 | HMCN1 | PREDICTED: hemicentin-1-like [Bombyx mori] | | | | |
| MSTRG.6268 | -- | PREDICTED: uncharacterized protein LOC105386827 [Plutella xylostella] | | | | |
| MSTRG.6311 | -- | Carboxypeptidase N subunit 2 [Operophtera brumata] | | | | |
| MSTRG.6364 | -- | Gag-pol polyprotein [Operophtera brumata] | | | | |
| MSTRG.640 | -- | Histone-lysine N-methyltransferase SETMAR, partial [Habropoda laboriosa] | | | | |
| MSTRG.6447 | -- | ATP-dependent helicase [Bombyx mori] | | | | |
| MSTRG.6574 | -- | PREDICTED: mucin-2-like [Amyelois transitella] | | | | |
| MSTRG.6579 | -- | PREDICTED: chondroitin proteoglycan-2-like [Bombyx mori] | | | | |
| MSTRG.6632 | cac | PREDICTED: voltage-dependent calcium channel type A subunit alpha-1 isoform X2 [Bombyx mori] | | | | |
| MSTRG.6658 | CCDC28A | PREDICTED: coiled-coil domain-containing protein 28B [Bombyx mori] | | | | |
| MSTRG.667 | -- | olfactory receptor [Bombyx mori] | | | | |
| MSTRG.6671 | CG42684 | PREDICTED: probable Ras GTPase-activating protein isoform X3 [Amyelois transitella] | | | | |
| MSTRG.6673 | CG42684 | PREDICTED: probable Ras GTPase-activating protein isoform X1 [Amyelois transitella] | | | | |
| MSTRG.6686 | Rop | PREDICTED: protein ROP isoform X1 [Bombyx mori] | | | | |
| MSTRG.6696 | SCYL3 | PREDICTED: protein-associating with the carboxyl-terminal domain of ezrin [Bombyx mori] | | | | |
| MSTRG.6711 | CG3527 | Nep1 protein [Bombyx mori] | | | | |
| MSTRG.6742 | -- | - | | | | |
| MSTRG.6747 | Nup54 | PREDICTED: probable nucleoporin Nup54 isoform X1 [Bombyx mori] | | | | |
| MSTRG.675 | CG12262 | PREDICTED: probable medium-chain specific acyl-CoA dehydrogenase, mitochondrial isoform X2 [Bombyx mori] | | | |
| MSTRG.6755 | -- | Death domain-associated protein 6 [Papilio xuthus] | | | |
| MSTRG.6756 | -- | Death domain-associated protein 6 [Papilio xuthus] | | | |
| MSTRG.6768 | -- | - | | | |
| MSTRG.6775 | obst-E | Obstructor-E [Operophtera brumata] | | | |
| MSTRG.6777 | -- | PREDICTED: chondroitin proteoglycan 2-like [Plutella xylostella] | | | |
| MSTRG.6780 | -- | PREDICTED: leucine-rich repeat extensin-like protein 5 [Bombyx mori] | | | |
| MSTRG.6789 | FNBP1 | PREDICTED: formin-binding protein 1-like isoform X1 [Bombyx mori] | | | |
| MSTRG.6790 | nrf-6 | PREDICTED: nose resistant to fluoxetine protein 6-like [Bombyx mori] | | | |
| MSTRG.6793 | Cyp6g2 | cytochrome P450 CYP332A1 [Bombyx mori] | | | |
| MSTRG.680 | -- | PREDICTED: polyhomeotic-proximal chromatin protein-like [Amyelois transitella] | | | |
| MSTRG.6810 | AGAP005199 | ubiquinone biosynthesis protein COQ4 homolog, mitochondrial [Bombyx mori] | | | |
| MSTRG.6817 | Brd2 | PREDICTED: homeotic protein female sterile isoform X1 [Bombyx mori] | | | |
| MSTRG.6818 | Brd2 | PREDICTED: homeotic protein female sterile isoform X1 [Bombyx mori] | | | |
| MSTRG.6823 | AGPAT1 | 1-acylglycerol-3-phosphate O-acyltransferase 2 [Bombyx mori] | | | |
| MSTRG.6851 | dare | PREDICTED: NADPH:adrenodoxin oxidoreductase, mitochondrial [Bombyx mori] | | | |
| MSTRG.6864 | nas-13 | PREDICTED: zinc metalloproteinase nas-4-like isoform X1 [Bombyx mori] | | | |
| MSTRG.6865 | nas-15 | PREDICTED: zinc metalloproteinase nas-4-like isoform X2 [Bombyx mori] | | | |
| MSTRG.6870 | -- | PREDICTED: mucin-5AC [Bombyx mori] | | | |
| MSTRG.6871 | -- | PREDICTED: mucin-5AC [Bombyx mori] | | | |
| MSTRG.6882 | -- | Peptidyl-prolyl cis-trans isomerase [Operophtera brumata] | | | |
| MSTRG.6893 | RIMS2 | PREDICTED: regulating synaptic membrane exocytosis protein 2 isoform X4 [Bombyx mori] | | | |
| MSTRG.692 | -- | mariner transposase [Bombyx mori] | | | |
| MSTRG.6929 | mTerf5 | PREDICTED: uncharacterized protein LOC101736537 [Bombyx mori] | | | |
| MSTRG.6941 | -- | PREDICTED: uncharacterized protein LOC101744707 [Bombyx mori] | | | |
| MSTRG.6957 | -- | PREDICTED: zinc finger protein 711-like [Halyomorpha halys] | | | |
| MSTRG.698 | PGBD4 | mariner transposase [Bombyx mori] | | | |
| MSTRG.6983 | SETMAR | PREDICTED: golgin subfamily A member 4-like [Bombyx mori] | | | |
| MSTRG.6984 | Golga4 | PREDICTED: golgin subfamily A member 4-like [Bombyx mori] | | | |
| MSTRG.6998 | Commd2 | PREDICTED: COMM domain-containing protein 2-like isoform X3 [Bombyx mori] | | | |
| MSTRG.700 | PGBD4 | mariner transposase [Bombyx mori] | | | |
| MSTRG.7035 | -- | PREDICTED: zinc finger CCHC domain-containing protein 24-like [Bombyx mori] | | | |
| MSTRG.7072 | -- | PREDICTED: uncharacterized protein LOC101747025 [Bombyx mori] | | | |
| MSTRG.7075 | Flnc | PREDICTED: filamin-A isoform X1 [Bombyx mori] | | | |
| MSTRG.7076 | Flnc | PREDICTED: filamin-A isoform X1 [Bombyx mori] | | | |
| MSTRG.7126 | -- | PREDICTED: sodium-dependent noradrenaline transporter-like isoform X2 [Bombyx mori] | | | |
| MSTRG.7205 | ITIH4 | PREDICTED: inter-alpha-trypsin inhibitor heavy chain H4-like isoform X1 [Bombyx mori] | | | |
| MSTRG.7240 | YTHDF3 | PREDICTED: YTH domain-containing family protein 3 isoform X2 [Bombyx mori] | | | |
| MSTRG.7241 | YTHDF3 | PREDICTED: YTH domain-containing family protein 3 isoform X2 [Bombyx mori] | | |
| MSTRG.7284 | ACPP | PREDICTED: prostatic acid phosphatase [Bombyx mori] | | |
| MSTRG.729 | TBC1D13 | PREDICTED: TBC1 domain family member 13 [Bombyx mori] | | |
| MSTRG.7296 | Tim14 | DnaJ domain-containing protein [Bombyx mori] | | |
| MSTRG.7304 | -- | Protein KIAA1731 [Papilio xuthus] | | |
| MSTRG.731 | -- | - | | |
| MSTRG.7387 | URH2 | salivary purine nucleosidase [Pararge aegeria] | | |
| MSTRG.7396 | RTase | PREDICTED: RNA-directed DNA polymerase from mobile element jockey-like, partial [Papilio machaon] | | |
| MSTRG.7400 | -- | serine protease inhibitor 22 precursor [Bombyx mori] | | |
| MSTRG.743 | scap | PREDICTED: sterol regulatory element-binding protein cleavage-activating protein [Bombyx mori] | | |
| MSTRG.7452 | EXD2 | PREDICTED: exonuclease 3'-5' domain-containing protein 2 [Bombyx mori] | | |
| MSTRG.7519 | -- | AGAP004528-PA [Anopheles gambiae str. PEST] [Anopheles gambiae] | | |
| MSTRG.7528 | -- | PREDICTED: filaggrin-2 [Bombyx mori] | | |
| MSTRG.7540 | pol | Transposon Tf2-9 polyprotein-like Protein [Tribolium castaneum] | | |
| MSTRG.7545 | -- | carboxypeptidase inhibitor precursor [Bombyx mori] | | |
| MSTRG.7613 | sgsm3 | PREDICTED: small G protein signaling modulator 3 homolog [Amyelois transitella] | | |
| MSTRG.7616 | -- | PREDICTED: fibrous sheath CABYR-binding protein-like [Bombyx mori] | | |
| MSTRG.7657 | -- | PREDICTED: uncharacterized protein LOC101742506 [Bombyx mori] | | |
| MSTRG.7671 | to | PREDICTED: protein takeout-like [Bombyx mori] | | |
| MSTRG.771 | acp2 | PREDICTED: prostatic acid phosphatase-like [Bombyx mori] | | |
| MSTRG.7710 | LIMK1 | PREDICTED: LIM domain kinase 1 [Bombyx mori] | | |
| MSTRG.781 | Slc7a14 | PREDICTED: cationic amino acid transporter 2 [Bombyx mori] | | |
| MSTRG.783 | Slc7a14 | PREDICTED: cationic amino acid transporter 2 [Bombyx mori] | | |
| MSTRG.7991 | TTC28 | PREDICTED: tetratricopeptide repeat protein 28 [Bombyx mori] | | |
| MSTRG.8014 | -- | hypothetical protein g.14677 [Pectinophora gossypiella] | | |
| MSTRG.8020 | Ptpn9 | PREDICTED: alpha-tocopherol transfer protein-like [Bombyx mori] | | |
| MSTRG.8028 | -- | PREDICTED: uncharacterized protein LOC101736812 [Bombyx mori] | | |
| MSTRG.8066 | Abhd18 | PREDICTED: protein ABHD18-like [Aedes albopictus] | | |
| MSTRG.8083 | Gld | PREDICTED: glucose dehydrogenase [FAD, quinone]-like [Bombyx mori] | | |
| MSTRG.8099 | -- | Hipothetical protein [Operophtera brumata] | | |
| MSTRG.8116 | vmp1 | PREDICTED: vacuole membrane protein 1 isoform X1 [Bombyx mori] | | |
| MSTRG.8120 | HSCB | PREDICTED: iron-sulfur cluster co-chaperone protein HscB, mitochondrial [Papilio xuthus] | | |
| MSTRG.8146 | -- | Alpha-tocopherol transfer protein [Papilio xuthus] | | |
| MSTRG.8151 | -- | CRAL-TRIO domain-containing protein, partial [Manduca sexta] | | |
| MSTRG.8171 | kz | PREDICTED: probable ATP-dependent RNA helicase kurz [Bombyx mori] | | |
| MSTRG.8172 | -- | hypothetical protein g.7559 [Pectinophora gossypiella] | | |
| MSTRG.8252 | paxip1 | PREDICTED: PAX-interacting protein 1 [Bombyx mori] | | |
| MSTRG.8253 | paxip1 | PREDICTED: PAX-interacting protein 1 [Bombyx mori] | | |
| MSTRG.8256 | MYO18B | PREDICTED: unconventional myosin-XVIIIa isoform X1 [Amyelois transitella] | |
| MSTRG.8269 | Pgam5 | cytochrome P450 CYP367B2 [Helicoverpa armigera] | |
| MSTRG.8293 | AAEL008004 | PREDICTED: elongation of very long chain fatty acids protein AAEL008004-like [Bombyx mori] | |
| MSTRG.8321 | SDR-1 | PREDICTED: farnesol dehydrogenase-like isoform X1 [Bombyx mori] | |
| MSTRG.8389 | X-element\ORF2 | reverse transcriptase [Bombyx mori] | |
| MSTRG.8547 | -- | PREDICTED: ankyrin-3-like [Amyelois transitella] | |
| MSTRG.8555 | PHF12 | PHD finger protein 12 [Papilio xuthus] | |
| MSTRG.8653 | Gld | ecdysone oxidase [Bombyx mori] | |
| MSTRG.8671 | -- | PREDICTED: LOW QUALITY PROTEIN: myosin heavy chain, non-muscle [Bombyx mori] | |
| MSTRG.8672 | -- | PREDICTED: LOW QUALITY PROTEIN: myosin heavy chain, non-muscle [Bombyx mori] | |
| MSTRG.8689 | Ggamma30A | G protein gamma subunit [Bombyx mori] | |
| MSTRG.8695 | -- | PREDICTED: probable chitinase 3 [Bombyx mori] | |
| MSTRG.8729 | CHIA | PREDICTED: probable chitinase 3 [Bombyx mori] | |
| MSTRG.8788 | AATK | serine/threonine-protein kinase (LMTK1) protein [Heliconius erato] | |
| MSTRG.8861 | -- | Aminoacylase-1 [Operophtera brumata] | |
| MSTRG.89 | Napg | PREDICTED: gamma-soluble NSF attachment protein-like [Bombyx mori] | |
| MSTRG.8954 | -- | - | |
| MSTRG.8973 | asp | PREDICTED: LOW QUALITY PROTEIN: protein abnormal spindle [Bombyx mori] | |
| MSTRG.8974 | asp | PREDICTED: LOW QUALITY PROTEIN: protein abnormal spindle [Bombyx mori] | |
| MSTRG.90 | Napg | PREDICTED: gamma-soluble NSF attachment protein-like [Bombyx mori] | |
| MSTRG.9020 | asun | PREDICTED: protein asunder-like [Bombyx mori] | |
| MSTRG.9098 | MARF1 | PREDICTED: meiosis arrest female protein 1 homolog [Bombyx mori] | |
| MSTRG.9099 | MARF1 | PREDICTED: meiosis arrest female protein 1 homolog [Bombyx mori] | |
| MSTRG.9113 | -- | Retrovirus-related Pol polyprotein from transposon 17.6 [Operophtera brumata] | |
| MSTRG.9119 | GA29066 | DNA polymerase accessory subunit [Bombyx mori] | |
| MSTRG.9174 | mRpS7 | PREDICTED: 28S ribosomal protein S7, mitochondrial [Bombyx mori] | |
| MSTRG.9193 | -- | PREDICTED: LOW QUALITY PROTEIN: 26S proteasome non-ATPase regulatory subunit 10-like [Bombyx mori] | |
| MSTRG.9215 | usp106 | splicing factor pTSR1 [Pararge aegeria] | |
| MSTRG.9232 | -- | Pastrel [Operophtera brumata] | |
| MSTRG.9233 | -- | Pastrel [Operophtera brumata] | |
| MSTRG.9235 | Fut7 | PREDICTED: alpha-(1,3)-fucosyltransferase 7-like [Bombyx mori] | |
| MSTRG.9237 | -- | PREDICTED: uncharacterized protein LOC101736340 [Bombyx mori] | |
| MSTRG.9264 | BAIAP2 | Brain-specific angiogenesis inhibitor 1-associated protein 2 [Papilio xuthus] | |
| MSTRG.9312 | TY3B-G | Transposon Ty3-G Gag-Pol polyprotein [Papilio machaon] | |
| MSTRG.9339 | -- | reverse transcriptase, partial [Bombyx mori] | |
| MSTRG.9340 | -- | AGAP000245-PA [Anopheles gambiae str. PEST] [Anopheles gambiae] | |
| MSTRG.9389 | -- | PREDICTED: protein snakeskin-like [Anoplophora glabripennis] | |
| MSTRG.9390 | pol | Transposon Ty3-G Gag-Pol polyprotein [Exaiptasia pallida] |
| MSTRG.9409 | FucTC | PREDICTED: alpha-(1,3)-fucosyltransferase C-like [Papilio machaon] |
| MSTRG.9445 | HS3ST3B1 | PREDICTED: heparan sulfate glucosamine 3-O-sulfotransferase 3B1 isoform X2 [Bombyx mori] |
| MSTRG.9478 | -- | olfactory receptor [Bombyx mori] |
| MSTRG.9536 | -- | BRCA1-associated ATM activator 1, partial [Pararge aegeria] |
| MSTRG.9560 | yellow-f2 | PREDICTED: major royal jelly protein 1 [Bombyx mori] |
| MSTRG.9562 | -- | PREDICTED: major royal jelly protein 1-like isoform X1 [Bombyx mori] |
| MSTRG.9569 | yellow-f2 | major royal jelly protein 1 precursor [Bombyx mori] |
| MSTRG.9574 | -- | olfactory receptor, partial [Bombyx mori] |
| MSTRG.964 | -- | PREDICTED: uncharacterized protein LOC106129409 [Amyelois transitella] |
| MSTRG.9672 | SLC26A11 | PREDICTED: sodium-independent sulfate anion transporter-like [Bombyx mori] |
| MSTRG.9689 | SETMAR | endonuclease and reverse transcriptase-like protein [Bombyx mori] |
| MSTRG.9705 | chn | PREDICTED: protein charlatan [Amyelois transitella] |
| MSTRG.9715 | XRCC1 | PREDICTED: DNA repair protein XRCC1 isoform X2 [Bombyx mori] |
| MSTRG.9795 | -- | PREDICTED: protein Smaug [Bombyx mori] |
| MSTRG.9796 | -- | PREDICTED: xanthine dehydrogenase-like [Bombyx mori] |
| MSTRG.9797 | hxnS | PREDICTED: xanthine dehydrogenase-like [Bombyx mori] |
| MSTRG.9799 | AOX2 | PREDICTED: xanthine dehydrogenase-like [Bombyx mori] |
| MSTRG.9809 | -- | PREDICTED: uncharacterized protein LOC103307937 [Acyrthosiphon pisum] |
| MSTRG.9816 | ANAPC1 | PREDICTED: anaphase-promoting complex subunit 1-like [Bombyx mori] |
| MSTRG.9818 | Anapc1 | PREDICTED: anaphase-promoting complex subunit 1-like [Bombyx mori] |
| MSTRG.983 | Uhrf1 | PREDICTED: E3 ubiquitin-protein ligase UHRF1-like isoform X1 [Bombyx mori] |
| MSTRG.991 | Eip78C | PREDICTED: ecdysone-induced protein 78C [Bombyx mori] |
| MSTRG.9957 | GIP | Retrovirus-related Pol polyprotein from transposon TNT 1-94 [Papilio machaon] |
| MSTRG.9958 | -- | PREDICTED: zinc finger protein OZF-like [Bombyx mori] |
| MSTRG.9990 | -- | olfactory receptor [Bombyx mori] |

| **Table S10 The lncRNA-mRNA pairs including 99 silkworm lncRNAs and 105 mRNAs were predicted in the microsporidia congenitally infected silkworm embryos and larvae** | | | | | |
| --- | --- | --- | --- | --- | --- |
| lncRNA_ID | strand | GeneID | | strand | up/down_Stream |
| MSTRG.1723.1 | - | BMSK0005644 | | + | DOWNSTREAM |
| MSTRG.1723.1 | - | BMSK0005645 | | + | DOWNSTREAM |
| MSTRG.2052.1 | - | MSTRG.2048 | | - | UPSTREAM |
| MSTRG.2168.3 | + | BMSK0005902 | | + | UPSTREAM |
| MSTRG.2308.1 | + | BMSK0005973 | | + | DOWNSTREAM |
| MSTRG.2913.1 | + | BMSK0006344 | | + | DOWNSTREAM |
| MSTRG.2991.1 | + | BMSK0006391 | | - | DOWNSTREAM |
| MSTRG.3036.6 | - | BMSK0006418 | | - | UPSTREAM |
| MSTRG.3036.8 | - | BMSK0006418 | | - | UPSTREAM |
| MSTRG.3192.1 | + | BMSK0006514 | | - | DOWNSTREAM |
| MSTRG.3200.3 | + | BMSK0006520 | | - | DOWNSTREAM |
| MSTRG.3197.4 | + | BMSK0006518 | | - | UPSTREAM |
| MSTRG.3197.4 | + | BMSK0006519 | | - | UPSTREAM |
| MSTRG.3204.2 | - | BMSK0006521 | | + | DOWNSTREAM |
| MSTRG.3204.2 | - | BMSK0006524 | | - | DOWNSTREAM |
| MSTRG.3214.1 | - | BMSK0006531 | | - | DOWNSTREAM |
| MSTRG.3214.1 | - | BMSK0006532 | | + | UPSTREAM |
| MSTRG.3220.1 | - | BMSK0006538 | | + | UPSTREAM |
| MSTRG.3211.6 | + | BMSK0006536 | | + | DOWNSTREAM |
| MSTRG.3211.6 | + | BMSK0006538 | | + | UPSTREAM |
| MSTRG.3223.1 | - | BMSK0006536 | | + | DOWNSTREAM |
| MSTRG.3223.1 | - | BMSK0006540 | | - | DOWNSTREAM |
| MSTRG.3358.1 | + | BMSK0006617 | | + | UPSTREAM |
| MSTRG.3712.1 | - | BMSK0006826 | | - | DOWNSTREAM |
| MSTRG.4655.1 | + | BMSK0007359 | | - | DOWNSTREAM |
| MSTRG.5251.1 | - | BMSK0007700 | | + | DOWNSTREAM |
| MSTRG.5977.1 | + | BMSK0008122 | | + | DOWNSTREAM |
| MSTRG.6110.1 | + | BMSK0008197 | | - | UPSTREAM |
| MSTRG.6626.1 | + | BMSK0008493 | | - | UPSTREAM |
| MSTRG.7053.1 | + | BMSK0008725 | | - | DOWNSTREAM |
| MSTRG.7747.1 | - | BMSK0009140 | | + | UPSTREAM |
| MSTRG.7921.1 | - | BMSK0009261 | | - | UPSTREAM |
| MSTRG.7981.1 | - | BMSK0009306 | | + | DOWNSTREAM |
| MSTRG.8021.1 | - | MSTRG.8020 | | - | UPSTREAM |
| MSTRG.8997.1 | - | BMSK0009985 | | - | UPSTREAM |
| MSTRG.9717.2 | - | BMSK0010405 | | + | UPSTREAM |
| MSTRG.9719.3 | - | BMSK0010405 | | + | DOWNSTREAM |
| MSTRG.9719.3 | - | BMSK0010410 | | + | UPSTREAM |
| MSTRG.9724.1 | - | BMSK0010410 | | + | DOWNSTREAM |
| MSTRG.9730.1 | + | BMSK0010414 | | - | DOWNSTREAM |
| MSTRG.9798.1 | - | MSTRG.9796 | | + | DOWNSTREAM |
| MSTRG.9798.1 | - | MSTRG.9799 | | + | UPSTREAM |
| MSTRG.10040.1 | - | BMSK0010574 | | + | DOWNSTREAM |
| MSTRG.10134.1 | + | MSTRG.10131 | | + | DOWNSTREAM |
| MSTRG.10208.1 | + | BMSK0010652 | | - | UPSTREAM |
| MSTRG.10255.2 | + | BMSK0010672 | | + | DOWNSTREAM |
| MSTRG.11149.2 | + | BMSK0011164 | | - | UPSTREAM |
| MSTRG.11281.1 | - | MSTRG.11283 | | + | UPSTREAM |
| MSTRG.11281.1 | - | MSTRG.11284 | | - | DOWNSTREAM |
| MSTRG.11281.1 | - | BMSK0011237 | | + | DOWNSTREAM |
| MSTRG.11306.1 | + | BMSK0011254 | | + | UPSTREAM |
| MSTRG.11306.2 | + | BMSK0011254 | | + | UPSTREAM |
| MSTRG.11308.1 | - | BMSK0011253 | | - | UPSTREAM |
| MSTRG.11729.1 | + | BMSK0000798 | | + | UPSTREAM |
| MSTRG.12045.1 | + | BMSK0000977 | | - | DOWNSTREAM |
| MSTRG.12045.1 | + | BMSK0000978 | | + | UPSTREAM |
| MSTRG.12045.1 | + | BMSK0000976 | | - | DOWNSTREAM |
| MSTRG.12111.1 | + | BMSK0001015 | | + | UPSTREAM |
| MSTRG.12307.1 | + | BMSK0011452 | | - | UPSTREAM |
| MSTRG.12458.1 | + | BMSK0011543 | | + | DOWNSTREAM |
| MSTRG.12465.1 | + | BMSK0011545 | | + | DOWNSTREAM |
| MSTRG.13167.1 | + | BMSK0011965 | | + | DOWNSTREAM |
| MSTRG.13339.1 | + | BMSK0012063 | | - | UPSTREAM |
| MSTRG.13339.1 | + | BMSK0012064 | | - | UPSTREAM |
| MSTRG.13339.1 | + | BMSK0012067 | | + | UPSTREAM |
| MSTRG.13376.1 | + | MSTRG.13378 | | + | UPSTREAM |
| MSTRG.13379.2 | - | BMSK0012088 | | - | DOWNSTREAM |
| MSTRG.13558.7 | - | BMSK0012199 | | - | UPSTREAM |
| MSTRG.13558.8 | - | BMSK0012199 | | - | UPSTREAM |
| MSTRG.14051.3 | + | BMSK0012507 | | - | UPSTREAM |
| MSTRG.14193.2 | + | BMSK0012603 | | + | DOWNSTREAM |
| MSTRG.14387.2 | - | BMSK0012727 | | + | DOWNSTREAM |
| MSTRG.14557.1 | - | MSTRG.14558 | | + | UPSTREAM |
| MSTRG.14874.2 | - | BMSK0012998 | | - | UPSTREAM |
| MSTRG.14885.1 | - | BMSK0013004 | | - | UPSTREAM |
| MSTRG.14897.1 | + | BMSK0013010 | | + | UPSTREAM |
| MSTRG.15336.1 | - | BMSK0013244 | | - | UPSTREAM |
| MSTRG.15337.1 | + | BMSK0013244 | | - | UPSTREAM |
| MSTRG.15338.1 | + | BMSK0013244 | | - | UPSTREAM |
| MSTRG.15479.1 | - | BMSK0013336 | | + | UPSTREAM |
| MSTRG.15729.1 | - | BMSK0013503 | | + | UPSTREAM |
| MSTRG.15849.1 | - | BMSK0013571 | | - | UPSTREAM |
| MSTRG.15849.1 | - | BMSK0013572 | | - | UPSTREAM |
| MSTRG.16090.1 | + | BMSK0013727 | | + | UPSTREAM |
| MSTRG.16090.2 | + | BMSK0013727 | | + | UPSTREAM |
| MSTRG.16480.1 | - | BMSK0013963 | | - | DOWNSTREAM |
| MSTRG.16480.1 | - | BMSK0013964 | | - | DOWNSTREAM |
| MSTRG.17255.1 | - | BMSK0014420 | | - | DOWNSTREAM |
| MSTRG.17259.1 | - | BMSK0014420 | | - | DOWNSTREAM |
| MSTRG.17462.1 | - | BMSK0014556 | | - | DOWNSTREAM |
| MSTRG.17890.6 | + | BMSK0014801 | | + | UPSTREAM |
| MSTRG.18753.1 | + | BMSK0015303 | | + | DOWNSTREAM |
| MSTRG.18753.1 | + | MSTRG.18757 | | + | UPSTREAM |
| MSTRG.18915.2 | - | BMSK0015395 | | - | UPSTREAM |
| MSTRG.18915.2 | - | BMSK0015404 | | + | UPSTREAM |
| MSTRG.18915.2 | - | BMSK0015405 | | - | DOWNSTREAM |
| MSTRG.18915.2 | - | BMSK0015392 | | - | UPSTREAM |
| MSTRG.18915.2 | - | BMSK0015393 | | - | UPSTREAM |
| MSTRG.18915.1 | - | BMSK0015395 | | - | UPSTREAM |
| MSTRG.18915.1 | - | BMSK0015405 | | - | DOWNSTREAM |
| MSTRG.18915.1 | - | BMSK0015392 | | - | UPSTREAM |
| MSTRG.18915.1 | - | BMSK0015393 | | - | UPSTREAM |
| MSTRG.18915.3 | - | BMSK0015405 | | - | DOWNSTREAM |
| MSTRG.18927.1 | - | BMSK0015405 | | - | UPSTREAM |
| MSTRG.18927.1 | - | BMSK0015407 | | + | DOWNSTREAM |
| MSTRG.19120.1 | + | BMSK0015522 | | - | UPSTREAM |
| MSTRG.19268.1 | + | BMSK0015594 | | - | UPSTREAM |
| MSTRG.19268.1 | + | BMSK0015595 | | + | UPSTREAM |
| MSTRG.19407.1 | + | BMSK0015667 | | + | UPSTREAM |
| MSTRG.19407.1 | + | MSTRG.19412 | | - | DOWNSTREAM |
| MSTRG.19414.1 | - | BMSK0015667 | | + | DOWNSTREAM |
| MSTRG.19414.1 | - | MSTRG.19412 | | - | UPSTREAM |
| MSTRG.19414.5 | - | BMSK0015667 | | + | DOWNSTREAM |
| MSTRG.19414.5 | - | MSTRG.19412 | | - | UPSTREAM |
| MSTRG.20027.1 | + | BMSK0016007 | | - | UPSTREAM |
| MSTRG.20303.2 | + | BMSK0001209 | | - | UPSTREAM |
| MSTRG.20535.1 | + | MSTRG.20534 | | + | DOWNSTREAM |
| MSTRG.21115.1 | + | BMSK0001674 | | + | UPSTREAM |
| MSTRG.21463.1 | - | BMSK0001903 | | - | DOWNSTREAM |
| MSTRG.21777.4 | - | BMSK0002099 | | + | UPSTREAM |
| MSTRG.23774.1 | + | BMSK0003353 | | - | UPSTREAM |
| MSTRG.24300.4 | - | BMSK0003664 | | + | UPSTREAM |
| MSTRG.25484.1 | + | BMSK0004343 | | + | UPSTREAM |
| MSTRG.25565.2 | - | MSTRG.25569 | | + | UPSTREAM |
| MSTRG.25922.1 | + | BMSK0004628 | + | | DOWNSTREAM |
| MSTRG.25923.1 | - | BMSK0004628 | + | | DOWNSTREAM |
| MSTRG.26579.1 | - | MSTRG.26581 | + | | UPSTREAM |
| MSTRG.26583.1 | - | MSTRG.26581 | + | | DOWNSTREAM |

| **Table S11 LncRNA-miRNA-mRNA networks of silkworm in the 5th day of *N. bombycis* congenitally infected silkworm embryos** | | | |
| --- | --- | --- | --- |
| ncRNA_ID | mRNA_ID | corr | miRNA_ID(both) |
| MSTRG.10034.1 | BMSK0000230 | 0.918480786 | novel-m0395-5p|novel-m0450-5p|novel-m0950-5p|novel-m1175-5p|novel-m1371-5p|novel-m1482-5p|novel-m1668-5p |
| MSTRG.10034.1 | BMSK0000273 | 0.963016123 | novel-m0395-5p|novel-m0450-5p|novel-m0950-5p|novel-m1175-5p|novel-m1371-5p|novel-m1482-5p|novel-m1668-5p |
| MSTRG.10034.1 | BMSK0001285 | 0.97142398 | novel-m0395-5p|novel-m0450-5p|novel-m0950-5p|novel-m1175-5p|novel-m1371-5p|novel-m1482-5p|novel-m1668-5p |
| MSTRG.10034.1 | BMSK0001933 | 0.945103457 | novel-m0395-5p|novel-m0450-5p|novel-m0950-5p|novel-m1175-5p|novel-m1371-5p|novel-m1482-5p|novel-m1668-5p |
| MSTRG.10034.1 | BMSK0002030 | 0.981958462 | novel-m0395-5p|novel-m0450-5p|novel-m0950-5p|novel-m1175-5p|novel-m1371-5p|novel-m1482-5p|novel-m1668-5p |
| MSTRG.10034.1 | BMSK0002866 | 0.915122274 | novel-m0395-5p|novel-m0450-5p|novel-m0950-5p|novel-m1175-5p|novel-m1371-5p|novel-m1482-5p|novel-m1668-5p |
| MSTRG.10034.1 | BMSK0003353 | 0.918774076 | novel-m0395-5p|novel-m0450-5p|novel-m0950-5p|novel-m1175-5p|novel-m1371-5p|novel-m1482-5p|novel-m1668-5p |
| MSTRG.10034.1 | BMSK0006528 | 0.989674641 | novel-m0395-5p|novel-m0450-5p|novel-m0950-5p|novel-m1175-5p|novel-m1371-5p|novel-m1482-5p|novel-m1668-5p |
| MSTRG.10034.1 | BMSK0007637 | 0.91141127 | novel-m0395-5p|novel-m0450-5p|novel-m0950-5p|novel-m1175-5p|novel-m1371-5p|novel-m1482-5p|novel-m1668-5p |
| MSTRG.10034.1 | BMSK0009085 | 0.911110791 | novel-m0395-5p|novel-m0450-5p|novel-m0950-5p|novel-m1175-5p|novel-m1371-5p|novel-m1482-5p|novel-m1668-5p |
| MSTRG.10034.1 | BMSK0013340 | 0.936598734 | novel-m0395-5p|novel-m0450-5p|novel-m0950-5p|novel-m1175-5p|novel-m1371-5p|novel-m1482-5p|novel-m1668-5p |
| MSTRG.10034.1 | BMSK0013484 | 0.95837904 | novel-m0395-5p|novel-m0450-5p|novel-m0950-5p|novel-m1175-5p|novel-m1371-5p|novel-m1482-5p|novel-m1668-5p |
| MSTRG.10255.2 | BMSK0002454 | 0.904951447 | novel-m1184-3p|novel-m1433-3p|novel-m1467-3p |
| MSTRG.10255.2 | BMSK0010293 | 0.96203568 | novel-m0490-3p|novel-m0712-3p|novel-m1323-3p|novel-m1433-3p|novel-m1467-3p |
| MSTRG.10457.1 | BMSK0000510 | 0.918774324 | novel-m0040-5p|novel-m0284-5p|novel-m0406-5p|novel-m0848-5p|novel-m0917-5p|novel-m1201-5p |
| MSTRG.10457.1 | BMSK0000887 | 0.937073317 | novel-m0040-5p|novel-m0284-5p|novel-m0406-5p|novel-m0848-5p|novel-m0917-5p|novel-m1201-5p |
| MSTRG.10457.1 | BMSK0000947 | 0.971900429 | novel-m0040-5p|novel-m0284-5p|novel-m0406-5p|novel-m0848-5p|novel-m0917-5p|novel-m1201-5p |
| MSTRG.10457.1 | BMSK0004077 | 0.985160876 | novel-m0040-5p|novel-m0284-5p|novel-m0406-5p|novel-m0848-5p|novel-m0917-5p|novel-m1201-5p |
| MSTRG.10457.1 | BMSK0005228 | 0.984069762 | novel-m0040-5p|novel-m0284-5p|novel-m0406-5p|novel-m0848-5p|novel-m0917-5p|novel-m1201-5p |
| MSTRG.10457.1 | BMSK0006077 | 0.939172007 | novel-m0040-5p|novel-m0284-5p|novel-m0406-5p|novel-m0848-5p|novel-m0917-5p|novel-m1201-5p |
| MSTRG.10457.1 | BMSK0006349 | 0.912242994 | novel-m0040-5p|novel-m0284-5p|novel-m0406-5p|novel-m0848-5p|novel-m0917-5p|novel-m1201-5p |
| MSTRG.10457.1 | BMSK0013484 | 0.992899587 | novel-m0040-5p|novel-m0284-5p|novel-m0406-5p|novel-m0848-5p|novel-m0917-5p|novel-m1201-5p |
| MSTRG.10457.1 | BMSK0013868 | 0.911725486 | novel-m0040-5p|novel-m0284-5p|novel-m0406-5p|novel-m0848-5p|novel-m0917-5p|novel-m1201-5p |
| MSTRG.10907.2 | BMSK0005228 | 0.94671481 | novel-m1020-3p|novel-m1211-3p |
| MSTRG.10907.2 | BMSK0006077 | 0.929907658 | novel-m0737-5p|novel-m1020-3p|novel-m1211-3p|novel-m1227-5p |
| MSTRG.10907.2 | BMSK0006528 | 0.906295508 | novel-m0737-5p|novel-m1227-5p |
| MSTRG.10907.2 | BMSK0006537 | 0.985254275 | miR-152-y|novel-m0737-5p|novel-m1227-5p |
| MSTRG.10907.2 | BMSK0009085 | 0.929350401 | novel-m0737-5p|novel-m1227-5p |
| MSTRG.11553.1 | BMSK0000366 | 0.968919586 | miR-411-x |
| MSTRG.11553.1 | BMSK0009032 | 0.967754946 | miR-10-x|miR-6980-x |
| MSTRG.11553.1 | BMSK0013098 | 0.937998386 | miR-411-x|miR-6980-x |
| MSTRG.11917.1 | BMSK0000230 | 0.930168414 | novel-m0395-5p|novel-m0450-5p|novel-m0950-5p|novel-m1175-5p|novel-m1371-5p|novel-m1482-5p|novel-m1668-5p |
| MSTRG.11917.1 | BMSK0000273 | 0.990805362 | novel-m0395-5p|novel-m0450-5p|novel-m0950-5p|novel-m1175-5p|novel-m1371-5p|novel-m1482-5p|novel-m1668-5p |
| MSTRG.11917.1 | BMSK0001086 | 0.955518024 | novel-m0395-5p|novel-m0450-5p|novel-m0950-5p|novel-m1175-5p|novel-m1371-5p|novel-m1482-5p|novel-m1668-5p |
| MSTRG.11917.1 | BMSK0001285 | 0.993945366 | novel-m0395-5p|novel-m0450-5p|novel-m0950-5p|novel-m1175-5p|novel-m1371-5p|novel-m1482-5p|novel-m1668-5p |
| MSTRG.11917.1 | BMSK0001933 | 0.991352858 | novel-m0395-5p|novel-m0450-5p|novel-m0950-5p|novel-m1175-5p|novel-m1371-5p|novel-m1482-5p|novel-m1668-5p |
| MSTRG.11917.1 | BMSK0002030 | 0.942526183 | miR-8517-x|novel-m0395-5p|novel-m0450-5p|novel-m0950-5p|novel-m1175-5p|novel-m1371-5p|novel-m1482-5p|novel-m1668-5p |
| MSTRG.11917.1 | BMSK0002736 | 0.914362179 | novel-m0395-5p|novel-m0450-5p|novel-m0950-5p|novel-m1175-5p|novel-m1371-5p|novel-m1482-5p|novel-m1668-5p |
| MSTRG.11917.1 | BMSK0002866 | 0.955863228 | novel-m0395-5p|novel-m0450-5p|novel-m0950-5p|novel-m1175-5p|novel-m1371-5p|novel-m1482-5p|novel-m1668-5p |
| MSTRG.11917.1 | BMSK0004663 | 0.947396305 | novel-m0395-5p|novel-m0450-5p|novel-m0950-5p|novel-m1175-5p|novel-m1371-5p|novel-m1482-5p|novel-m1668-5p |
| MSTRG.11917.1 | BMSK0005258 | 0.988427404 | miR-8517-x|novel-m0786-3p|novel-m1442-3p|novel-m1679-3p |
| MSTRG.11917.1 | BMSK0005697 | 0.918362962 | novel-m0395-5p|novel-m0450-5p|novel-m0950-5p|novel-m1175-5p|novel-m1371-5p|novel-m1482-5p|novel-m1668-5p |
| MSTRG.11917.1 | BMSK0005711 | 0.942975402 | novel-m0395-5p|novel-m0450-5p|novel-m0950-5p|novel-m1175-5p|novel-m1371-5p|novel-m1482-5p|novel-m1668-5p |
| MSTRG.11917.1 | BMSK0006301 | 0.967383876 | novel-m0786-3p|novel-m1442-3p|novel-m1679-3p |
| MSTRG.11917.1 | BMSK0006528 | 0.944386207 | miR-8517-x|novel-m0395-5p|novel-m0450-5p|novel-m0737-5p|novel-m0950-5p|novel-m1175-5p|novel-m1227-5p|novel-m1371-5p|novel-m1482-5p|novel-m1668-5p |
| MSTRG.11917.1 | BMSK0006537 | 0.940217865 | miR-8517-x|novel-m0307-3p|novel-m0737-5p|novel-m1227-5p |
| MSTRG.11917.1 | BMSK0006982 | 0.913158381 | novel-m0395-5p|novel-m0450-5p|novel-m0950-5p|novel-m1175-5p|novel-m1371-5p|novel-m1482-5p|novel-m1668-5p |
| MSTRG.11917.1 | BMSK0007637 | 0.970813379 | novel-m0395-5p|novel-m0450-5p|novel-m0950-5p|novel-m1175-5p|novel-m1371-5p|novel-m1482-5p|novel-m1668-5p |
| MSTRG.11917.1 | BMSK0008518 | 0.938057881 | novel-m0395-5p|novel-m0450-5p|novel-m0950-5p|novel-m1175-5p|novel-m1371-5p|novel-m1482-5p|novel-m1668-5p |
| MSTRG.11917.1 | BMSK0009085 | 0.990243843 | novel-m0395-5p|novel-m0450-5p|novel-m0737-5p|novel-m0950-5p|novel-m1175-5p|novel-m1227-5p|novel-m1371-5p|novel-m1482-5p|novel-m1668-5p |
| MSTRG.11917.1 | BMSK0012494 | 0.948738701 | novel-m0737-5p|novel-m0786-3p|novel-m1227-5p|novel-m1442-3p|novel-m1679-3p |
| MSTRG.11917.1 | BMSK0013340 | 0.99536586 | novel-m0395-5p|novel-m0450-5p|novel-m0786-3p|novel-m0950-5p|novel-m1175-5p|novel-m1371-5p|novel-m1442-3p|novel-m1482-5p|novel-m1668-5p|novel-m1679-3p |
| MSTRG.11917.1 | BMSK0013484 | 0.986809238 | miR-8517-x|novel-m0395-5p|novel-m0450-5p|novel-m0786-3p|novel-m0950-5p|novel-m1175-5p|novel-m1371-5p|novel-m1442-3p|novel-m1482-5p|novel-m1668-5p|novel-m1679-3p |
| MSTRG.12449.1 | BMSK0002454 | 0.928575467 | novel-m1433-3p|novel-m1467-3p |
| MSTRG.12449.1 | BMSK0005879 | 0.927838141 | novel-m0401-3p |
| MSTRG.12449.1 | BMSK0010293 | 0.953630525 | miR-3267-y|novel-m0401-3p|novel-m1433-3p|novel-m1467-3p |
| MSTRG.12449.1 | BMSK0010795 | 0.974945786 | miR-3267-y|novel-m0401-3p |
| MSTRG.12449.1 | BMSK0012465 | 0.961143273 | miR-3267-y|novel-m0401-3p |
| MSTRG.12449.1 | BMSK0014140 | 0.972832835 | miR-3267-y |
| MSTRG.12565.1 | BMSK0000504 | 0.909656778 | bmo-miR-3362 |
| MSTRG.12565.1 | BMSK0003227 | 0.922328991 | bmo-miR-3362 |
| MSTRG.12565.1 | BMSK0013340 | 0.921802885 | bmo-miR-2770-5p|novel-m1539-5p |
| MSTRG.13167.1 | BMSK0004077 | 0.939334221 | novel-m0040-5p|novel-m0284-5p|novel-m0406-5p|novel-m0848-5p|novel-m0917-5p|novel-m1201-5p |
| MSTRG.13167.1 | BMSK0005228 | 0.914409013 | novel-m0040-5p|novel-m0284-5p|novel-m0406-5p|novel-m0848-5p|novel-m0917-5p|novel-m1201-5p |
| MSTRG.13167.1 | BMSK0006077 | 0.927848145 | novel-m0040-5p|novel-m0284-5p|novel-m0406-5p|novel-m0848-5p|novel-m0917-5p|novel-m1201-5p |
| MSTRG.13167.1 | BMSK0013098 | 0.981997925 | bmo-miR-2760-5p|bmo-miR-3400 |
| MSTRG.13167.1 | BMSK0013484 | 0.91242412 | novel-m0040-5p|novel-m0284-5p|novel-m0406-5p|novel-m0848-5p|novel-m0917-5p|novel-m1201-5p |
| MSTRG.13963.4 | BMSK0000066 | 0.936546876 | novel-m1345-3p |
| MSTRG.13963.4 | BMSK0001934 | 0.980238344 | miR-7-x |
| MSTRG.13963.4 | BMSK0004519 | 0.952338788 | miR-7-x |
| MSTRG.13963.4 | BMSK0006523 | 0.919690791 | miR-7-x |
| MSTRG.14415.1 | BMSK0001934 | 0.987542349 | miR-7-x |
| MSTRG.14415.1 | BMSK0004519 | 0.979935632 | miR-7-x |
| MSTRG.14415.1 | BMSK0006523 | 0.971803912 | miR-7-x |
| MSTRG.14415.1 | BMSK0009032 | 0.997719622 | miR-10-x |
| MSTRG.14868.1 | BMSK0001975 | 0.987691207 | novel-m1143-5p|novel-m1158-5p |
| MSTRG.14868.1 | BMSK0010080 | 0.933499134 | novel-m1143-5p|novel-m1158-5p|novel-m1276-3p |
| MSTRG.14868.1 | BMSK0010217 | 0.970972266 | novel-m1143-5p|novel-m1158-5p |
| MSTRG.14868.1 | BMSK0015533 | 0.911540356 | novel-m1143-5p|novel-m1158-5p |
| MSTRG.14869.1 | BMSK0013662 | 0.925262755 | miR-2805-y |
| MSTRG.15812.1 | BMSK0007541 | 0.952349385 | bmo-miR-124 |
| MSTRG.15812.1 | BMSK0007700 | 0.97724175 | bmo-miR-2754|novel-m1276-5p |
| MSTRG.17900.1 | BMSK0012199 | 0.955340217 | miR-216-x |
| MSTRG.18153.1 | BMSK0002736 | 0.962285861 | novel-m0824-5p|novel-m1096-5p|novel-m1438-5p |
| MSTRG.18449.1 | BMSK0000055 | 0.911660499 | novel-m0375-3p |
| MSTRG.18449.1 | BMSK0000230 | 0.990898342 | bmo-miR-3347a |
| MSTRG.18449.1 | BMSK0002310 | 0.977291448 | bmo-miR-3347a |
| MSTRG.18449.1 | BMSK0002320 | 0.962607888 | novel-m0375-3p |
| MSTRG.18449.1 | BMSK0002736 | 0.938507657 | bmo-miR-3347a|novel-m0375-3p |
| MSTRG.18449.1 | BMSK0006111 | 0.913083511 | novel-m0375-3p |
| MSTRG.18449.1 | BMSK0007150 | 0.94327921 | bmo-miR-3347a |
| MSTRG.18449.1 | BMSK0013080 | 0.943109909 | novel-m0375-3p |
| MSTRG.18449.1 | BMSK0013768 | 0.970538818 | bmo-miR-3347a |
| MSTRG.19569.1 | BMSK0002270 | 0.964221998 | novel-m0304-3p |
| MSTRG.19569.1 | BMSK0006537 | 0.941868548 | novel-m1538-3p|novel-m1541-3p |
| MSTRG.19569.1 | BMSK0012455 | 0.964311605 | novel-m0304-3p|novel-m0458-5p |
| MSTRG.205.1 | BMSK0003071 | 0.91858877 | miR-971-y |
| MSTRG.205.1 | BMSK0003930 | 0.970470586 | bmo-miR-2738|novel-m1141-3p |
| MSTRG.205.1 | BMSK0007421 | 0.902180888 | novel-m1141-3p |
| MSTRG.205.1 | BMSK0007700 | 0.937269212 | bmo-miR-2738|novel-m1141-3p |
| MSTRG.205.1 | BMSK0007748 | 0.970151323 | novel-m1141-3p |
| MSTRG.20575.4 | BMSK0001898 | 0.976732462 | miR-2808-y |
| MSTRG.20575.4 | BMSK0002623 | 0.970816163 | bmo-miR-33-5p |
| MSTRG.20575.4 | BMSK0005879 | 0.900393021 | miR-2808-y | | |
| MSTRG.20575.4 | BMSK0015832 | 0.960668056 | bmo-miR-33-5p | | |
| MSTRG.21107.2 | BMSK0000947 | 0.971645667 | bmo-miR-3385-3p | | |
| MSTRG.21107.2 | BMSK0012494 | 0.956433034 | bmo-miR-3385-3p | | |
| MSTRG.2123.2 | BMSK0008518 | 0.96689419 | novel-m0096-5p|novel-m0396-5p|novel-m0608-5p|novel-m0649-5p|novel-m0655-5p|novel-m0885-5p|novel-m0961-5p|novel-m1123-5p|novel-m1295-5p|novel-m1405-5p|novel-m1420-5p|novel-m1469-5p|novel-m1504-5p|novel-m1610-5p | | |
| MSTRG.21463.1 | BMSK0002454 | 0.933713349 | bmo-miR-3350|novel-m1184-3p|novel-m1433-3p|novel-m1467-3p|novel-m1678-3p | | |
| MSTRG.21463.1 | BMSK0005379 | 0.987848258 | miR-217-x | | |
| MSTRG.21463.1 | BMSK0006816 | 0.95958834 | novel-m1678-3p | | |
| MSTRG.21463.1 | BMSK0010080 | 0.924786304 | novel-m0490-3p|novel-m0712-3p|novel-m1323-3p | | |
| MSTRG.21463.1 | BMSK0010293 | 0.965376769 | novel-m0490-3p|novel-m0712-3p|novel-m1323-3p|novel-m1433-3p|novel-m1467-3p | | |
| MSTRG.21463.1 | BMSK0015832 | 0.960937023 | bmo-miR-3350|miR-217-x | | |
| MSTRG.22299.1 | BMSK0004519 | 0.900620047 | miR-7-x|novel-m0338-5p|novel-m1566-5p | | |
| MSTRG.22443.1 | BMSK0001934 | 0.97803017 | miR-7-x | | |
| MSTRG.22443.1 | BMSK0002310 | 0.990860147 | novel-m0743-5p | | |
| MSTRG.22443.1 | BMSK0005228 | 0.965150929 | novel-m1020-3p|novel-m1211-3p | | |
| MSTRG.22443.1 | BMSK0006077 | 0.921498983 | novel-m1020-3p|novel-m1211-3p | | |
| MSTRG.22443.1 | BMSK0006523 | 0.974776352 | miR-7-x | | |
| MSTRG.22498.1 | BMSK0000510 | 0.907365 | bmo-miR-3001|bmo-miR-3362 | | |
| MSTRG.22498.1 | BMSK0001550 | 0.992643436 | bmo-miR-3001 | | |
| MSTRG.22498.1 | BMSK0002270 | 0.918575711 | novel-m0304-3p | | |
| MSTRG.22498.1 | BMSK0002866 | 0.973466177 | bmo-miR-3001|bmo-miR-3362 | | |
| MSTRG.22498.1 | BMSK0003227 | 0.987363589 | bmo-miR-3362 | | |
| MSTRG.22498.1 | BMSK0003694 | 0.934560811 | bmo-miR-3001 | | |
| MSTRG.22498.1 | BMSK0005258 | 0.992017475 | miR-2755-y|miR-998-y | | |
| MSTRG.22498.1 | BMSK0010061 | 0.960094717 | bmo-miR-3384-3p | | |
| MSTRG.22498.1 | BMSK0012319 | 0.986644679 | bmo-miR-3001 | | |
| MSTRG.22894.1 | BMSK0001215 | 0.927305508 | novel-m0348-5p|novel-m0411-5p|novel-m0753-5p|novel-m1039-5p | | |
| MSTRG.23024.2 | BMSK0000230 | 0.903492432 | novel-m0395-5p|novel-m0450-5p|novel-m0950-5p|novel-m1175-5p|novel-m1371-5p|novel-m1482-5p|novel-m1668-5p | | |
| MSTRG.23024.2 | BMSK0000273 | 0.936863846 | novel-m0395-5p|novel-m0450-5p|novel-m0950-5p|novel-m1175-5p|novel-m1371-5p|novel-m1482-5p|novel-m1668-5p | | |
| MSTRG.23024.2 | BMSK0001086 | 0.943635851 | novel-m0395-5p|novel-m0450-5p|novel-m0950-5p|novel-m1175-5p|novel-m1371-5p|novel-m1482-5p|novel-m1668-5p | | |
| MSTRG.23024.2 | BMSK0001285 | 0.941230675 | novel-m0395-5p|novel-m0450-5p|novel-m0950-5p|novel-m1175-5p|novel-m1371-5p|novel-m1482-5p|novel-m1668-5p | | |
| MSTRG.23024.2 | BMSK0001550 | 0.948804664 | bmo-miR-3001 | | |
| MSTRG.23024.2 | BMSK0001933 | 0.96723259 | novel-m0395-5p|novel-m0450-5p|novel-m0950-5p|novel-m1175-5p|novel-m1371-5p|novel-m1482-5p|novel-m1668-5p | | |
| MSTRG.23024.2 | BMSK0002736 | 0.918527844 | novel-m0395-5p|novel-m0450-5p|novel-m0950-5p|novel-m1175-5p|novel-m1371-5p|novel-m1482-5p|novel-m1668-5p | | |
| MSTRG.23024.2 | BMSK0002866 | 0.947089328 | bmo-miR-3001|novel-m0395-5p|novel-m0450-5p|novel-m0950-5p|novel-m1175-5p|novel-m1371-5p|novel-m1482-5p|novel-m1668-5p | | |
| MSTRG.23024.2 | BMSK0004663 | 0.976753958 | novel-m0395-5p|novel-m0450-5p|novel-m0950-5p|novel-m1175-5p|novel-m1371-5p|novel-m1482-5p|novel-m1668-5p | | |
| MSTRG.23024.2 | BMSK0005711 | 0.963959703 | novel-m0395-5p|novel-m0450-5p|novel-m0950-5p|novel-m1175-5p|novel-m1371-5p|novel-m1482-5p|novel-m1668-5p | | |
| MSTRG.23024.2 | BMSK0006982 | 0.965210961 | bmo-miR-3001|novel-m0395-5p|novel-m0450-5p|novel-m0950-5p|novel-m1175-5p|novel-m1371-5p|novel-m1482-5p|novel-m1668-5p | | |
| MSTRG.23024.2 | BMSK0007637 | 0.958886616 | novel-m0395-5p|novel-m0450-5p|novel-m0950-5p|novel-m1175-5p|novel-m1371-5p|novel-m1482-5p|novel-m1668-5p | | |
| MSTRG.23024.2 | BMSK0008518 | 0.945765115 | novel-m0395-5p|novel-m0450-5p|novel-m0950-5p|novel-m1175-5p|novel-m1371-5p|novel-m1482-5p|novel-m1668-5p | | |
| MSTRG.23024.2 | BMSK0009085 | 0.982496216 | novel-m0395-5p|novel-m0450-5p|novel-m0950-5p|novel-m1175-5p|novel-m1371-5p|novel-m1482-5p|novel-m1668-5p | | |
| MSTRG.23024.2 | BMSK0013340 | 0.96971752 | novel-m0395-5p|novel-m0450-5p|novel-m0950-5p|novel-m1175-5p|novel-m1371-5p|novel-m1482-5p|novel-m1668-5p | | |
| MSTRG.23024.2 | BMSK0013484 | 0.93846226 | novel-m0395-5p|novel-m0450-5p|novel-m0950-5p|novel-m1175-5p|novel-m1371-5p|novel-m1482-5p|novel-m1668-5p | | |
| MSTRG.23024.2 | BMSK0013868 | 0.910349148 | novel-m0395-5p|novel-m0450-5p|novel-m0950-5p|novel-m1175-5p|novel-m1371-5p|novel-m1482-5p|novel-m1668-5p | | |
| MSTRG.23025.1 | BMSK0000230 | 0.921753715 | novel-m0395-5p|novel-m0450-5p|novel-m0950-5p|novel-m1175-5p|novel-m1371-5p|novel-m1482-5p|novel-m1668-5p | | |
| MSTRG.23025.1 | BMSK0000273 | 0.993811283 | novel-m0395-5p|novel-m0450-5p|novel-m0950-5p|novel-m1175-5p|novel-m1371-5p|novel-m1482-5p|novel-m1668-5p | | |
| MSTRG.23025.1 | BMSK0000504 | 0.97321962 | novel-m0824-5p|novel-m1096-5p|novel-m1438-5p | | |
| MSTRG.23025.1 | BMSK0001086 | 0.957340279 | novel-m0395-5p|novel-m0450-5p|novel-m0950-5p|novel-m1175-5p|novel-m1371-5p|novel-m1482-5p|novel-m1668-5p | | |
| MSTRG.23025.1 | BMSK0001285 | 0.99287057 | novel-m0395-5p|novel-m0450-5p|novel-m0950-5p|novel-m1175-5p|novel-m1371-5p|novel-m1482-5p|novel-m1668-5p | | |
| MSTRG.23025.1 | BMSK0001933 | 0.987685891 | novel-m0395-5p|novel-m0450-5p|novel-m0950-5p|novel-m1175-5p|novel-m1371-5p|novel-m1482-5p|novel-m1668-5p | | |
| MSTRG.23025.1 | BMSK0002030 | 0.956596347 | novel-m0395-5p|novel-m0450-5p|novel-m0950-5p|novel-m1175-5p|novel-m1371-5p|novel-m1482-5p|novel-m1668-5p | | |
| MSTRG.23025.1 | BMSK0002736 | 0.90724905 | novel-m0395-5p|novel-m0450-5p|novel-m0824-5p|novel-m0950-5p|novel-m1096-5p|novel-m1175-5p|novel-m1371-5p|novel-m1438-5p|novel-m1482-5p|novel-m1668-5p | | |
| MSTRG.23025.1 | BMSK0002866 | 0.945745161 | novel-m0395-5p|novel-m0450-5p|novel-m0950-5p|novel-m1175-5p|novel-m1371-5p|novel-m1482-5p|novel-m1668-5p | | |
| MSTRG.23025.1 | BMSK0004663 | 0.93172524 | novel-m0395-5p|novel-m0450-5p|novel-m0950-5p|novel-m1175-5p|novel-m1371-5p|novel-m1482-5p|novel-m1668-5p | | |
| MSTRG.23025.1 | BMSK0005697 | 0.91250381 | novel-m0395-5p|novel-m0450-5p|novel-m0950-5p|novel-m1175-5p|novel-m1371-5p|novel-m1482-5p|novel-m1668-5p | | |
| MSTRG.23025.1 | BMSK0005711 | 0.929305981 | novel-m0395-5p|novel-m0450-5p|novel-m0950-5p|novel-m1175-5p|novel-m1371-5p|novel-m1482-5p|novel-m1668-5p | | |
| MSTRG.23025.1 | BMSK0006077 | 0.91098884 | novel-m0824-5p|novel-m1096-5p|novel-m1438-5p | | |
| MSTRG.23025.1 | BMSK0006528 | 0.959182044 | novel-m0395-5p|novel-m0450-5p|novel-m0950-5p|novel-m1175-5p|novel-m1371-5p|novel-m1482-5p|novel-m1668-5p | | |
| MSTRG.23025.1 | BMSK0007637 | 0.964273902 | novel-m0395-5p|novel-m0450-5p|novel-m0950-5p|novel-m1175-5p|novel-m1371-5p|novel-m1482-5p|novel-m1668-5p | | |
| MSTRG.23025.1 | BMSK0008518 | 0.927679738 | novel-m0395-5p|novel-m0450-5p|novel-m0950-5p|novel-m1175-5p|novel-m1371-5p|novel-m1482-5p|novel-m1668-5p | | |
| MSTRG.23025.1 | BMSK0009085 | 0.982259234 | bmo-miR-2770-5p|novel-m0395-5p|novel-m0450-5p|novel-m0950-5p|novel-m1175-5p|novel-m1371-5p|novel-m1482-5p|novel-m1668-5p | | |
| MSTRG.23025.1 | BMSK0013340 | 0.989862222 | bmo-miR-2770-5p|novel-m0395-5p|novel-m0450-5p|novel-m0950-5p|novel-m1175-5p|novel-m1371-5p|novel-m1482-5p|novel-m1668-5p | | |
| MSTRG.23025.1 | BMSK0013484 | 0.982807441 | bmo-miR-2770-5p|novel-m0395-5p|novel-m0450-5p|novel-m0950-5p|novel-m1175-5p|novel-m1371-5p|novel-m1482-5p|novel-m1668-5p | |
| MSTRG.23380.1 | BMSK0002899 | 0.983321065 | bmo-miR-2744|novel-m0745-5p|novel-m0779-5p|novel-m1602-3p | |
| MSTRG.24222.1 | BMSK0002809 | 0.935436418 | novel-m0373-5p | |
| MSTRG.24222.1 | BMSK0010795 | 0.923442533 | bmo-miR-745-3p|novel-m0401-3p | |
| MSTRG.25257.4 | BMSK0002899 | 0.979066181 | novel-m0312-5p|novel-m0923-5p | |
| MSTRG.25269.1 | BMSK0001898 | 0.952698153 | miR-3316-y | |
| MSTRG.25269.1 | BMSK0002127 | 0.974685086 | miR-3316-y | |
| MSTRG.25269.1 | BMSK0003930 | 0.946599417 | bmo-miR-2794|miR-3316-y | |
| MSTRG.25269.1 | BMSK0004306 | 0.945507259 | miR-3316-y | |
| MSTRG.25269.1 | BMSK0013662 | 0.934177562 | miR-2805-y | |
| MSTRG.25269.1 | BMSK0015533 | 0.924218677 | bmo-miR-2794 | |
| MSTRG.25484.1 | BMSK0002454 | 0.920257475 | novel-m0745-5p|novel-m0779-5p | |
| MSTRG.25484.1 | BMSK0002899 | 0.965826388 | novel-m0745-5p|novel-m0779-5p | |
| MSTRG.25484.1 | BMSK0014031 | 0.999642402 | novel-m0745-5p|novel-m0779-5p | |
| MSTRG.25565.2 | BMSK0000230 | 0.93534986 | novel-m0395-5p|novel-m0450-5p|novel-m0950-5p|novel-m1175-5p|novel-m1371-5p|novel-m1482-5p|novel-m1668-5p | |
| MSTRG.25565.2 | BMSK0000273 | 0.984311295 | novel-m0395-5p|novel-m0450-5p|novel-m0950-5p|novel-m1175-5p|novel-m1371-5p|novel-m1482-5p|novel-m1668-5p | |
| MSTRG.25565.2 | BMSK0001086 | 0.929530144 | novel-m0395-5p|novel-m0450-5p|novel-m0950-5p|novel-m1175-5p|novel-m1371-5p|novel-m1482-5p|novel-m1668-5p | |
| MSTRG.25565.2 | BMSK0001285 | 0.997442965 | bmo-miR-3362|novel-m0395-5p|novel-m0450-5p|novel-m0950-5p|novel-m1175-5p|novel-m1371-5p|novel-m1482-5p|novel-m1668-5p | |
| MSTRG.25565.2 | BMSK0001933 | 0.983246933 | novel-m0395-5p|novel-m0450-5p|novel-m0950-5p|novel-m1175-5p|novel-m1371-5p|novel-m1482-5p|novel-m1668-5p | |
| MSTRG.25565.2 | BMSK0002030 | 0.944168843 | bmo-miR-3362|miR-8517-x|novel-m0395-5p|novel-m0450-5p|novel-m0950-5p|novel-m1175-5p|novel-m1371-5p|novel-m1482-5p|novel-m1668-5p | |
| MSTRG.25565.2 | BMSK0002531 | 0.928090358 | novel-m0089-5p|novel-m0509-5p|novel-m0573-5p|novel-m0834-5p|novel-m0948-5p|novel-m1045-5p|novel-m1193-5p|novel-m1266-5p|novel-m1320-5p|novel-m1368-5p|novel-m1380-5p|novel-m1397-5p|novel-m1404-5p|novel-m1426-5p|novel-m1514-5p|novel-m1582-5p|novel-m1614-5p|novel-m1633-5p|novel-m1652-5p|novel-m1677-5p | |
| MSTRG.25565.2 | BMSK0002866 | 0.954831188 | bmo-miR-3001|bmo-miR-3362|novel-m0395-5p|novel-m0450-5p|novel-m0950-5p|novel-m1175-5p|novel-m1371-5p|novel-m1482-5p|novel-m1668-5p | |
| MSTRG.25565.2 | BMSK0004663 | 0.939042068 | novel-m0395-5p|novel-m0450-5p|novel-m0950-5p|novel-m1175-5p|novel-m1371-5p|novel-m1482-5p|novel-m1668-5p | |
| MSTRG.25565.2 | BMSK0005697 | 0.928016886 | novel-m0395-5p|novel-m0450-5p|novel-m0950-5p|novel-m1175-5p|novel-m1371-5p|novel-m1482-5p|novel-m1668-5p | |
| MSTRG.25565.2 | BMSK0005711 | 0.940731986 | novel-m0395-5p|novel-m0450-5p|novel-m0950-5p|novel-m1175-5p|novel-m1371-5p|novel-m1482-5p|novel-m1668-5p | |
| MSTRG.25565.2 | BMSK0006528 | 0.946753202 | miR-8517-x|novel-m0395-5p|novel-m0450-5p|novel-m0950-5p|novel-m1175-5p|novel-m1371-5p|novel-m1482-5p|novel-m1668-5p | |
| MSTRG.25565.2 | BMSK0006982 | 0.901740947 | bmo-miR-3001|novel-m0395-5p|novel-m0450-5p|novel-m0950-5p|novel-m1175-5p|novel-m1371-5p|novel-m1482-5p|novel-m1668-5p | |
| MSTRG.25565.2 | BMSK0007637 | 0.963387451 | novel-m0395-5p|novel-m0450-5p|novel-m0950-5p|novel-m1175-5p|novel-m1371-5p|novel-m1482-5p|novel-m1668-5p | |
| MSTRG.25565.2 | BMSK0008518 | 0.927486977 | novel-m0395-5p|novel-m0450-5p|novel-m0950-5p|novel-m1175-5p|novel-m1371-5p|novel-m1482-5p|novel-m1668-5p | |
| MSTRG.25565.2 | BMSK0009085 | 0.985838599 | novel-m0395-5p|novel-m0450-5p|novel-m0950-5p|novel-m1175-5p|novel-m1371-5p|novel-m1482-5p|novel-m1668-5p | |
| MSTRG.25565.2 | BMSK0013340 | 0.99483231 | novel-m0395-5p|novel-m0450-5p|novel-m0950-5p|novel-m1175-5p|novel-m1371-5p|novel-m1482-5p|novel-m1668-5p | |
| MSTRG.25565.2 | BMSK0013484 | 0.995749452 | bmo-miR-3362|miR-8517-x|novel-m0395-5p|novel-m0450-5p|novel-m0950-5p|novel-m1175-5p|novel-m1371-5p|novel-m1482-5p|novel-m1668-5p | |
| MSTRG.26308.1 | BMSK0005258 | 0.992483998 | novel-m0786-3p|novel-m1442-3p|novel-m1679-3p | |
| MSTRG.26308.1 | BMSK0006301 | 0.915518441 | novel-m0786-3p|novel-m1442-3p|novel-m1679-3p | |
| MSTRG.26308.1 | BMSK0012494 | 0.960620318 | novel-m0786-3p|novel-m1442-3p|novel-m1679-3p | |
| MSTRG.26308.1 | BMSK0013340 | 0.980375479 | novel-m0786-3p|novel-m1442-3p|novel-m1679-3p | |
| MSTRG.26308.1 | BMSK0013484 | 0.966001913 | novel-m0786-3p|novel-m1442-3p|novel-m1679-3p | |
| MSTRG.2638.1 | BMSK0006077 | 0.947024674 | novel-m0737-5p|novel-m1227-5p | |
| MSTRG.2638.1 | BMSK0006537 | 0.96390538 | novel-m0737-5p|novel-m1227-5p | |
| MSTRG.2638.1 | BMSK0009085 | 0.95004242 | novel-m0737-5p|novel-m1227-5p | |
| MSTRG.2638.1 | BMSK0012494 | 0.937850059 | bmo-miR-3385-3p|novel-m0737-5p|novel-m1227-5p | |
| MSTRG.3192.1 | BMSK0001550 | 0.959754935 | bmo-miR-3001 | |
| MSTRG.3192.1 | BMSK0002310 | 0.991170943 | novel-m0743-5p | |
| MSTRG.3192.1 | BMSK0003694 | 0.940702718 | bmo-miR-3001 | |
| MSTRG.3192.1 | BMSK0012319 | 0.986007915 | bmo-miR-3001 | |
| MSTRG.3192.1 | BMSK0013098 | 0.956247242 | bmo-miR-3001|novel-m0743-5p | |
| MSTRG.3192.1 | BMSK0015675 | 0.961752551 | bmo-miR-3001 | |
| MSTRG.3211.6 | BMSK0002320 | 0.90915398 | miR-34-x|novel-m0375-3p|novel-m0496-3p | |
| MSTRG.3211.6 | BMSK0002736 | 0.90597909 | novel-m0375-3p|novel-m1435-5p | |
| MSTRG.3211.6 | BMSK0006077 | 0.914596909 | novel-m0737-5p|novel-m1227-5p|novel-m1435-5p | |
| MSTRG.3211.6 | BMSK0006528 | 0.977168079 | miR-34-x|novel-m0737-5p|novel-m1227-5p | |
| MSTRG.3211.6 | BMSK0006537 | 0.941796663 | novel-m0737-5p|novel-m1227-5p | |
| MSTRG.3211.6 | BMSK0007777 | 0.981157392 | novel-m1435-5p | |
| MSTRG.3211.6 | BMSK0009085 | 0.959721986 | novel-m0375-3p|novel-m0737-5p|novel-m1227-5p | |
| MSTRG.3211.6 | BMSK0012494 | 0.903511271 | novel-m0737-5p|novel-m1227-5p | |
| MSTRG.3211.6 | BMSK0012727 | 0.955417457 | miR-34-x | |
| MSTRG.3211.6 | BMSK0013080 | 0.982340463 | novel-m0375-3p|novel-m1435-5p | |
| MSTRG.3712.1 | BMSK0001934 | 0.975842268 | miR-7-x | |
| MSTRG.3712.1 | BMSK0006523 | 0.914942584 | miR-7-x|miR-765-y | |
| MSTRG.3712.1 | BMSK0009032 | 0.926505911 | miR-6980-x |
| MSTRG.3970.1 | BMSK0006528 | 0.967204321 | miR-3224-x|novel-m1067-3p |
| MSTRG.4046.2 | BMSK0002122 | 0.90482919 | miR-183-x |
| MSTRG.4457.1 | BMSK0000066 | 0.96359701 | novel-m0304-3p|novel-m0792-3p |
| MSTRG.4457.1 | BMSK0000510 | 0.924876988 | bmo-miR-2770-5p|novel-m0040-5p|novel-m0284-5p|novel-m0406-5p|novel-m0848-5p|novel-m0917-5p|novel-m1201-5p |
| MSTRG.4457.1 | BMSK0000887 | 0.979302632 | novel-m0040-5p|novel-m0284-5p|novel-m0406-5p|novel-m0848-5p|novel-m0917-5p|novel-m1201-5p |
| MSTRG.4457.1 | BMSK0000947 | 0.979571889 | novel-m0040-5p|novel-m0284-5p|novel-m0406-5p|novel-m0848-5p|novel-m0917-5p|novel-m1201-5p |
| MSTRG.4457.1 | BMSK0004077 | 0.973967616 | novel-m0040-5p|novel-m0284-5p|novel-m0406-5p|novel-m0848-5p|novel-m0917-5p|novel-m1201-5p |
| MSTRG.4457.1 | BMSK0005228 | 0.984312353 | miR-8517-x|novel-m0040-5p|novel-m0284-5p|novel-m0406-5p|novel-m0848-5p|novel-m0917-5p|novel-m1201-5p |
| MSTRG.4457.1 | BMSK0005258 | 0.980386868 | miR-8517-x|novel-m0786-3p|novel-m1442-3p|novel-m1679-3p |
| MSTRG.4457.1 | BMSK0006077 | 0.950039736 | novel-m0040-5p|novel-m0284-5p|novel-m0406-5p|novel-m0848-5p|novel-m0917-5p|novel-m1201-5p |
| MSTRG.4457.1 | BMSK0006301 | 0.942042839 | novel-m0786-3p|novel-m1442-3p|novel-m1679-3p |
| MSTRG.4457.1 | BMSK0012494 | 0.979080721 | bmo-miR-2770-5p|novel-m0786-3p|novel-m1067-3p|novel-m1442-3p|novel-m1679-3p |
| MSTRG.4457.1 | BMSK0013340 | 0.977573661 | bmo-miR-2770-5p|novel-m0786-3p|novel-m1442-3p|novel-m1679-3p |
| MSTRG.4457.1 | BMSK0013484 | 0.95449569 | bmo-miR-2770-5p|bmo-miR-3296-5p|miR-8517-x|novel-m0040-5p|novel-m0284-5p|novel-m0406-5p|novel-m0786-3p|novel-m0848-5p|novel-m0917-5p|novel-m1201-5p|novel-m1442-3p|novel-m1679-3p |
| MSTRG.4457.1 | BMSK0013868 | 0.92048791 | novel-m0040-5p|novel-m0284-5p|novel-m0406-5p|novel-m0848-5p|novel-m0917-5p|novel-m1201-5p |
| MSTRG.463.1 | BMSK0000510 | 0.926685806 | bmo-miR-2770-5p|miR-100-x|novel-m0040-5p|novel-m0284-5p|novel-m0406-5p|novel-m0848-5p|novel-m0917-5p|novel-m1201-5p |
| MSTRG.463.1 | BMSK0000887 | 0.958532757 | novel-m0040-5p|novel-m0284-5p|novel-m0406-5p|novel-m0848-5p|novel-m0917-5p|novel-m1201-5p |
| MSTRG.463.1 | BMSK0000947 | 0.991184041 | novel-m0040-5p|novel-m0284-5p|novel-m0406-5p|novel-m0848-5p|novel-m0917-5p|novel-m1201-5p |
| MSTRG.463.1 | BMSK0002320 | 0.957444773 | miR-100-x|miR-34-x |
| MSTRG.463.1 | BMSK0004077 | 0.984252747 | novel-m0040-5p|novel-m0284-5p|novel-m0406-5p|novel-m0848-5p|novel-m0917-5p|novel-m1201-5p |
| MSTRG.463.1 | BMSK0005228 | 0.974891305 | miR-8517-x|novel-m0040-5p|novel-m0284-5p|novel-m0406-5p|novel-m0848-5p|novel-m0917-5p|novel-m1201-5p |
| MSTRG.463.1 | BMSK0005258 | 0.995540485 | miR-2755-y|miR-8517-x |
| MSTRG.463.1 | BMSK0006077 | 0.92088354 | novel-m0040-5p|novel-m0284-5p|novel-m0406-5p|novel-m0848-5p|novel-m0917-5p|novel-m1201-5p |
| MSTRG.463.1 | BMSK0006349 | 0.926827966 | miR-34-x|novel-m0040-5p|novel-m0284-5p|novel-m0406-5p|novel-m0848-5p|novel-m0917-5p|novel-m1201-5p |
| MSTRG.463.1 | BMSK0007150 | 0.954849395 | bmo-miR-3347a |
| MSTRG.463.1 | BMSK0012727 | 0.991145603 | miR-34-x |
| MSTRG.463.1 | BMSK0013484 | 0.989361209 | bmo-miR-2770-5p|miR-2755-y|miR-8517-x|novel-m0040-5p|novel-m0284-5p|novel-m0406-5p|novel-m0848-5p|novel-m0917-5p|novel-m1201-5p |
| MSTRG.463.1 | BMSK0013768 | 0.982204396 | bmo-miR-3347a |
| MSTRG.463.1 | BMSK0013868 | 0.921374748 | novel-m0040-5p|novel-m0284-5p|novel-m0406-5p|novel-m0848-5p|novel-m0917-5p|novel-m1201-5p |
| MSTRG.463.1 | BMSK0014227 | 0.95839269 | miR-8517-x |
| MSTRG.4937.1 | BMSK0000298 | 0.911969634 | novel-m1505-3p|novel-m1678-3p |
| MSTRG.5154.2 | BMSK0002122 | 0.909146548 | miR-183-x |
| MSTRG.5154.2 | BMSK0006537 | 0.978039944 | novel-m1538-3p|novel-m1541-3p |
| MSTRG.5347.1 | BMSK0012519 | 0.901706886 | novel-m1055-3p |
| MSTRG.6303.1 | BMSK0009652 | 0.947960036 | novel-m0281-5p|novel-m0615-5p|novel-m0740-5p |
| MSTRG.6303.1 | BMSK0012465 | 0.909600008 | novel-m0281-5p|novel-m0615-5p|novel-m0740-5p |
| MSTRG.6403.1 | BMSK0000230 | 0.959079868 | novel-m0395-5p|novel-m0450-5p|novel-m0950-5p|novel-m1175-5p|novel-m1371-5p|novel-m1482-5p|novel-m1668-5p |
| MSTRG.6403.1 | BMSK0000273 | 0.981067066 | novel-m0395-5p|novel-m0450-5p|novel-m0950-5p|novel-m1175-5p|novel-m1371-5p|novel-m1482-5p|novel-m1668-5p |
| MSTRG.6403.1 | BMSK0001086 | 0.923827585 | novel-m0395-5p|novel-m0450-5p|novel-m0950-5p|novel-m1175-5p|novel-m1371-5p|novel-m1482-5p|novel-m1668-5p |
| MSTRG.6403.1 | BMSK0001285 | 0.988016208 | novel-m0395-5p|novel-m0450-5p|novel-m0950-5p|novel-m1175-5p|novel-m1371-5p|novel-m1482-5p|novel-m1668-5p |
| MSTRG.6403.1 | BMSK0001933 | 0.995983718 | novel-m0395-5p|novel-m0450-5p|novel-m0950-5p|novel-m1175-5p|novel-m1371-5p|novel-m1482-5p|novel-m1668-5p |
| MSTRG.6403.1 | BMSK0002030 | 0.905040952 | novel-m0395-5p|novel-m0450-5p|novel-m0950-5p|novel-m1175-5p|novel-m1371-5p|novel-m1482-5p|novel-m1668-5p |
| MSTRG.6403.1 | BMSK0002736 | 0.945287081 | novel-m0395-5p|novel-m0450-5p|novel-m0950-5p|novel-m1175-5p|novel-m1371-5p|novel-m1482-5p|novel-m1668-5p |
| MSTRG.6403.1 | BMSK0002866 | 0.9810143 | novel-m0395-5p|novel-m0450-5p|novel-m0950-5p|novel-m1175-5p|novel-m1371-5p|novel-m1482-5p|novel-m1668-5p |
| MSTRG.6403.1 | BMSK0004663 | 0.97202757 | novel-m0395-5p|novel-m0450-5p|novel-m0950-5p|novel-m1175-5p|novel-m1371-5p|novel-m1482-5p|novel-m1668-5p |
| MSTRG.6403.1 | BMSK0005711 | 0.939550876 | novel-m0395-5p|novel-m0450-5p|novel-m0950-5p|novel-m1175-5p|novel-m1371-5p|novel-m1482-5p|novel-m1668-5p |
| MSTRG.6403.1 | BMSK0006524 | 0.901207646 | novel-m0395-5p|novel-m0450-5p|novel-m0950-5p|novel-m1175-5p|novel-m1371-5p|novel-m1482-5p|novel-m1668-5p |
| MSTRG.6403.1 | BMSK0006528 | 0.910963005 | novel-m0395-5p|novel-m0450-5p|novel-m0950-5p|novel-m1175-5p|novel-m1371-5p|novel-m1482-5p|novel-m1668-5p |
| MSTRG.6403.1 | BMSK0006982 | 0.950332778 | bmo-miR-2770-5p|novel-m0395-5p|novel-m0450-5p|novel-m0950-5p|novel-m1175-5p|novel-m1371-5p|novel-m1482-5p|novel-m1668-5p |
| MSTRG.6403.1 | BMSK0007637 | 0.986598866 | novel-m0395-5p|novel-m0450-5p|novel-m0950-5p|novel-m1175-5p|novel-m1371-5p|novel-m1482-5p|novel-m1668-5p |
| MSTRG.6403.1 | BMSK0008518 | 0.967449955 | novel-m0395-5p|novel-m0450-5p|novel-m0950-5p|novel-m1175-5p|novel-m1371-5p|novel-m1482-5p|novel-m1668-5p |
| MSTRG.6403.1 | BMSK0009032 | 0.998211951 | bmo-miR-3328|miR-10-x |
| MSTRG.6403.1 | BMSK0009085 | 0.991868085 | bmo-miR-2770-5p|novel-m0395-5p|novel-m0450-5p|novel-m0950-5p|novel-m1175-5p|novel-m1371-5p|novel-m1482-5p|novel-m1668-5p |
| MSTRG.6403.1 | BMSK0013340 | 0.995190756 | bmo-miR-2770-5p|novel-m0395-5p|novel-m0450-5p|novel-m0950-5p|novel-m1175-5p|novel-m1371-5p|novel-m1482-5p|novel-m1668-5p |
| MSTRG.6403.1 | BMSK0013484 | 0.989662054 | bmo-miR-2770-5p|novel-m0395-5p|novel-m0450-5p|novel-m0950-5p|novel-m1175-5p|novel-m1371-5p|novel-m1482-5p|novel-m1668-5p |
| MSTRG.6403.1 | BMSK0013868 | 0.922457511 | novel-m0395-5p|novel-m0450-5p|novel-m0950-5p|novel-m1175-5p|novel-m1371-5p|novel-m1482-5p|novel-m1668-5p |
| MSTRG.6580.1 | BMSK0000230 | 0.968185989 | bmo-miR-3347a|novel-m0395-5p|novel-m0450-5p|novel-m0950-5p|novel-m1175-5p|novel-m1371-5p|novel-m1482-5p|novel-m1668-5p |
| MSTRG.6580.1 | BMSK0000273 | 0.978543067 | novel-m0395-5p|novel-m0450-5p|novel-m0950-5p|novel-m1175-5p|novel-m1371-5p|novel-m1482-5p|novel-m1668-5p |
| MSTRG.6580.1 | BMSK0001285 | 0.984294349 | novel-m0395-5p|novel-m0450-5p|novel-m0950-5p|novel-m1175-5p|novel-m1371-5p|novel-m1482-5p|novel-m1668-5p |
| MSTRG.6580.1 | BMSK0001933 | 0.984535043 | novel-m0395-5p|novel-m0450-5p|novel-m0950-5p|novel-m1175-5p|novel-m1371-5p|novel-m1482-5p|novel-m1668-5p |
| MSTRG.6580.1 | BMSK0002030 | 0.942190286 | novel-m0395-5p|novel-m0450-5p|novel-m0950-5p|novel-m1175-5p|novel-m1371-5p|novel-m1482-5p|novel-m1668-5p |
| MSTRG.6580.1 | BMSK0002638 | 0.921858735 | novel-m0395-5p|novel-m0450-5p|novel-m0950-5p|novel-m1175-5p|novel-m1371-5p|novel-m1482-5p|novel-m1668-5p |
| MSTRG.6580.1 | BMSK0002736 | 0.943004149 | bmo-miR-3347a|novel-m0395-5p|novel-m0450-5p|novel-m0950-5p|novel-m1175-5p|novel-m1371-5p|novel-m1482-5p|novel-m1668-5p |
| MSTRG.6580.1 | BMSK0002866 | 0.972657369 | novel-m0395-5p|novel-m0450-5p|novel-m0950-5p|novel-m1175-5p|novel-m1371-5p|novel-m1482-5p|novel-m1668-5p |
| MSTRG.6580.1 | BMSK0003353 | 0.904875813 | bmo-miR-3347a|novel-m0395-5p|novel-m0450-5p|novel-m0950-5p|novel-m1175-5p|novel-m1371-5p|novel-m1482-5p|novel-m1668-5p |
| MSTRG.6580.1 | BMSK0004663 | 0.937809391 | novel-m0395-5p|novel-m0450-5p|novel-m0950-5p|novel-m1175-5p|novel-m1371-5p|novel-m1482-5p|novel-m1668-5p |
| MSTRG.6580.1 | BMSK0006524 | 0.938661668 | novel-m0395-5p|novel-m0450-5p|novel-m0950-5p|novel-m1175-5p|novel-m1371-5p|novel-m1482-5p|novel-m1668-5p |
| MSTRG.6580.1 | BMSK0006528 | 0.952082745 | novel-m0395-5p|novel-m0450-5p|novel-m0950-5p|novel-m1175-5p|novel-m1371-5p|novel-m1482-5p|novel-m1668-5p |
| MSTRG.6580.1 | BMSK0006982 | 0.902864367 | novel-m0395-5p|novel-m0450-5p|novel-m0950-5p|novel-m1175-5p|novel-m1371-5p|novel-m1482-5p|novel-m1668-5p |
| MSTRG.6580.1 | BMSK0007150 | 0.983943284 | bmo-miR-3347a |
| MSTRG.6580.1 | BMSK0007637 | 0.965617915 | novel-m0395-5p|novel-m0450-5p|novel-m0950-5p|novel-m1175-5p|novel-m1371-5p|novel-m1482-5p|novel-m1668-5p |
| MSTRG.6580.1 | BMSK0008518 | 0.956782791 | novel-m0395-5p|novel-m0450-5p|novel-m0950-5p|novel-m1175-5p|novel-m1371-5p|novel-m1482-5p|novel-m1668-5p |
| MSTRG.6580.1 | BMSK0009085 | 0.952588926 | novel-m0395-5p|novel-m0450-5p|novel-m0950-5p|novel-m1175-5p|novel-m1371-5p|novel-m1482-5p|novel-m1668-5p |
| MSTRG.6580.1 | BMSK0013340 | 0.967928747 | novel-m0395-5p|novel-m0450-5p|novel-m0950-5p|novel-m1175-5p|novel-m1371-5p|novel-m1482-5p|novel-m1668-5p |
| MSTRG.6580.1 | BMSK0013484 | 0.97855052 | novel-m0395-5p|novel-m0450-5p|novel-m0950-5p|novel-m1175-5p|novel-m1371-5p|novel-m1482-5p|novel-m1668-5p |
| MSTRG.6580.1 | BMSK0013768 | 0.988327902 | bmo-miR-3347a |
| MSTRG.6580.1 | BMSK0013868 | 0.905507716 | novel-m0395-5p|novel-m0450-5p|novel-m0950-5p|novel-m1175-5p|novel-m1371-5p|novel-m1482-5p|novel-m1668-5p |
| MSTRG.6580.1 | BMSK0014377 | 0.980362446 | novel-m0344-3p |
| MSTRG.6626.1 | BMSK0001934 | 0.980347226 | miR-7-x |
| MSTRG.6626.1 | BMSK0006523 | 0.983560902 | miR-7-x |
| MSTRG.6626.1 | BMSK0013484 | 0.994167223 | bmo-miR-2842|novel-m0698-5p |
| MSTRG.6626.1 | BMSK0015675 | 0.989047824 | bmo-miR-3328 |
| MSTRG.678.1 | BMSK0009991 | 0.951347073 | novel-m0667-3p |
| MSTRG.678.1 | BMSK0010293 | 0.947355905 | novel-m0490-3p|novel-m0712-3p|novel-m1323-3p |
| MSTRG.7651.1 | BMSK0000230 | 0.979819876 | novel-m0395-5p|novel-m0450-5p|novel-m0950-5p|novel-m1175-5p|novel-m1371-5p|novel-m1482-5p|novel-m1668-5p |
| MSTRG.7651.1 | BMSK0000273 | 0.930541532 | novel-m0395-5p|novel-m0450-5p|novel-m0950-5p|novel-m1175-5p|novel-m1371-5p|novel-m1482-5p|novel-m1668-5p |
| MSTRG.7651.1 | BMSK0001215 | 0.932125299 | novel-m0395-5p|novel-m0450-5p|novel-m0950-5p|novel-m1175-5p|novel-m1371-5p|novel-m1482-5p|novel-m1668-5p |
| MSTRG.7651.1 | BMSK0001285 | 0.930336165 | novel-m0395-5p|novel-m0450-5p|novel-m0950-5p|novel-m1175-5p|novel-m1371-5p|novel-m1482-5p|novel-m1668-5p |
| MSTRG.7651.1 | BMSK0001933 | 0.954772292 | novel-m0395-5p|novel-m0450-5p|novel-m0950-5p|novel-m1175-5p|novel-m1371-5p|novel-m1482-5p|novel-m1668-5p |
| MSTRG.7651.1 | BMSK0002638 | 0.979970449 | novel-m0395-5p|novel-m0450-5p|novel-m0950-5p|novel-m1175-5p|novel-m1371-5p|novel-m1482-5p|novel-m1668-5p |
| MSTRG.7651.1 | BMSK0002736 | 0.973387661 | novel-m0395-5p|novel-m0450-5p|novel-m0950-5p|novel-m1175-5p|novel-m1371-5p|novel-m1482-5p|novel-m1668-5p | |
| MSTRG.7651.1 | BMSK0002866 | 0.974176793 | novel-m0395-5p|novel-m0450-5p|novel-m0950-5p|novel-m1175-5p|novel-m1371-5p|novel-m1482-5p|novel-m1668-5p | |
| MSTRG.7651.1 | BMSK0003353 | 0.908676949 | novel-m0395-5p|novel-m0450-5p|novel-m0950-5p|novel-m1175-5p|novel-m1371-5p|novel-m1482-5p|novel-m1668-5p | |
| MSTRG.7651.1 | BMSK0004663 | 0.92836171 | novel-m0395-5p|novel-m0450-5p|novel-m0950-5p|novel-m1175-5p|novel-m1371-5p|novel-m1482-5p|novel-m1668-5p | |
| MSTRG.7651.1 | BMSK0006524 | 0.986941673 | novel-m0395-5p|novel-m0450-5p|novel-m0950-5p|novel-m1175-5p|novel-m1371-5p|novel-m1482-5p|novel-m1668-5p | |
| MSTRG.7651.1 | BMSK0006982 | 0.920217349 | novel-m0395-5p|novel-m0450-5p|novel-m0950-5p|novel-m1175-5p|novel-m1371-5p|novel-m1482-5p|novel-m1668-5p | |
| MSTRG.7651.1 | BMSK0007637 | 0.959675482 | novel-m0395-5p|novel-m0450-5p|novel-m0950-5p|novel-m1175-5p|novel-m1371-5p|novel-m1482-5p|novel-m1668-5p | |
| MSTRG.7651.1 | BMSK0008518 | 0.972046832 | novel-m0395-5p|novel-m0450-5p|novel-m0950-5p|novel-m1175-5p|novel-m1371-5p|novel-m1482-5p|novel-m1668-5p | |
| MSTRG.7651.1 | BMSK0009085 | 0.908008699 | novel-m0395-5p|novel-m0450-5p|novel-m0950-5p|novel-m1175-5p|novel-m1371-5p|novel-m1482-5p|novel-m1668-5p | |
| MSTRG.7651.1 | BMSK0010061 | 0.982549256 | bmo-miR-3384-3p | |
| MSTRG.7651.1 | BMSK0013340 | 0.922405358 | novel-m0395-5p|novel-m0450-5p|novel-m0950-5p|novel-m1175-5p|novel-m1371-5p|novel-m1482-5p|novel-m1668-5p | |
| MSTRG.7651.1 | BMSK0013484 | 0.939686011 | bmo-miR-2842|novel-m0395-5p|novel-m0450-5p|novel-m0950-5p|novel-m1175-5p|novel-m1371-5p|novel-m1482-5p|novel-m1668-5p | |
| MSTRG.7651.1 | BMSK0013868 | 0.952432103 | novel-m0395-5p|novel-m0450-5p|novel-m0950-5p|novel-m1175-5p|novel-m1371-5p|novel-m1482-5p|novel-m1668-5p | |
| MSTRG.7651.2 | BMSK0000230 | 0.901734484 | novel-m0395-5p|novel-m0450-5p|novel-m0950-5p|novel-m1175-5p|novel-m1371-5p|novel-m1482-5p|novel-m1668-5p | |
| MSTRG.7651.2 | BMSK0000273 | 0.962390065 | novel-m0395-5p|novel-m0450-5p|novel-m0950-5p|novel-m1175-5p|novel-m1371-5p|novel-m1482-5p|novel-m1668-5p | |
| MSTRG.7651.2 | BMSK0001086 | 0.971925233 | novel-m0395-5p|novel-m0450-5p|novel-m0950-5p|novel-m1175-5p|novel-m1371-5p|novel-m1482-5p|novel-m1668-5p | |
| MSTRG.7651.2 | BMSK0001285 | 0.948434362 | novel-m0395-5p|novel-m0450-5p|novel-m0950-5p|novel-m1175-5p|novel-m1371-5p|novel-m1482-5p|novel-m1668-5p | |
| MSTRG.7651.2 | BMSK0001933 | 0.981140173 | novel-m0395-5p|novel-m0450-5p|novel-m0950-5p|novel-m1175-5p|novel-m1371-5p|novel-m1482-5p|novel-m1668-5p | |
| MSTRG.7651.2 | BMSK0002736 | 0.939705351 | novel-m0395-5p|novel-m0450-5p|novel-m0950-5p|novel-m1175-5p|novel-m1371-5p|novel-m1482-5p|novel-m1668-5p | |
| MSTRG.7651.2 | BMSK0002866 | 0.944744533 | novel-m0395-5p|novel-m0450-5p|novel-m0950-5p|novel-m1175-5p|novel-m1371-5p|novel-m1482-5p|novel-m1668-5p | |
| MSTRG.7651.2 | BMSK0004663 | 0.964572672 | novel-m0395-5p|novel-m0450-5p|novel-m0950-5p|novel-m1175-5p|novel-m1371-5p|novel-m1482-5p|novel-m1668-5p | |
| MSTRG.7651.2 | BMSK0005711 | 0.942091009 | novel-m0395-5p|novel-m0450-5p|novel-m0950-5p|novel-m1175-5p|novel-m1371-5p|novel-m1482-5p|novel-m1668-5p | |
| MSTRG.7651.2 | BMSK0006982 | 0.950041816 | novel-m0395-5p|novel-m0450-5p|novel-m0950-5p|novel-m1175-5p|novel-m1371-5p|novel-m1482-5p|novel-m1668-5p | |
| MSTRG.7651.2 | BMSK0007637 | 0.968878369 | novel-m0395-5p|novel-m0450-5p|novel-m0950-5p|novel-m1175-5p|novel-m1371-5p|novel-m1482-5p|novel-m1668-5p | |
| MSTRG.7651.2 | BMSK0008518 | 0.952048603 | novel-m0395-5p|novel-m0450-5p|novel-m0950-5p|novel-m1175-5p|novel-m1371-5p|novel-m1482-5p|novel-m1668-5p | |
| MSTRG.7651.2 | BMSK0009085 | 0.980477059 | novel-m0395-5p|novel-m0450-5p|novel-m0950-5p|novel-m1175-5p|novel-m1371-5p|novel-m1482-5p|novel-m1668-5p | |
| MSTRG.7651.2 | BMSK0013340 | 0.970688969 | novel-m0395-5p|novel-m0450-5p|novel-m0950-5p|novel-m1175-5p|novel-m1371-5p|novel-m1482-5p|novel-m1668-5p | |
| MSTRG.7651.2 | BMSK0013484 | 0.936804022 | bmo-miR-2842|novel-m0395-5p|novel-m0450-5p|novel-m0950-5p|novel-m1175-5p|novel-m1371-5p|novel-m1482-5p|novel-m1668-5p | |
| MSTRG.7651.2 | BMSK0013868 | 0.911142659 | novel-m0395-5p|novel-m0450-5p|novel-m0950-5p|novel-m1175-5p|novel-m1371-5p|novel-m1482-5p|novel-m1668-5p | |
| MSTRG.8021.1 | BMSK0002809 | 0.942720887 | novel-m0373-5p | |
| MSTRG.8021.1 | BMSK0005379 | 0.929047889 | miR-217-x | |
| MSTRG.8021.1 | BMSK0005879 | 0.934420734 | novel-m0401-3p | |
| MSTRG.8021.1 | BMSK0010293 | 0.932537548 | novel-m0373-5p|novel-m0401-3p | |
| MSTRG.8021.1 | BMSK0010795 | 0.910917147 | miR-217-x|novel-m0401-3p | |
| MSTRG.8632.1 | BMSK0001796 | 0.984411361 | bmo-miR-2813 | |
| MSTRG.8632.1 | BMSK0002809 | 0.982895761 | novel-m0373-5p | |
| MSTRG.8632.1 | BMSK0004306 | 0.980569764 | bmo-miR-2813 | |
| MSTRG.8632.1 | BMSK0013626 | 0.97998714 | bmo-miR-2813 | |
| MSTRG.9283.1 | BMSK0000230 | 0.981862109 | novel-m0395-5p|novel-m0450-5p|novel-m0950-5p|novel-m1175-5p|novel-m1371-5p|novel-m1482-5p|novel-m1668-5p | |
| MSTRG.9283.1 | BMSK0000273 | 0.972133751 | novel-m0395-5p|novel-m0450-5p|novel-m0950-5p|novel-m1175-5p|novel-m1371-5p|novel-m1482-5p|novel-m1668-5p | |
| MSTRG.9283.1 | BMSK0001285 | 0.977224069 | novel-m0395-5p|novel-m0450-5p|novel-m0950-5p|novel-m1175-5p|novel-m1371-5p|novel-m1482-5p|novel-m1668-5p | |
| MSTRG.9283.1 | BMSK0001933 | 0.992189991 | novel-m0395-5p|novel-m0450-5p|novel-m0950-5p|novel-m1175-5p|novel-m1371-5p|novel-m1482-5p|novel-m1668-5p | |
| MSTRG.9283.1 | BMSK0002638 | 0.941078074 | novel-m0395-5p|novel-m0450-5p|novel-m0950-5p|novel-m1175-5p|novel-m1371-5p|novel-m1482-5p|novel-m1668-5p | |
| MSTRG.9283.1 | BMSK0002736 | 0.970120588 | novel-m0395-5p|novel-m0450-5p|novel-m0950-5p|novel-m1175-5p|novel-m1371-5p|novel-m1482-5p|novel-m1668-5p | |
| MSTRG.9283.1 | BMSK0002866 | 0.992380827 | bmo-miR-3001|novel-m0395-5p|novel-m0450-5p|novel-m0950-5p|novel-m1175-5p|novel-m1371-5p|novel-m1482-5p|novel-m1668-5p | |
| MSTRG.9283.1 | BMSK0004276 | 0.936203499 | miR-192-x|miR-215-x | |
| MSTRG.9283.1 | BMSK0004663 | 0.968590253 | novel-m0395-5p|novel-m0450-5p|novel-m0950-5p|novel-m1175-5p|novel-m1371-5p|novel-m1482-5p|novel-m1668-5p | |
| MSTRG.9283.1 | BMSK0006524 | 0.949882082 | novel-m0395-5p|novel-m0450-5p|novel-m0950-5p|novel-m1175-5p|novel-m1371-5p|novel-m1482-5p|novel-m1668-5p | |
| MSTRG.9283.1 | BMSK0006528 | 0.90470704 | novel-m0395-5p|novel-m0450-5p|novel-m0950-5p|novel-m1175-5p|novel-m1371-5p|novel-m1482-5p|novel-m1668-5p | |
| MSTRG.9283.1 | BMSK0006982 | 0.951535995 | bmo-miR-3001|novel-m0395-5p|novel-m0450-5p|novel-m0950-5p|novel-m1175-5p|novel-m1371-5p|novel-m1482-5p|novel-m1668-5p | |
| MSTRG.9283.1 | BMSK0007637 | 0.98776545 | novel-m0395-5p|novel-m0450-5p|novel-m0950-5p|novel-m1175-5p|novel-m1371-5p|novel-m1482-5p|novel-m1668-5p | |
| MSTRG.9283.1 | BMSK0008518 | 0.983391484 | novel-m0395-5p|novel-m0450-5p|novel-m0950-5p|novel-m1175-5p|novel-m1371-5p|novel-m1482-5p|novel-m1668-5p | |
| MSTRG.9283.1 | BMSK0009085 | 0.969588112 | novel-m0395-5p|novel-m0450-5p|novel-m0950-5p|novel-m1175-5p|novel-m1371-5p|novel-m1482-5p|novel-m1668-5p | | | |
| MSTRG.9283.1 | BMSK0012319 | 0.972325443 | bmo-miR-3001 | | | |
| MSTRG.9283.1 | BMSK0013340 | 0.97753186 | novel-m0395-5p|novel-m0450-5p|novel-m0950-5p|novel-m1175-5p|novel-m1371-5p|novel-m1482-5p|novel-m1668-5p | | | |
| MSTRG.9283.1 | BMSK0013484 | 0.981481722 | bmo-miR-2842|novel-m0395-5p|novel-m0450-5p|novel-m0950-5p|novel-m1175-5p|novel-m1371-5p|novel-m1482-5p|novel-m1668-5p | | | |
| MSTRG.9283.1 | BMSK0013868 | 0.948525102 | novel-m0395-5p|novel-m0450-5p|novel-m0950-5p|novel-m1175-5p|novel-m1371-5p|novel-m1482-5p|novel-m1668-5p | | | |
| MSTRG.9758.1 | BMSK0002127 | 0.968686266 | bmo-miR-308-3p | | | |
| MSTRG.9758.1 | BMSK0002623 | 0.988677612 | bmo-miR-308-3p | | | |
| MSTRG.9758.1 | BMSK0002664 | 0.943260389 | bmo-miR-308-3p | | | |
| MSTRG.9758.1 | BMSK0008516 | 0.923113577 | bmo-miR-308-3p | | | |
| MSTRG.9758.1 | BMSK0012866 | 0.970217592 | bmo-miR-308-3p | | | |
| MSTRG.9758.1 | BMSK0013371 | 0.934561059 | bmo-miR-308-3p | | | |
| MSTRG.9758.1 | BMSK0013626 | 0.947736806 | bmo-miR-308-3p | | | |
| MSTRG.9896.1 | BMSK0000230 | 0.917283003 | novel-m0395-5p|novel-m0450-5p|novel-m0950-5p|novel-m1175-5p|novel-m1371-5p|novel-m1482-5p|novel-m1668-5p | | | |
| MSTRG.9896.1 | BMSK0001215 | 0.930354465 | novel-m0348-5p|novel-m0395-5p|novel-m0411-5p|novel-m0450-5p|novel-m0496-3p|novel-m0753-5p|novel-m0950-5p|novel-m1039-5p|novel-m1175-5p|novel-m1371-5p|novel-m1482-5p|novel-m1668-5p | | | |
| MSTRG.9896.1 | BMSK0001390 | 0.935290679 | miR-25-y|miR-92-y | | | |
| MSTRG.9896.1 | BMSK0002638 | 0.941489488 | novel-m0338-5p|novel-m0395-5p|novel-m0450-5p|novel-m0950-5p|novel-m1175-5p|novel-m1371-5p|novel-m1482-5p|novel-m1566-5p|novel-m1668-5p | | | |
| MSTRG.9896.1 | BMSK0003353 | 0.967530925 | novel-m0395-5p|novel-m0450-5p|novel-m0950-5p|novel-m1175-5p|novel-m1371-5p|novel-m1482-5p|novel-m1668-5p | | | |
| MSTRG.9896.1 | BMSK0004519 | 0.913723906 | novel-m0338-5p|novel-m1566-5p | | | |
| MSTRG.9896.1 | BMSK0006524 | 0.973084214 | novel-m0395-5p|novel-m0450-5p|novel-m0950-5p|novel-m1175-5p|novel-m1371-5p|novel-m1482-5p|novel-m1668-5p | | | |

| **Table S12 CircRNA-miRNA-mRNA networks of silkworm in the 5th day of *N. bombycis* congenitally infected silkworm embryos** | | | |
| --- | --- | --- | --- |
| ncRNA_ID | mRNA_ID | corr | miRNA_ID(both) |
| novel_circ_000107 | BMSK0003200 | 0.932653092 | bmo-miR-2774b|miR-516-x |
| novel_circ_000293 | BMSK0000611 | 0.931027748 | miR-8517-x |
| novel_circ_000293 | BMSK0006523 | 0.912278426 | miR-765-y |
| novel_circ_000293 | BMSK0014227 | 0.930576385 | miR-8517-x |
| novel_circ_000352 | BMSK0009481 | 0.992324023 | miR-3759-y |
| novel_circ_000733 | BMSK0009275 | 0.967776098 | novel-m1026-5p|novel-m1027-5p|novel-m1028-5p |
| novel_circ_000733 | BMSK0012455 | 0.927085649 | novel-m0304-3p|novel-m0496-3p |
| novel_circ_000847 | BMSK0008516 | 0.900420174 | bmo-miR-308-3p |
| novel_circ_000847 | BMSK0012519 | 0.946013147 | novel-m1549-3p|novel-m1600-3p |
| novel_circ_000891 | BMSK0000298 | 0.920156807 | miR-971-y|novel-m1232-5p |
| novel_circ_000891 | BMSK0010949 | 0.907100477 | novel-m0557-3p |
| novel_circ_001156 | BMSK0005258 | 0.940516605 | miR-998-y |
| novel_circ_001979 | BMSK0003850 | 0.947191676 | miR-8517-x |
| novel_circ_001979 | BMSK0006522 | 0.94163589 | miR-8517-x |
| novel_circ_002017 | BMSK0006349 | 0.943331893 | novel-m0040-5p|novel-m0284-5p|novel-m0406-5p|novel-m0848-5p|novel-m0917-5p|novel-m1201-5p |
| novel_circ_002096 | BMSK0002226 | 0.942941165 | bmo-miR-2813|bmo-miR-2817|novel-m1549-3p|novel-m1600-3p |
| novel_circ_002096 | BMSK0002454 | 0.987872276 | bmo-miR-2817|novel-m0745-5p|novel-m0779-5p|novel-m1184-3p|novel-m1276-3p|novel-m1678-3p |
| novel_circ_002096 | BMSK0004306 | 0.964313972 | bmo-miR-2813|miR-3316-y |
| novel_circ_002096 | BMSK0007700 | 0.911903185 | bmo-miR-2738|novel-m1184-3p|novel-m1276-3p|novel-m1276-5p |
| novel_circ_002096 | BMSK0010080 | 0.933948507 | miR-10477-y|novel-m0490-3p|novel-m0712-3p|novel-m1276-3p|novel-m1276-5p|novel-m1323-3p |
| novel_circ_002096 | BMSK0010293 | 0.973837887 | bmo-miR-2817|miR-10477-y|miR-3267-y|miR-3316-y|novel-m0373-5p|novel-m0401-3p|novel-m0463-5p|novel-m0490-3p|novel-m0712-3p|novel-m1323-3p |
| novel_circ_002096 | BMSK0012199 | 0.982766289 | bmo-miR-2738|novel-m0281-5p|novel-m0363-5p|novel-m0615-5p|novel-m0740-5p |
| novel_circ_002096 | BMSK0012465 | 0.956437447 | miR-3267-y|miR-338-y|novel-m0281-5p|novel-m0401-3p|novel-m0463-5p|novel-m0615-5p|novel-m0740-5p |
| novel_circ_002096 | BMSK0014031 | 0.91536015 | miR-3267-y|novel-m0557-3p|novel-m0745-5p|novel-m0779-5p|novel-m1276-3p |
| novel_circ_002096 | BMSK0014140 | 0.919511401 | miR-3267-y|novel-m0452-3p |
| novel_circ_002114 | BMSK0001934 | 0.913579168 | miR-7-x |
| novel_circ_002114 | BMSK0006523 | 0.94735469 | miR-7-x |
| novel_circ_002619 | BMSK0001401 | 0.918137433 | miR-278-x|miR-7-x |
| novel_circ_002619 | BMSK0009752 | 0.919540113 | novel-m1184-5p |
| novel_circ_002762 | BMSK0001401 | 0.984406103 | novel-m0395-5p|novel-m0450-5p|novel-m0950-5p|novel-m1175-5p|novel-m1371-5p|novel-m1482-5p|novel-m1668-5p |
| novel_circ_002762 | BMSK0002030 | 0.917808052 | novel-m0395-5p|novel-m0450-5p|novel-m0950-5p|novel-m1175-5p|novel-m1371-5p|novel-m1482-5p|novel-m1668-5p |
| novel_circ_002762 | BMSK0006528 | 0.915817528 | novel-m0395-5p|novel-m0450-5p|novel-m0737-5p|novel-m0950-5p|novel-m1175-5p|novel-m1227-5p|novel-m1371-5p|novel-m1482-5p|novel-m1668-5p |
| novel_circ_002762 | BMSK0007084 | 0.996598899 | novel-m0395-5p|novel-m0450-5p|novel-m0950-5p|novel-m1175-5p|novel-m1371-5p|novel-m1482-5p|novel-m1668-5p |
| novel_circ_003600 | BMSK0002866 | 0.911299789 | novel-m1184-5p |
| novel_circ_003600 | BMSK0013098 | 0.904824904 | novel-m1184-5p |
| novel_circ_003603 | BMSK0012199 | 0.920182881 | bmo-miR-308-3p |
| novel_circ_003603 | BMSK0013371 | 0.90571557 | bmo-miR-308-3p |
| novel_circ_004489 | BMSK0009085 | 0.904827507 | novel-m0737-5p|novel-m1184-5p|novel-m1227-5p |
| novel_circ_004489 | BMSK0012494 | 0.916204608 | novel-m0737-5p|novel-m1184-5p|novel-m1227-5p |
| novel_circ_004592 | BMSK0001110 | 0.94137243 | novel-m0736-5p|novel-m1464-3p|novel-m1554-3p|novel-m1597-3p |
| novel_circ_004592 | BMSK0007988 | 0.980685849 | novel-m0736-5p|novel-m1464-3p|novel-m1554-3p|novel-m1597-3p |
| novel_circ_004592 | BMSK0008332 | 0.907673947 | novel-m0736-5p|novel-m1464-3p|novel-m1554-3p|novel-m1597-3p |
| novel_circ_004592 | BMSK0010293 | 0.921713928 | novel-m0329-3p|novel-m0736-5p|novel-m1464-3p|novel-m1554-3p|novel-m1597-3p |
| novel_circ_004592 | BMSK0014140 | 0.944639646 | novel-m0452-3p |
| novel_circ_004870 | BMSK0005264 | 0.969283984 | miR-126-x|novel-m0330-5p |
| novel_circ_004906 | BMSK0013098 | 0.943423096 | novel-m1057-5p |
| novel_circ_005993 | BMSK0001898 | 0.92599607 | miR-2808-y|miR-3316-y|novel-m0777-5p |
[truncated: 176,347 more chars]
